# Infective Endocarditis in North Africa and the Middle East, 1990‒2019: Updates from the Global Burden of Disease Study 2019

**DOI:** 10.34172/aim.2024.34

**Published:** 2024-05-01

**Authors:** Elaheh Malakan Rad, Sara Momtazmanesh, Sahar Saeedi Moghaddam, Negar Rezaei, Nazila Rezaei, Hamidreza Jamshidi, Mohsen Naghavi, Bagher Larijani, Farshad Farzadfar, GBD 2019 NAME Endocarditis Collaborators

**Affiliations:** ^1^Department of Pediatric Cardiology, Tehran University of Medical Sciences, Tehran, Iran; ^2^School of Medicine, Tehran University of Medical Sciences, Tehran, Iran; ^3^Non-Communicable Diseases Research Center, Endocrinology and Metabolism Population Sciences Institute, Tehran University of Medical Sciences, Tehran, Iran; ^4^Kiel Institute for the World Economy, Kiel, Germany; ^5^Endocrinology and Metabolism Research Center, Endocrinology and Metabolism Clinical Sciences Institute, Tehran University of Medical Sciences, Tehran, Iran; ^6^Department of Pharmacology, Shahid Beheshti University of Medical Sciences, Tehran, Iran; ^7^Ministry of Health and Medical Education, Tehran, Iran; ^8^Institute for Health Metrics and Evaluation, University of Washington, Seattle, WA, USA; ^9^Department of Health Metrics Sciences, School of Medicine, University of Washington, Seattle, WA, USA

**Keywords:** Africa, Northern, Burden of disease, Endocarditis, Epidemiology, Middle East

## Abstract

**Background:**

Infective endocarditis (IE), a severe and economically impactful condition, lacks substantial epidemiological data in the North Africa and Middle East (NAME) region. This study focused on analyzing the trends and burden of IE in NAME from 1990 to 2019, taking into account factors like age, gender, and socio-demographic index (SDI).

**Methods:**

The Global Burden of Disease data from 1990 to 2019 was retrieved from the Institute for Health Metrics and Evaluation (IHME) website.

**Results:**

Between 1990 and 2019, the age-standardized rates (ASR) for IE incidence increased by 59%, and prevalence and years lived with disability (YLDs) rose by 12% and 9%, respectively, while the ASRs for deaths, disability-adjusted life years (DALYs), and years of life lost (YLLs) saw reductions of 22%, 34%, and 34% in the NAME region. Death rates among children under five declined by 72%. Gender and the SDI did not significantly influence these changes. Saudi Arabia witnessed the most significant increase in ASR of IE incidence since 1990, while Turkey had the highest rates in 2019. The year 2019 also saw the highest death rate among those aged 70 and over, with over 91000 DALYs from IE. DALYs decreased by 71.5% for children under five from 1990 to 2019 but remained stable for individuals in their seventies. Jordan showed the most notable decrease in ASRs for deaths, DALYs, and YLLs among children under five.

**Conclusion:**

This study highlights the changing epidemiology of IE in the NAME region, recommending the establishment of multidisciplinary IE registries, antibiotic prophylaxis guidelines for healthcare-associated IE, and strategies to control antimicrobial resistance as key mitigation measures.

## Introduction

 Infective endocarditis (IE) is a potentially fatal condition with serious cardiac and extracardiac complications, an average in-hospital mortality rate of 20%, and a high economic burden with hospitalization costs ranging from $37 000 to $55 000 per patient on average.^1-5^ Recent decades have seen significant changes in risk factors, microorganisms, antibacterial resistance, diagnostic criteria, techniques, and therapeutic modalities for this disease.^6-13^ The North Africa and Middle East (NAME) region, with over 470 million people in 2021 and 21 countries with diverse and dynamic socio-demographic and progress levels, has a global significance out of proportion to its size, with the population doubling in 2019 compared to 1990.^14,15^ The goal of this study was to look at the epidemiologic measures and burden of IE in NAME and its 21 countries from 1990 to 2019 by country socio-demographic index (SDI), age groups and gender, and to compare trends over three time periods when the American Heart Association (AHA) issued different antibiotic prophylaxis (AP) recommendations.^16-18^

## Materials and Methods

###  Overview of the Global Burden of Disease Study

 The Global Burden of Disease (GBD) 2019 is an international collaborative study that estimates the burden of 369 diseases and injuries and 87 risk factors in 204 countries and territories since 1990. These countries and territories are grouped into 21 regions and seven super-regions. The methodology of the GBD 2019 study is reported elsewhere.^19^ The estimation process for infective IE is described in Supplementary file 1.

###  Data Resources

 The Institute for Health Metrics and Evaluation (IHME) provided the 1990–2019 GBD data. The GBD online results tool and GBD compare tool allow interactive viewing of the data under the diagnosis code B.2.11 in the “Cause” section. The definition of endocarditis is established based on clinical criteria. The 10th revision of the International Classification of Disease (ICD-10) codes that were linked to the Global Burden of Disease for endocarditis as a cause of mortality encompassed the ranges I33–I33.9 and I38–I39.9.^19-22^

####  Estimation of Non-fatal Health Outcome and Cause of Death Using Modeling Strategies for the Global Burden of Endocarditis 

 The IHME employed robust modeling strategies and statistical methods to estimate non-fatal health outcomes and causes of death in order to assess the global burden of endocarditis. The following summary is derived from the extensive methodology appendix in the original sources published in the *Lancet* journal.^19^

 For estimation of non-fatal measures (incidence, prevalence, and years lived with disability [YLD]), the input data consisted of model inputs that encompassed a revised systematic review of the GBD 2015, hospital inpatient data, and claims data. The estimation of endocarditis for GBD 2019 was conducted using DisMod-MR 2.1. This method is extensively explained elsewhere.^23^ The combination of prevalence and incidence of causes and sequelae with levels of severity related to disability (by using disability weights) while correcting for comorbidity resulted in the YLD calculation. The evaluation of models conducted by IHME involved a comparison of the model’s fit with both the available data and the outcomes obtained from previous estimation cycles. Figure S1 in Supplementary file 2 illustrates the flow chart pertaining to the estimation of non-fatal health outcomes.

 The assessment of mortality related to endocarditis involved using vital registration data to create a model specific to this condition. After addressing issues related to data quality, the Global Burden of Disease study in 2019 employed various statistical techniques to determine the number of deaths associated with each distinct cause. This process was executed using the Cause of Death Ensemble model algorithm (CODEm).^24^ In order to prevent the occurrence of a situation where the cumulative number of deaths attributed to various causes surpasses the overall estimated number of deaths, a correction method known as CoDCorrect was employed.^25^

 YLL were calculated by multiplying the estimated number of deaths by the standard life expectancy at the age of death. Figure S2 displays the flow chart outlining the process for estimating endocarditis as a potential cause of death. Lastly, the index pages containing statistical, analytical, processing, and estimation code for GBD 2019 can be found in alternative sources.^26^

 The Guidelines for Accurate and Transparent Health Estimates Reporting (GATHER) were followed.^27^

 Afghanistan, Algeria, Bahrain, Egypt, Iran (the Islamic Republic of), Iraq, Jordan, Kuwait, Lebanon, Libya, Morocco, Oman, Palestine, Qatar, Saudi Arabia, Sudan, the Syrian Arab Republic, Tunisia, Turkey, United Arab Emirates, and Yemen comprise NAME. The SDI summarizes “average income per person, educational attainment, and total fertility rate,” with higher numbers indicating higher socio-economic development. NAME region countries were classified into high, high-middle, middle, low-middle, and low SDI. In accordance with AHA guideline updates, we divided the time period from 1990 to 2019 into three eras: the first (1990–1997), the second (1998–2007), and the third (2008–2019).

###  Decomposition Analysis

 We conducted decomposition analysis on the 1990‒2019 incidence rate of IE to identify the factors that influenced it, following the approach used in previous GBD studies.^28^ Factors such as population growth, aging, and age-specific IE incidence rates drive the trend. The study estimated age-specific IE incidence rate changes while controlling for population size and age structure. The expected IE incidence in 2019 was calculated in two phases: accounting for population growth by applying 1990 age-specific IE incidence rates to 2019 population size, and accounting for both population growth and age structure change by applying 1990 age structure and age-specific IE incidence rates to the 2019 population size and age structure.

###  Statistical Analysis

 We used the R statistical package version 4.0.4 to analyze data, ensuring that all values were within a 95% uncertainty interval (UI) calculated from the 25th and 975th ordered values between 1000 draws in each computational step. No overlap between UIs or opposing signs indicated statistical significance.

Supplementary files 2 and 3 contain supplementary figures and tables.

## Results

###  Incidence

 In 2019, there were 81.5 thousand (67‒98) new cases of IE in NAME, which showed a 144% rise compared with 1990. Decomposition analysis revealed that 58.8% of this increase was due to the rise in incidence rate rather than population growth or aging (Tables 1-4). Age-standardized rate (ASR) increased by 34% (31 to 37%) in 2019, reaching 15.4 per 100 000 (95% UI: 12.9‒18.2). Age-standardized and all-age incidence rates of IE were not significantly influenced by gender (Figures S3-S5). Furthermore, the number of new IE cases was unrelated to the SDI type.

**Table 1:** All-Ages Number, All-Ages Rate, and Age-Standardized Rate of Incidence, Prevalence, Deaths, DALYs, YLLs, and of Infective Endocarditis in 1990 and 2019 in NAME with 95% Uncertainty Intervals

**Measures**	**Metrics**	**Years**					
		**1990**			**2019**		
		**Both**	**Female**	**Male**	**Both**	**Female**	**Male**
**Incidence**	AA number	33371.03 (27156.84-40600.00)	14854.05 (12.52.64-18098.11)	18516.98 (15017.98-22528.29)	81527.94 (67012.50-97904.88)	36487.94 (30067.58-43692.66)	45039.20 (36874.62-54348.41)
	AA rate	9.67(7.87-11.77)	8.83(7.16-10.75)	10.48 (8.50-12.75)	13.39 (11.01-16.08)	12.47 (10.28-14.94)	14.21 (11.66-17.19)
	ASR	11.47(9.62-13.59)	10.37(8.68-12.30)	12.51 (10.47-14.88)	15.35 (12.89-18.20)	14.48 (12.16-17.08)	16.12 (13.49- 19.21)
**Prevalence **	AA number	6253.32 (5368.83-7297.01)	3209.73 (2742.54-3775.20)	3043.59 (3541.01-2614.16)	15496.57 (13681.82-17612.81)	7956.45 (7054.33-8977.18)	7540.12 (6598.95-8673.52)
	AA rate	1.81(1.56-2.11)	1.91 (1.63-2.24)	1.72 (2.00-1.48)	2.55 (2.25-2.89)	2.72 (2.41-3.07)	2.38 (2.09-2.74)
	ASR	3.08(2.62-3.66)	3.27 (2.78-3.88)	2.87 (3.42-2.45)	3.44 (3.07-3.95)	3.71 (3.31-4.21)	3.17 (2.79-3.68)
**Deaths**	AA number	1732.60 (1252.95-2117.91)	866.36 (518.62-1133.14)	866.24 (616.88-1100.38)	2744.22 (2070.62-3292.05)	1362.72 (937.36-)	1381.49 (1002.78-1718.84)
	AA rate	0.51 (0.36-0.61)	0.51 (0.31-0.67)	0.49 (0.35-0.62)	0.44 (0.32-0.54)	0.47 (0.32-0.60)	0.44 (0.32-0.54)
	ASR	0.84 (0.64-0.99)	0.84 (0.56-1.05)	0.83 (0.63-1.02)	0.65 (0.51-0.79)	0.69(0.48-0.91)	0.62 (0.45-0.76)
**DALYs**	AA number	82567.88 (45093.46-114668.50)	41988.76 (17449.89-64712.21)	40579.12 (24494.65-57921.82)	91251.66 (65940.57-111615.70)	42063.46 (27395.50-53482.61)	49188.20 (34270.88-62273.69)
	AA rate	23.93 (13.07-32.23)	24.95 (10.37-38.45)	22.96 (13.86-32.77)	14.99 (10.83-18.34)	14.38 (9.37-18.28)	15.56 (10.84-19.69)
	ASR	26.69 (18.81-32.98)	26.70 (15.39-35.43)	26.61 (18.91-34.11)	17.62 (12.91-21.40)	17.30 (11.55-21.82)	17.85 (12.54-22.32)
**YLLs**	AA number	82085.05 (44665.17-114250.30)	41736.53 (17206.24-64445.60)	40348.52 (24238.29-57563.25)	90070.07 (64887.92-110355.37)	41448.73 (26802.84-52855.76)	48621.34 (33577.19-61596.67)
	AA rate	23.79 (12.95-33.11)	24.80 (10.22-38.30)	22.83 (13.71-32.57)	14.80 (10.66-18.13)	14.17 (9.16-18.07)	15.38 (10.62-19.48)
	ASR	26.44 (18.52-32.73)	26.44 (15.15-35.20)	26.39 (18.66-33.88)	17.35 (12.70-21.13)	17.01 (11.27-21.56)	17.60 (12.22-22.06)
**YLDs**	AA number	482.83 (323.39-684.28)	252.23 (167.27-360.42)	230.60 (152.44-326.19)	1181.59 (791.35-1661.63)	614.73 (408.79-866.64)	566.86 (376.97-792.76)
	AA rate	0.14 (0.09-0.20)	0.15 (0.10-0.21)	0.13 (0.09-0.18)	0.19 (0.13-0.27)	0.21 (0.14-0.30)	0.18 (0.12-0.25)
	ASR	0.25 (0.17-0.35)	0.26 (0.18-0.38)	0.23 (0.15-0.32)	0.27 (0.18-0.37)	0.29 (0.19-0.41)	0.24 (0.17-0.34)

AA, all-ages; ASR, age-standardized rate; DALYs, disability-adjusted life years; YLLs, years of life lost; YLDs, years lived with disability; NAME, North Africa and the Middle East

**Table 2:** Percent Changes (%) of Different Metrics of Epidemiologic Measures of Infective Endocarditis in North Africa and the Middle East between 1990 and 2019

**Measures**	**Percent changes**	**Value (Uncertainty Intervals) **			**Interpretation**	**Differences in Direction of Percent Changes Across All-Age Numbers, Percentages, Rates, and Age-Standardized Rates and Percentages**
		**Point** **Estimate**	**Lower** **Bound**	**Upper** **Bound**		
Incidence	AAN	1.44	1.26	1.63	Increased	No
	AAP	0.41	0.30	0.53	Increased	
	AAR	0.38	0.28	0.49	Increased	
	ASP	0.29	0.26	0.32	Increased	
	ASR	0.34	0.31	0.37	Increased	
Prevalence	AAN	1.48	1.35	1.62	Increased	No
	AAP	39.98	32.82	48.15	Increased	
	AAR	0.40	0.33	0.49	Increased	
	ASP	13.01	5.88	21.02	Increased	
	ASR	0.12	0.05	0.20	Increased	
Deaths	AAN	0.58	0.30	0.92	Increased	Yes
	AAP	29.72	8.41	56.48	Increased	
	AAR	-0.10	-0.27	0.09	Decreased (not significant)	
	ASP	-	-	-	-	
	ASR	-0.22	-0.31	-0.09	Decrease	
DALYs	AAN	0.11	-0.21	0.63	Increased (not significant)	Yes
	AAP	12.25	-19.12	65.66	Increased (not significant)	
	AAR	-0.37	-0.55	-0.07	Decreased	
	ASP	-	-	-	-	
	ASR	-0.34	-0.46	-0.19	Decreased	
YLLs	AAN	0.10	-0.22	0.63	Increased (not significant)	Yes
	AAP	46.57	6.53	116.33	Increased	
	AAR	-0.38	-0.56	-0.08	Decreased	
	ASP	-	-	-	-	
	ASR	-0.34	-0.47	-0.20	Decreased	
YLDs	AAN	1.45	1.26	1.67	Increased	No
	AAP	20.53	11.30	31.49	Increased	
	AAR	0.39	0.28	0.51	Increased (not significant)	
	ASP	9.08	-1.11	21.39	Increased (not significant)	
	ASR	0.09	-0.02	0.20	Increased (not significant)	

AAN, All ages number; AAP, All ages percent; AAR, All ages rate; ASP, Age-standardized percent; ASR, Age-standardized rate.

**Table 3:** Decomposition Analysis of Incidence Rate Change of Infective Endocarditis between 1990 and 2019 in North Africa ‎and the Middle East and the 21 Countries of This Region

**Location**		**New Cases**		**Expected New Cases in 2019**		**% 1990–2019New** **Cases ChangeCause**			**% 1990-2019** **New Cases Overall Change (%)**
		**1990**	**2019**	**Population Growth**	**Population Growth+Aging**	**Population Growth (%)**	**Age Structure Change (%)**	**Incidence Rate Change** **(%)**	
North Africa and the Middle East		33371	81527	58875	61914	76.4	9.1	58.8	144.3
Country	Afghanistan	843	3589	2827	2616	235.2	-25	115.4	325.6
	Algeria	2195	5309	3633	4041	65.5	18.6	57.8	141.9
	Bahrain	46	204	131	158	184	58.9	98.1	341
	Egypt	5523	12702	9824	9533	77.9	-5.3	57.4	130
	Iran (Islamic Republic of)	6069	12992	8739	9400	44	10.9	59.2	114.1
	Iraq	1587	4807	3798	3657	139.4	-8.9	72.5	203
	Jordan	423	1653	1304	1496	208.4	45.6	37.1	291.1
	Kuwait	156	594	392	443	151.6	32.2	96.8	280.6
	Lebanon	409	963	646	719	58.1	17.8	59.7	135.6
	Libya	451	779	716	626	59	-20	33.9	72.9
	Morocco	1946	4365	2765	2977	42.1	10.9	71.3	124.3
	Oman	168	540	397	379	135.9	-10.7	95.9	221.1
	Palestine	168	537	402	391	139.4	-6.8	87.3	219.9
	Qatar	39	337	249	253	543.5	10.2	215.2	768.9
	Saudi Arabia	1379	4948	3070	3160	122.7	6.5	129.7	259
	Sudan	1521	3754	3074	2935	102	-9.1	53.8	146.7
	Syrian Arab Republic	1081	1817	1215	1300	12.4	7.8	47.9	68
	Tunisia	782	1823	1072	1265	37.1	24.6	71.4	133.1
	Turkey	7421	15709	10101	13093	36.1	40.3	35.3	111.7
	United Arab Emirates	168	1153	828	902	393.7	44.5	149.6	587.8
	Yemen	975	2868	2238	2151	129.5	-8.9	73.5	194.1

**Table 4:** All-Ages Number, All-Ages Rate, and Age-Standardized Rate of Incidence, Prevalence, Deaths, DALYs, YLLs, and YLDs of Infec-tive Endocarditis in North Africa and the Middle East in 1997 and 2007

**Measures**	**Metrics**	**Years**					
		**1997**			**2007**		
		**Both**	**Female**	**Male**	**Both**	**Female**	**Male**
Incidence	AA number	40964.50 (33778.60-48875.96)	18169.54 (14928.20-21753.72)	22794.96 (18787.21-27137.33)	55286.63 (45852.14-65587.90)	24584.57 (20470.59-29089.06)	30702.06 (25263.58-36506.39)
	AA rate	10.08 (8.31-12.03)	9.17 (7.54-10.98)	10.94 (9.02-13.03)	(9.28-13.27)	10.24 (8.52-12.11)	(9.94-14.36)
	ASR	12.13 (10.29-14.16)	11.05 (9.37-12.95)	13.14 (11.17-15.35)	(11.42-15.82)	12.50 (10.65-14.64)	(12.11-16.88)
Prevalence	AA number	7845.96 (6759.41-9116.74)	4008.29 (3438.06-4694.61)	3837.67 (3326.33-4440.16)	10973.66 (9775.64-12390.29)	5748.54 (5140.28-6450.17)	5225.13 (4593.96-5962.77)
	AA rate	1.93 (1.66-2.24)	2.02 (1.74-2.37)Table	1.84 (1.60-2.13)	2.39 (1.98-2.51)	2.39 (2.14-2.69)	2.06 (1.81-2.35)
	ASR	3.19 (2.73-3.75)	3.40 (2.91-4.03)	2.97 (2.55-3.52)	3.48 (3.10-3.95)	3.84 (3.41-4.34)	3.10 (2.74-3.58)
Deaths	AA number	1890.20 (1417.33-2210.31)	930.05 (590.13-1154.34)	960.15 (717.50-1205.25)	2157.00 (2549.46-1572.12)	1072.24(701.12-1307.65)	1084.76 (768.08-1348.52)
	AA rate	0.47 (0.35-0.54)	0.47 (0.30-0.58)	0.46 (0.34-0.58)	0.44 (0.32-0.52)	0.45 (0.29-0.54)	0.43 (0.30-0.53)
	ASR	0.79 (0.61-0.95)	0.80 (0.54-1)	0.78 (0.59-0.98)	0.71 (0.53-0.85)	0.74 (0.49-0.96)	0.68 (0.48-0.84)
DALYs	AA number	81123.30 (51566.62-101757.35)	40050.05 (19888.02-54843.98)	41073.25 (27617.38-52421.87)	83261.97 (55725.70-101329.02)	39727.03 (22783.24-51637.30)	43534.94 (29091.38-56024.08)
	AA rate	19.96 (12.69-25.04)	20.22 (10.04-27.59)	19.72 (13.26-25.17)	16.84 (11.27-20.50)	16.54 (9.49-21.50)	17.13 (11.44-22.04)
	ASR	24.09 (17.63-28.37)	23.93 (14.12-30.41)	24.19 (17.73-30.12)	20.52 (14.50-24.43)	20.37 (13.05-25.14)	20.58 (14.32-25.73)
YLLs	AA number	80517.58 (50911.07-101197.92)	39735.63 (19512.71-54526.08)	40781.95 (27339.24-52212.97)	82419.35 (54993.79-100408.99)	39279.43 (22304.73-51190.71)	43139.92 (28781.29-55614.12)
	AA rate	19.82 (12.53-24.91)	20.06 (9.85-27.53)	19.58 (13.13-25.07)	16.67 (11.12-20.31)	16.36 (9.29-21.32)	16.97 (11.32-21.88)
	ASR	23.83 (17.36-28.10)	23.66 (13.81-30.15)	23.96 (17.54-29.84)	20.24 (14.25-24.12)	20.06 (12.77-24.88)	20.34 (14.03-25.49)
YLDs	AA number	605.72 (403.00-852.49)	314.42 (208.84-450.18)	291.30 (191.72-410.20)	842.62 (565.04-1170.45)	447.60 (300.21-629.34)	395.02 (263.77-552.44)
	AA rate	0.15 (0.10-0.21)	0.16 (0.11-0.23)	0.14 (0.09-0.20)	0.17 (0.11-0.24)	0.19 (0.13-0.26)	0.16 (0.10-0.22)
	ASR	0.25 (0.17-0.36)	0.27 (0.18-0.40)	0.23 (0.16-0.33)	0.27 (0.19-0.38)	0.30 (0.20-0.43)	0.24 (0.16-0.34)

AA: all-ages; ASR: age-standardized rate; DALYs, disability-adjusted life years; YLLs, years ‎of life lost; YLDs, years lived with disability; NAME, North Africa and the Middle East

 In 2019, the incidence of IE was highest among those aged 70 and above (54.8 per 100 000, 95% UI: 43.3‒97.55) and was five times higher than those under 20 (10.9 per 100 000, 95% UI: 7.6‒16). In 2019, there were 10,694.6 (8445.4–13,175.4) new 70 + -year-old cases for both sexes, up 46% (38–53%) from 1990.

 At the country level, every nation’s age-standardized incidence rate (ASIR) of IE increased significantly from 1990 to 2019. Saudi Arabia saw the most significant increase (59 %, 51‒68 %), with its ASIR rising from 10.8 per 100 000 (8.9‒13) in 1990 to 17.2 per 100 000 (14.4‒20.4) in 2019. Jordan had the lowest increase in ASIR (10%, 4–16%). Turkey had the highest ASIR (19 per 100 000, 95% UI:16.1‒22.2) in 2019 (Tables S1 and S2).

###  Prevalence

 In 2019, there were 15.1 (13.7–17.6) thousand prevalent cases of IE in the NAME region, with comparable numbers in both genders, which reflected an increase of 12 % (5–20%) from 1990 to reach an age-standardized prevalence rate (ASPR) of 3.4 per 100 000 (95% UI: 3.1‒4) in 2019. The IE prevalent rates rose sharply with age, reaching 4.4 (3.7–5.5) thousand per 100 000 septuagenarians in 2019. In this age group, the incidence rate of IE has gone up by 24.3% (UI: 11.2%‒40.8%) between 1990 and 2019.

 Except for Turkey (-2%, UI: -1.5 to 1.3), ASPR rose from a minimum of 7% (UI: 1‒14%) in Jordan to a maximum of 45% (36–55%) in Oman. Turkey and Afghanistan experienced the highest and lowest ASPRs in 2019, respectively, at respective rates of 5.7 per 100 000 (95% UI:5.2–6.3.3) and 1.3 per 100 000 (95% UI:1.1-1.5) (Table S3 and Figure S6).

###  Deaths

 The age-standardized death rate (ASDR) in the NAME region decreased by 22% (9–31%) from 1990 to 2019, with a total of 2744 (UI: 2070–3292) deaths attributed to IE, with comparable rates in both sexes, and an ASDR of 0.7 per 100 000 (UI: 0.5–0.8) in 2019. Notably, the ASDR for children under five significantly decreased in 2019 compared to 1990, declining by 72% (19%‒85%) and reaching a death rate of 0.22 per 100 000 (0.08‒0.4 per 100 000) in 2019.

*Staphylococcus aureus* was the most common culprit pathogen for IE and other cardiac infections in the NAME region, with an ASDR of 0.18 per 100 000 (UI: 0.13‒0.25). IE and other cardiac infections caused by resistant strains had an all-age mortality rate that was more than twice as high as those caused by susceptible strains (0.56 per 100 000, UI: 0.39–0.79 versus 0.22, UI: 0.15–0.32)^29^ (Tables S4 and S5).

 Patients older than 70 had the highest death rates in 2019 (5.3 per 100 000, UI: 4–6.6) and the lowest decrease (percent change: -0.03, UI: -0.2 to + 0.2) compared with 1990. From 1990 to 2019, ASDR showed no significant change in the region except for Jordan, Bahrain, Turkey, Lebanon, and Algeria, which declined. Jordan had the largest 2019 ASDR decline (43%, 23–60%) (Figure S7). Although the point estimates for Morocco and Kuwait indicated an increase in ASDRs, the lower and upper bounds of the uncertainty intervals in both cases included zero (Morocco: 2, 95% UI: -13 to 95%, and Kuwait: 14%, 95% UI: -44 to 81%). The highest and lowest ASDRs in 2019 were recorded by Turkey (0.8, 95% UI: 0.6‒1.1) and Palestine (0.3, 95% UI: 0.2‒0.4), respectively.

 There was no consistent link between the SDI category of a country and the ASDR.

###  Disability-Adjusted Life Years 

 In 2019, there was a total number of 91.3 thousand disability-adjusted life-years and ASR of 17.6 per 100 000 (12.9–21.4) due to IE in the NAME region, which showed a 34% decrease (19–46%) compared to 1990. There was no gender difference between ASRs of DALYs in 2019. *Acinetobacter baumannii* was responsible for the highest portion of DALYs with an ASR of 3.91 (2.65‒5.72) per 100 000 (Tables S6 and S7).

 Next to neonates aged less than 28 days, patients aged 70 and above had the highest rate of DALYs (71.5 per 100 000, UI: 55.1‒88) in 2019, more than eight times higher than those aged less than 20 years, with no significant change since 1990 (percent change: -8.5%, UI: -27% to + 12.6%). Contrarily, patients under the age of 5 years showed the greatest reduction in DALYs since 1990 (71.5%, UI: 18.9‒85%).

 In almost 50% of the region’s countries, there was a decreasing trend in the ASR of DALYs compared with 1990. These countries, in order of decrease in DALYs, from highest to lowest, were Libya (-48%, 95% UI: -11 to -69%), Jordan (-46%, 95% UI: -25 to -60%), Turkey (-45%, 95% UI: -27 to -59%), Bahrain (-44%, -18 to -58%), Egypt (95% UI: -42%, -4 to -63%), Algeria (36%, 95% UI: -5 to -55%), Qatar (35%, 95% UI: -6 to -56% ), Lebanon (33%, -10 to -51%), Iran (33%, -17 to -47%), and Tunisia (-28%, 95% UI: -3 to -49%). The ASR of DALYs did not change significantly in other countries (Figure S8). There was no correlation between the DALYs and SDIs of countries.

###  Years of Life Lost 

 Following a 34% (20–47%) decrease in the ASR of YLLs from 1990 to 2019, the YLL ASR decreased to 17.4 per 100 000 (95% UI: 21.1–12.7) in 2019. However, in 2019, more than 90.1 (95% UI: 64.9–110.4) thousand years of life were lost due to IE in the NAME region, with no gender difference.

 As expected, neonates aged less than six and less than 28 days had the highest ASR of YLL (377.2 per 100 000, 95% UI: 76–975.6; and 251.9 per 100 000, 95% UI: 52.6–569.3, respectively), whereas 5‒9-year-old children had the lowest ASR of YLL (3.2 per 100 000, 95% UI: 1.8‒5.2), and children under five had the greatest decrease in YLL compared to 1990 (-72%, UI: -19%‒85%).

 Egypt had the highest YLL ASR in 2019 at 25.2 per 100 000 (95% UI: 13.8–35.8), while Palestine had the lowest at 7.6 per 100 000 (95% UI: 4.8‒9.8).

 In 11 out of the 21 countries, the YLL ASR decreased significantly, with the highest decline occurring in Libya, with a 49 percent decline (-70 to -11%), falling to a YLL ASR of 16.7 per 100 000 (95% UI: 9.9‒25) in 2019 (Figure S9).

 Tunisia experienced the lowest significant decrease in YLL, with a 29 percent reduction (3-50%) in ASIR to achieve a YLL ASR of 12.3 per 100 000 (95% UI: 8.7‒17) in 2019. In the Syrian Arab Republic, the United Arab Emirates, Iraq, Oman, Yemen, Saudi Arabia, Afghanistan, Morocco, and Kuwait, the observed percent change, as a decrease or increase, was not statistically significant (Table S8).

 There was no consistent relationship between the ASR of YLL and the SDI of countries.

###  Years Lived With Disability 

 In the NAME region, the YLD ASR increased by 9% (95% UI: -2% to 2%) between 1990 and 2019, reaching 0.27 per 100 000 (95% UI: 0.18‒0.37) in 2019 with no sex predominance and a total of 1181.6 YLD (95% UI: 793.4‒1661.6).

 With the exception of Turkey, Jordan, Bahrain, and Qatar, YLD ASR increased significantly across the board in the region in 2019, with percentage increases ranging from 7% (1-15%) in the United Arab Emirates to 42% (31‒55%) in Oman. Only in Turkey did the ASR of YLD experience a small decline (6%, -25% to 20%) (Table S9 and S10; Figure S10 - Figure S12).

 The ASR of YLD and the SDI of nations did not correlate with one another (Figure S13).

###  Impact of Three Iterations of AHA Guidelines in 1990, 1997, and 2007 on the Burden of IE in the NAME Region

 From 1990 to 2019, there was a steady upward trend in incidence, prevalence, and YLDs and a downward trend in ASRs of deaths, DALYs, and YLLs. However, there was no temporal correlation between these trends and the dates that the AHA released new guidelines in 1990, 1997, and 2007 (Tables S10 and S11, Figure S14 and Figure S15).

 Table S12 shows NAME countries with the highest and lowest IE epidemiologic measures and burden in 2019.

 Figures S16–S19 illustrate the trend according to SDI and age groups.

## Discussion

 This study showed that, at the regional level, between 1990 and 2019, ASRs of incidence, prevalence, and YLDs increased by 59%,12%, and 9%, respectively, whereas ASRs of deaths, DALYs, and YLLs declined by 22%, 34%, and 34%, respectively (Figure S20). Furthermore, incidence rates of IE increased by 56% (46‒64) in the 70 + -year-old population in the NAME region. Less than 5-year-old children witnessed a conspicuous drop in rates of deaths, YLLs, and DALYs in respective order of 72% (-19 to -85%), 72% (-19 to -85%), and 71% (-19 to -85%). At the country level, in 2019, Turkey experienced the highest ASR of IE incidence, prevalence, death, and YLD, whereas Saudi Arabia showed the highest percentage increase in ASIR of IE during these three decades. However, the percent change for ASR of prevalence and YLD was at the lowest level for this country. In 2019, Afghanistan had the lowest ASR of incidence, prevalence, DALYs, and YLDs. Palestine had the lowest ASRs of death, DALY, and YLL. Oman experienced the highest percent change in ASPR, and Egypt witnessed the highest ASR of DALY and YLL (Table S10).

 A study on the epidemiology and the burden of IE in the countries of NAME spanning the three decades of 1990 to 2019 has been conducted only in Iran.^30^ Ajam et al conducted a comprehensive investigation on the epidemiology and impact of IE in Iran, spanning the period from 1990 to 2019. Their findings revealed a notable rise in the ASIR and ASPR of IE, while the age-standardized mortality rate (ASMR) and age-standardized DALYs rate exhibited a decline over the course of three decades in Iran. The majority of provinces exhibited a consistent trend, wherein North Khorasan displayed the highest rates of ASIR, ASPR, ASMR, and DALYs in both years.

 In a study involving 132 cases of definite IE conducted in Egypt, El-Kholy et al discovered that 69.7% of patients exhibited blood culture-negative IE (BCNIE). Upon implementing serology and polymerase chain reaction (PCR) on both blood and valvular tissue, this percentage decreased to 49.2%.^31^ Considering that cases are enrolled based on clinical diagnosis, it can be inferred that the reported incidence by IHME might underestimate the actual frequency of IE (Supplementary file 1). To prevent the potential underdiagnosis of IE and to ensure effective treatment, the development and implementation of a consistent, stepwise guideline for approaching BCNIE is of utmost importance.

 Similarly, Zaqout et al studied 57 patients with IE between 2015 and 207 in Qatar, 17 of whom had prosthetic cardiac valves or implantable cardiac devices. Staphylococci were the most common etiologic agent.^32^

 The development of more sensitive diagnostic criteria can partially explain the higher rates of IE incidence in 2019.^33-35^ Emerging risk factors, changing demographics of the at-risk population to an elderly population with coexisting comorbidities or healthcare-associated infections, and evolving causative microorganisms and their invasiveness and resistance can result in further increase IE incidence and prevalence.

 Regarding the risk factors for IE (Table S13), the increase in ASIR of IE is primarily associated with the increase in CKD and DM, rather than with rheumatic heart disease (RHD), non-rheumatic valvular disease, or CHD. Between 1990 and 2019, ASIRs of RHD, non-rheumatic valvular heart disease, drug use disorders, CKD, and DM increased by 8% (3–12%), 8% (2-13%), 13% (11–15%), 71% (67–75%), and 80% (75–85%), while CHD ASIR decreased by 7% (-3% to -11%).

 The significant increase in ASRs of DM and CKD calls for immediate action by health policymakers to implement strategies for reducing the modifiable risk factors of DM and CKD. A study by Li-Jin et al identifies hypertension as the top risk factor for IE mortality from 1990 to 2030.^36^

 The ASIR of IE in Saudi Arabia increased from 10.8 per 100 000 in 1990 to 17.7 per 100 000 in 2019, but this increase is not solely due to the SDI level, as Jordan experienced the smallest increase between 1990 and 2019. It seems that advancements in invasive medical procedures, particularly transcatheter cardiovascular interventions, play a greater role.^37^

 The epidemiologic transition of IE involves a shift towards older, complex, and comorbid populations, which are more likely to acquire invasive microorganisms due to their higher risk of healthcare-associated infections. The age range shift is partly a reflection of the global improvement in life expectancy and the aging of the world population.^38^

 IE in the elderly and in patients with healthcare-associated IE is often caused by more virulent microorganisms with higher rates of antibacterial tolerance, persistence, and resistance.^39-41^ Consequently, these factors can result in lower rates of cure, as well as increased rates of prevalence and YLDs of IE.

 The significant decrease in under-5 mortality may indicate success in achieving Sustainable Development Goal 3.2.1, approved by the United Nations in 2015.^42^

 To prevent a rise in the number of new cases and stop the increasing prevalence of IE, it is necessary to implement primary, secondary, and tertiary prevention measures at both the national and regional levels.

 Primary prevention interventions may include public education regarding lifestyle optimization to prevent obesity and hypertension (as modifiable risk factors for DM and chronic kidney disease) and to adhere to doctors’ prescriptions for IE antibiotic prophylaxis. Physicians must be informed about evolving IE risk factors, emerging microorganisms, at-risk populations, and AP guidelines, with specific guidelines aimed at preventing healthcare-associated infective endocarditis. (HAIE).

 Secondary prevention strategies involve physician education for IE management, improved diagnostic criteria, and a multidisciplinary team to shorten the time between diagnosis and cardiac surgery for patients.^43-45^ Moreover, policies should be implemented to prevent and control antimicrobial resistance.^46^

 Certain infrastructure initiatives, such as the establishment of healthcare-related IE registries and the Artificial Intelligence-Enhanced Electronic IE Registry Network (AIERN) of the NAME region, can be crucial for registering IE-related strategic health data.^47,48^

## Conclusion

 The study revealed a significant shift in IE epidemiology in the NAME region, notably an increase in elderly cases. From 1990 to 2019, there was a rise in IE incidence, prevalence, and YLDs. In 2019, IE rates increased significantly in septuagenarians, while deaths and DALYs remained stable. Turkeyexperienced the highest ASR of incidence, prevalence, and deaths. No correlation was found between IE rates and gender, SDI, or the AHA guideline updates. ASR for deaths, DALYs, and YLLs decreased, especially in children under five in Jordan. To mitigate IE, comprehensive prevention, a regional registry, a multidisciplinary team approach, antimicrobial resistance protocols, and public education on lifestyle factors are crucial. Additionally, integrating AP for IE into the core curricula of medical and dental education and continuing professional development are highly recommended.

## Supplementary Files

Supplementary file 1. Methods appendix to “Infective Endocarditis in North Africa and the Middle East, 1990-2019: Updates from the Global Burden of Disease Study 2019”.

Supplementary file 2 contains Figures S1 to S20.

Supplementary file 3 contains Tables S1 to S13.

Supplementary file 4. List of the GBD 2019 NAME Endocarditis Collaborators and their affiliations and contribution.
