# Supplementary material for: Infective Endocarditis in North Africa and the Middle East, 1990‒2019: Updates from the Global Burden of Disease Study 2019
Source: Arch Iran Med. 2024 May 1;27(5):229–38. doi: 10.34172/aim.2024.34 (PMC11097324; doi:10.34172/aim.2024.34)
Supplement: Supplementary file 1 — Methods appendix to “Infective Endocarditis in North Africa and the Middle East, 1990-2019: Updates from the Global Burden of Disease Study 2019”. [file aim-27-229-s001.pdf]

S1 Appendix

Methods appendix to

Infective Endocarditis in North Africa and the Middle East, 1990-2019: Updates from the Global Burden of Disease Study 2019

Portions of this appendix have been reproduced or adopted from:
Reference 19:
Diseases GBD, Injuries C. Global burden of 369 diseases and injuries in 204 countries and territories, 1990-2019: a systematic analysis for the Global Burden of Disease Study 2019. Lancet. 2020;396(10258):1204-22. doi: 10.1016/S0140-6736(20)30925-9. PubMed PMID: 33069326; PubMed Central PMCID: PMCPMC7567026.

Cause of Death (CoD) modeling description

Endocarditis

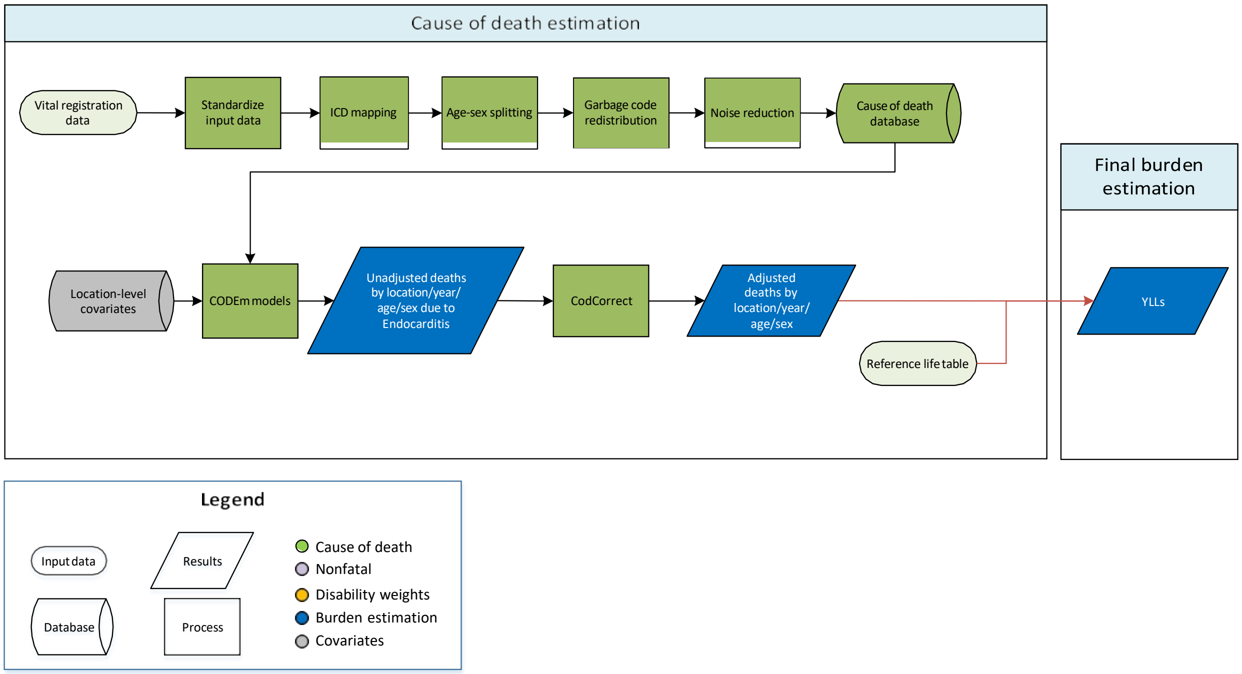

Input data
Vital registration data were used to model endocarditis. We outliered data in Mozambique as these were non-representative for sub-Saharan Africa and were causing regional estimates to be implausiblylow. We also outliered ICD8 data that were discontinuous from the rest of the data series and created an implausible time trend.

Modeling strategy
We used a standard CODEm approach to model deaths from endocarditis. Covariates selected for inclusion in the CODEm ensemble modelling process are listed in the table below. For GBD 2019, the same covariates as GBD 2017 were used. We changed the level of the healthcare access and quality index covariate from 1 to 2 for consistency with our *a priori* hypothesis about the relative impact of the covariate on mortality from endocarditis. We also changed the direction of the socio-demographic indexcovariate from 0 to -1. Apart from these updates to the covariates, there have been no substantive changes from the approach used in GBD 2016.

Table: Selected covariates for CODEm models, endocarditis

Covariate	Transformation	Level	Direction
Summary exposure value, endocarditis	None	1	1
Improved water (proportion)	None	1	-1
Sanitation (proportion with access)	None	1	-1
Healthcare access and quality index	None	2	-1
Lag distributed income per capita (I$)	Log	3	-1
Socio-demographic Index	None	3	-1

Non-fatal modeling description

Acute Endocarditis

Flowchart

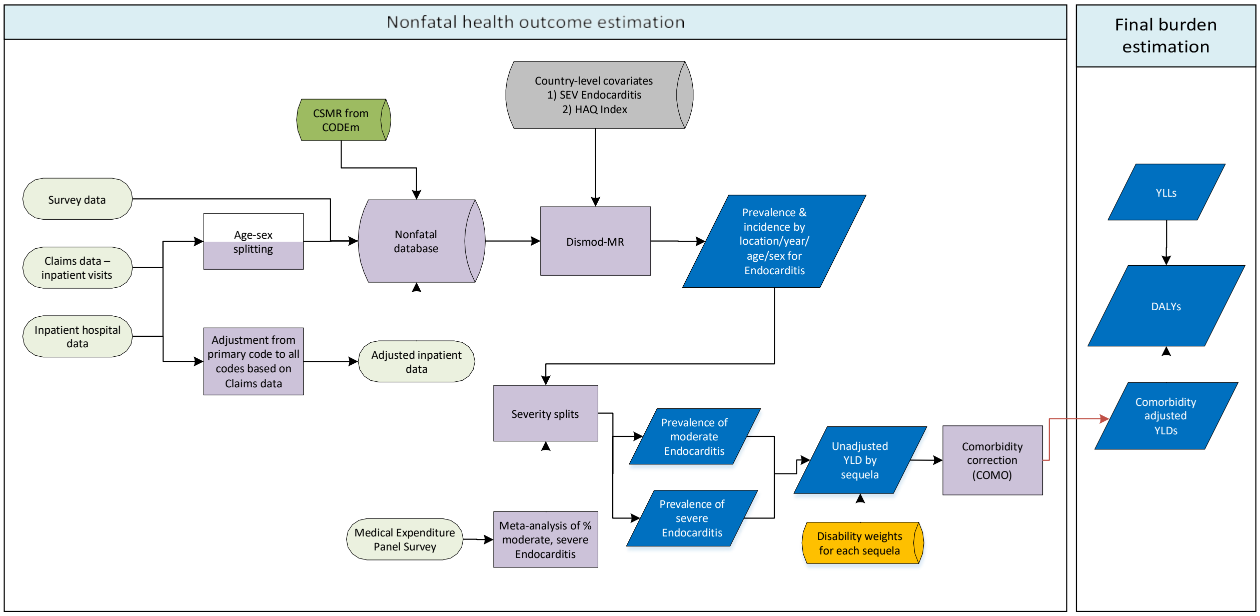

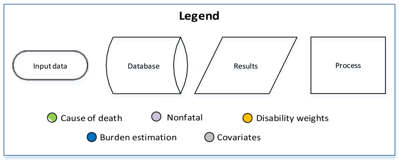

Input data and methodological appendix

Case definition

Our case definition for acute endocarditis was a clinical diagnosis of infective endocarditis. The ICD codesincluded can be found elsewhere in the appendix.

Input data

Model inputs

Table 1: Source counts for acute endocarditis

Measure	Total sources	Countries with data
All measures	303	41
Incidence	303	41

Table 1 displays the source counts for the non-fatal acute endocarditis model. We did not perform a systematic review for GBD 2019. A systematic review was performed for GBD 2013 and updated for GBD2015. . The following search terms were used: ((‘endocarditis’[MeSH Terms] OR ‘endocarditis’[All Fields]) AND ‘epidemiology’[Subheading]) OR ((‘endocarditis’[MeSH Terms] OR ‘endocarditis’[All Fields]) AND

((‘epidemiology’[Subheading] OR ‘epidemiology’[All Fields] OR ‘incidence’[All Fields] OR ‘incidence’[MeSHTerms]) OR (‘epidemiology’[Subheading] OR ‘epidemiology’[All Fields] OR ‘prevalence’[All Fields] OR ‘prevalence’[MeSH Terms]) OR ‘case fatality’[All Fields])) OR ((‘endocardium’[MeSH Terms] OR ‘endocardium’[All Fields]) AND inflammation[TIAB] AND ‘epidemiology’[Subheading]) OR ((‘endocardium’[MeSH Terms] OR ‘endocardium’[All Fields]) AND inflammation[TIAB] AND ((‘epidemiology’[Subheading] OR ‘epidemiology’[All Fields] OR ‘incidence’[All Fields] OR ‘incidence’[MeSHTerms]) OR (‘epidemiology’[Subheading] OR ‘epidemiology’[All Fields] OR ‘prevalence’[All Fields] OR ‘prevalence’[MeSH Terms]) OR ‘case fatality’[All Fields]))

- Dates included in search: 1/1/2013 – 3/16/2015
- Number of initial hits: 1,246
- Number of sources included: 6

We did not include any non-literature-based data types apart from the hospital and claims data described elsewhere. We excluded all outpatient data, as they were implausibly low when compared withinpatient data from the same locations and claims data. We used hospital data corrected for readmission and primary to any diagnosis based on the correction factors generated by the clinical informatics team. We excluded any inpatient hospital data points that were more than two-fold higher or 0.5-fold lower than the median absolute deviation value for high-income North America, Central Europe, and Western Europe for that age-sex group. No data adjustments were made for acute endocarditis in GBD 2019.

Severity split inputs

We used the standard GBD approach, which utilizes MEPS data to split overall estimates of endocarditis into moderate and severe categories. The table below includes the severity level, lay descriptions, and DWs associated with acute endocarditis.

Table 2. Severity distribution, details on the severity levels for Acute Endocarditis in GBD 2019, and theassociated disability weight (DW) with that severity.

Severity level	Lay description	DW (95% CI)
Moderate	Has a fever and aches and feels weak, which causes somedifficulty with daily activities.	0.051 (0.032–0.074)
Severe	Has a high fever and pain and feels very weak, which causes great difficulty with daily activities.	0.133 (0.088–0.19)

Modeling strategy

For GBD 2019, we estimated endocarditis using a DisMod-MR Bayesian meta-regression model, setting aminimum of 11 and maximum of 13 as value priors on remission to establish an average duration of one month. For GBD 2019, we outliered cause-specific mortality rate data from Mali due to implausibly high estimates. Country-level covariates used included the endocarditis summary exposure variable (SEV) on incidence and the Health Access and Quality Index on excess mortality.
We evaluated models by comparing model fits with the data and with results from previous GBDestimation cycles.

The table below gives the parameters, betas, and exponentiated betas for study-level and country-level covariates used in the model.

Table 3. Covariates. Summary of covariates used in the Acute Endocarditis DisMod-MR meta-regression model

Covariate	Parameter	Beta	Exponentiated beta (95% Uncertainty Interval)
Health Access and Quality Index	Excess mortality rate	-0.1 (-0.1 to -0.1)	0.90 (0.90 to 0.90)
Log-transformed age-standardized SEV scalar: endocarditis	Incidence	0.78 (0.75 to 0.83)	2.19 (2.12 to 2.30)

No significant changes were made to the modeling strategy from GBD 2017.
