# Supplementary material for: Infective Endocarditis in North Africa and the Middle East, 1990‒2019: Updates from the Global Burden of Disease Study 2019
Source: Arch Iran Med. 2024 May 1;27(5):229–38. doi: 10.34172/aim.2024.34 (PMC11097324; doi:10.34172/aim.2024.34)
Supplement: Supplementary file 2 — contains Figures S1 to S20. [file aim-27-229-s002.pdf]

# S2 Appendix

| Table of contents of S3 Appendix | | |
|---|---|---|
| **Figure's Number** | **Title** | **Page** |
| **S1 Fig.** | **The flow-chart for estimation of non-fatal health outcome of infective endocarditis by the Institute for Health Metrics and** Evaluatio**n (IHME)** *Reference: Diseases GBD, Injuries C. Global burden of 369 diseases and injuries in 204 countries and territories, 1990-2019: a systematic analysis for the Global Burden of Disease Study 2019. Lancet. 2020;396(10258):1204-22. doi: 10.1016/S0140-6736(20)30925-9. PubMed PMID: 33069326; PubMed Central PMCID: PMCPMC7567026.* | **1** |
| **S2 Fig.** | **The flow-chart for estimation of fatal outcome of infective endocarditis The flow-chart for estimation of fatal outcome of infective endocarditis by The Institute for Health Metrics and Evaluation (IHME)** *Reference: Diseases GBD, Injuries C. Global burden of 369 diseases and injuries in 204 countries and territories, 1990-2019: a systematic analysis for the Global Burden of Disease Study 2019. Lancet. 2020;396(10258):1204-22. doi: 10.1016/S0140-6736(20)30925-9. PubMed PMID: 33069326; PubMed Central PMCID: PMCPMC7567026.* | **3** |
| **S3 Fig.** | **The temporal trend of age-standardized incidence, prevalence, deaths, and DALYs of infective endocarditis in North Africa and the Middle East between 1990 and 2019 in the three eras (1990–1997, 1998–2007, and 2008–2019)** | **5** |

| Table of contents of S3 Appendix | | |
|---|---|---|
| **Figure's Number** | **Title** | **Page** |
| **S4 Fig.** | **The temporal trend of age-standardized incidence, prevalence, deaths, and DALYs of infective endocarditis in North Africa and the Middle East between 1990 and 2019 in the three eras (1990–1997, 1998–2007, and 2008–2019) according to sex** | **7** |
| **S5 Fig.** | **Upper panel: Age-standardized incidence rate (per 100,000) of infective endocarditis for the nations of North Africa and the Middle East in 1990, 1997, 2007, and 2019; Lower panel: percentage changes (%) in the age-standardized incidence rate for the countries of North Africa and the Middle East from 1990 to 1997, 1998 to 2007, and 2008 to 2019** | **9** |
| **S6 Fig.** | **Upper panel: Age-standardized prevalence rate (per 100,000) of infective endocarditis for the nations of North Africa and the Middle East in 1990, 1997, 2007, and 2019; Lower panel: percentage changes (%) in age-standardized prevalence rate for the countries of North Africa and the Middle East from 1990 to 1997, 1998 to 2007, and 2008 to 2019** | **11** |
| **S7 Fig.** | **Upper panel: Age-standardized death rate (per 100,000) of infective endocarditis for the nations of North Africa and the Middle East in 1990, 1997, 2007, and 2019; Lower panel: percentage changes (%) in age-standardized death rate for the countries of North Africa and the Middle East from 1990 to 1997, 1998 to 2007, and 2008 to 2019** | **13** |

| Table of contents of S3 Appendix | | |
|---|---|---|
| **Figure's Number** | **Title** | **Page** |
| **S8 Fig.** | **Upper panel: Age-standardized rate of disability-adjusted life years (DALY) (per 100,000) of infective endocarditis for the nations of North Africa and the Middle East in 1990, 1997, 2007, and 2019; Lower panel: percentage changes (%) in age-standardized death rate for the countries of North Africa and the Middle East from 1990to 1997, 1998 to 2007, and 2008 to 2019** | |
| **S9 Fig.** | **Upper panel: Age-standardized rate (per 100, 000) of YLLs of infective endocarditis for the nations of North Africa and the Middle East in 1990, 1997, 2007, and 2019; Lower panel: Percentage changes (%) in age-standardized rate (per 100, 000) of YLLs in North Africa and the Middle East from 1990 to 1997, 1998 to 2007, and 2008 to 2019** | **17** |
| **S10 Fig.** | **Upper panel: Age-standardized rate (per 100, 000) of YLDs of infective endocarditis for the nations of North Africa and the Middle East in 1990, 1997, 2007, and 2019; Lower panel: Percentage changes (%) in age-standardized rate (per 100, 000) of YLDs in North Africa and the Middle East from 1990 to 1997, 1998 to 2007, and 2008 to 2019** | **19** |
| **S11 Fig.** | **Comparison of rates (per 100,000) of incidence, prevalence, deaths, and DALYs of infective endocarditis in North Africa and the Middle East in 1990 and 2019 according to eighteen agegroups and sex** | **21** |

| Table of contents of S3 Appendix | | |
|---|---|---|
| **Figure's Number** | **Title** | **Page** |
| **S12 Fig.** | **Rankings of 21 countries of North African and Middle Eastern countries based on age-standardized rates of incidence, prevalence, deaths, and DALYs of infective endocarditis in 1990 and 2019** | **23** |
| **S13 Fig.** | **Age-standardized rates (per 100,000) of YLDs and YLLs of infective endocarditis in 21 North African and Middle Eastern countries in 2019 by quintiles of the countries' sociodemographicindex (SDI)** | **25** |
| **S14 Fig.** | **The temporal trend of age-standardized incidence, prevalence, deaths, and DALYs of infective endocarditis in 21 countries of North Africa and the Middle East between 1990 and 2019 in the three eras (1990–1997, 1998–2007, and 2008–2019)** | **27** |
| **S15 Fig.** | **Percent changes (%) in age-standardized rates (per 100,000) of incidence, prevalence, deaths, and DALYs of infective endocarditis between 1997 and 2007, and 2007 and 2019 in 21 countries of North Africa and the Middle East** | **29** |
| **S16 Fig.** | **Age-standardized rates (per 100,000) of incidence, prevalence, deaths, and disability-adjusted life years (DALYs) of infective endocarditis in 21 North African and Middle Eastern countries in 1990, 1997, 2007, and 2019 by quintiles of the countries' sociodemographic index (SDI)** | **31** |

| Table of contents of S3 Appendix | | |
|---|---|---|
| **Figure's Number** | **Title** | **Page** |
| **S17 Fig.** | **Age-standardized rates (per 100,000) of incidence, prevalence, deaths, and disability-adjusted life years (DALYs) of infective endocarditis in 21 North African and Middle Eastern nations from 1990 to 2019 at three-year intervals (1990, 1993, 1996, 1999, 2002, 2005, 2008, 2011, 2014, 2017 and 2019) by quintiles of the countries' sociodemographic index (SDI)** | **33** |
| **S18 Fig.** | **Rates (per 100,000) of incidence, prevalence, deaths, and DALYs of infective endocarditis in 21 countries of North Africa and the Middle East in 1990 compared with 1997, in 1998 versus 2007,and in 2008 versus 2019 according to eighteen age groups and sex** | **35** |
| **S19 Fig.** | **Rates (per 100,000) of incidence, prevalence, deaths, and DALYs of infective endocarditis in 21 countries of North Africa and the Middle East in 1990 compared with 2019 according to eighteenage groups and sex** | **57** |
| **S20 Fig.** | **Temporal trend of age-standardized incidence rate per 100,000 in North Africa and the Middle East region and the 21 countries of this region** | **79** |

**S1 Fig.**

The flow-chart for estimation of non-fatal health outcome of infective endocarditis by The Institute for Health Metrics and Evaluation (IHME)

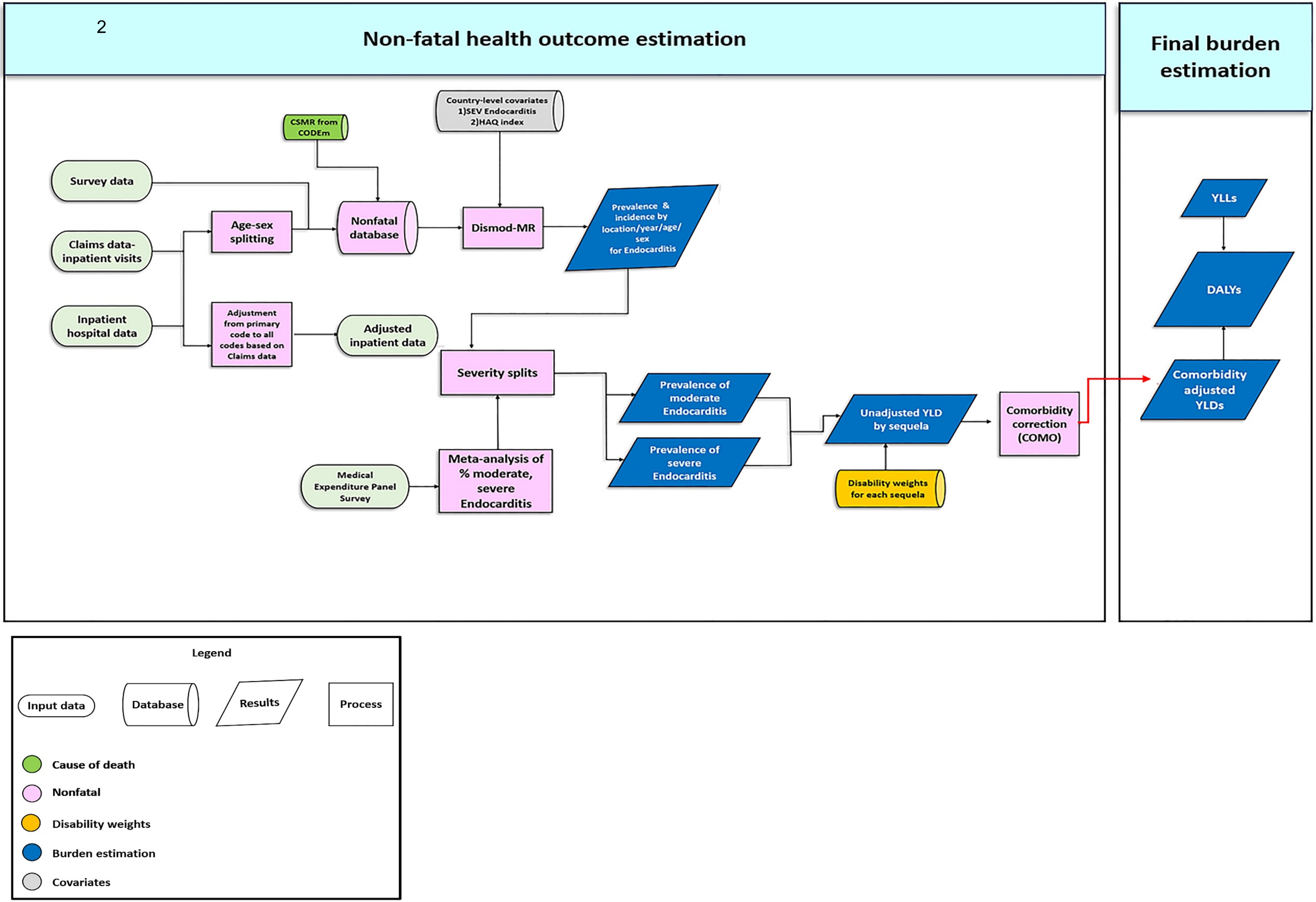
Non-fatal health outcome estimation
Final burden estimation
CSMR from CODEm
Country-level covariates 1)SEV Endocarditis 2)HAQ index
Survey data
Claims data-inpatient visits
Inpatient hospital data
Age-sex splitting
Nonfatal database
Dismod-MR
Prevalence & incidence by location/year/age/sex for Endocarditis
Adjustment from primary code to all codes based on Claims data
Adjusted inpatient data
Severity splits
Prevalence of moderate Endocarditis
Prevalence of severe Endocarditis
Unadjusted YLD by sequela
Comorbidity correction (COMO)
Disability weights for each sequela
Medical Expenditure Panel Survey
Meta-analysis of % moderate, severe Endocarditis
YLLs
DALYs
Comorbidity adjusted YLDs
Legend
Input data
Database
Results
Process
Cause of death
Nonfatal
Disability weights
Burden estimation
Covariates

**S2 Fig.**

The flow-chart for estimation of fatal outcome of infective endocarditis by The Institute for Health Metrics and Evaluation (IHME)

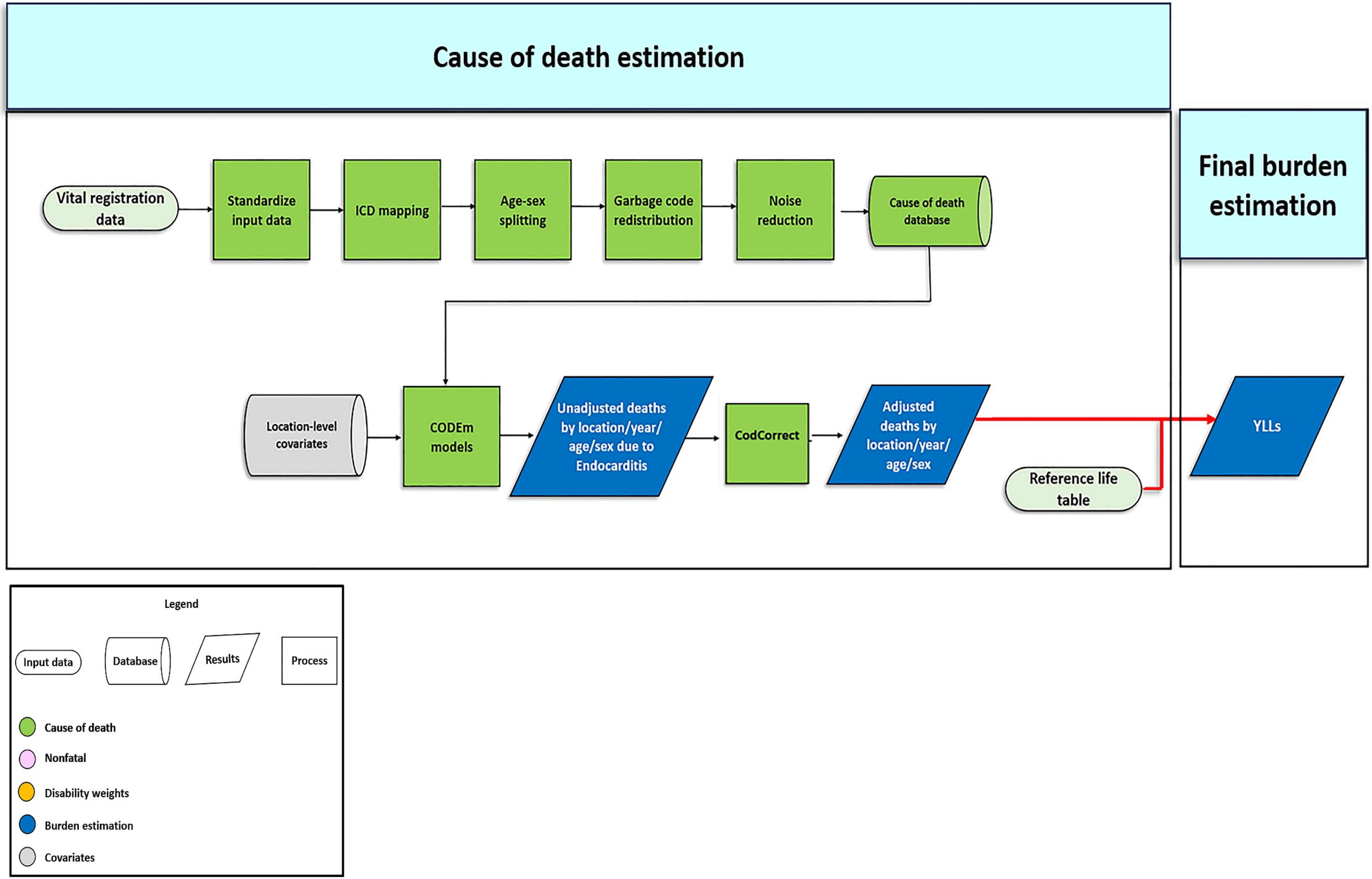
Cause of death estimation
Final burden estimation
Vital registration data
Standardize input data
ICD mapping
Age-sex splitting
Garbage code redistribution
Noise reduction
Cause of death database
Location-level covariates
CODEm models
Unadjusted deaths by location/year/ age/sex due to Endocarditis
CodCorrect
Adjusted deaths by location/year/ age/sex
Reference life table
YLLs
Legend
Input data
Database
Results
Process
Cause of death
Nonfatal
Disability weights
Burden estimation
Covariates

**S3 Fig.**

**The temporal trend of age-standardized incidence, prevalence, deaths, and DALYs of infective endocarditis in North Africa and the Middle East between 1990 and 2019 in the three eras (1990–1997, 1998–2007, and 2008–2019).**

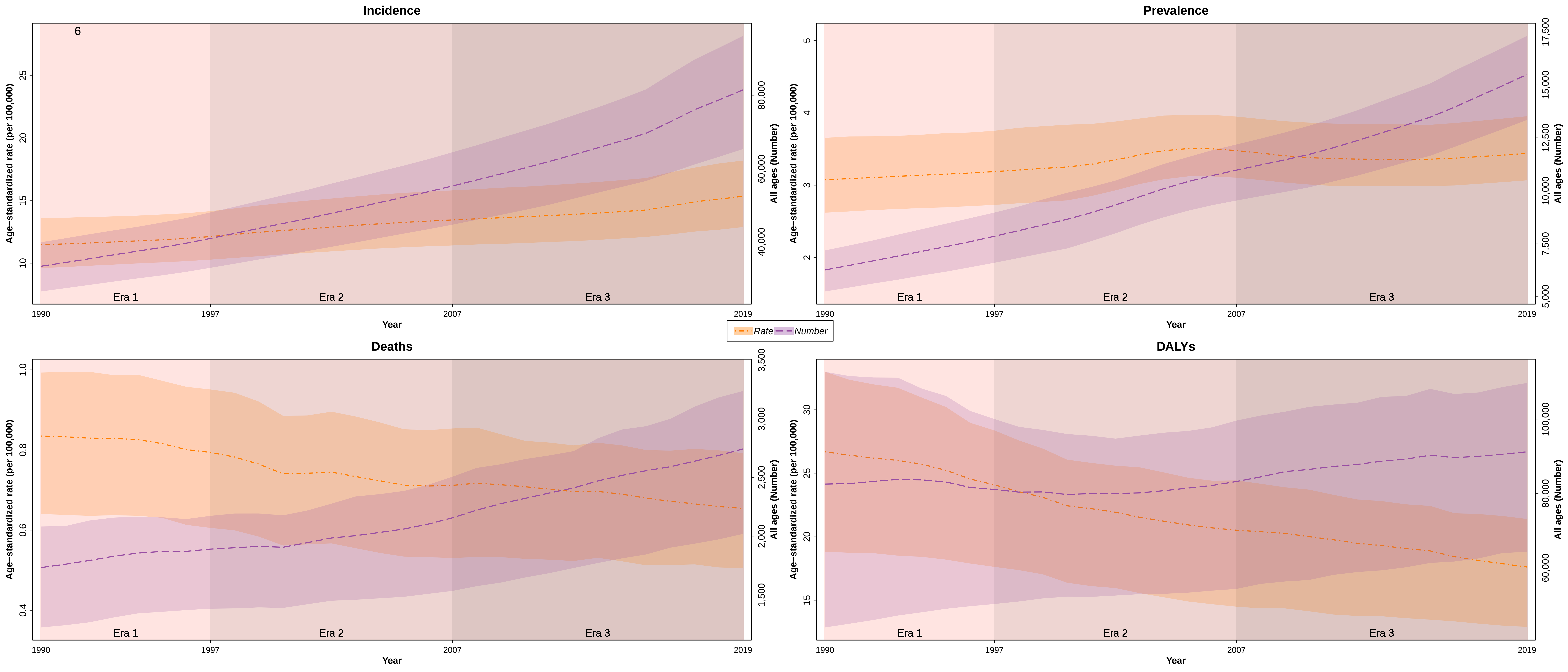

Incidence
6
Age−standardized rate (per 100,000)
All ages (Number)
Era 1
Era 2
Era 3
Year
Prevalence
Deaths
DALYs
Rate
Number

**S4 Fig.**

**The temporal trend of age-standardized incidence, prevalence, deaths, and DALYs of infective endocarditis in North Africa and the Middle East between 1990 and 2019 in the three eras (1990–1997, 1998–2007, and 2008–2019) according to sex**

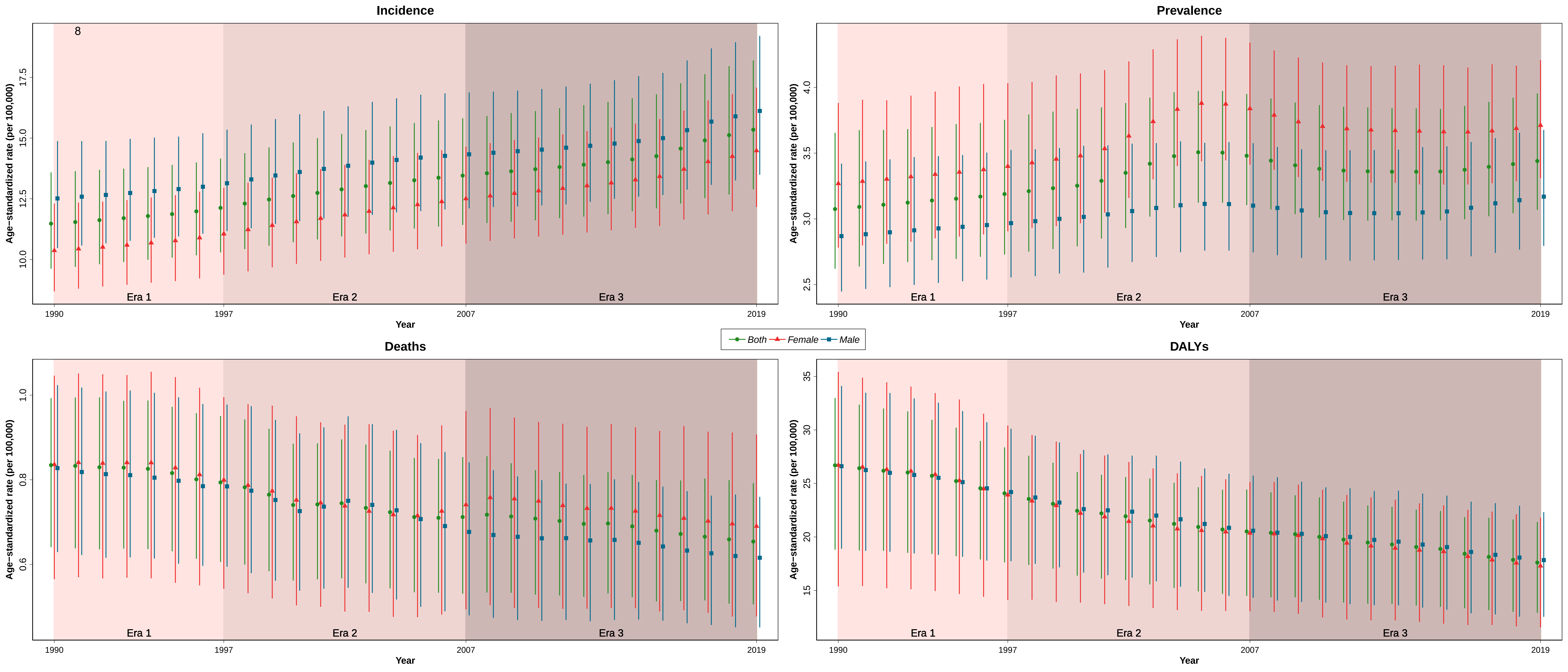

Incidence
8
Era 1
Era 2
Era 3
Age−standardized rate (per 100,000)
Year
Prevalence
Deaths
DALYs
Both
Female
Male

**S5 Fig.**

**Upper panel: Age-standardized incidence rate (per 100,000) of infective endocarditis for the nations of North Africa and the Middle East in 1990, 1997, 2007, and 2019; Lower panel: percentage changes (%) in the age-standardized incidence rate for the countries of North Africa and the Middle East from 1990 to 1997, 1998 to 2007, and 2008 to 2019**

***Contains information from OpenStreetMap and OpenStreetMap Foundation, which is made available under the Open Database License.***

10

**Incidence**

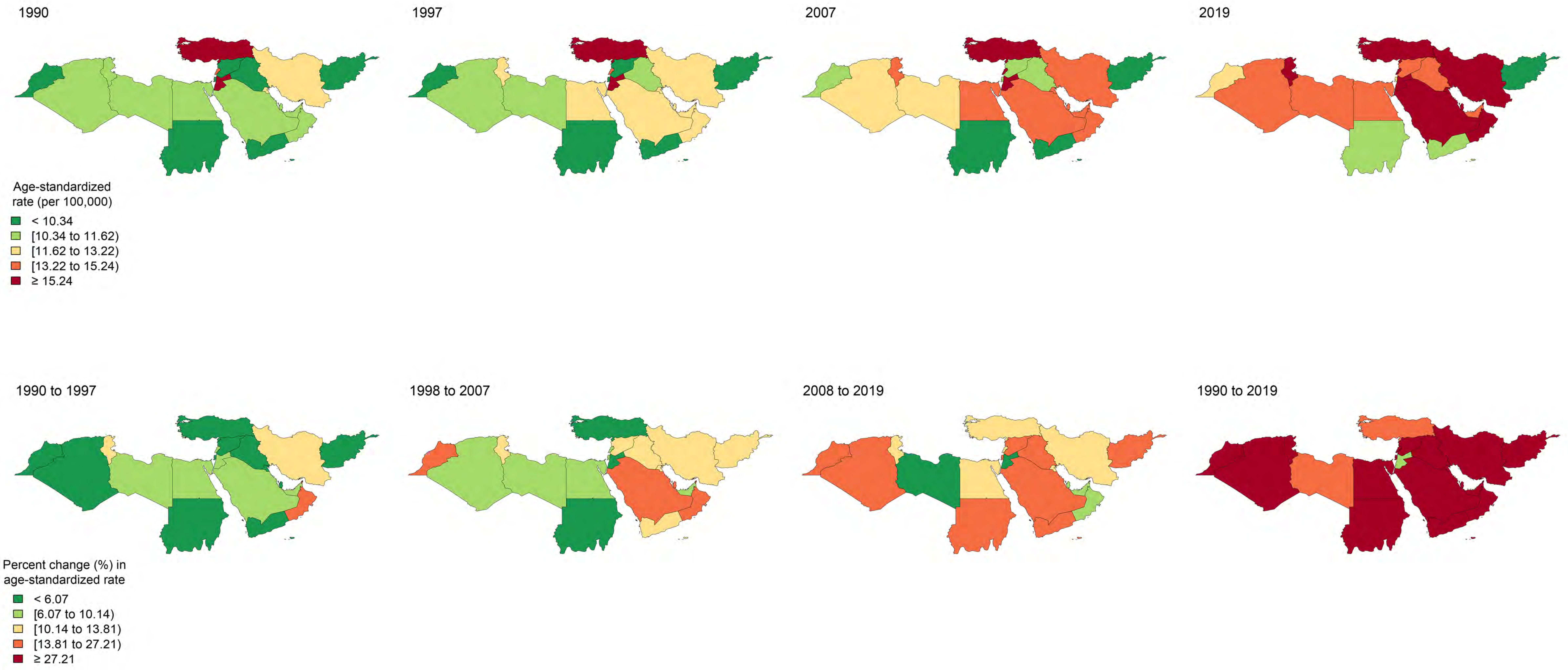

**S6 Fig.**

**Upper panel: Age-standardized prevalence rate (per 100,000) of infective endocarditis for the nations of North Africa and the Middle East in 1990, 1997, 2007, and 2019; Lower panel: percentage changes (%) in age-standardized prevalence rate for the countries of North Africa and the Middle East from 1990 to 1997, 1998 to 2007, and 2008 to 2019**

***Contains information from OpenStreetMap and OpenStreetMap Foundation, which is made available under the Open Database License.***

12

**Prevalence**

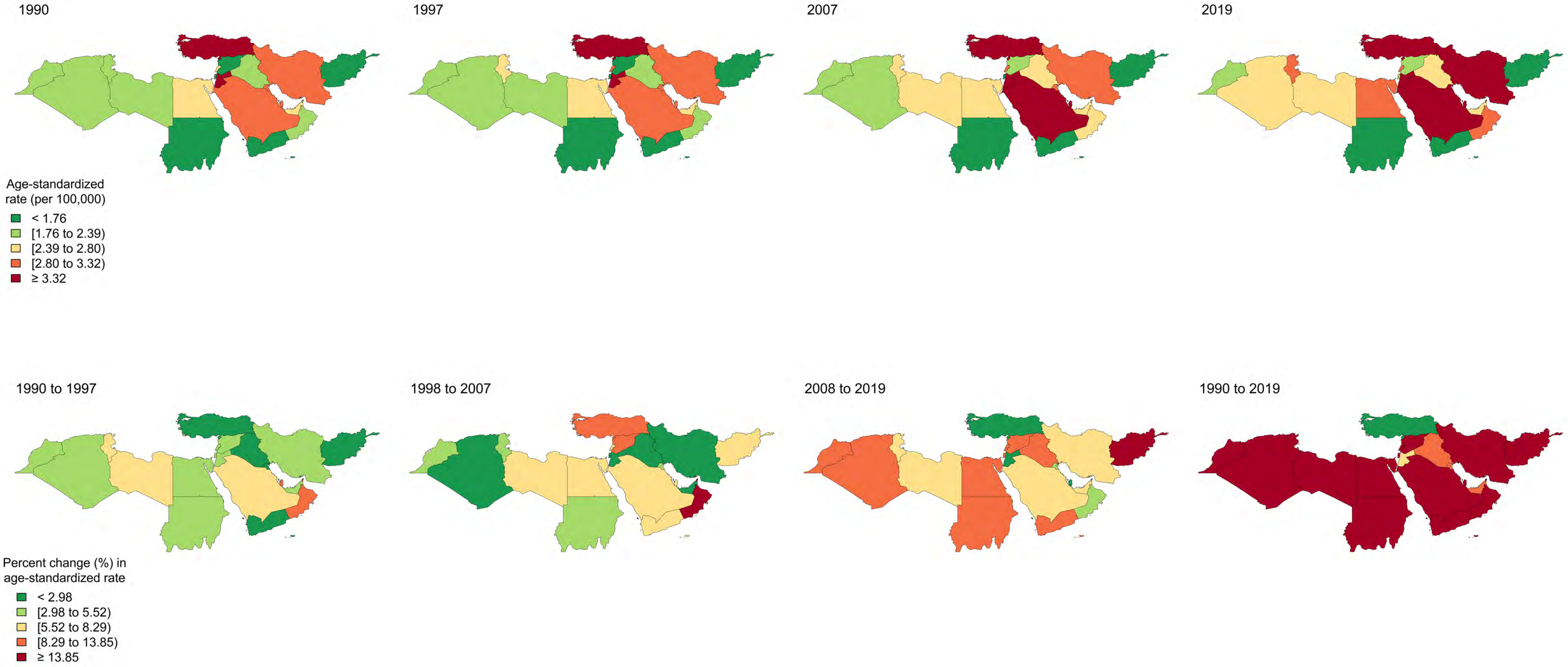

**S7 Fig.**

**Upper panel: Age-standardized death rate (per 100,000) of infective endocarditis for the nations of North Africa and the Middle East in 1990, 1997, 2007, and 2019; Lower panel: percentage changes (%) in age-standardized death rate for the countries of North Africa and the Middle East from 1990 to 1997, 1998 to 2007, and 2008 to 2019**

***Contains information from OpenStreetMap and OpenStreetMap Foundation, which is made available under the Open Database License.***

14

**Deaths**

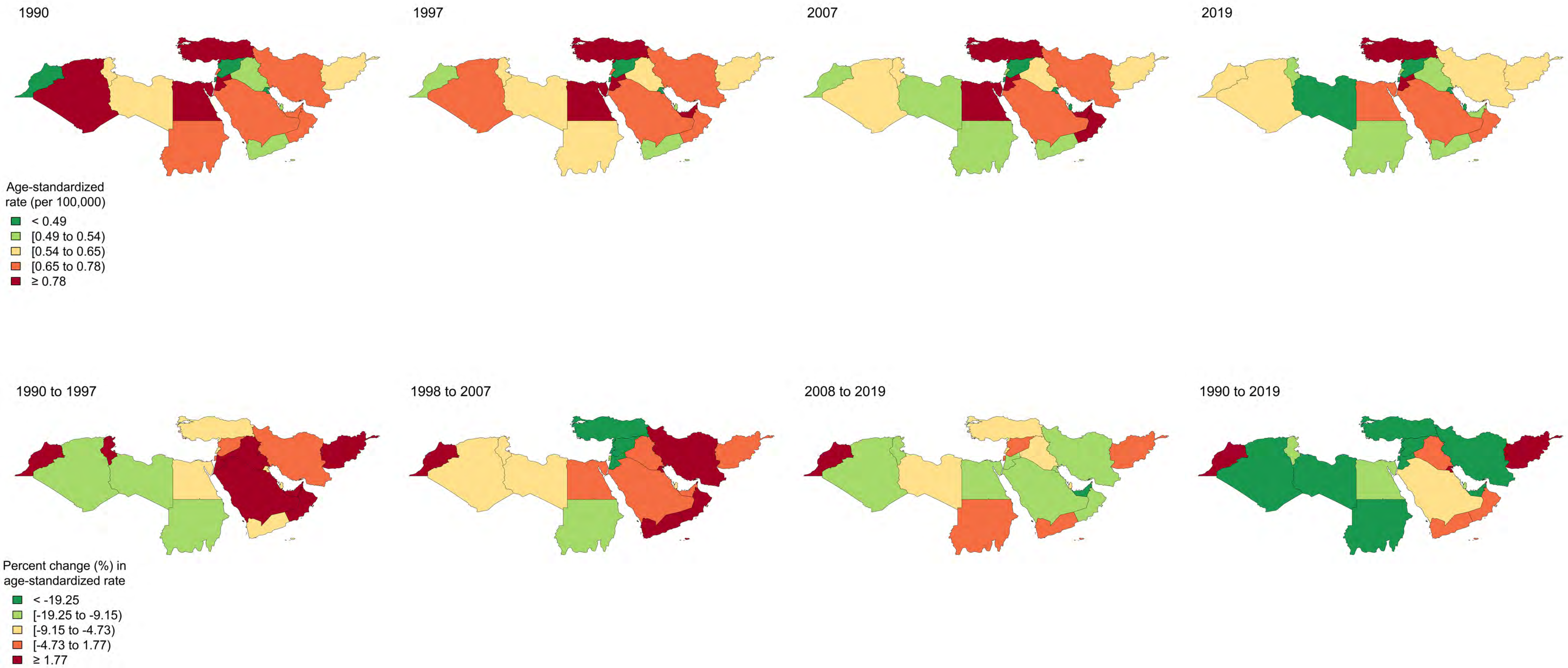
1990
1997
2007
2019
Age-standardized rate (per 100,000)
< 0.49
[0.49 to 0.54)
[0.54 to 0.65)
[0.65 to 0.78)
≥ 0.78
1990 to 1997
1998 to 2007
2008 to 2019
1990 to 2019
Percent change (%) in age-standardized rate
< -19.25
[-19.25 to -9.15)
[-9.15 to -4.73)
[-4.73 to 1.77)
≥ 1.77

## S8 Fig.

**Upper panel: Age-standardized rate (per 100, 000) of DALYs of infective endocarditis for the nations of North Africa and the Middle East in 1990, 1997, 2007, and 2019; Lower panel: Percentage changes (%) in age-standardized rate (per 100, 000) of DALYs in North Africa and the Middle East from 1990 to 1997, 1998 to 2007, and 2008 to 2019**

***Contains information from OpenStreetMap and OpenStreetMap Foundation, which is made available under the Open Database License.***

16

**DALYs**

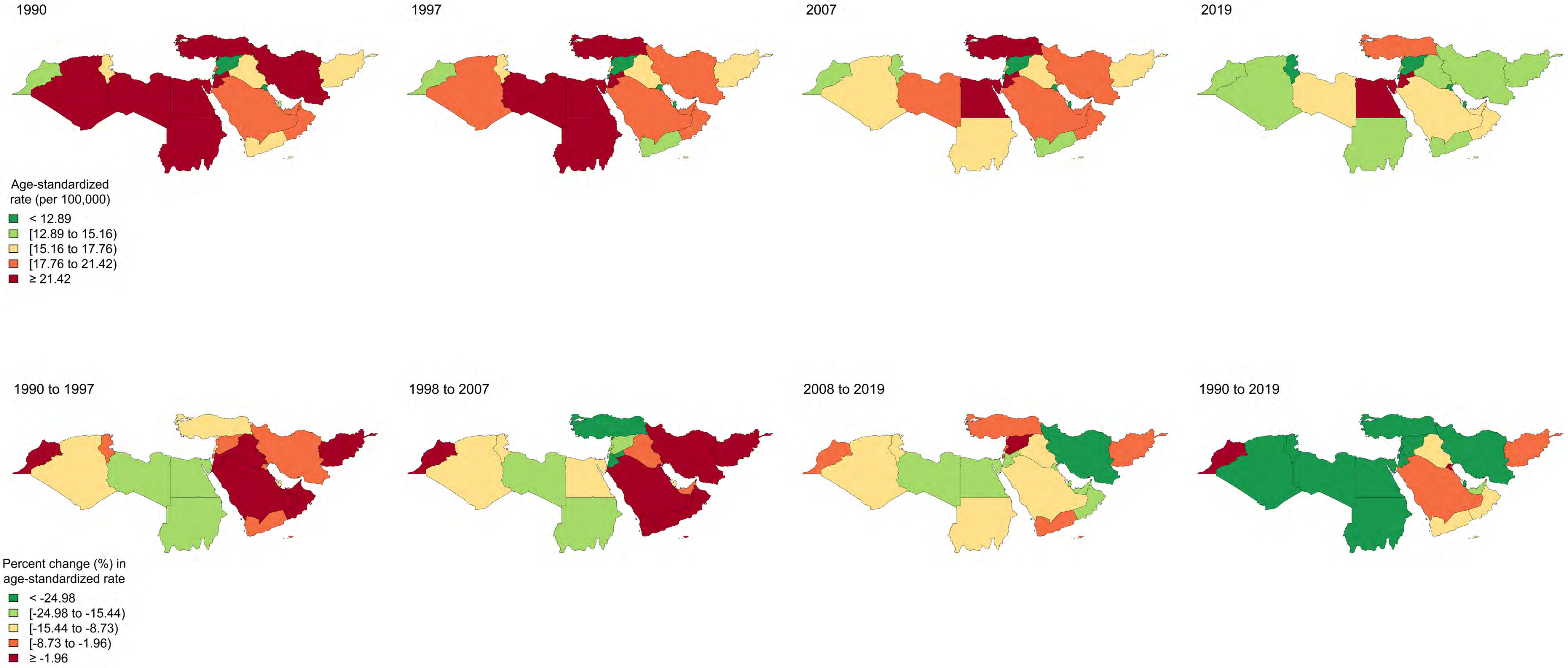
1990
1997
2007
2019
Age-standardized rate (per 100,000)
< 12.89
[12.89 to 15.16)
[15.16 to 17.76)
[17.76 to 21.42)
≥ 21.42
1990 to 1997
1998 to 2007
2008 to 2019
1990 to 2019
Percent change (%) in age-standardized rate
< -24.98
[-24.98 to -15.44)
[-15.44 to -8.73)
[-8.73 to -1.96)
≥ -1.96

**S9 Fig.**

**Upper panel: Age-standardized rate (per 100, 000) of YLLs of infective endocarditis for the nations of North Africa and the Middle East in 1990, 1997, 2007, and 2019; Lower panel: Percentage changes (%) in age-standardized rate (per 100, 000) of YLLs in North Africa and the Middle East from 1990 to 1997, 1998 to 2007, and 2008 to 2019**

***Contains information from OpenStreetMap and OpenStreetMap Foundation, which is made available under the Open Database License.***

**YLLs**

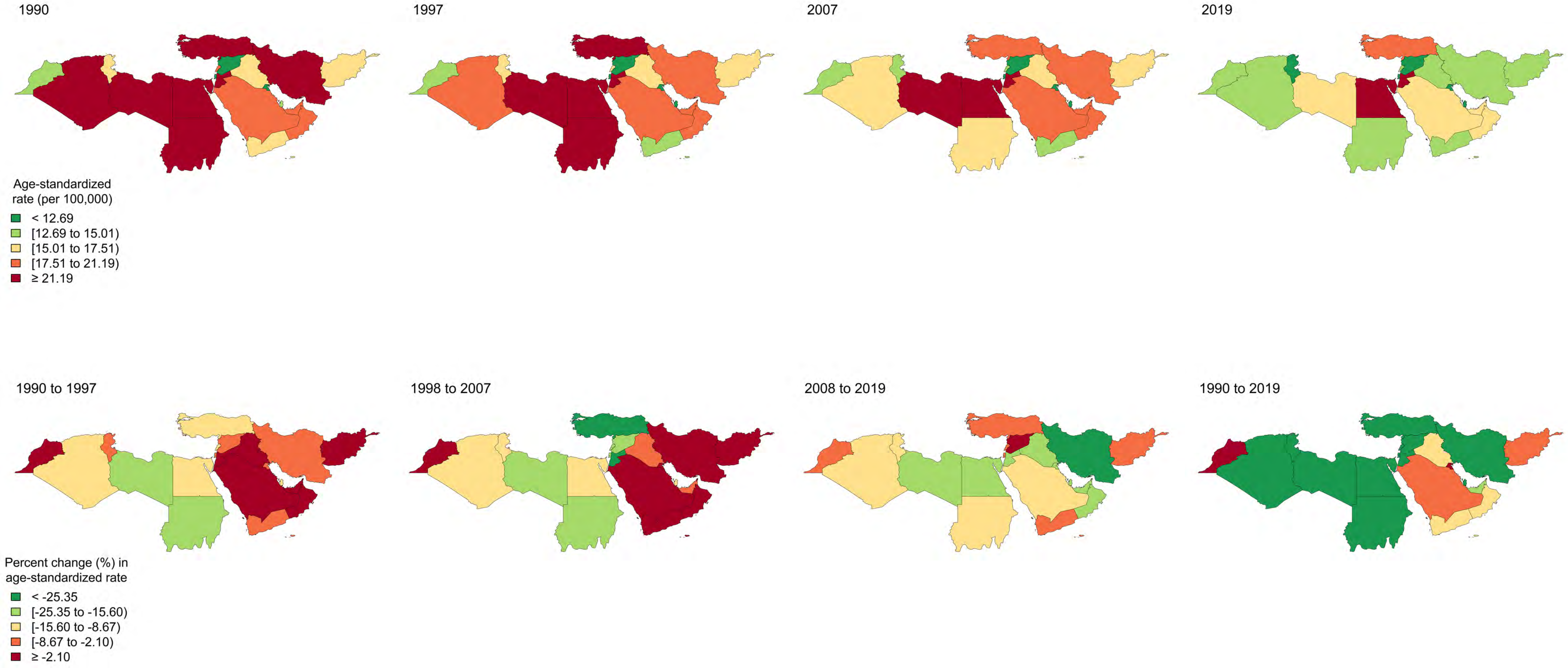
1990
1997
2007
2019
Age-standardized rate (per 100,000)
< 12.69
[12.69 to 15.01)
[15.01 to 17.51)
[17.51 to 21.19)
≥ 21.19
1990 to 1997
1998 to 2007
2008 to 2019
1990 to 2019
Percent change (%) in age-standardized rate
< -25.35
[-25.35 to -15.60)
[-15.60 to -8.67)
[-8.67 to -2.10)
≥ -2.10

**S10 Fig.**

**Upper panel: Age-standardized rate (per 100, 000) of YLDs of infective endocarditis for the nations of North Africa and the Middle East in 1990, 1997, 2007, and 2019; Lower panel: Percentage changes (%) in age-standardized rate (per 100, 000) of YLDs in North Africa and the Middle East from 1990 to 1997, 1998 to 2007, and 2008 to 2019**

***Contains information from OpenStreetMap and OpenStreetMap Foundation, which is made available under the Open Database License.***

**YLDs**

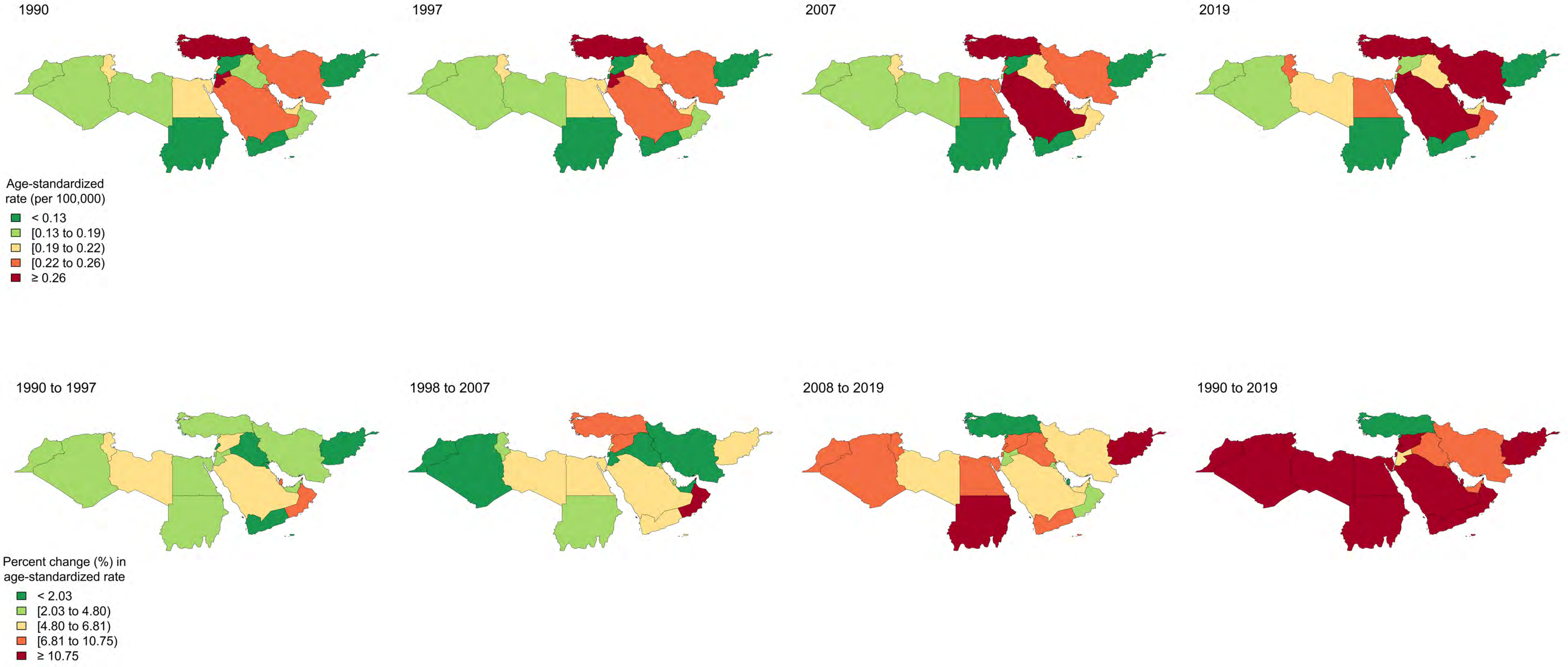
1990
1997
2007
2019
Age-standardized rate (per 100,000)
< 0.13
[0.13 to 0.19)
[0.19 to 0.22)
[0.22 to 0.26)
≥ 0.26
1990 to 1997
1998 to 2007
2008 to 2019
1990 to 2019
Percent change (%) in age-standardized rate
< 2.03
[2.03 to 4.80)
[4.80 to 6.81)
[6.81 to 10.75)
≥ 10.75

## S11 Fig.

**Comparison of rates (per 100,000) of incidence, prevalence, deaths, and DALYs of infective endocarditis in North Africa and the Middle East in 1990 and 2019 according to eighteen age groups and sex**

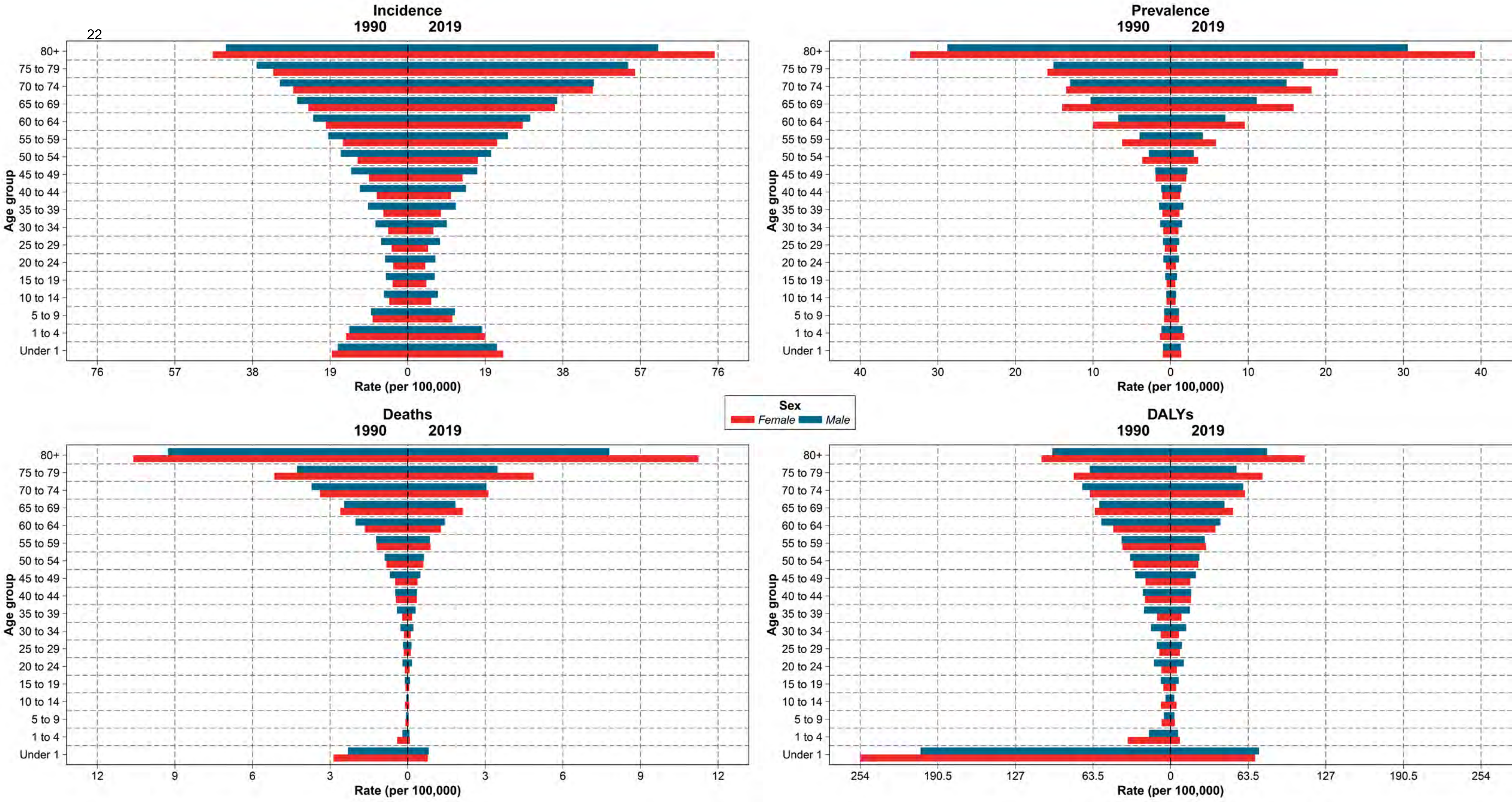
Incidence
1990 2019
Prevalence
1990 2019
Deaths
1990 2019
DALYs
1990 2019
Sex
Female
Male
Age group
80+
75 to 79
70 to 74
65 to 69
60 to 64
55 to 59
50 to 54
45 to 49
40 to 44
35 to 39
30 to 34
25 to 29
20 to 24
15 to 19
10 to 14
5 to 9
1 to 4
Under 1
76 57 38 19 0 19 38 57 76
40 30 20 10 0 10 20 30 40
12 9 6 3 0 3 6 9 12
254 190.5 127 63.5 0 63.5 127 190.5 254
Rate (per 100,000)

**S12 Fig.**

**Rankings of 21 countries of  North African and Middle Eastern countries based on age-standardized rates of incidence, prevalence, deaths, and DALYs of infective endocarditis in 1990 and 2019**

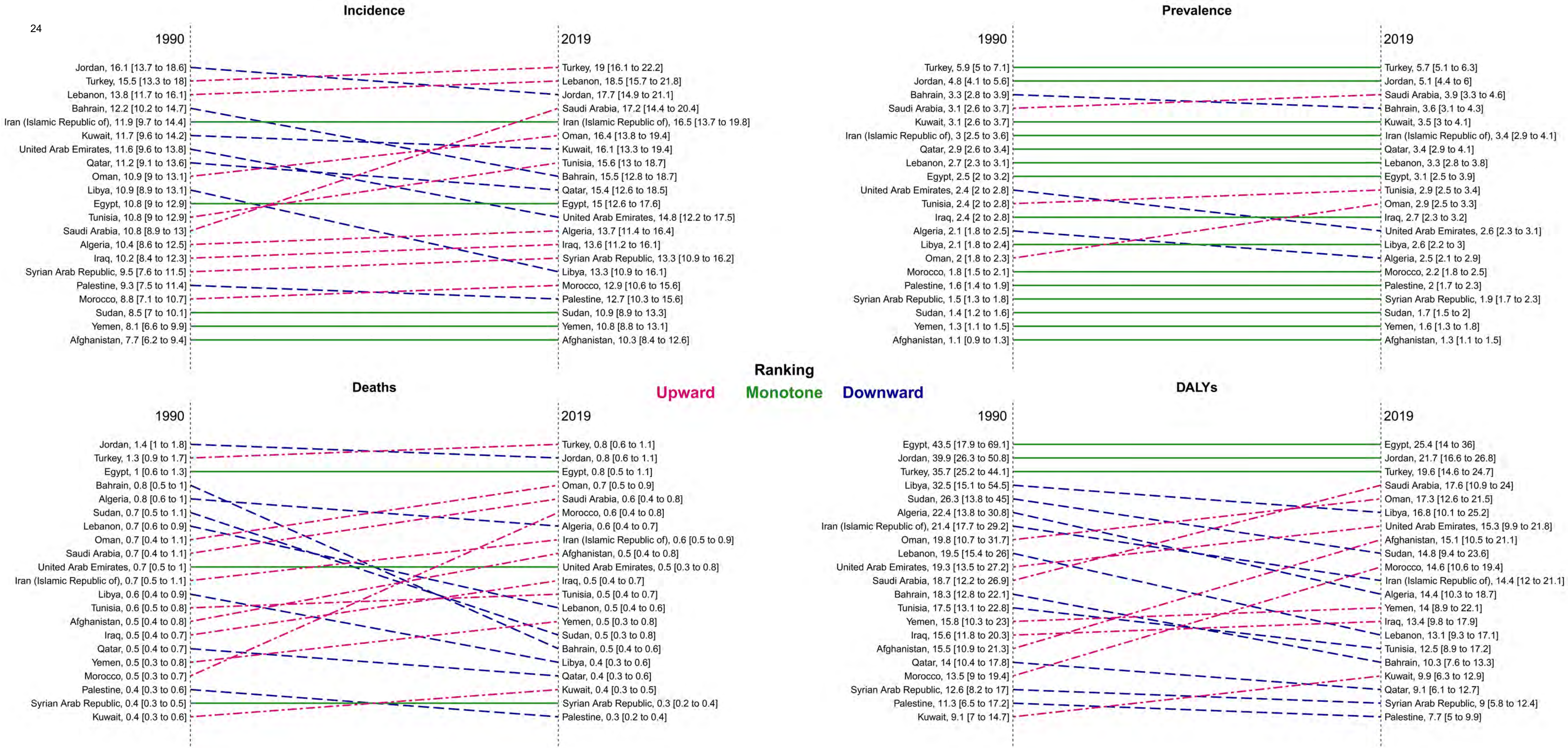
Incidence
1990
2019
Jordan, 16.1 [13.7 to 18.6]
Turkey, 15.5 [13.3 to 18]
Lebanon, 13.8 [11.7 to 16.1]
Bahrain, 12.2 [10.2 to 14.7]
Iran (Islamic Republic of), 11.9 [9.7 to 14.4]
Kuwait, 11.7 [9.6 to 14.2]
United Arab Emirates, 11.6 [9.6 to 13.8]
Qatar, 11.2 [9.1 to 13.6]
Oman, 10.9 [9 to 13.1]
Libya, 10.9 [8.9 to 13.1]
Egypt, 10.8 [9 to 12.9]
Tunisia, 10.8 [9 to 12.9]
Saudi Arabia, 10.8 [8.9 to 13]
Algeria, 10.4 [8.6 to 12.5]
Iraq, 10.2 [8.4 to 12.3]
Syrian Arab Republic, 9.5 [7.6 to 11.5]
Palestine, 9.3 [7.5 to 11.4]
Morocco, 8.8 [7.1 to 10.7]
Sudan, 8.5 [7 to 10.1]
Yemen, 8.1 [6.6 to 9.9]
Afghanistan, 7.7 [6.2 to 9.4]
Turkey, 19 [16.1 to 22.2]
Lebanon, 18.5 [15.7 to 21.8]
Jordan, 17.7 [14.9 to 21.1]
Saudi Arabia, 17.2 [14.4 to 20.4]
Iran (Islamic Republic of), 16.5 [13.7 to 19.8]
Oman, 16.4 [13.8 to 19.4]
Kuwait, 16.1 [13.3 to 19.4]
Tunisia, 15.6 [13 to 18.7]
Bahrain, 15.5 [12.8 to 18.7]
Qatar, 15.4 [12.6 to 18.5]
Egypt, 15 [12.6 to 17.6]
United Arab Emirates, 14.8 [12.2 to 17.5]
Algeria, 13.7 [11.4 to 16.4]
Iraq, 13.6 [11.2 to 16.1]
Syrian Arab Republic, 13.3 [10.9 to 16.2]
Libya, 13.3 [10.9 to 16.1]
Morocco, 12.9 [10.6 to 15.6]
Palestine, 12.7 [10.3 to 15.6]
Sudan, 10.9 [8.9 to 13.3]
Yemen, 10.8 [8.8 to 13.1]
Afghanistan, 10.3 [8.4 to 12.6]
Prevalence
1990
2019
Turkey, 5.9 [5 to 7.1]
Jordan, 4.8 [4.1 to 5.6]
Bahrain, 3.3 [2.8 to 3.9]
Saudi Arabia, 3.1 [2.6 to 3.7]
Kuwait, 3.1 [2.6 to 3.7]
Iran (Islamic Republic of), 3 [2.5 to 3.6]
Qatar, 2.9 [2.6 to 3.4]
Lebanon, 2.7 [2.3 to 3.1]
Egypt, 2.5 [2 to 3.2]
United Arab Emirates, 2.4 [2 to 2.8]
Tunisia, 2.4 [2 to 2.8]
Iraq, 2.4 [2 to 2.8]
Algeria, 2.1 [1.8 to 2.5]
Libya, 2.1 [1.8 to 2.4]
Oman, 2 [1.8 to 2.3]
Morocco, 1.8 [1.5 to 2.1]
Palestine, 1.6 [1.4 to 1.9]
Syrian Arab Republic, 1.5 [1.3 to 1.8]
Sudan, 1.4 [1.2 to 1.6]
Yemen, 1.3 [1.1 to 1.5]
Afghanistan, 1.1 [0.9 to 1.3]
Turkey, 5.7 [5.1 to 6.3]
Jordan, 5.1 [4.4 to 6]
Saudi Arabia, 3.9 [3.3 to 4.6]
Bahrain, 3.6 [3.1 to 4.3]
Kuwait, 3.5 [3 to 4.1]
Iran (Islamic Republic of), 3.4 [2.9 to 4.1]
Qatar, 3.4 [2.9 to 4.1]
Lebanon, 3.3 [2.8 to 3.8]
Egypt, 3.1 [2.5 to 3.9]
Tunisia, 2.9 [2.5 to 3.4]
Oman, 2.9 [2.5 to 3.3]
Iraq, 2.7 [2.3 to 3.2]
United Arab Emirates, 2.6 [2.3 to 3.1]
Libya, 2.6 [2.2 to 3]
Algeria, 2.5 [2.1 to 2.9]
Morocco, 2.2 [1.8 to 2.5]
Palestine, 2 [1.7 to 2.3]
Syrian Arab Republic, 1.9 [1.7 to 2.3]
Sudan, 1.7 [1.5 to 2]
Yemen, 1.6 [1.3 to 1.8]
Afghanistan, 1.3 [1.1 to 1.5]
Ranking
Upward
Monotone
Downward
Deaths
1990
2019
Jordan, 1.4 [1 to 1.8]
Turkey, 1.3 [0.9 to 1.7]
Egypt, 1 [0.6 to 1.3]
Bahrain, 0.8 [0.5 to 1]
Algeria, 0.8 [0.6 to 1]
Sudan, 0.7 [0.5 to 1.1]
Lebanon, 0.7 [0.6 to 0.9]
Oman, 0.7 [0.4 to 1.1]
Saudi Arabia, 0.7 [0.4 to 1.1]
United Arab Emirates, 0.7 [0.5 to 1]
Iran (Islamic Republic of), 0.7 [0.5 to 1.1]
Libya, 0.6 [0.4 to 0.9]
Tunisia, 0.6 [0.5 to 0.8]
Afghanistan, 0.5 [0.4 to 0.8]
Iraq, 0.5 [0.4 to 0.7]
Qatar, 0.5 [0.4 to 0.7]
Yemen, 0.5 [0.3 to 0.8]
Morocco, 0.5 [0.3 to 0.7]
Palestine, 0.4 [0.3 to 0.6]
Syrian Arab Republic, 0.4 [0.3 to 0.5]
Kuwait, 0.4 [0.3 to 0.6]
Turkey, 0.8 [0.6 to 1.1]
Jordan, 0.8 [0.6 to 1.1]
Egypt, 0.8 [0.5 to 1.1]
Oman, 0.7 [0.5 to 0.9]
Saudi Arabia, 0.6 [0.4 to 0.8]
Morocco, 0.6 [0.4 to 0.8]
Algeria, 0.6 [0.4 to 0.7]
Iran (Islamic Republic of), 0.6 [0.5 to 0.9]
Afghanistan, 0.5 [0.4 to 0.8]
United Arab Emirates, 0.5 [0.3 to 0.8]
Iraq, 0.5 [0.4 to 0.7]
Tunisia, 0.5 [0.4 to 0.7]
Lebanon, 0.5 [0.4 to 0.6]
Yemen, 0.5 [0.3 to 0.8]
Sudan, 0.5 [0.3 to 0.8]
Bahrain, 0.5 [0.4 to 0.6]
Libya, 0.4 [0.3 to 0.6]
Qatar, 0.4 [0.3 to 0.6]
Kuwait, 0.4 [0.3 to 0.5]
Syrian Arab Republic, 0.3 [0.2 to 0.4]
Palestine, 0.3 [0.2 to 0.4]
DALYs
1990
2019
Egypt, 43.5 [17.9 to 69.1]
Jordan, 39.9 [26.3 to 50.8]
Turkey, 35.7 [25.2 to 44.1]
Libya, 32.5 [15.1 to 54.5]
Sudan, 26.3 [13.8 to 45]
Algeria, 22.4 [13.8 to 30.8]
Iran (Islamic Republic of), 21.4 [17.7 to 29.2]
Oman, 19.8 [10.7 to 31.7]
Lebanon, 19.5 [15.4 to 26]
United Arab Emirates, 19.3 [13.5 to 27.2]
Saudi Arabia, 18.7 [12.2 to 26.9]
Bahrain, 18.3 [12.8 to 22.1]
Tunisia, 17.5 [13.1 to 22.8]
Yemen, 15.8 [10.3 to 23]
Iraq, 15.6 [11.8 to 20.3]
Afghanistan, 15.5 [10.9 to 21.3]
Qatar, 14 [10.4 to 17.8]
Morocco, 13.5 [9 to 19.4]
Syrian Arab Republic, 12.6 [8.2 to 17]
Palestine, 11.3 [6.5 to 17.2]
Kuwait, 9.1 [7 to 14.7]
Egypt, 25.4 [14 to 36]
Jordan, 21.7 [16.6 to 26.8]
Turkey, 19.6 [14.6 to 24.7]
Saudi Arabia, 17.6 [10.9 to 24]
Oman, 17.3 [12.6 to 21.5]
Libya, 16.8 [10.1 to 25.2]
United Arab Emirates, 15.3 [9.9 to 21.8]
Afghanistan, 15.1 [10.5 to 21.1]
Sudan, 14.8 [9.4 to 23.6]
Morocco, 14.6 [10.6 to 19.4]
Iran (Islamic Republic of), 14.4 [12 to 21.1]
Algeria, 14.4 [10.3 to 18.7]
Yemen, 14 [8.9 to 22.1]
Iraq, 13.4 [9.8 to 17.9]
Lebanon, 13.1 [9.3 to 17.1]
Tunisia, 12.5 [8.9 to 17.2]
Bahrain, 10.3 [7.6 to 13.3]
Kuwait, 9.9 [6.3 to 12.9]
Qatar, 9.1 [6.1 to 12.7]
Syrian Arab Republic, 9 [5.8 to 12.4]
Palestine, 7.7 [5 to 9.9]

**S13 Fig.**

**Age-standardized rates (per 100,000) of YLDs and YLLs of infective endocarditis in 21 North African and Middle Eastern countries in 2019 by quintiles of the countries' socio-demographic index (SDI)**

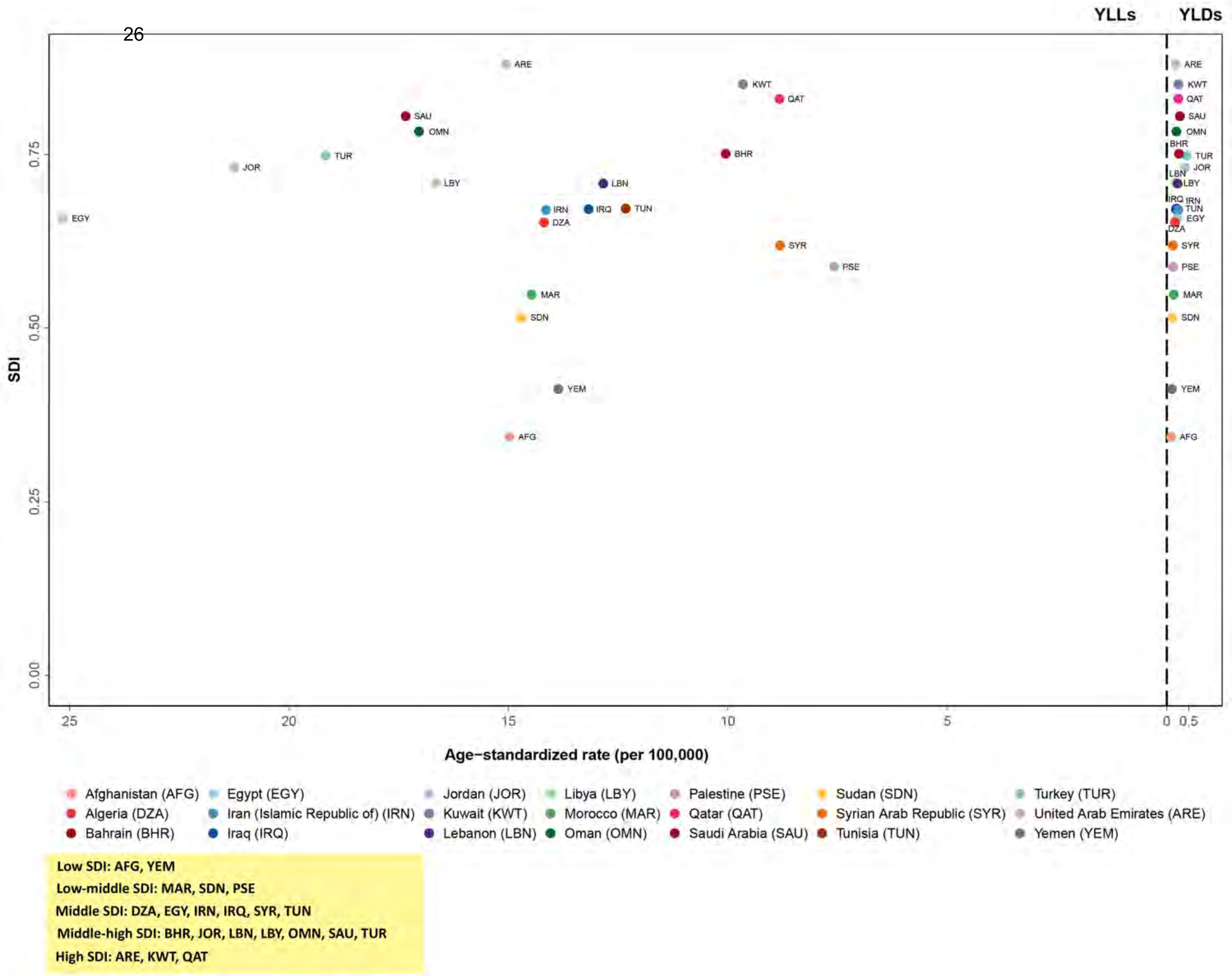
YLLs
YLDs
SDI
0.00
0.25
0.50
0.75
25
20
15
10
5
0
0.5
Age−standardized rate (per 100,000)
ARE
KWT
QAT
SAU
OMN
TUR
BHR
JOR
LBY
LBN
IRN
IRQ
TUN
DZA
EGY
SYR
PSE
MAR
SDN
YEM
AFG
Afghanistan (AFG)
Algeria (DZA)
Bahrain (BHR)
Egypt (EGY)
Iran (Islamic Republic of) (IRN)
Iraq (IRQ)
Jordan (JOR)
Kuwait (KWT)
Lebanon (LBN)
Libya (LBY)
Morocco (MAR)
Oman (OMN)
Palestine (PSE)
Qatar (QAT)
Saudi Arabia (SAU)
Sudan (SDN)
Syrian Arab Republic (SYR)
Tunisia (TUN)
Turkey (TUR)
United Arab Emirates (ARE)
Yemen (YEM)
Low SDI: AFG, YEM
Low-middle SDI: MAR, SDN, PSE
Middle SDI: DZA, EGY, IRN, IRQ, SYR, TUN
Middle-high SDI: BHR, JOR, LBN, LBY, OMN, SAU, TUR
High SDI: ARE, KWT, QAT

**S14 Fig.**

**The temporal trend of age-standardized incidence, prevalence, deaths, and DALYs of infective endocarditis in 21 countries of North Africa and the Middle East between 1990 and 2019 in the three eras (1990–1997, 1998–2007, and 2008–2019)**

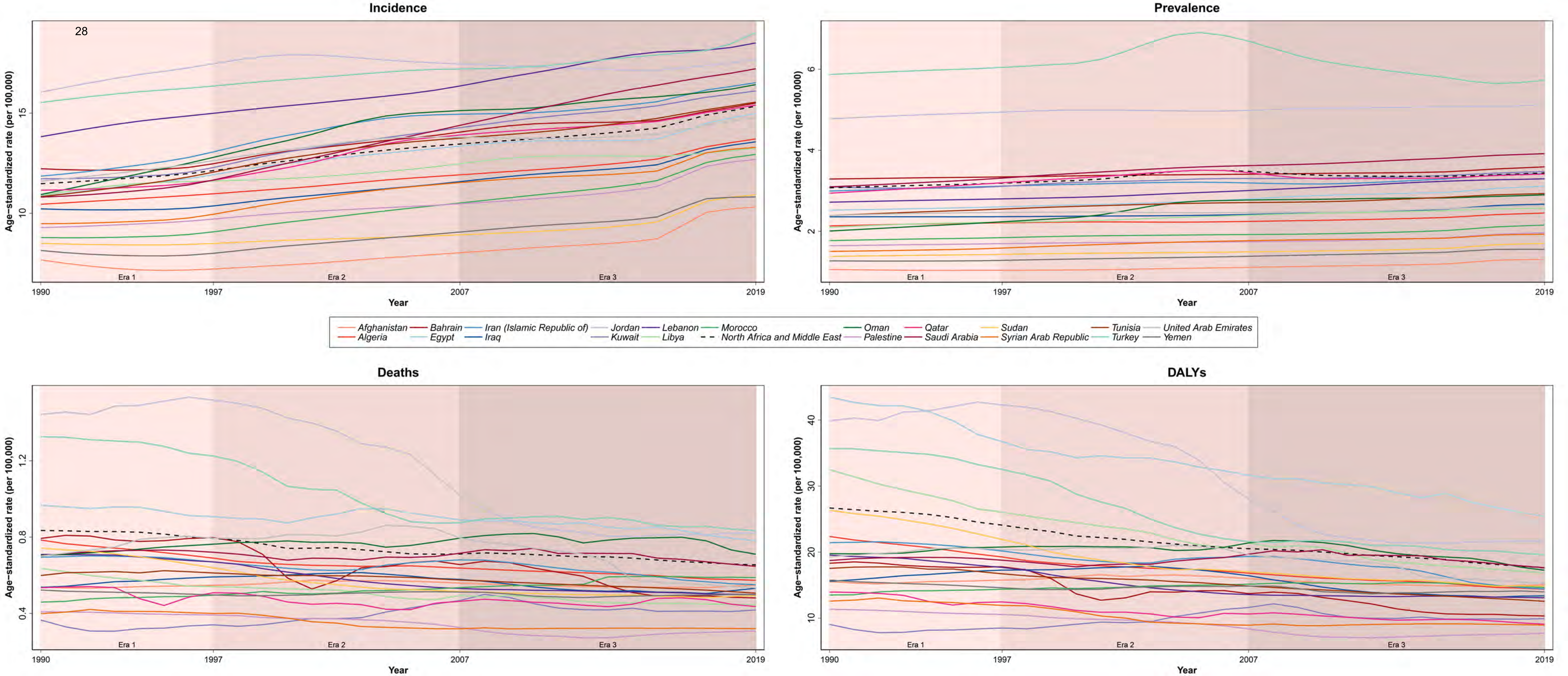
Incidence
Prevalence
Deaths
DALYs
Age−standardized rate (per 100,000)
Year
1990
1997
2007
2019
Era 1
Era 2
Era 3
Afghanistan
Algeria
Bahrain
Egypt
Iran (Islamic Republic of)
Iraq
Jordan
Kuwait
Lebanon
Libya
Morocco
North Africa and Middle East
Oman
Palestine
Qatar
Saudi Arabia
Sudan
Syrian Arab Republic
Tunisia
Turkey
United Arab Emirates
Yemen

## S15 Fig.

**Percent changes (%) in age-standardized rates (per 100,000) of incidence, prevalence, deaths, and DALYs of infective endocarditis between 1997 and 2007, and 2007 and 2019 in 21 countries of North Africa and the Middle East**

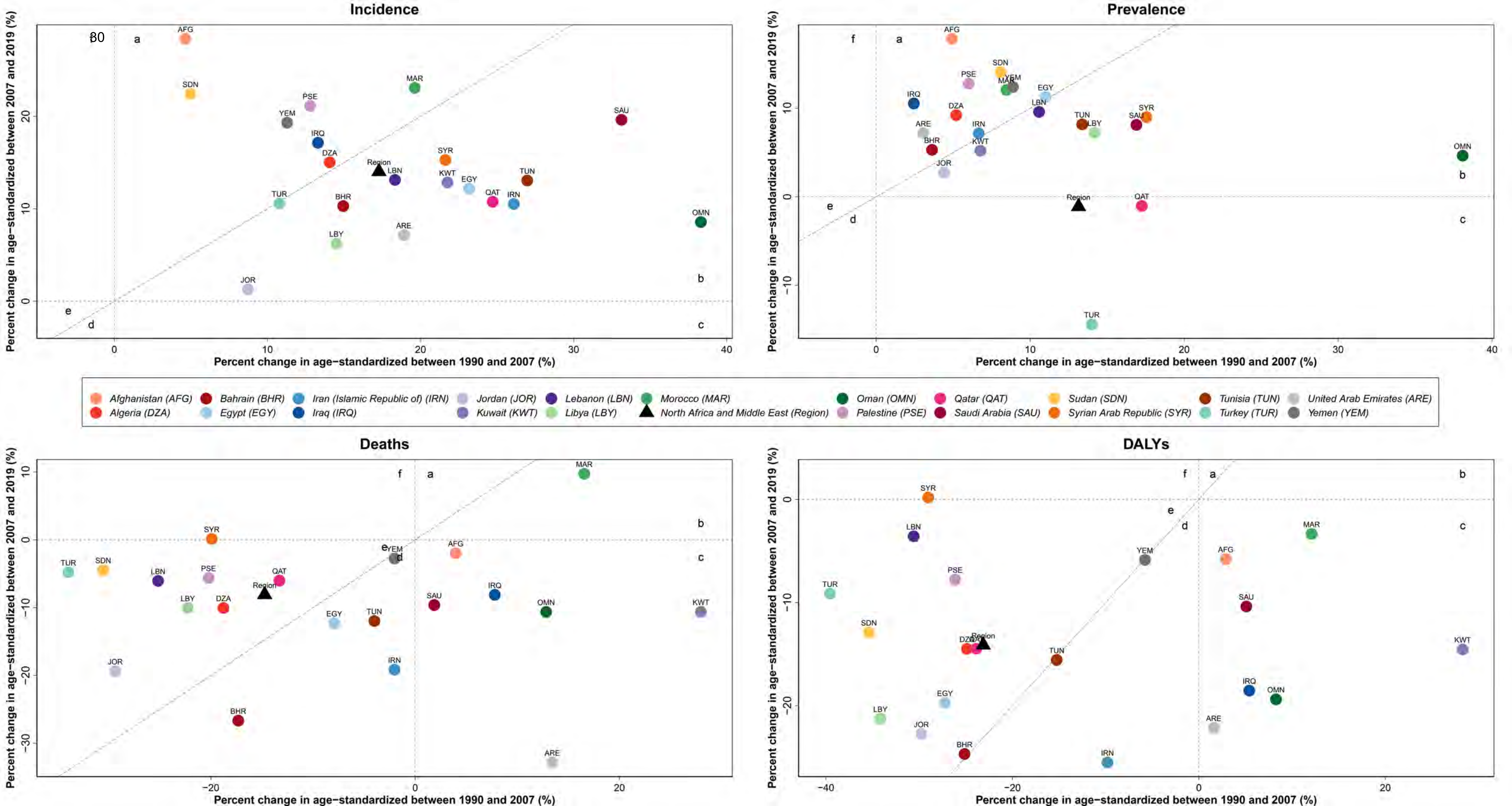

Incidence
Prevalence
Deaths
DALYs
Percent change in age−standardized between 2007 and 2019 (%)
Percent change in age−standardized between 1990 and 2007 (%)
Afghanistan (AFG)
Algeria (DZA)
Bahrain (BHR)
Egypt (EGY)
Iran (Islamic Republic of) (IRN)
Iraq (IRQ)
Jordan (JOR)
Kuwait (KWT)
Lebanon (LBN)
Libya (LBY)
Morocco (MAR)
North Africa and Middle East (Region)
Oman (OMN)
Palestine (PSE)
Qatar (QAT)
Saudi Arabia (SAU)
Sudan (SDN)
Syrian Arab Republic (SYR)
Tunisia (TUN)
Turkey (TUR)
United Arab Emirates (ARE)
Yemen (YEM)

**S16 Fig.**

**Age-standardized rates (per 100,000) of incidence, prevalence, deaths, and disability-adjusted life years (DALYs) of infective endocarditis in 21 North African and Middle Eastern countries in 1990, 1997, 2007, and 2019 by quintiles of the countries' socio-demographic index**

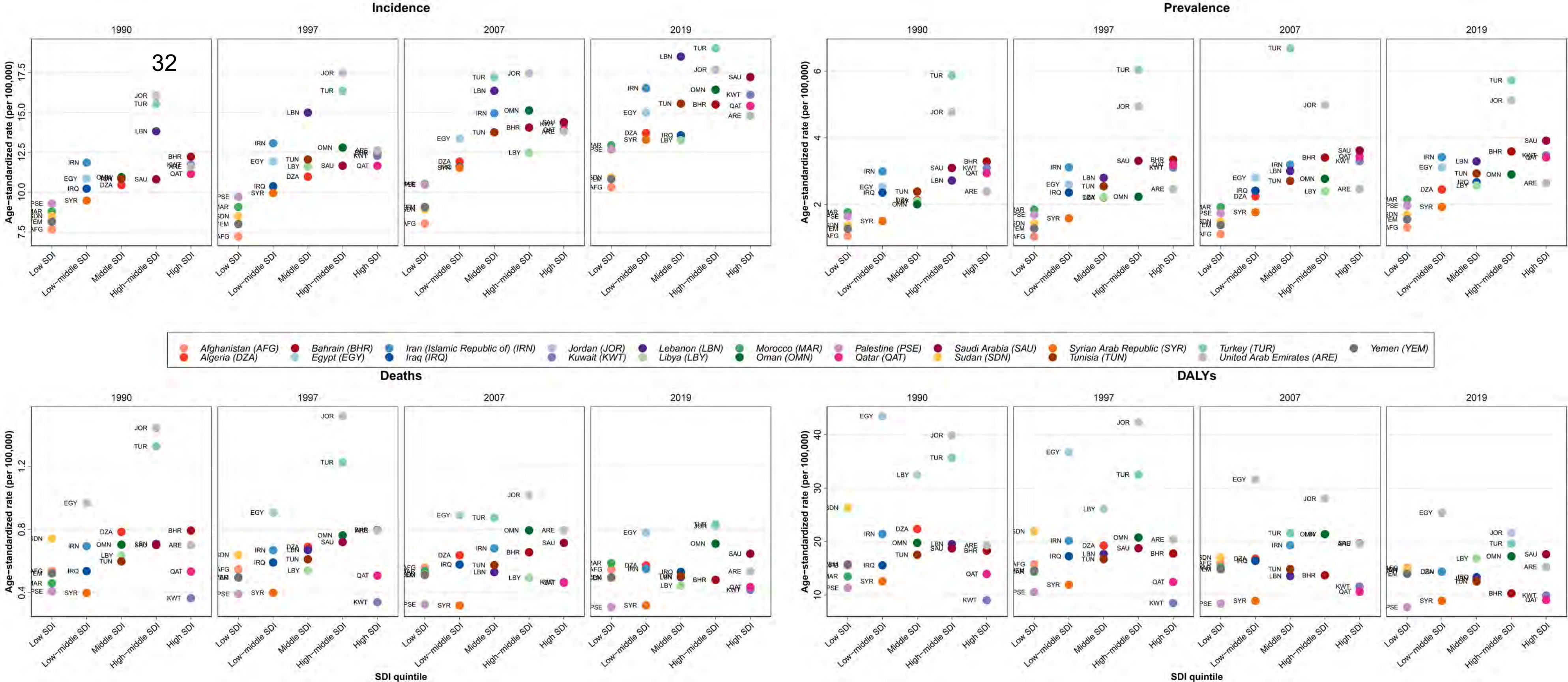

Incidence
Prevalence
Deaths
DALYs
1990
1997
2007
2019
Age−standardized rate (per 100,000)
Low SDI
Low−middle SDI
Middle SDI
High−middle SDI
High SDI
SDI quintile
Afghanistan (AFG)
Algeria (DZA)
Bahrain (BHR)
Egypt (EGY)
Iran (Islamic Republic of) (IRN)
Iraq (IRQ)
Jordan (JOR)
Kuwait (KWT)
Lebanon (LBN)
Libya (LBY)
Morocco (MAR)
Oman (OMN)
Palestine (PSE)
Qatar (QAT)
Saudi Arabia (SAU)
Sudan (SDN)
Syrian Arab Republic (SYR)
Tunisia (TUN)
Turkey (TUR)
United Arab Emirates (ARE)
Yemen (YEM)

**S17 Fig.**

**Age-standardized rates (per 100,000) of incidence, prevalence, deaths, and disability-adjusted life years (DALYs) of infective endocarditis in 21 North African and Middle Eastern nations from 1990 to 2019 at three-year intervals (1990, 1993, 1996, 1999, 2002, 2005, 2008, 2011, 2014, 2017 and 2019) by quintiles of the countries' sociodemographic index (SDI)**

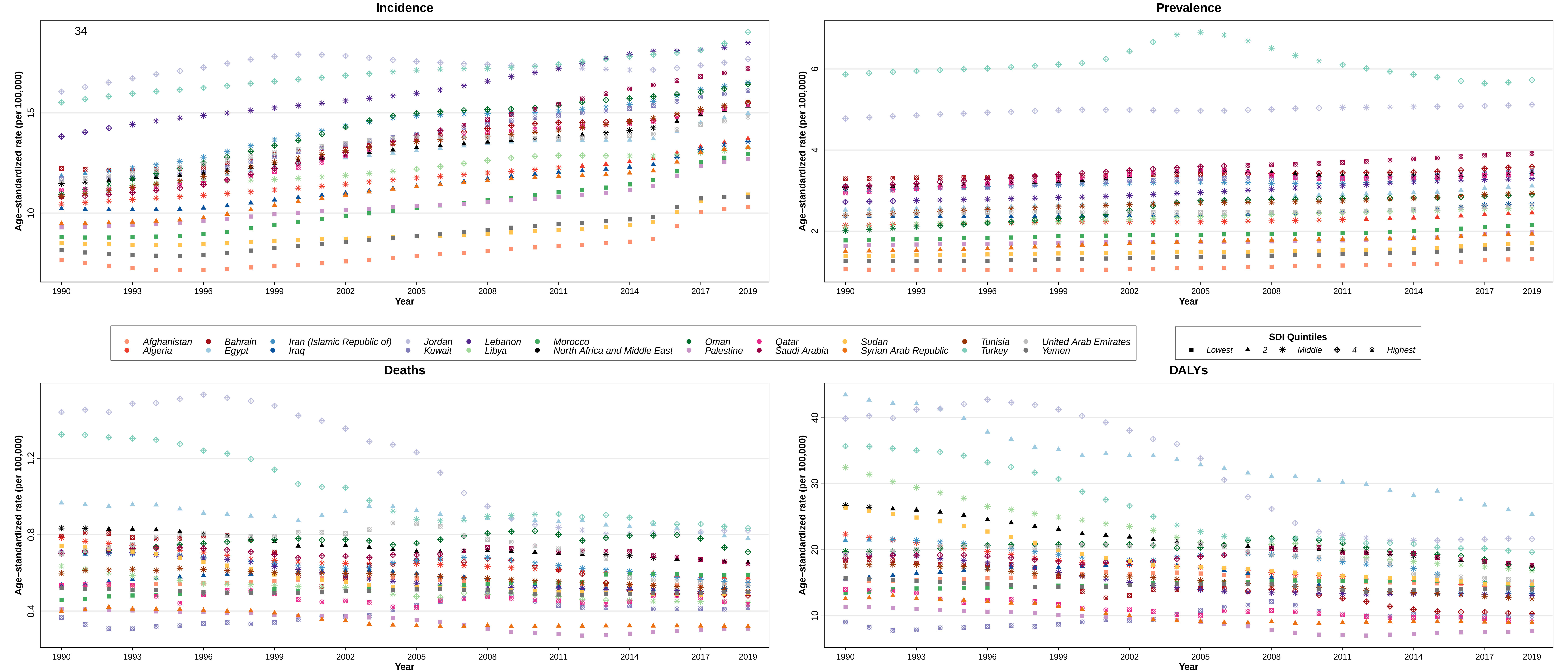

34
Incidence
Prevalence
Deaths
DALYs
Age−standardized rate (per 100,000)
Year
Afghanistan
Algeria
Bahrain
Egypt
Iran (Islamic Republic of)
Iraq
Jordan
Kuwait
Lebanon
Libya
Morocco
North Africa and Middle East
Oman
Palestine
Qatar
Saudi Arabia
Sudan
Syrian Arab Republic
Tunisia
Turkey
United Arab Emirates
Yemen
SDI Quintiles
Lowest
2
Middle
4
Highest

**S18 Fig.**

**Rates (per 100,000) of incidence, prevalence, deaths, and DALYs of infective endocarditis in 21 countries of North Africa and the Middle East in 1990 compared with 1997, in 1998 versus 2007, and in 2008 versus 2019 according to eighteen age groups and sex**

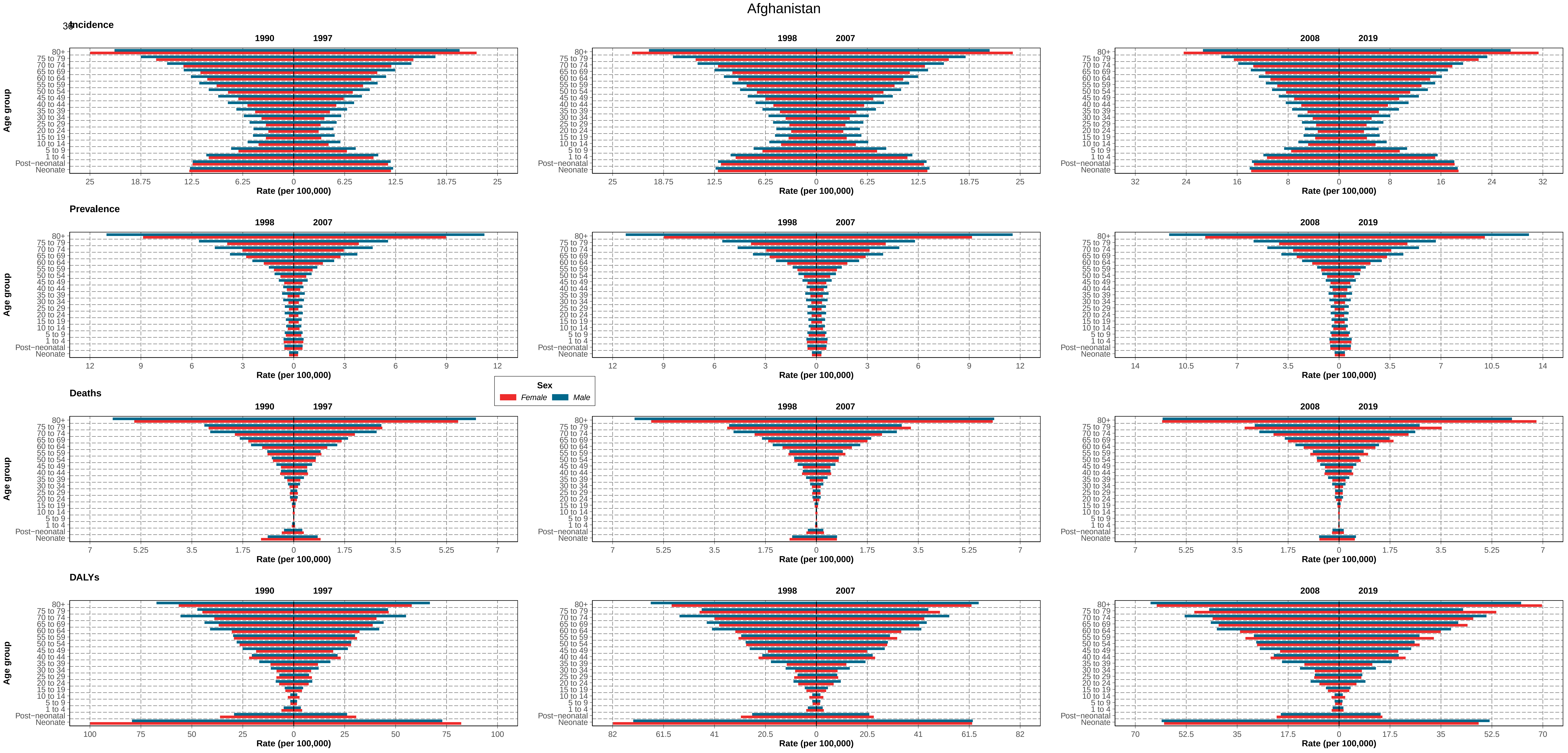
Afghanistan
Incidence
Prevalence
Deaths
DALYs
1990
1997
1998
2007
2008
2019
Age group
Rate (per 100,000)
80+
75 to 79
70 to 74
65 to 69
60 to 64
55 to 59
50 to 54
45 to 49
40 to 44
35 to 39
30 to 34
25 to 29
20 to 24
15 to 19
10 to 14
5 to 9
1 to 4
Post−neonatal
Neonate
Sex
Female
Male

# Algeria

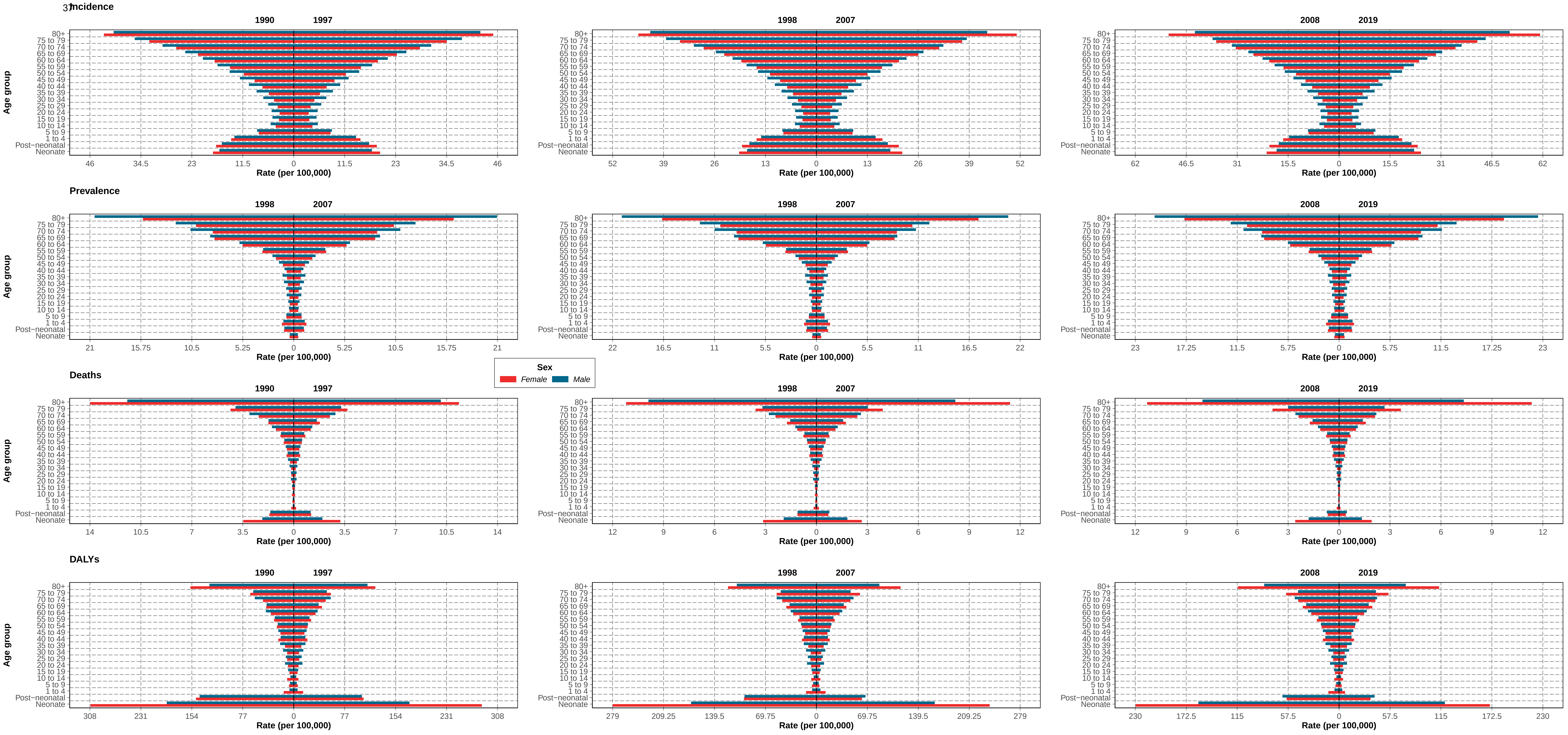
Incidence
Prevalence
Deaths
DALYs
1990
1997
1998
2007
2008
2019
Age group
80+
75 to 79
70 to 74
65 to 69
60 to 64
55 to 59
50 to 54
45 to 49
40 to 44
35 to 39
30 to 34
25 to 29
20 to 24
15 to 19
10 to 14
5 to 9
1 to 4
Post−neonatal
Neonate
Rate (per 100,000)
Sex
Female
Male

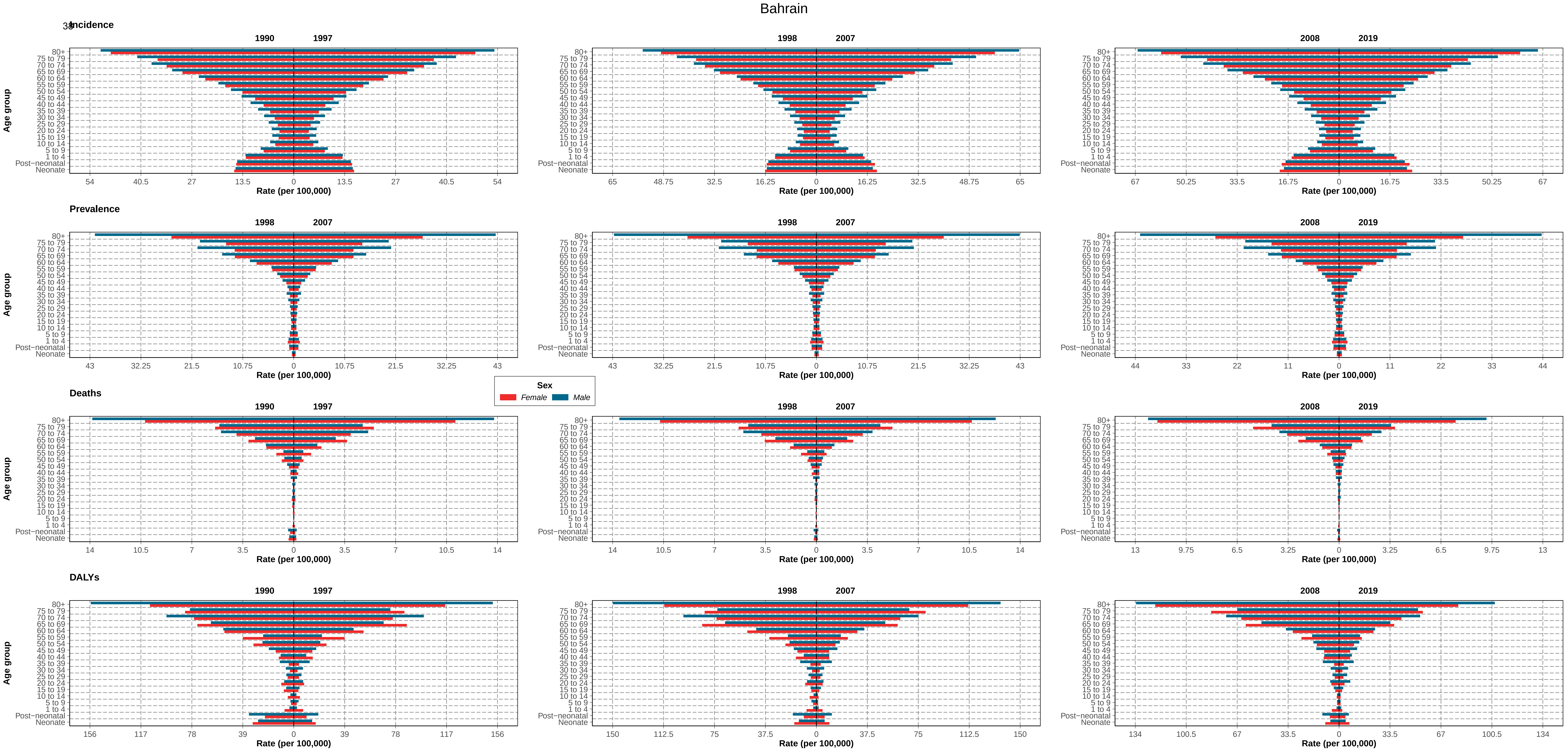
Bahrain
Incidence
Prevalence
Deaths
DALYs
1990
1997
1998
2007
2008
2019
Age group
Rate (per 100,000)
Sex
Female
Male
80+
75 to 79
70 to 74
65 to 69
60 to 64
55 to 59
50 to 54
45 to 49
40 to 44
35 to 39
30 to 34
25 to 29
20 to 24
15 to 19
10 to 14
5 to 9
1 to 4
Post−neonatal
Neonate

Egypt

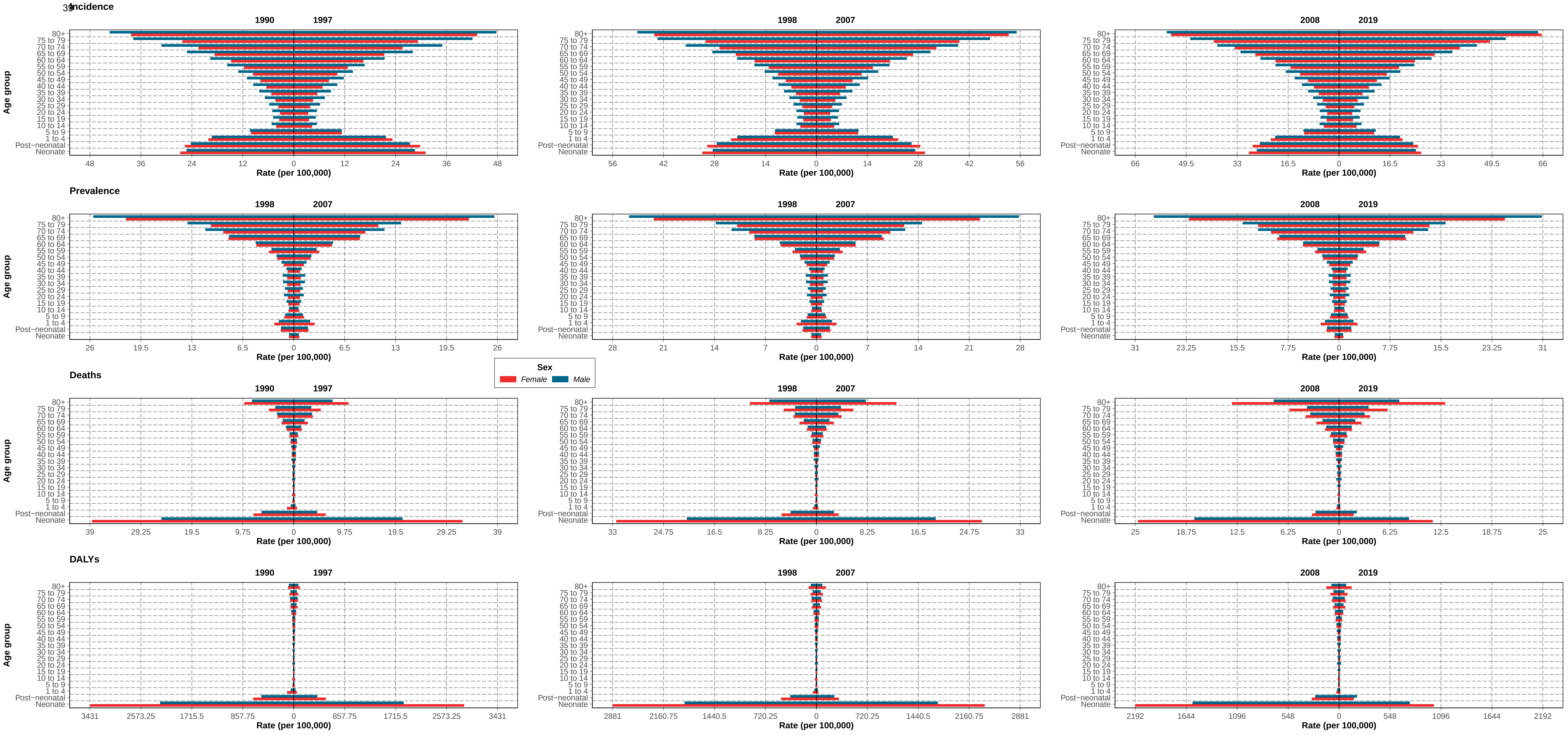
Incidence
Prevalence
Deaths
DALYs
1990
1997
1998
2007
2008
2019
Age group
Rate (per 100,000)
80+
75 to 79
70 to 74
65 to 69
60 to 64
55 to 59
50 to 54
45 to 49
40 to 44
35 to 39
30 to 34
25 to 29
20 to 24
15 to 19
10 to 14
5 to 9
1 to 4
Post−neonatal
Neonate
Sex
Female
Male

Iran (Islamic Republic of)

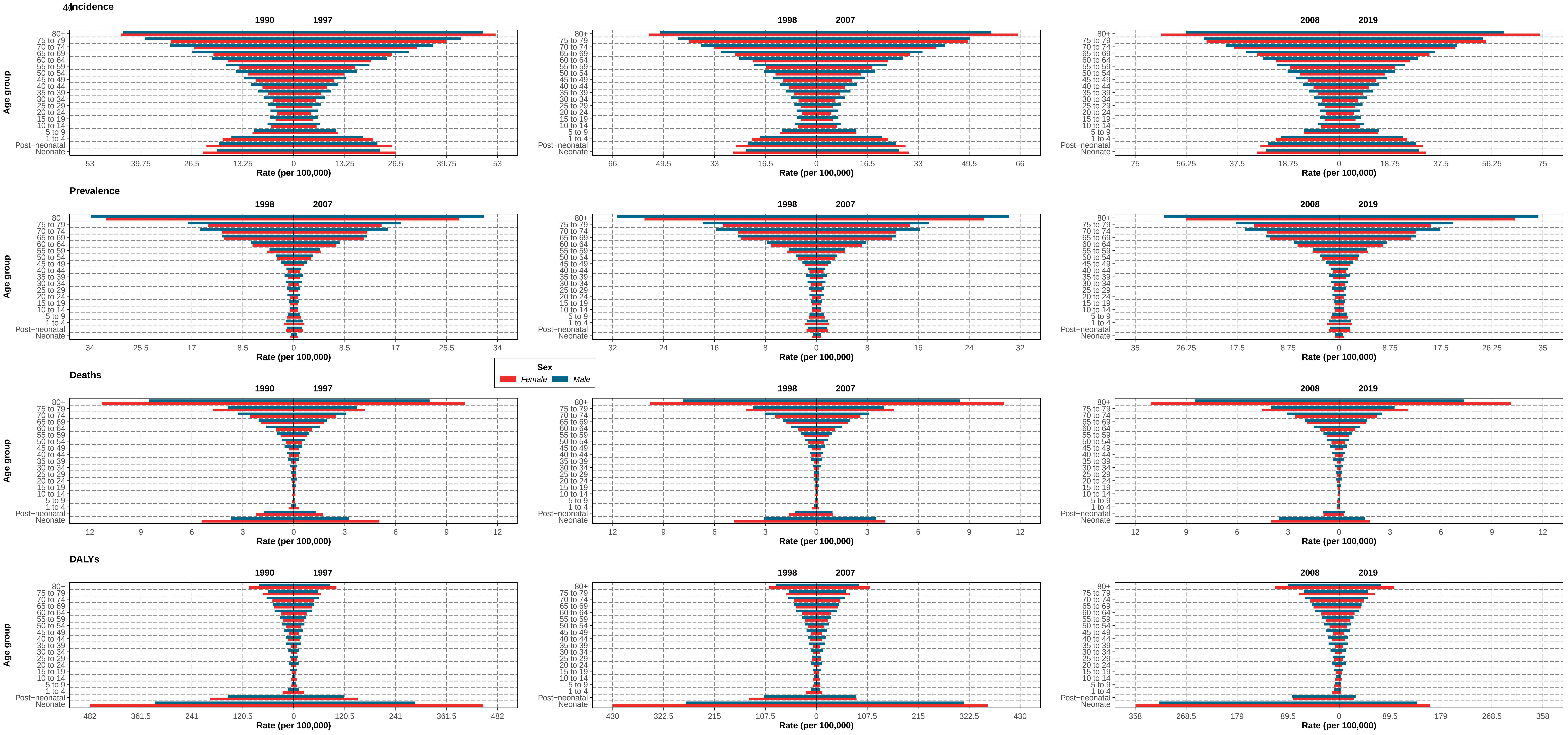

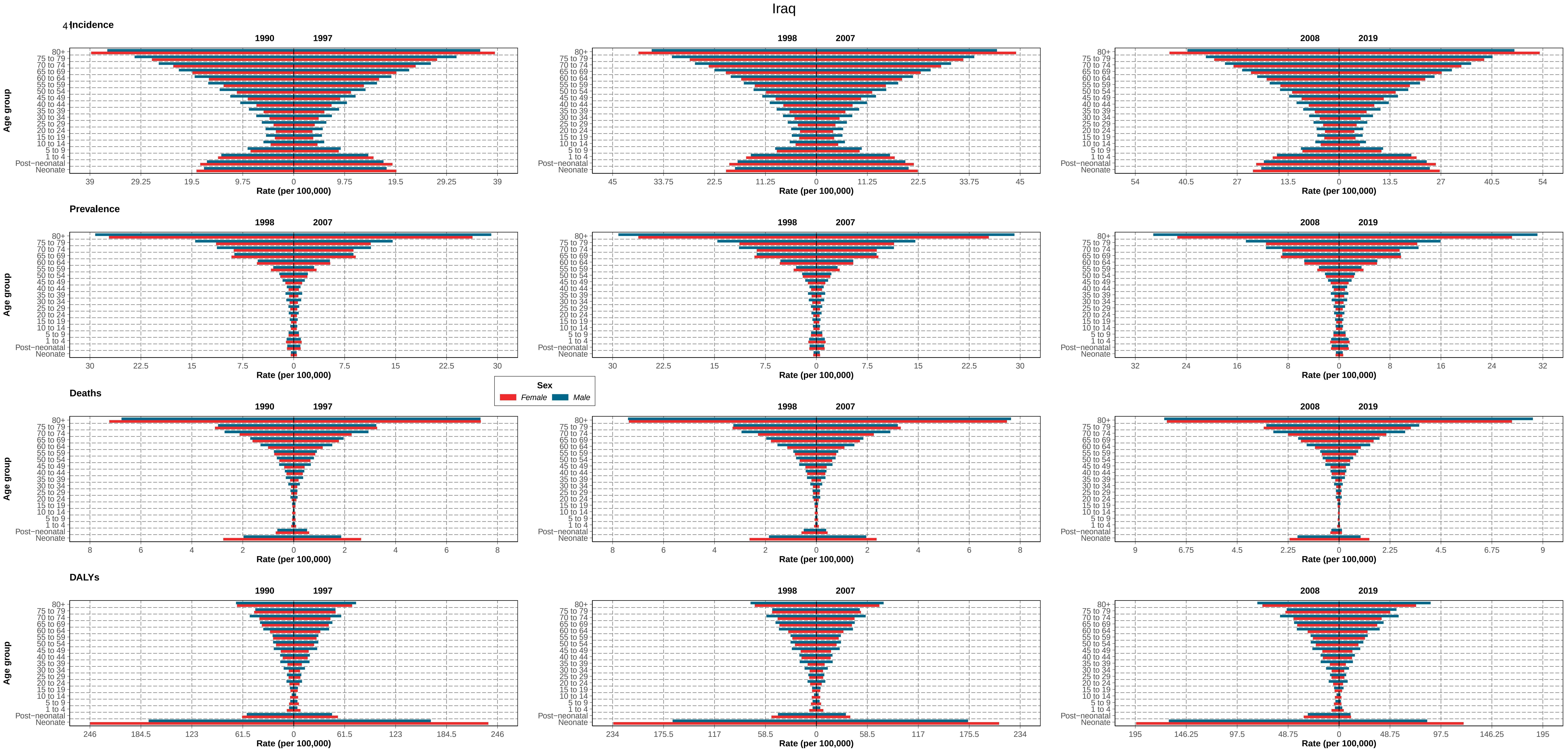
4
Iraq
Incidence
1990
1997
1998
2007
2008
2019
Prevalence
Deaths
DALYs
Age group
Rate (per 100,000)
Sex
Female
Male
80+
75 to 79
70 to 74
65 to 69
60 to 64
55 to 59
50 to 54
45 to 49
40 to 44
35 to 39
30 to 34
25 to 29
20 to 24
15 to 19
10 to 14
5 to 9
1 to 4
Post−neonatal
Neonate

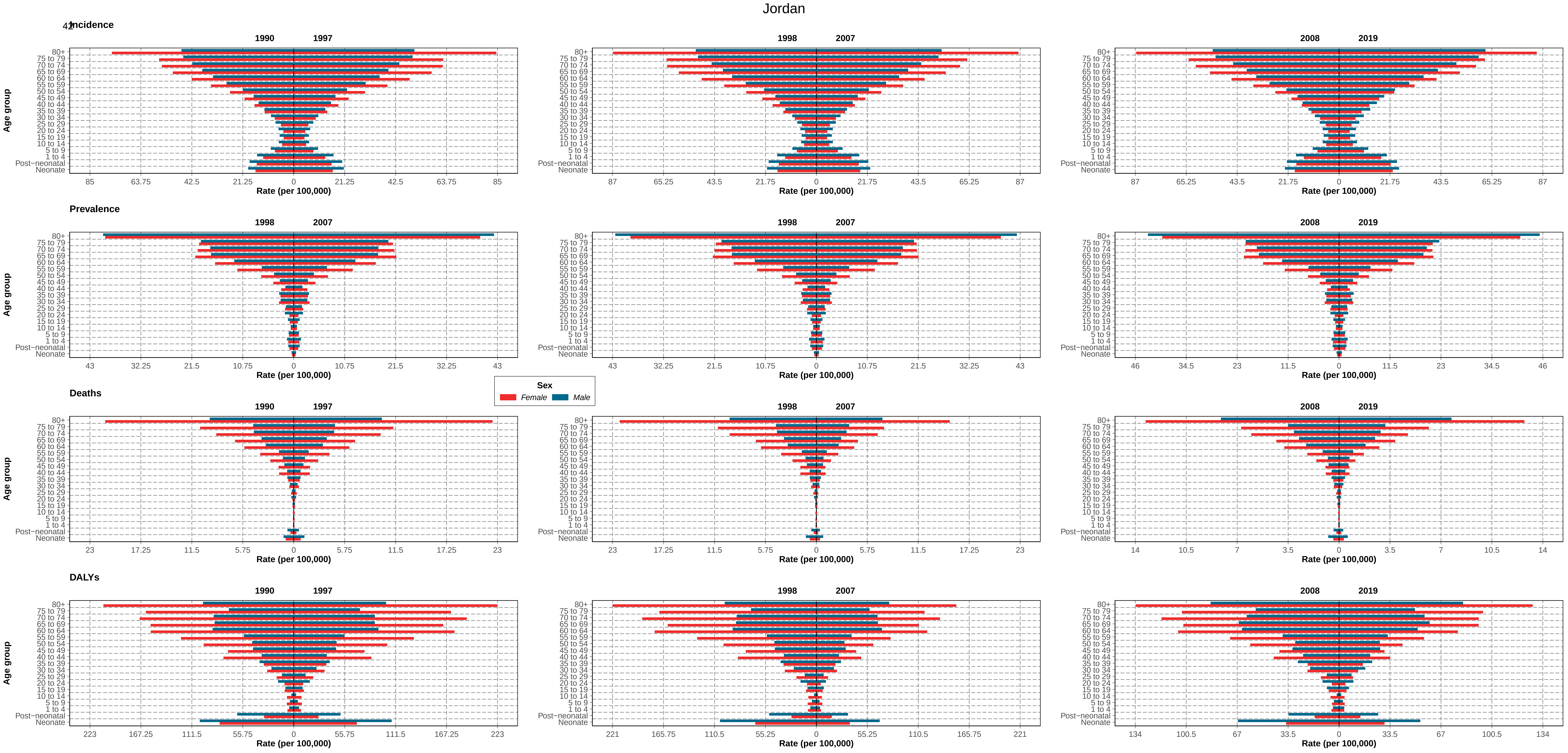
Jordan
Incidence
Prevalence
Deaths
DALYs
1990
1997
1998
2007
2008
2019
Age group
Rate (per 100,000)
Sex
Female
Male
80+
75 to 79
70 to 74
65 to 69
60 to 64
55 to 59
50 to 54
45 to 49
40 to 44
35 to 39
30 to 34
25 to 29
20 to 24
15 to 19
10 to 14
5 to 9
1 to 4
Post−neonatal
Neonate

Kuwait

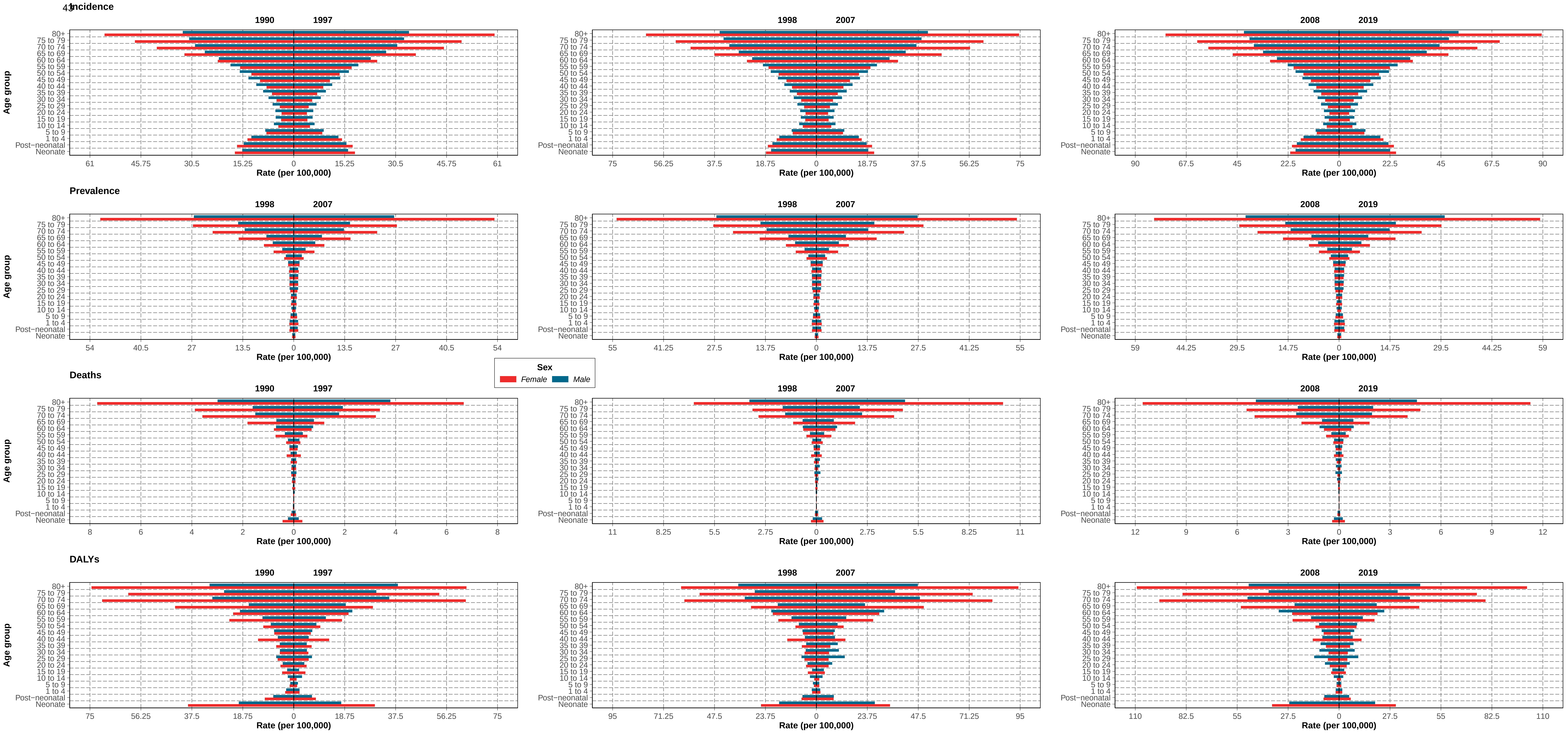
Incidence
1990
1997
1998
2007
2008
2019
Prevalence
Deaths
DALYs
Sex
Female
Male
Age group
Rate (per 100,000)
80+
75 to 79
70 to 74
65 to 69
60 to 64
55 to 59
50 to 54
45 to 49
40 to 44
35 to 39
30 to 34
25 to 29
20 to 24
15 to 19
10 to 14
5 to 9
1 to 4
Post−neonatal
Neonate

# Lebanon

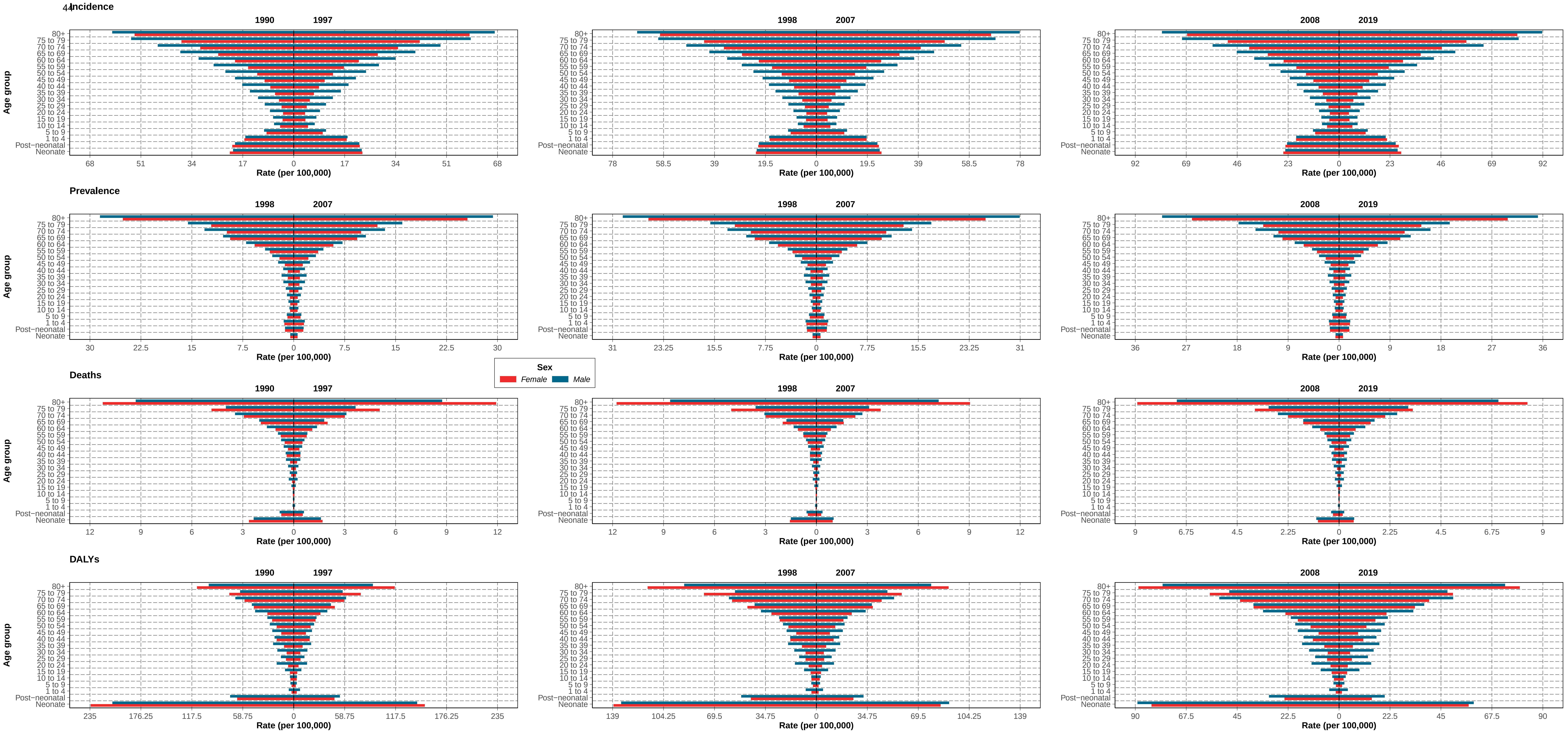
Incidence
Prevalence
Deaths
DALYs
1990
1997
1998
2007
2008
2019
Age group
Rate (per 100,000)
80+
75 to 79
70 to 74
65 to 69
60 to 64
55 to 59
50 to 54
45 to 49
40 to 44
35 to 39
30 to 34
25 to 29
20 to 24
15 to 19
10 to 14
5 to 9
1 to 4
Post−neonatal
Neonate
Sex
Female
Male

Libya

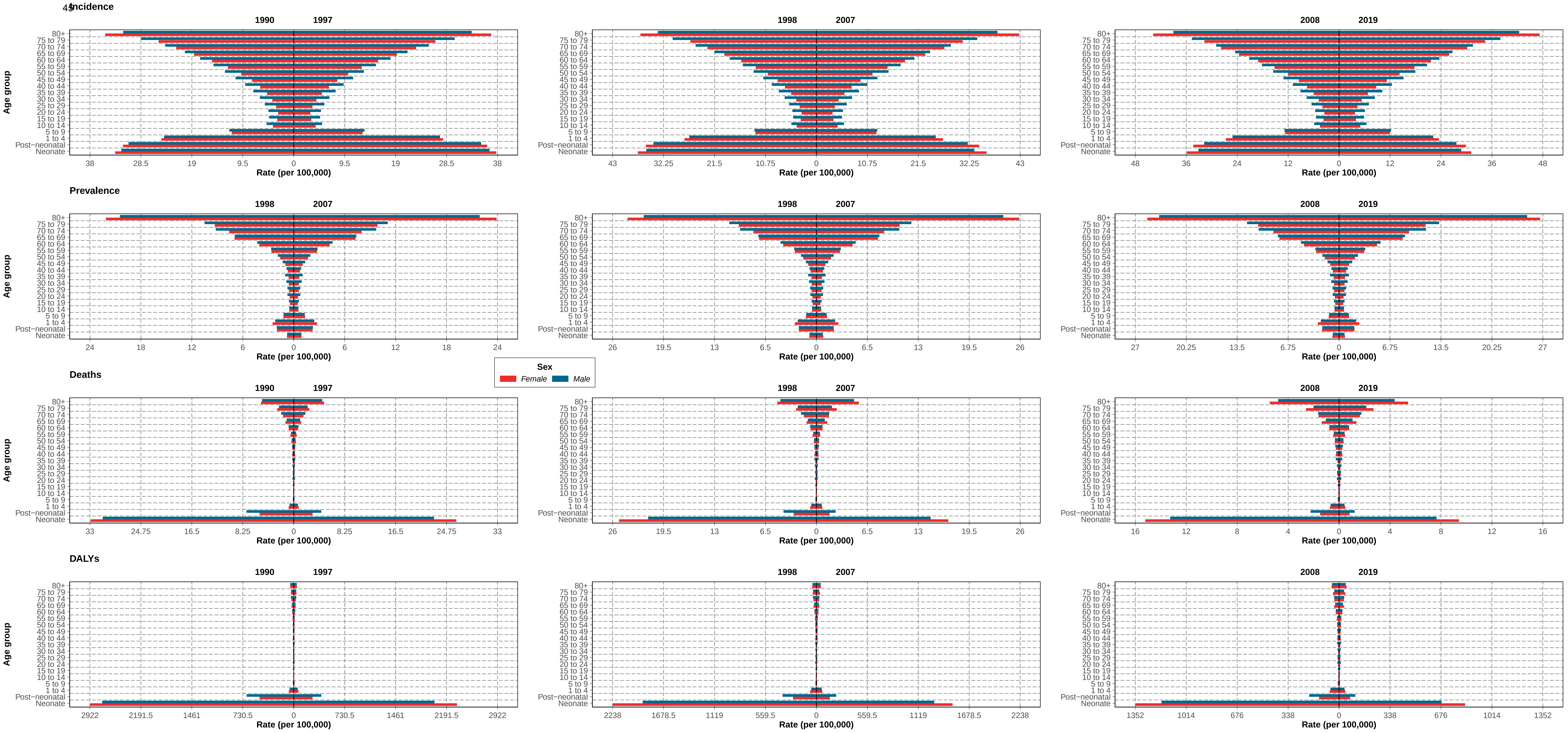
Incidence
1990
1997
1998
2007
2008
2019
80+
75 to 79
70 to 74
65 to 69
60 to 64
55 to 59
50 to 54
45 to 49
40 to 44
35 to 39
30 to 34
25 to 29
20 to 24
15 to 19
10 to 14
5 to 9
1 to 4
Post−neonatal
Neonate
Age group
38 28.5 19 9.5 0 9.5 19 28.5 38
43 32.25 21.5 10.75 0 10.75 21.5 32.25 43
48 36 24 12 0 12 24 36 48
Rate (per 100,000)
Prevalence
1998
2007
24 18 12 6 0 6 12 18 24
26 19.5 13 6.5 0 6.5 13 19.5 26
27 20.25 13.5 6.75 0 6.75 13.5 20.25 27
Sex
Female
Male
Deaths
33 24.75 16.5 8.25 0 8.25 16.5 24.75 33
26 19.5 13 6.5 0 6.5 13 19.5 26
16 12 8 4 0 4 8 12 16
DALYs
2922 2191.5 1461 730.5 0 730.5 1461 2191.5 2922
2238 1678.5 1119 559.5 0 559.5 1119 1678.5 2238
1352 1014 676 338 0 338 676 1014 1352

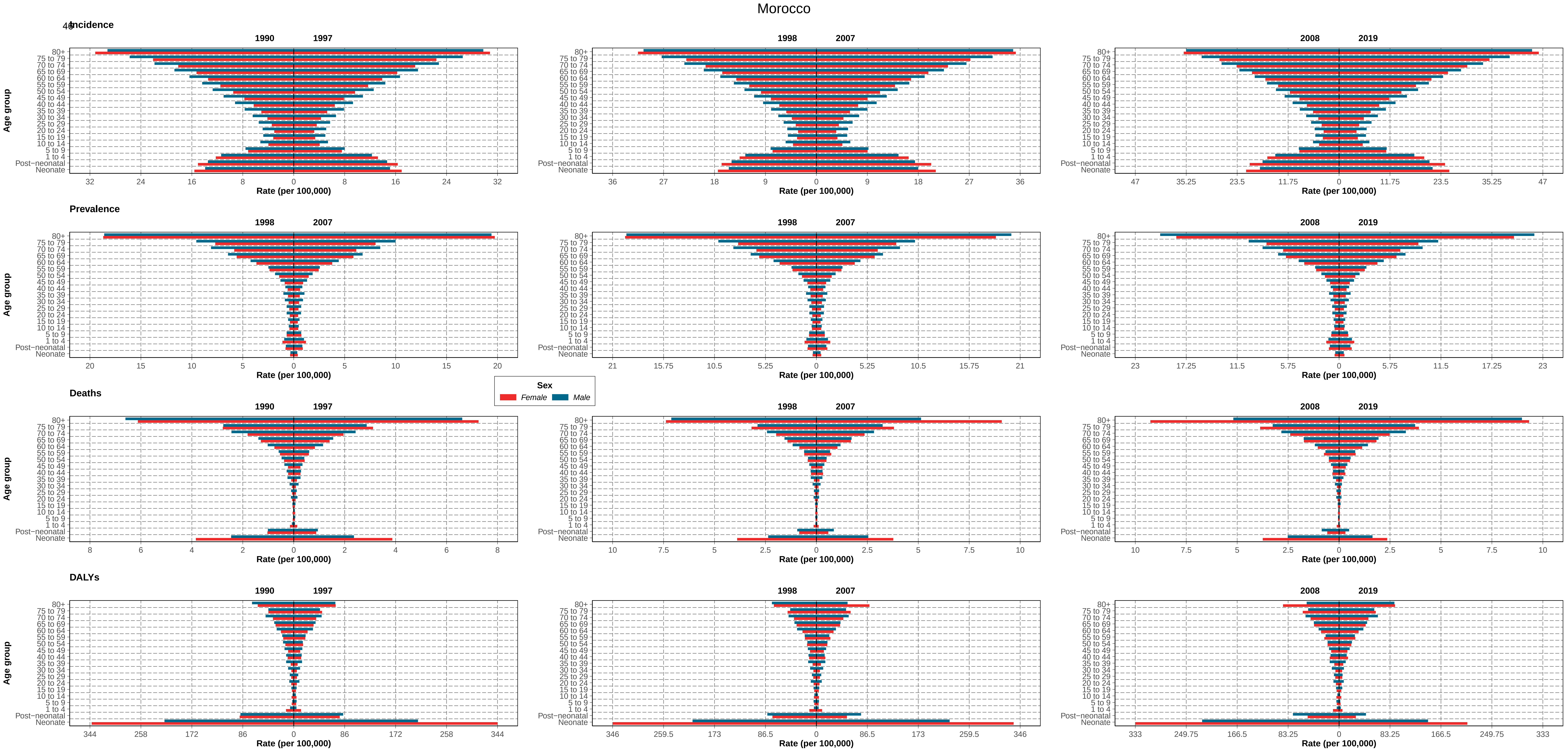
Morocco
40
Incidence
Prevalence
Deaths
DALYs
1990
1997
1998
2007
2008
2019
Age group
80+
75 to 79
70 to 74
65 to 69
60 to 64
55 to 59
50 to 54
45 to 49
40 to 44
35 to 39
30 to 34
25 to 29
20 to 24
15 to 19
10 to 14
5 to 9
1 to 4
Post−neonatal
Neonate
Rate (per 100,000)
Sex
Female
Male

Oman

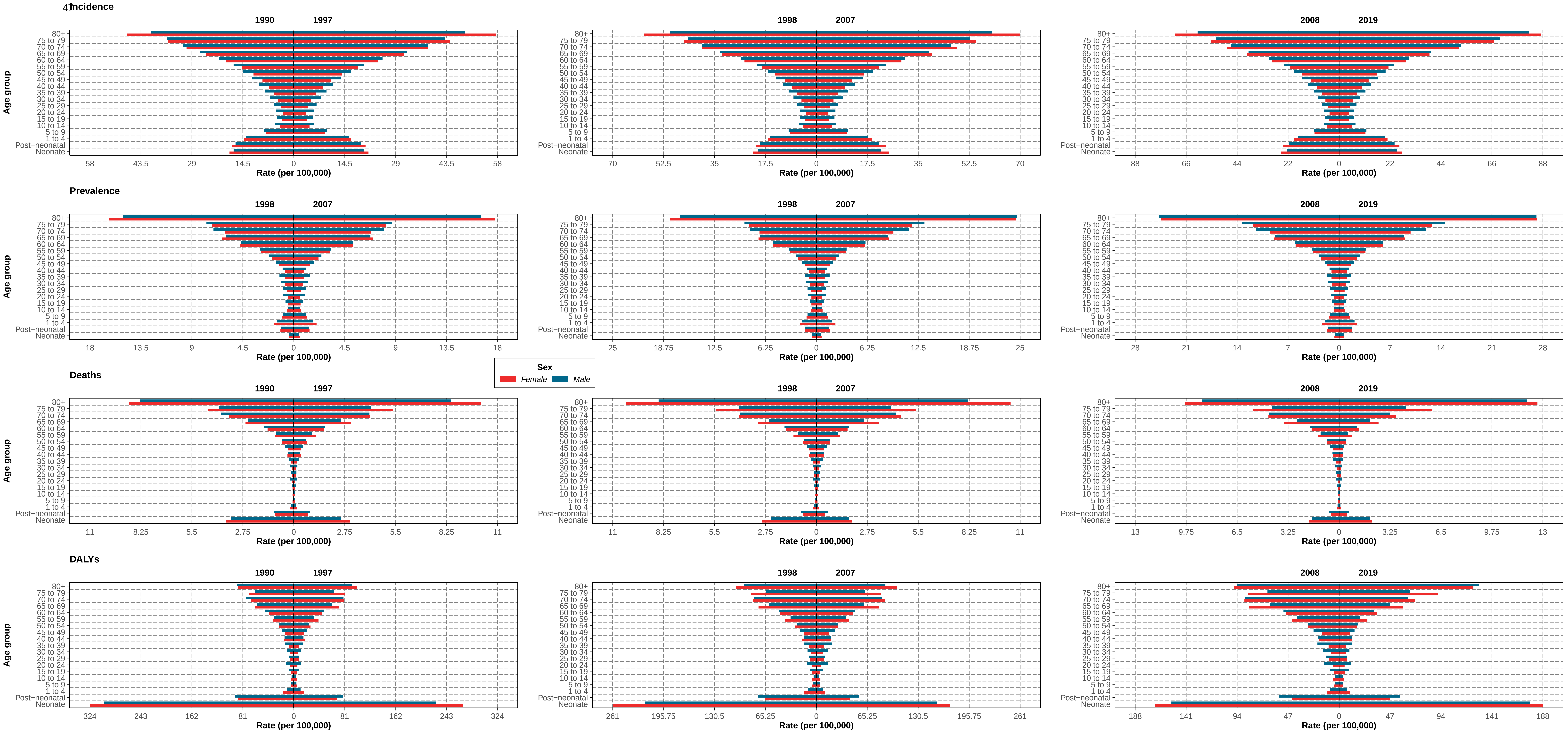
Incidence
Prevalence
Deaths
DALYs
1990
1997
1998
2007
2008
2019
Age group
Rate (per 100,000)
80+
75 to 79
70 to 74
65 to 69
60 to 64
55 to 59
50 to 54
45 to 49
40 to 44
35 to 39
30 to 34
25 to 29
20 to 24
15 to 19
10 to 14
5 to 9
1 to 4
Post−neonatal
Neonate
Sex
Female
Male

# Palestine

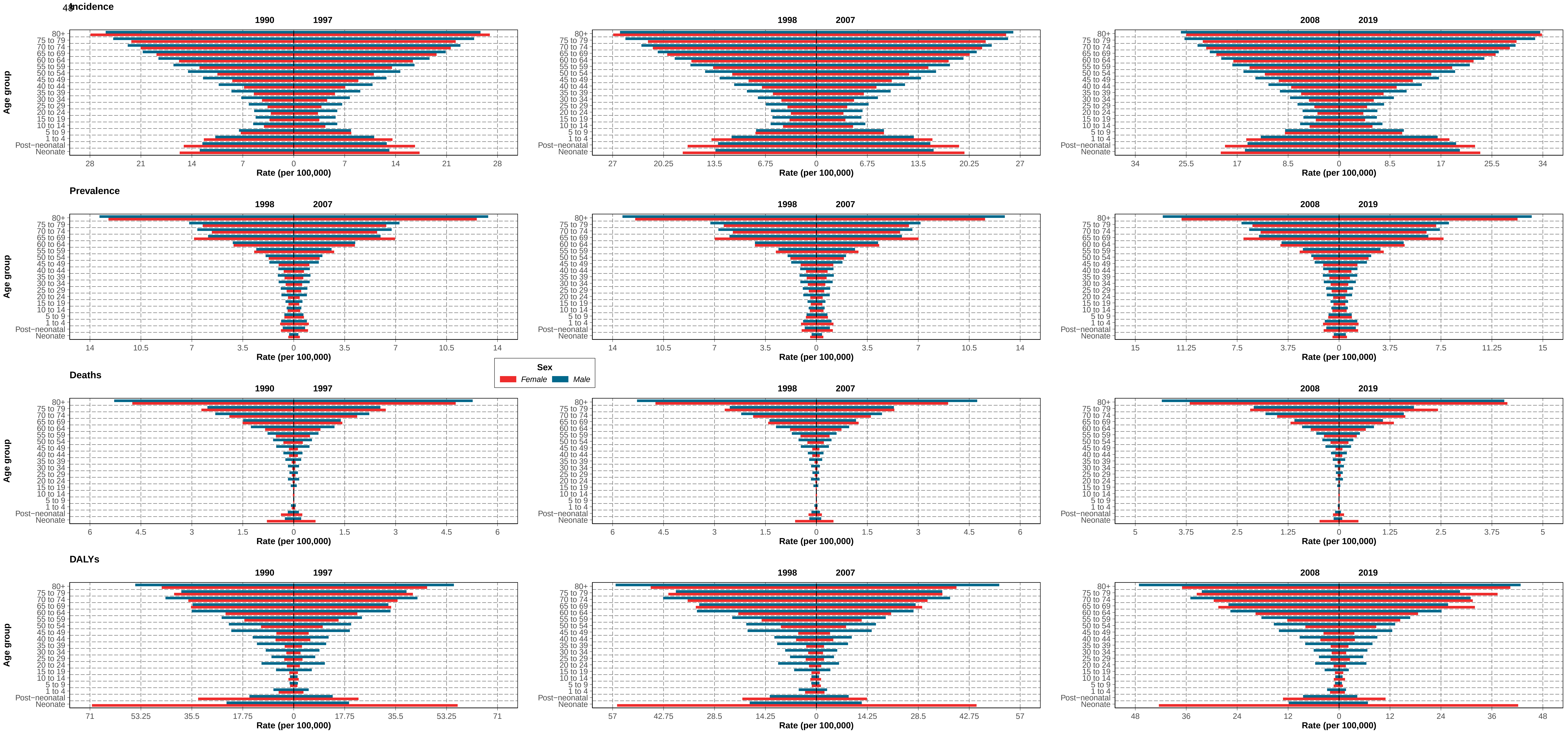
Incidence
Prevalence
Deaths
DALYs
1990
1997
1998
2007
2008
2019
Age group
80+
75 to 79
70 to 74
65 to 69
60 to 64
55 to 59
50 to 54
45 to 49
40 to 44
35 to 39
30 to 34
25 to 29
20 to 24
15 to 19
10 to 14
5 to 9
1 to 4
Post−neonatal
Neonate
Rate (per 100,000)
28 21 14 7 0 7 14 21 28
27 20.25 13.5 6.75 0 6.75 13.5 20.25 27
34 25.5 17 8.5 0 8.5 17 25.5 34
14 10.5 7 3.5 0 3.5 7 10.5 14
15 11.25 7.5 3.75 0 3.75 7.5 11.25 15
6 4.5 3 1.5 0 1.5 3 4.5 6
5 3.75 2.5 1.25 0 1.25 2.5 3.75 5
71 53.25 35.5 17.75 0 17.75 35.5 53.25 71
57 42.75 28.5 14.25 0 14.25 28.5 42.75 57
48 36 24 12 0 12 24 36 48
Sex
Female
Male

# Qatar

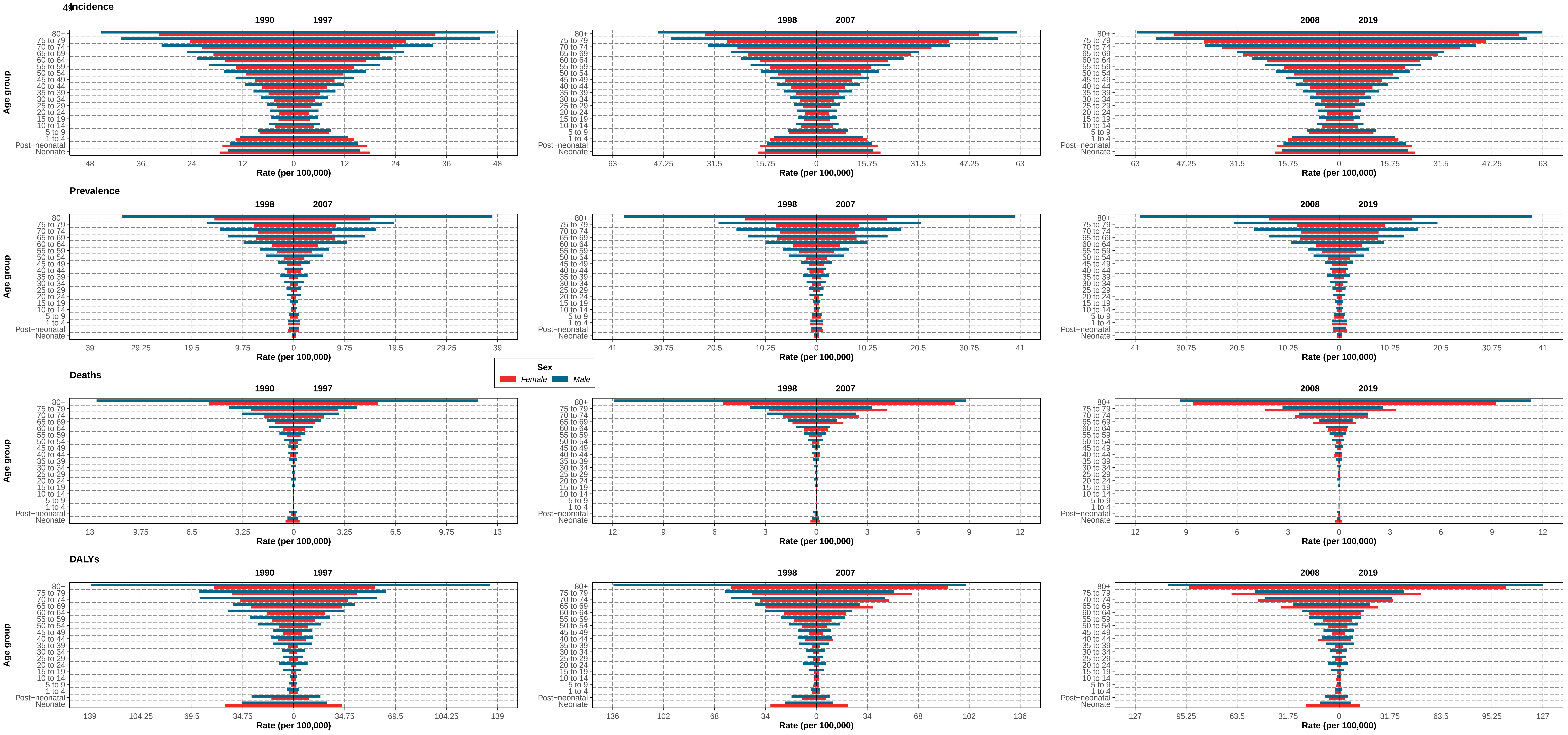
Incidence
Prevalence
Deaths
DALYs
1990
1997
1998
2007
2008
2019
Age group
Rate (per 100,000)
80+
75 to 79
70 to 74
65 to 69
60 to 64
55 to 59
50 to 54
45 to 49
40 to 44
35 to 39
30 to 34
25 to 29
20 to 24
15 to 19
10 to 14
5 to 9
1 to 4
Post−neonatal
Neonate
48 36 24 12 0 12 24 36 48
63 47.25 31.5 15.75 0 15.75 31.5 47.25 63
39 29.25 19.5 9.75 0 9.75 19.5 29.25 39
41 30.75 20.5 10.25 0 10.25 20.5 30.75 41
13 9.75 6.5 3.25 0 3.25 6.5 9.75 13
12 9 6 3 0 3 6 9 12
139 104.25 69.5 34.75 0 34.75 69.5 104.25 139
136 102 68 34 0 34 68 102 136
127 95.25 63.5 31.75 0 31.75 63.5 95.25 127
Sex
Female
Male

# Saudi Arabia

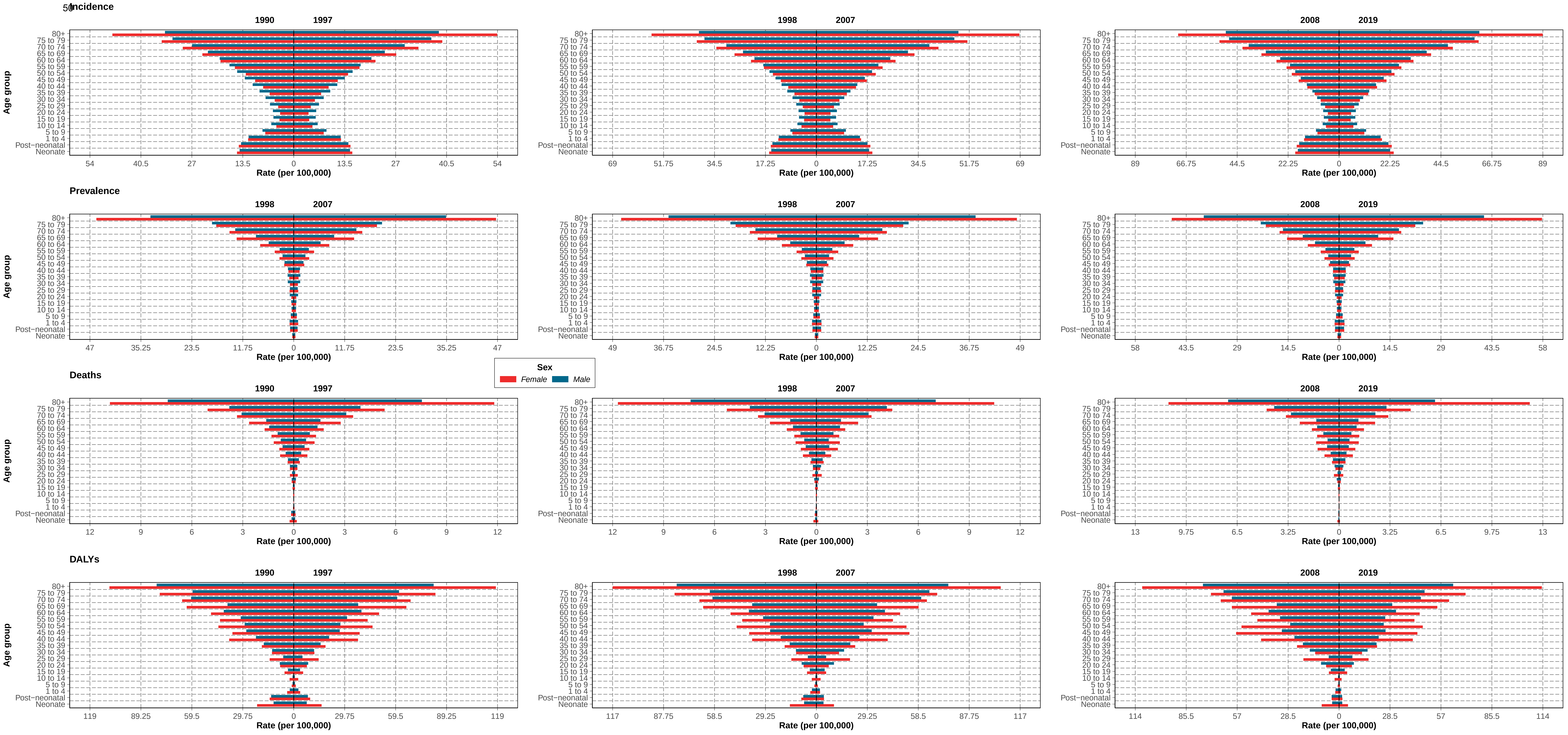

# Sudan

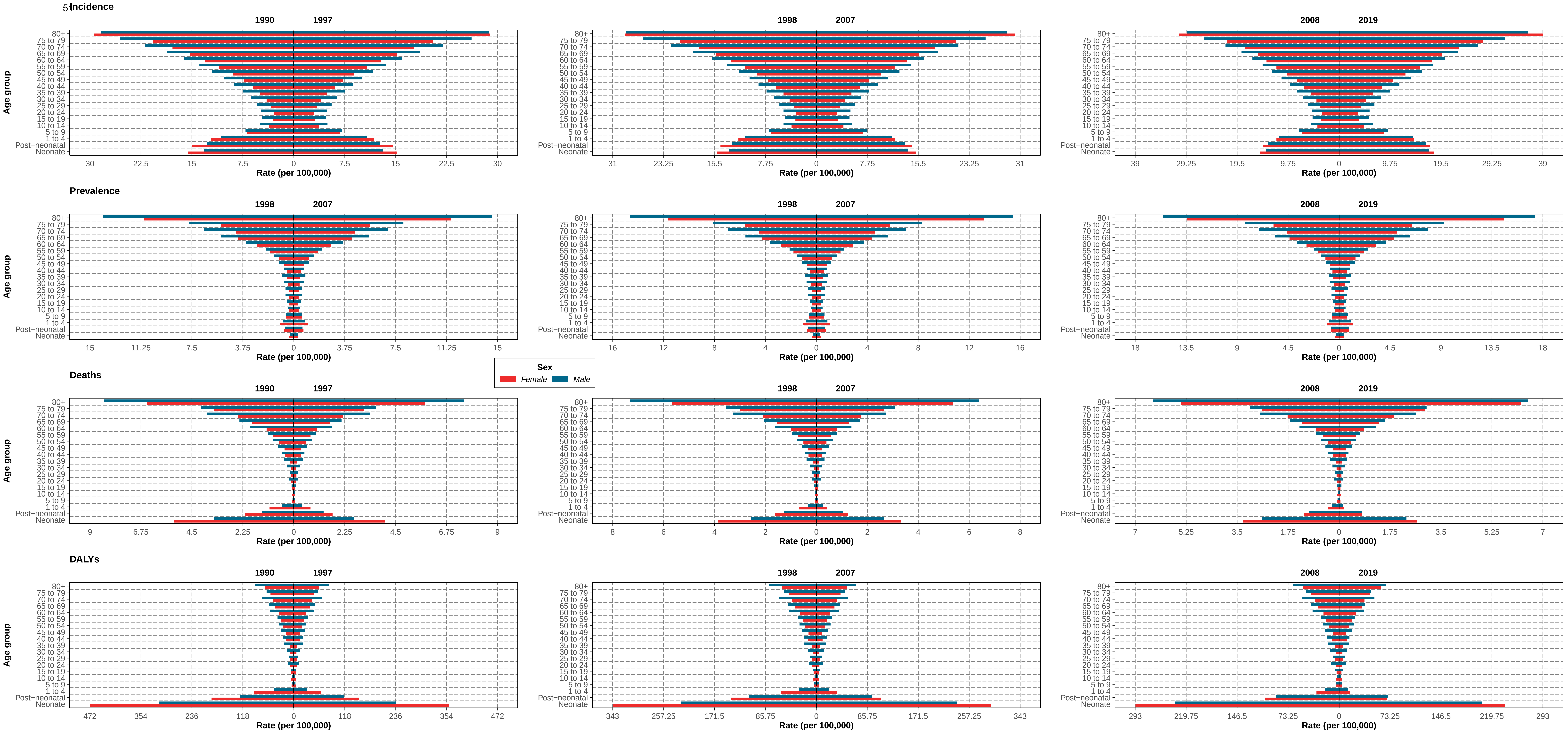
Incidence
Prevalence
Deaths
DALYs
1990
1997
1998
2007
2008
2019
Age group
80+
75 to 79
70 to 74
65 to 69
60 to 64
55 to 59
50 to 54
45 to 49
40 to 44
35 to 39
30 to 34
25 to 29
20 to 24
15 to 19
10 to 14
5 to 9
1 to 4
Post−neonatal
Neonate
Rate (per 100,000)
30 22.5 15 7.5 0 7.5 15 22.5 30
31 23.25 15.5 7.75 0 7.75 15.5 23.25 31
39 29.25 19.5 9.75 0 9.75 19.5 29.25 39
15 11.25 7.5 3.75 0 3.75 7.5 11.25 15
16 12 8 4 0 4 8 12 16
18 13.5 9 4.5 0 4.5 9 13.5 18
9 6.75 4.5 2.25 0 2.25 4.5 6.75 9
8 6 4 2 0 2 4 6 8
7 5.25 3.5 1.75 0 1.75 3.5 5.25 7
472 354 236 118 0 118 236 354 472
343 257.25 171.5 85.75 0 85.75 171.5 257.25 343
293 219.75 146.5 73.25 0 73.25 146.5 219.75 293
Sex
Female
Male

# Syrian Arab Republic

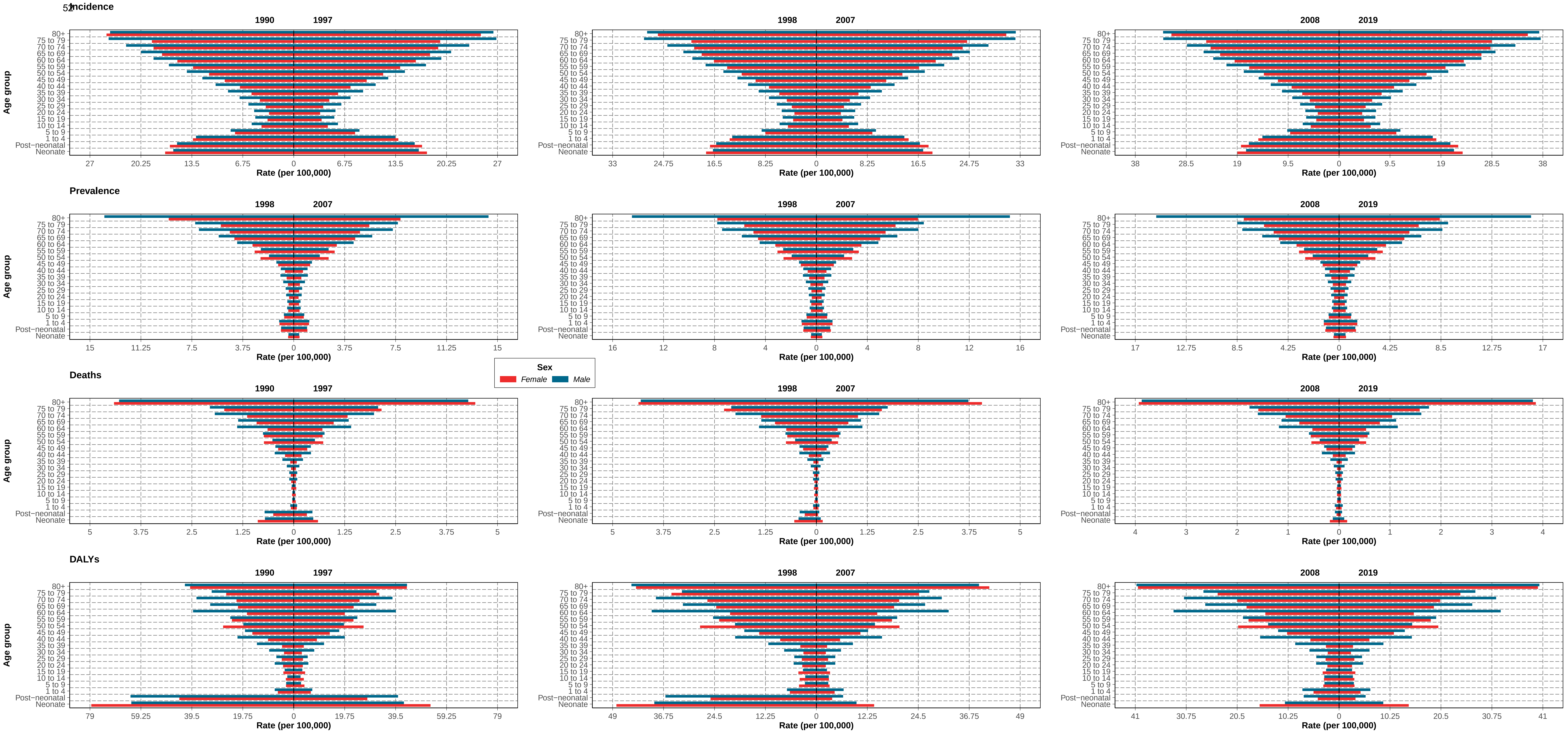

# Tunisia

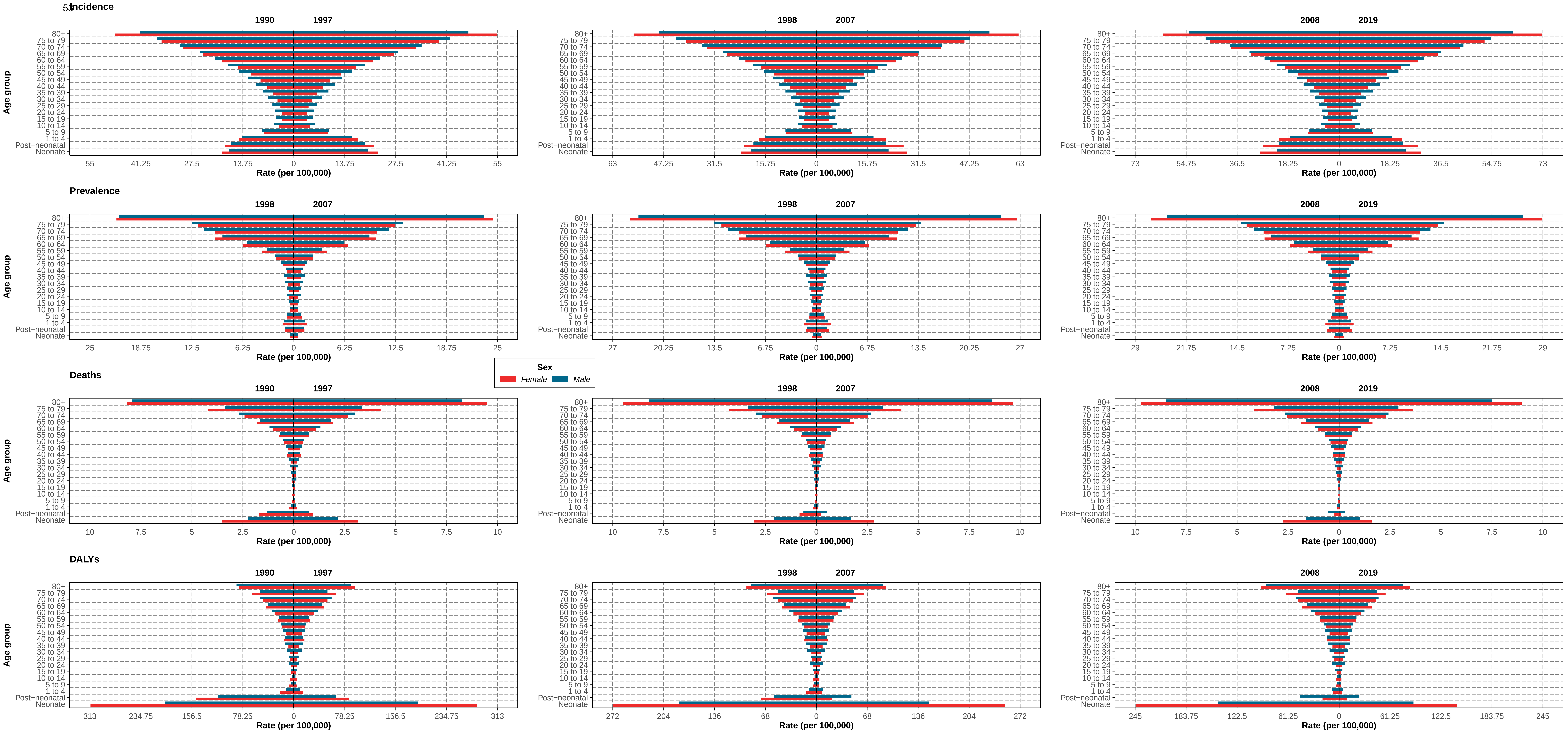

Incidence
Prevalence
Deaths
DALYs
1990
1997
1998
2007
2008
2019
Age group
Rate (per 100,000)
80+
75 to 79
70 to 74
65 to 69
60 to 64
55 to 59
50 to 54
45 to 49
40 to 44
35 to 39
30 to 34
25 to 29
20 to 24
15 to 19
10 to 14
5 to 9
1 to 4
Post−neonatal
Neonate
55 41.25 27.5 13.75 0 13.75 27.5 41.25 55
63 47.25 31.5 15.75 0 15.75 31.5 47.25 63
73 54.75 36.5 18.25 0 18.25 36.5 54.75 73
25 18.75 12.5 6.25 0 6.25 12.5 18.75 25
27 20.25 13.5 6.75 0 6.75 13.5 20.25 27
29 21.75 14.5 7.25 0 7.25 14.5 21.75 29
10 7.5 5 2.5 0 2.5 5 7.5 10
313 234.75 156.5 78.25 0 78.25 156.5 234.75 313
272 204 136 68 0 68 136 204 272
245 183.75 122.5 61.25 0 61.25 122.5 183.75 245
Sex
Female
Male

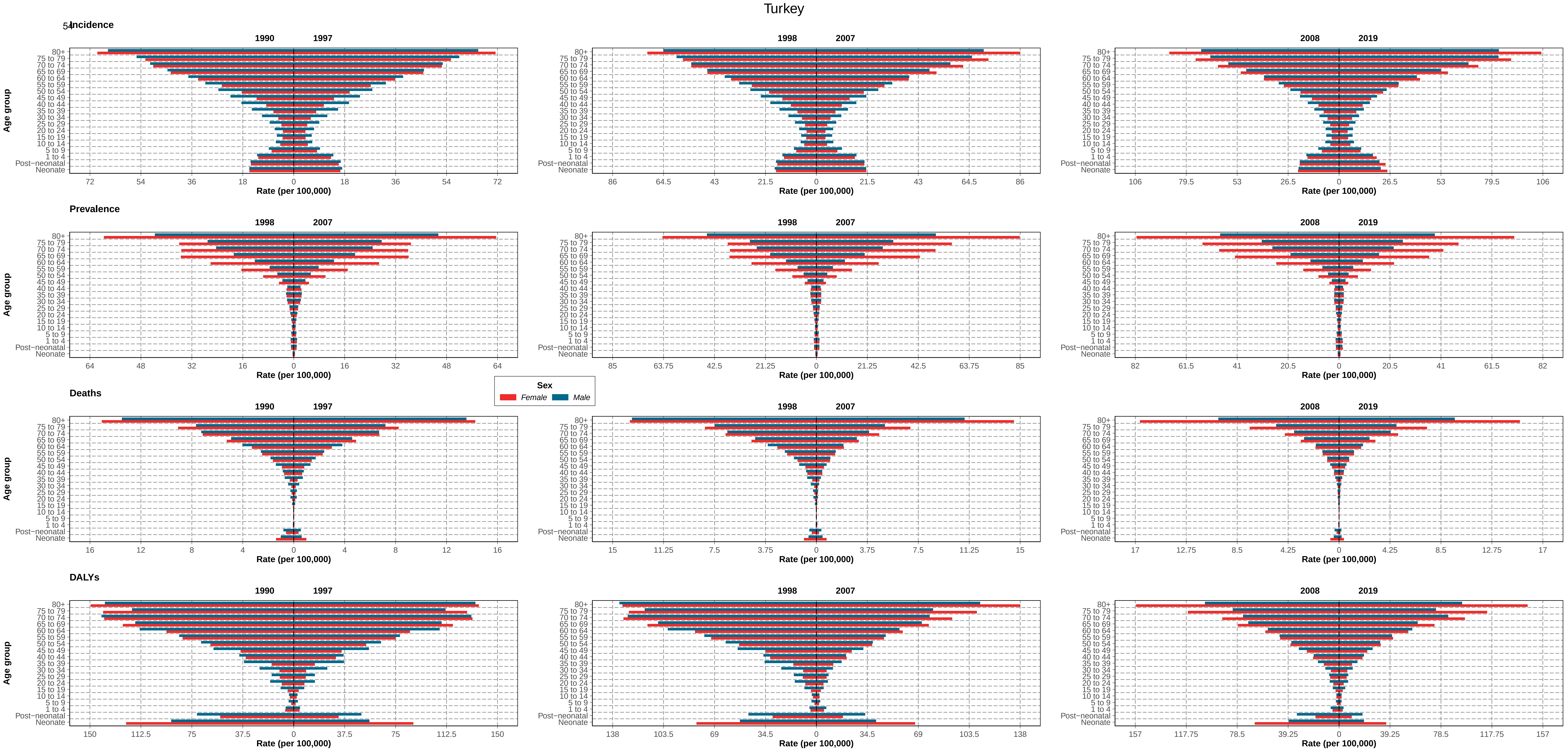
Turkey
Incidence
Prevalence
Deaths
DALYs
1990
1997
1998
2007
2008
2019
Age group
Rate (per 100,000)
80+
75 to 79
70 to 74
65 to 69
60 to 64
55 to 59
50 to 54
45 to 49
40 to 44
35 to 39
30 to 34
25 to 29
20 to 24
15 to 19
10 to 14
5 to 9
1 to 4
Post−neonatal
Neonate
Sex
Female
Male

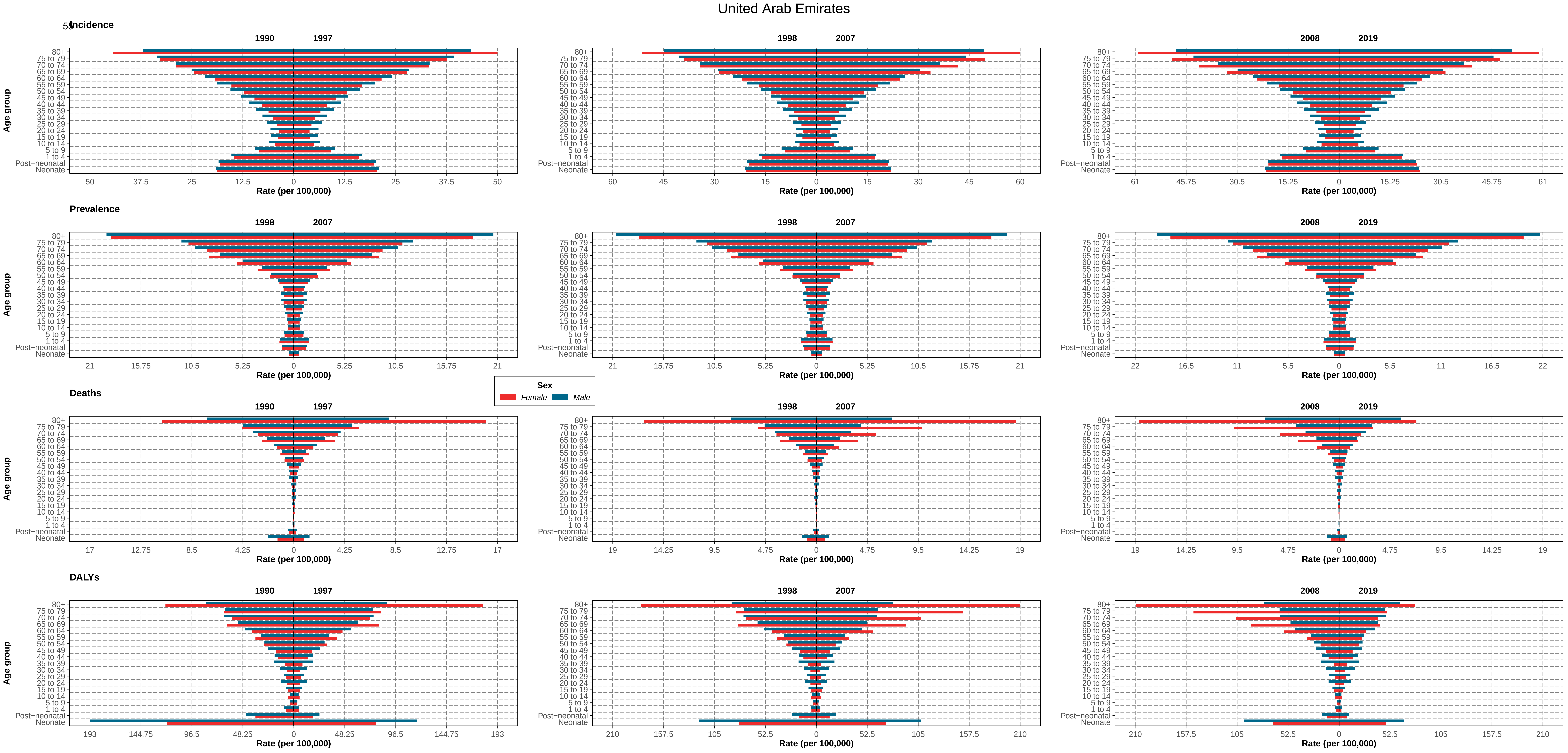

United Arab Emirates
59
Incidence
Prevalence
Deaths
DALYs
1990
1997
1998
2007
2008
2019
Age group
Rate (per 100,000)
80+
75 to 79
70 to 74
65 to 69
60 to 64
55 to 59
50 to 54
45 to 49
40 to 44
35 to 39
30 to 34
25 to 29
20 to 24
15 to 19
10 to 14
5 to 9
1 to 4
Post−neonatal
Neonate
Sex
Female
Male

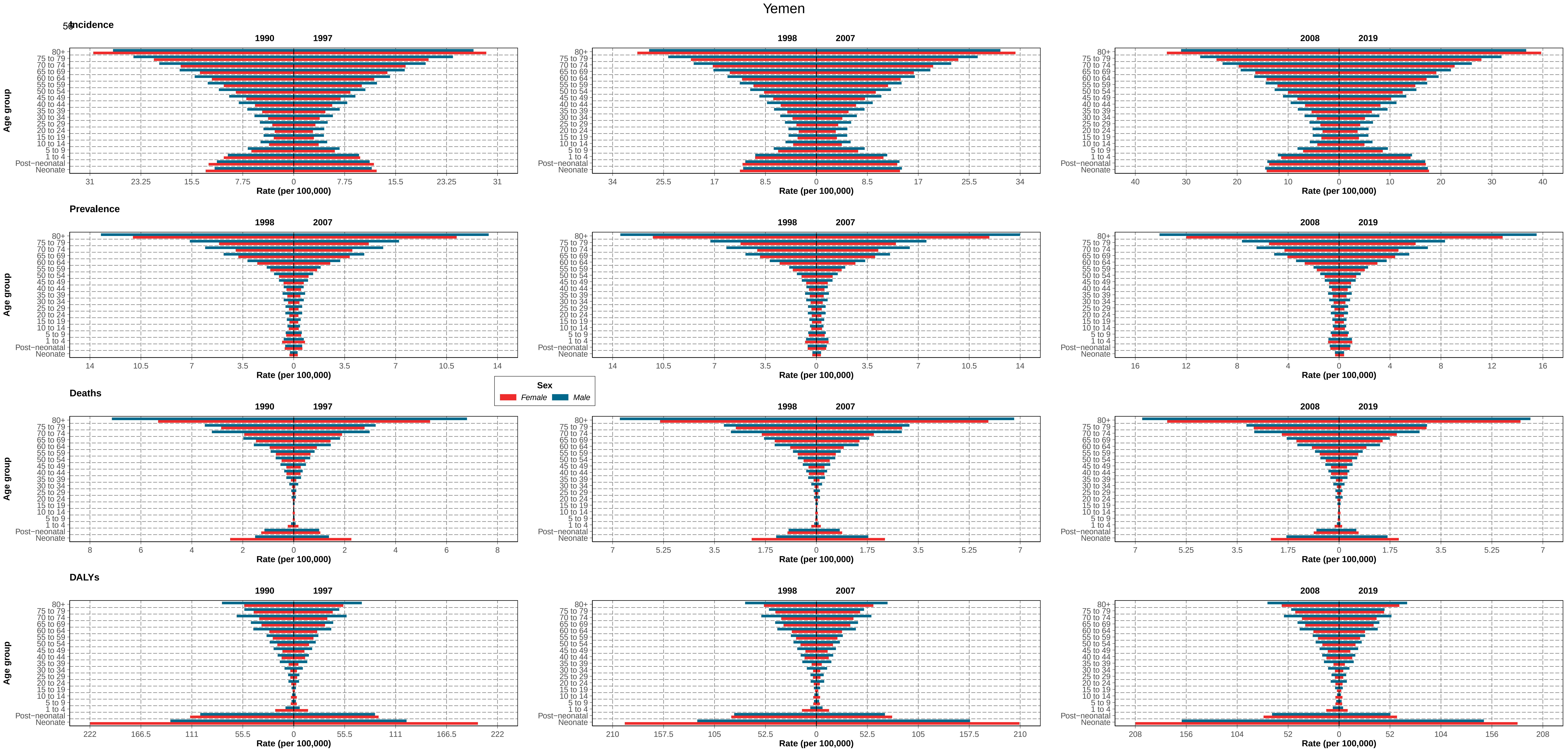
Yemen
Incidence
Prevalence
Deaths
DALYs
1990
1997
1998
2007
2008
2019
Age group
80+
75 to 79
70 to 74
65 to 69
60 to 64
55 to 59
50 to 54
45 to 49
40 to 44
35 to 39
30 to 34
25 to 29
20 to 24
15 to 19
10 to 14
5 to 9
1 to 4
Post−neonatal
Neonate
31 23.25 15.5 7.75 0 7.75 15.5 23.25 31
34 25.5 17 8.5 0 8.5 17 25.5 34
40 30 20 10 0 10 20 30 40
14 10.5 7 3.5 0 3.5 7 10.5 14
16 12 8 4 0 4 8 12 16
8 6 4 2 0 2 4 6 8
7 5.25 3.5 1.75 0 1.75 3.5 5.25 7
222 166.5 111 55.5 0 55.5 111 166.5 222
210 157.5 105 52.5 0 52.5 105 157.5 210
208 156 104 52 0 52 104 156 208
Rate (per 100,000)
Sex
Female
Male

**S19Fig.**

**Rates (per 100,000) of incidence, prevalence, deaths, and DALYs of infective endocarditis in 21 countries of North Africa and the Middle East in 1990 compared with 2019 according to eighteen age groups and sex**

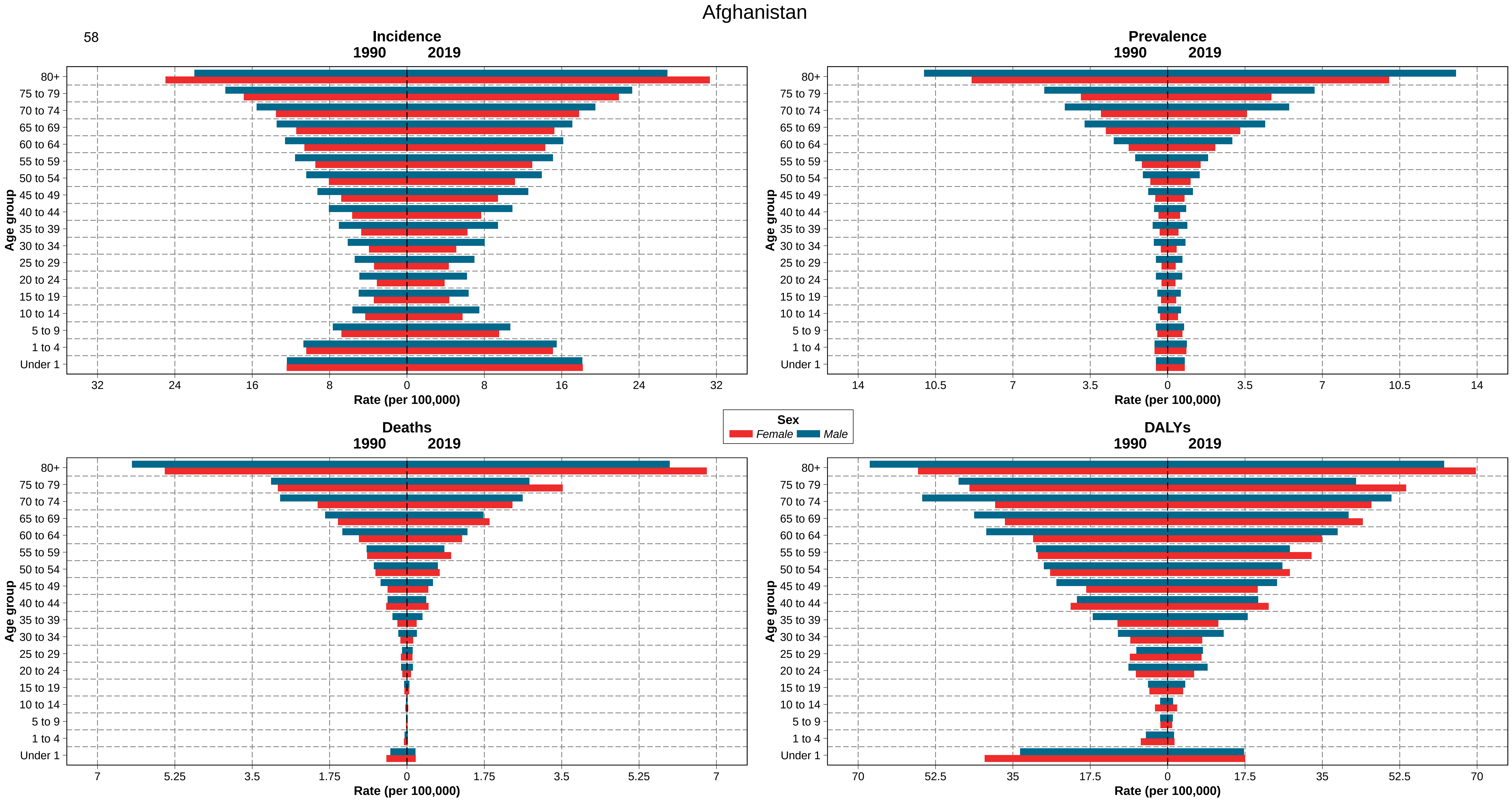
Afghanistan
Incidence
1990
2019
Prevalence
1990
2019
Deaths
1990
2019
DALYs
1990
2019
Age group
80+
75 to 79
70 to 74
65 to 69
60 to 64
55 to 59
50 to 54
45 to 49
40 to 44
35 to 39
30 to 34
25 to 29
20 to 24
15 to 19
10 to 14
5 to 9
1 to 4
Under 1
Rate (per 100,000)
Sex
Female
Male

# Algeria

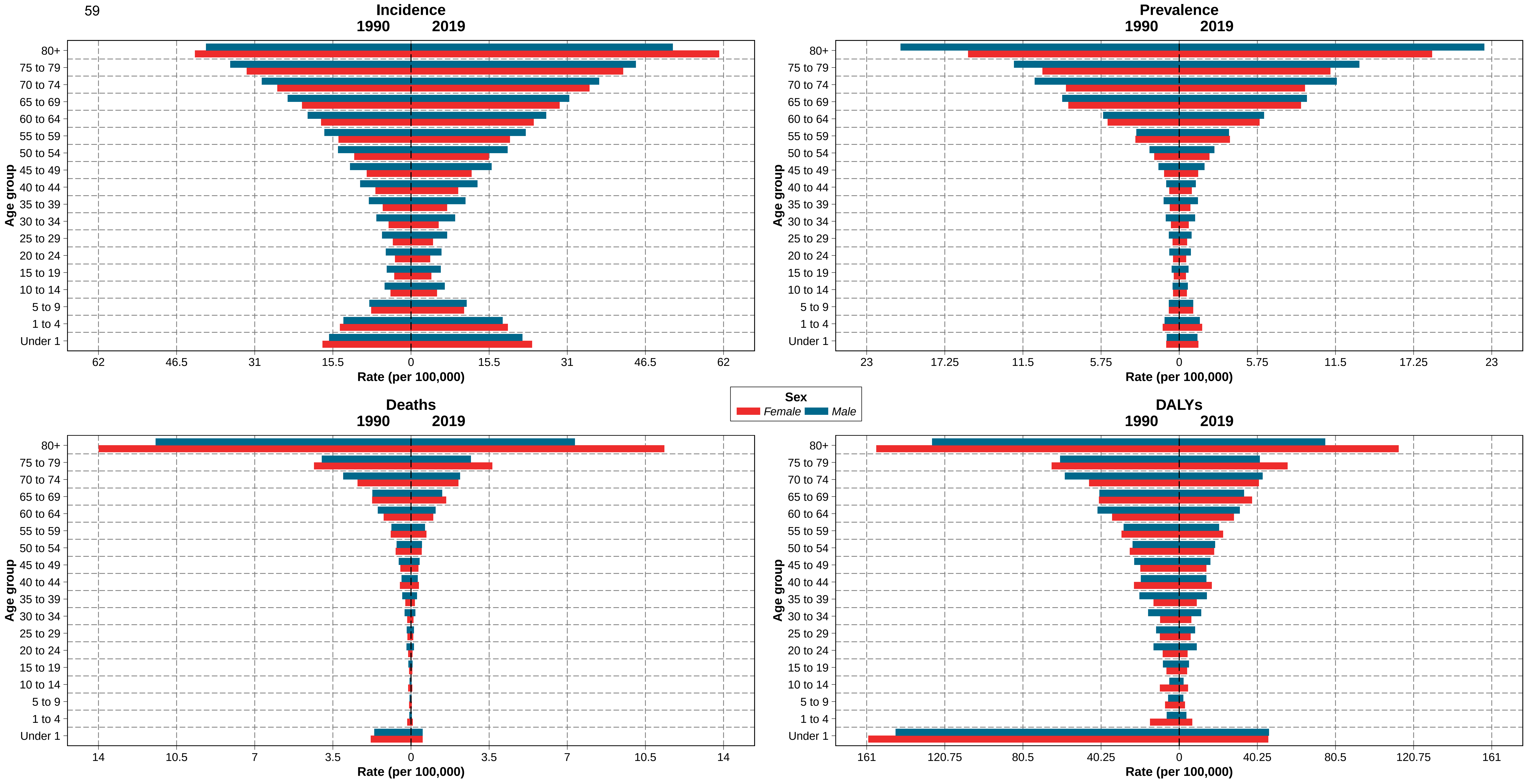
Incidence
1990
2019
Prevalence
1990
2019
Deaths
1990
2019
DALYs
1990
2019
Age group
80+
75 to 79
70 to 74
65 to 69
60 to 64
55 to 59
50 to 54
45 to 49
40 to 44
35 to 39
30 to 34
25 to 29
20 to 24
15 to 19
10 to 14
5 to 9
1 to 4
Under 1
62 46.5 31 15.5 0 15.5 31 46.5 62
23 17.25 11.5 5.75 0 5.75 11.5 17.25 23
14 10.5 7 3.5 0 3.5 7 10.5 14
161 120.75 80.5 40.25 0 40.25 80.5 120.75 161
Rate (per 100,000)
Sex
Female
Male

# Bahrain

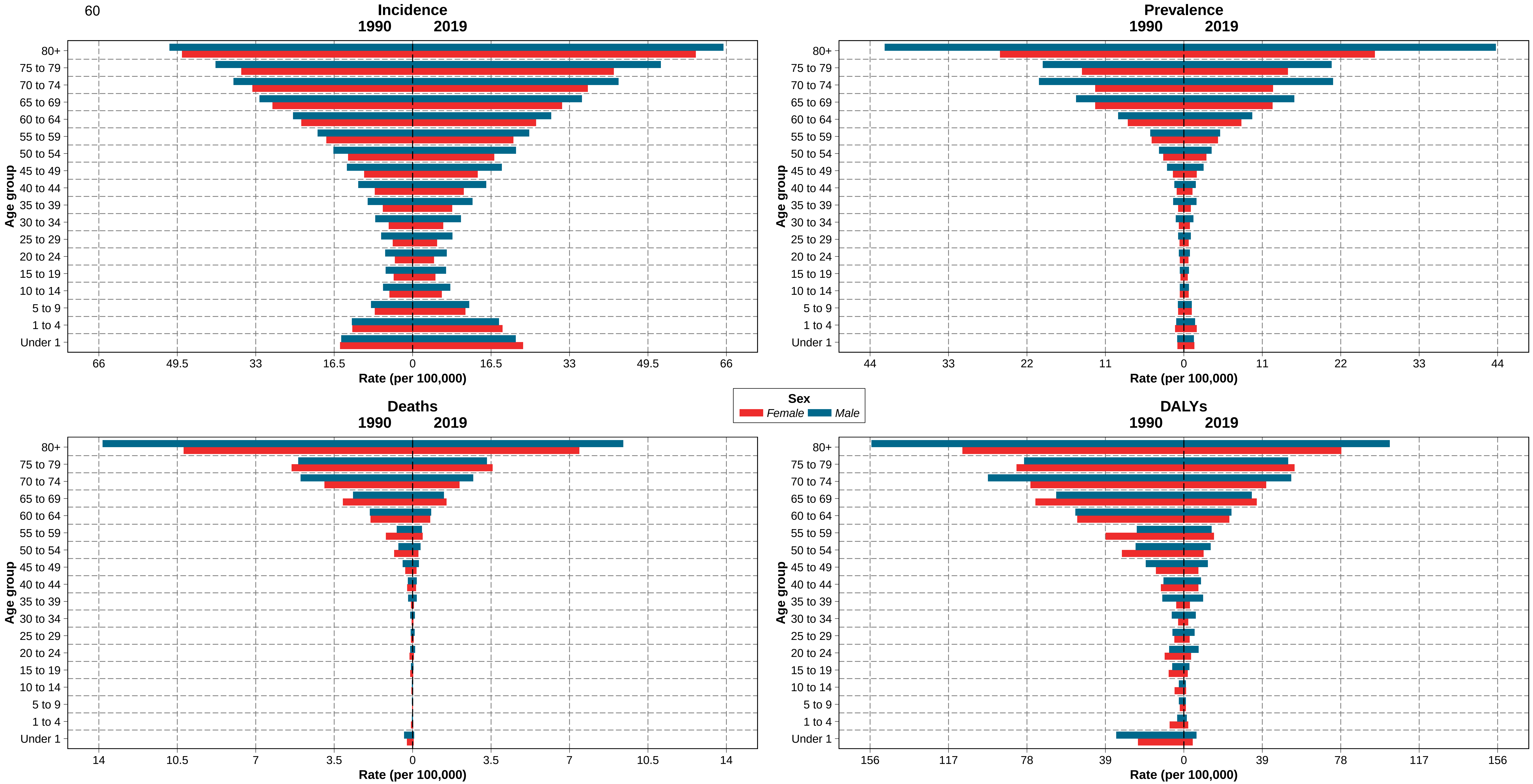
Incidence
1990
2019
Age group
80+
75 to 79
70 to 74
65 to 69
60 to 64
55 to 59
50 to 54
45 to 49
40 to 44
35 to 39
30 to 34
25 to 29
20 to 24
15 to 19
10 to 14
5 to 9
1 to 4
Under 1
66 49.5 33 16.5 0 16.5 33 49.5 66
Rate (per 100,000)
Prevalence
1990
2019
44 33 22 11 0 11 22 33 44
Rate (per 100,000)
Sex
Female
Male
Deaths
1990
2019
14 10.5 7 3.5 0 3.5 7 10.5 14
Rate (per 100,000)
DALYs
1990
2019
156 117 78 39 0 39 78 117 156
Rate (per 100,000)

# Egypt

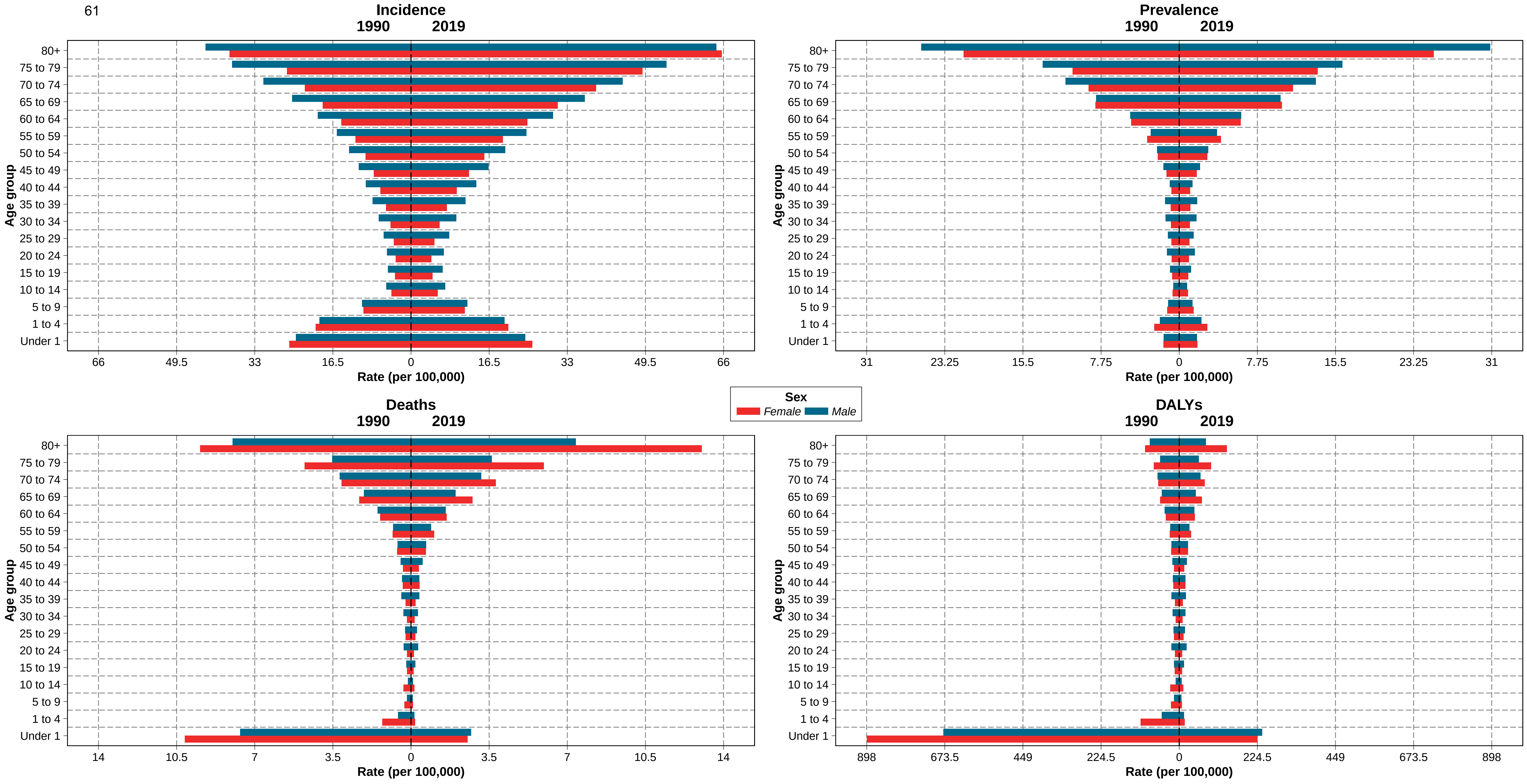
Incidence
1990
2019
Prevalence
1990
2019
Deaths
1990
2019
DALYs
1990
2019
Age group
80+
75 to 79
70 to 74
65 to 69
60 to 64
55 to 59
50 to 54
45 to 49
40 to 44
35 to 39
30 to 34
25 to 29
20 to 24
15 to 19
10 to 14
5 to 9
1 to 4
Under 1
66 49.5 33 16.5 0 16.5 33 49.5 66
31 23.25 15.5 7.75 0 7.75 15.5 23.25 31
14 10.5 7 3.5 0 3.5 7 10.5 14
898 673.5 449 224.5 0 224.5 449 673.5 898
Rate (per 100,000)
Sex
Female
Male

# Iran (Islamic Republic of)



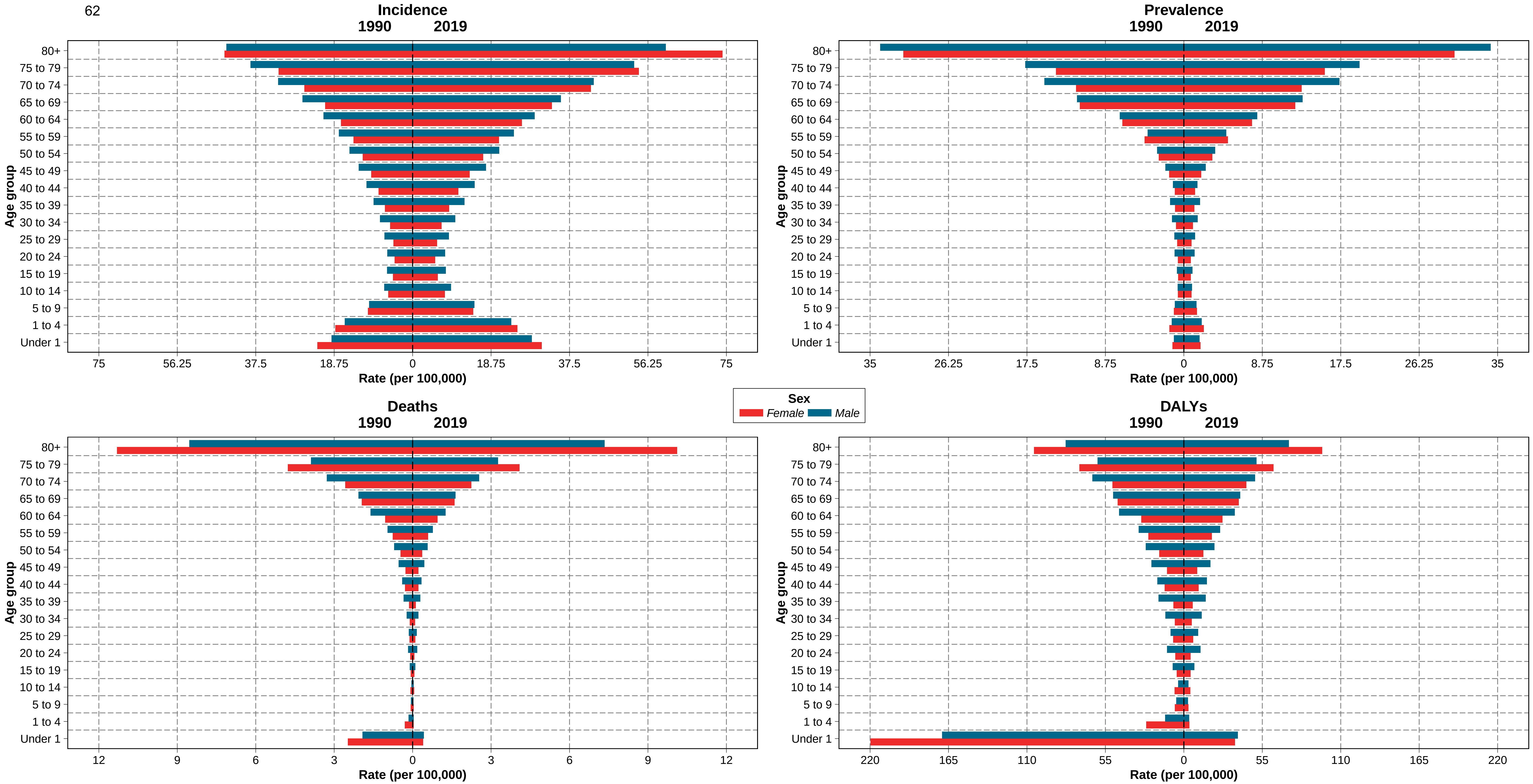

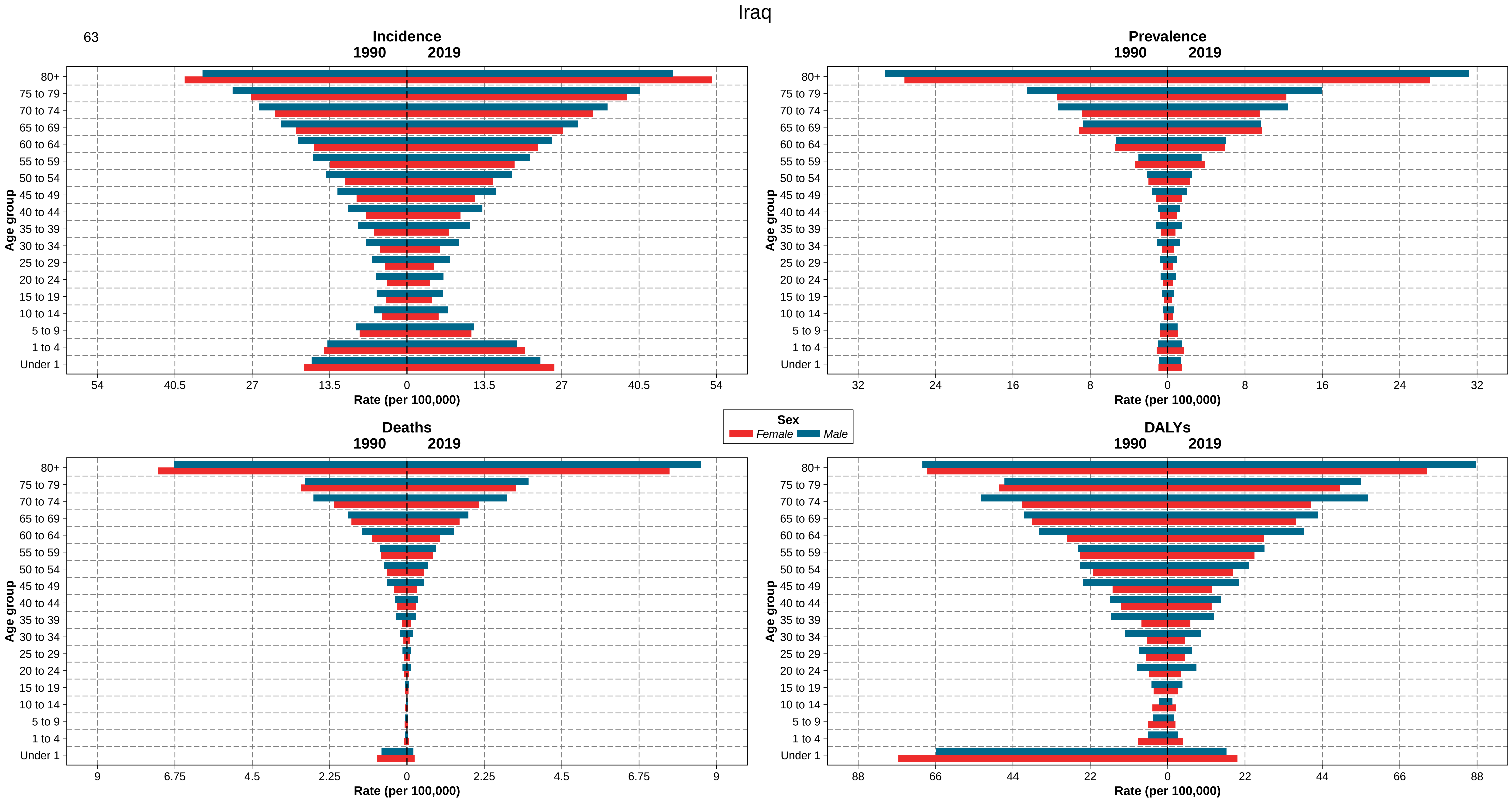
Iraq
Incidence
1990
2019
Prevalence
1990
2019
Deaths
1990
2019
DALYs
1990
2019
Age group
80+
75 to 79
70 to 74
65 to 69
60 to 64
55 to 59
50 to 54
45 to 49
40 to 44
35 to 39
30 to 34
25 to 29
20 to 24
15 to 19
10 to 14
5 to 9
1 to 4
Under 1
Rate (per 100,000)
54 40.5 27 13.5 0 13.5 27 40.5 54
32 24 16 8 0 8 16 24 32
9 6.75 4.5 2.25 0 2.25 4.5 6.75 9
88 66 44 22 0 22 44 66 88
Sex
Female
Male

# Jordan

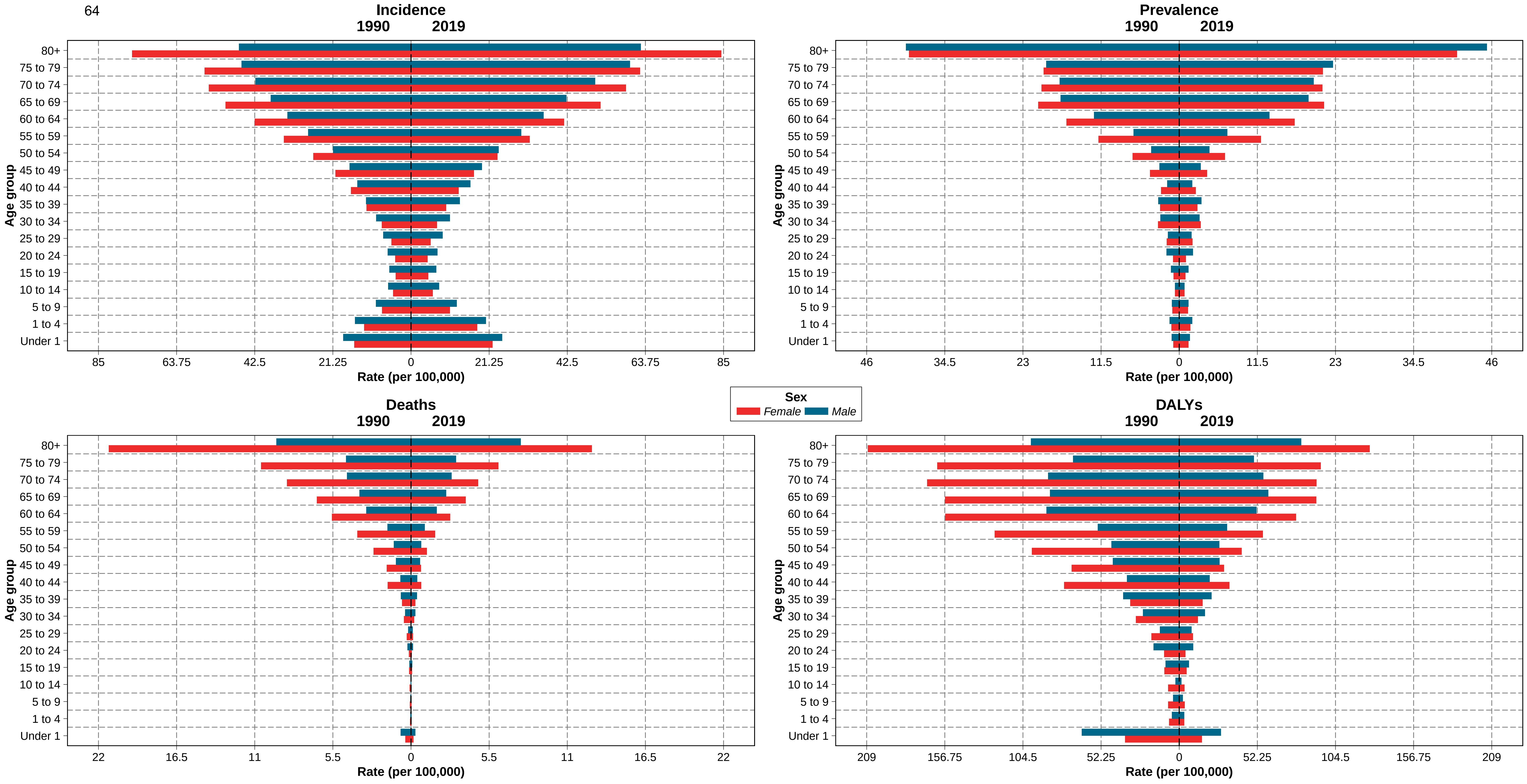
Incidence
1990
2019
Prevalence
Deaths
DALYs
Age group
80+
75 to 79
70 to 74
65 to 69
60 to 64
55 to 59
50 to 54
45 to 49
40 to 44
35 to 39
30 to 34
25 to 29
20 to 24
15 to 19
10 to 14
5 to 9
1 to 4
Under 1
85
63.75
42.5
21.25
0
46
34.5
23
11.5
22
16.5
11
5.5
209
156.75
104.5
52.25
Rate (per 100,000)
Sex
Female
Male

# Kuwait

65

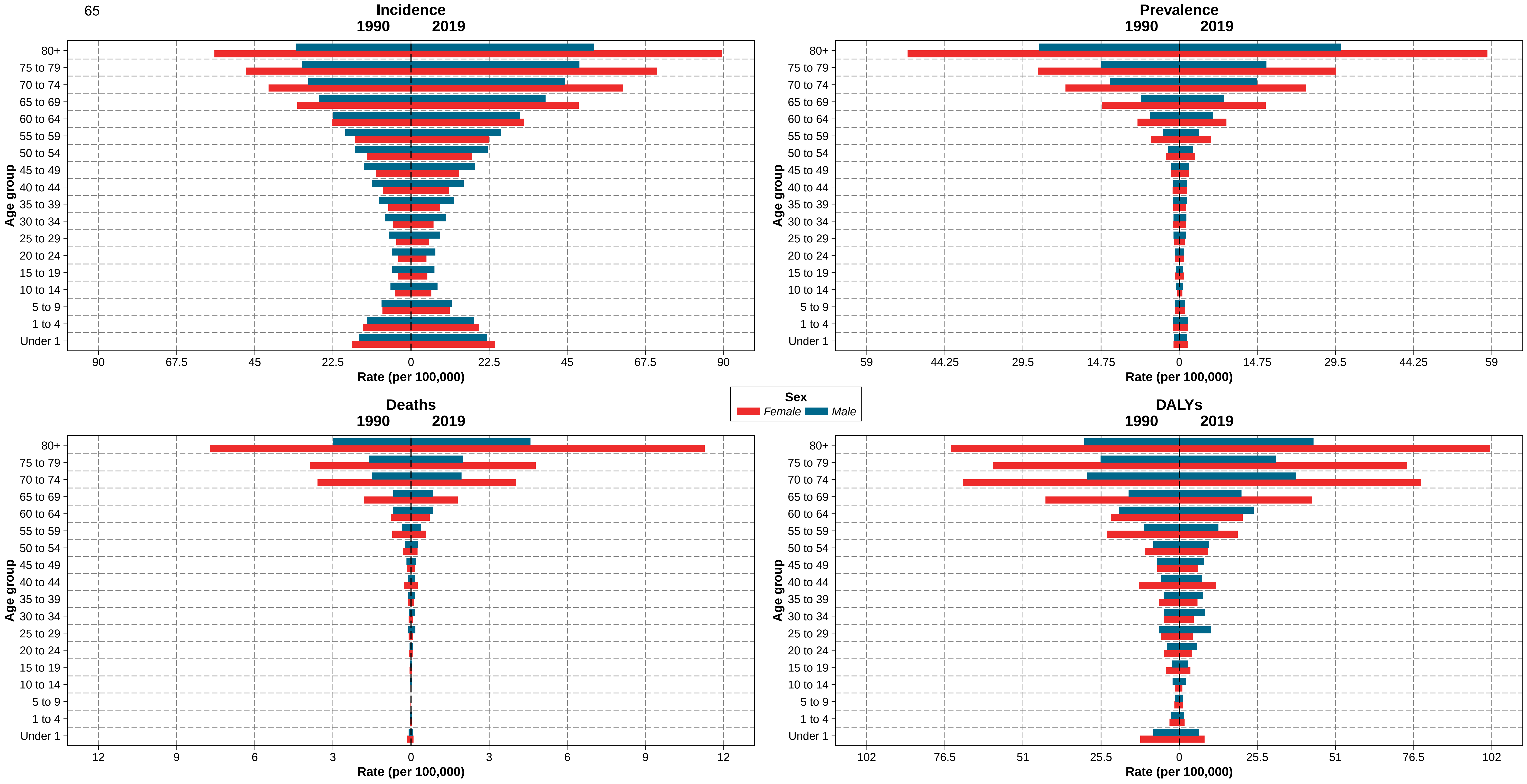

Incidence
1990
2019
Prevalence
1990
2019
Deaths
1990
2019
DALYs
1990
2019
Age group
80+
75 to 79
70 to 74
65 to 69
60 to 64
55 to 59
50 to 54
45 to 49
40 to 44
35 to 39
30 to 34
25 to 29
20 to 24
15 to 19
10 to 14
5 to 9
1 to 4
Under 1
90 67.5 45 22.5 0 22.5 45 67.5 90
59 44.25 29.5 14.75 0 14.75 29.5 44.25 59
12 9 6 3 0 3 6 9 12
102 76.5 51 25.5 0 25.5 51 76.5 102
Rate (per 100,000)
Sex
Female
Male

# Lebanon

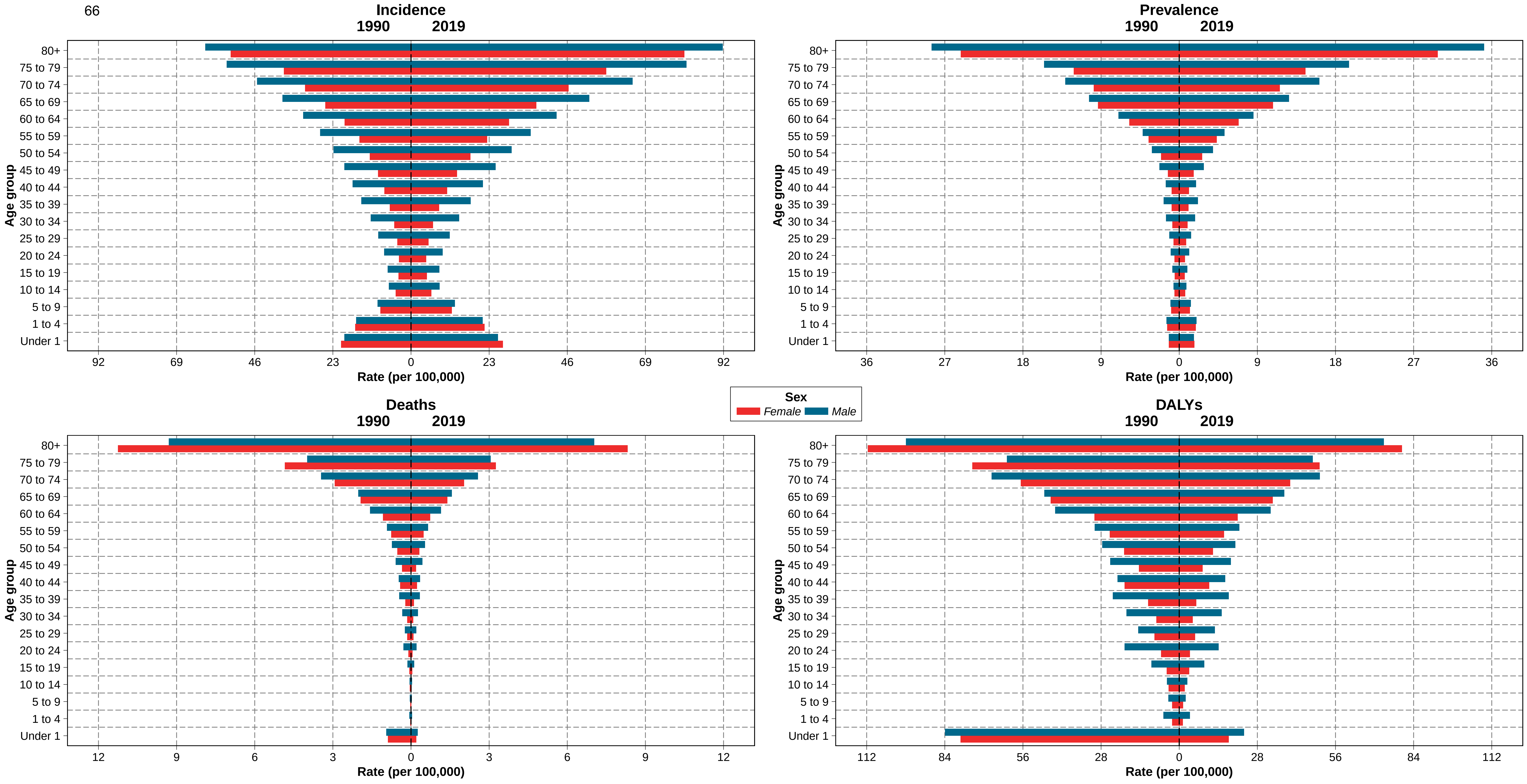
66
Incidence
1990
2019
Prevalence
1990
2019
Deaths
1990
2019
DALYs
1990
2019
80+
75 to 79
70 to 74
65 to 69
60 to 64
55 to 59
50 to 54
45 to 49
40 to 44
35 to 39
30 to 34
25 to 29
20 to 24
15 to 19
10 to 14
5 to 9
1 to 4
Under 1
Age group
92 69 46 23 0 23 46 69 92
36 27 18 9 0 9 18 27 36
12 9 6 3 0 3 6 9 12
112 84 56 28 0 28 56 84 112
Rate (per 100,000)
Sex
Female
Male

# Libya

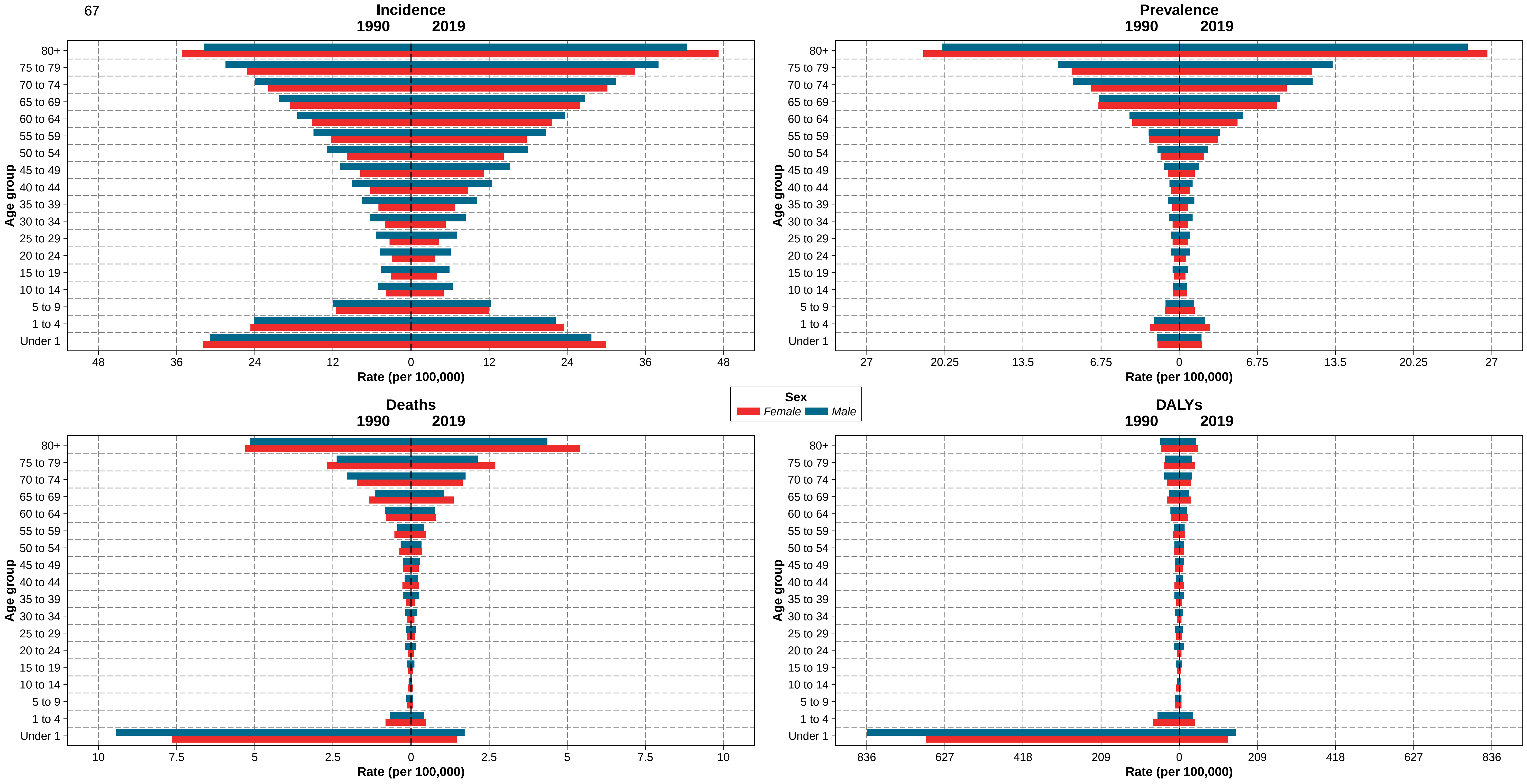
Incidence
1990
2019
Prevalence
Deaths
DALYs
Age group
80+
75 to 79
70 to 74
65 to 69
60 to 64
55 to 59
50 to 54
45 to 49
40 to 44
35 to 39
30 to 34
25 to 29
20 to 24
15 to 19
10 to 14
5 to 9
1 to 4
Under 1
48 36 24 12 0 12 24 36 48
27 20.25 13.5 6.75 0 6.75 13.5 20.25 27
10 7.5 5 2.5 0 2.5 5 7.5 10
836 627 418 209 0 209 418 627 836
Rate (per 100,000)
Sex
Female
Male

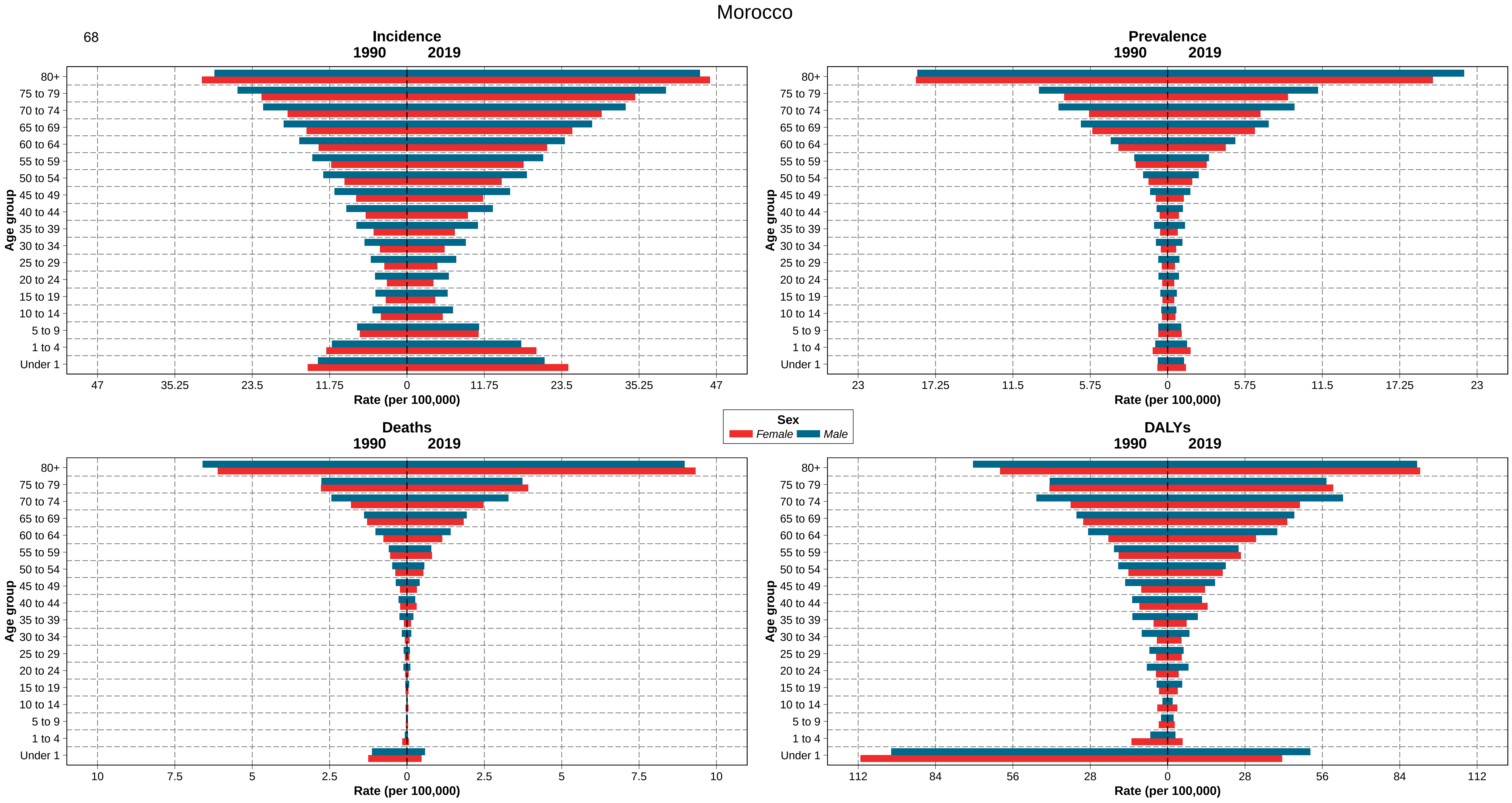
Morocco
Incidence
1990
2019
Prevalence
1990
2019
Deaths
1990
2019
DALYs
1990
2019
Sex
Female
Male
Age group
80+
75 to 79
70 to 74
65 to 69
60 to 64
55 to 59
50 to 54
45 to 49
40 to 44
35 to 39
30 to 34
25 to 29
20 to 24
15 to 19
10 to 14
5 to 9
1 to 4
Under 1
47 35.25 23.5 11.75 0 11.75 23.5 35.25 47
23 17.25 11.5 5.75 0 5.75 11.5 17.25 23
10 7.5 5 2.5 0 2.5 5 7.5 10
112 84 56 28 0 28 56 84 112
Rate (per 100,000)

# Oman



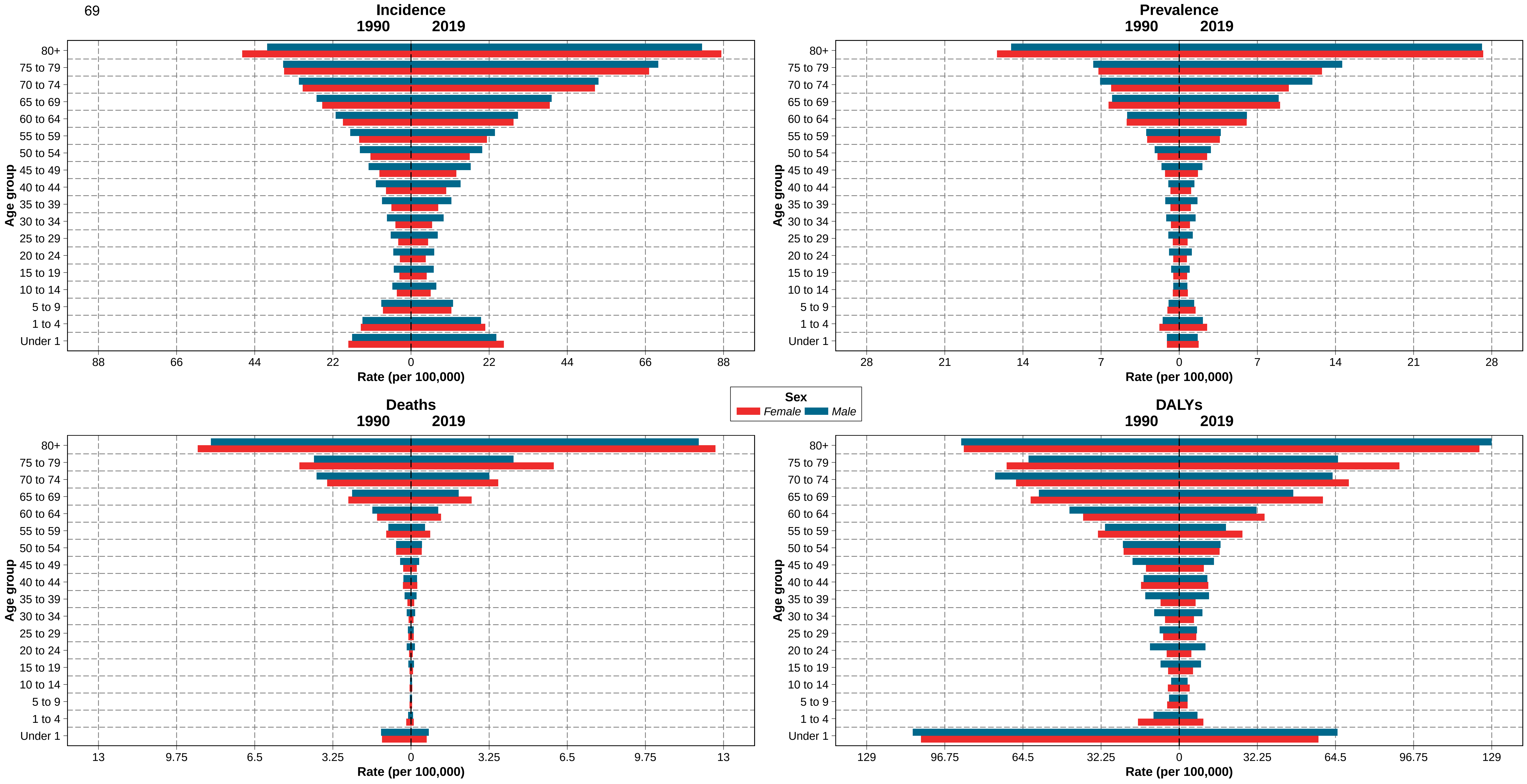
Incidence
1990
2019
Prevalence
Deaths
DALYs
80+
75 to 79
70 to 74
65 to 69
60 to 64
55 to 59
50 to 54
45 to 49
40 to 44
35 to 39
30 to 34
25 to 29
20 to 24
15 to 19
10 to 14
5 to 9
1 to 4
Under 1
Age group
88
66
44
22
0
28
21
14
7
13
9.75
6.5
3.25
129
96.75
64.5
32.25
Rate (per 100,000)
Sex
Female
Male

# Palestine

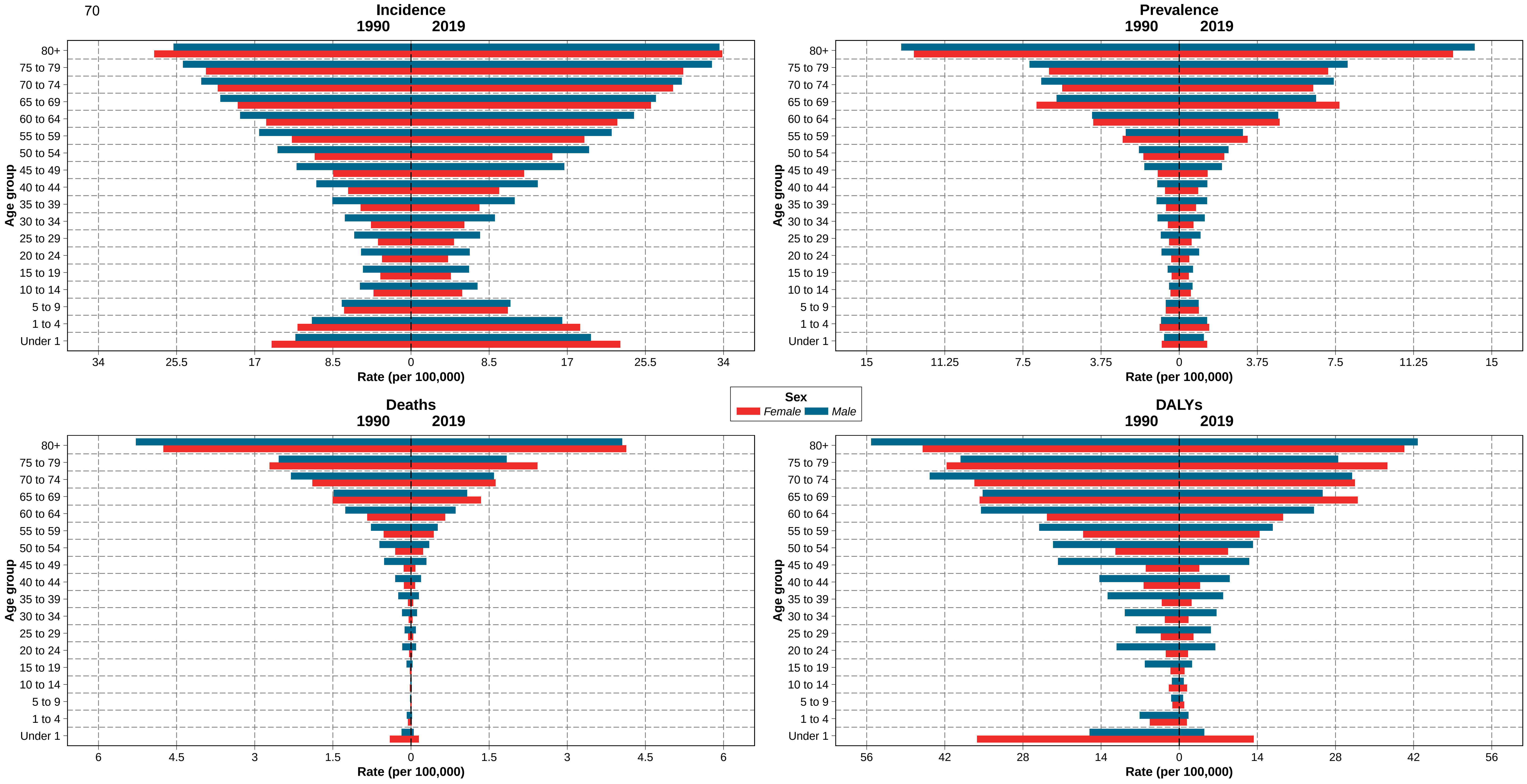
Incidence
1990
2019
80+
75 to 79
70 to 74
65 to 69
60 to 64
55 to 59
50 to 54
45 to 49
40 to 44
35 to 39
30 to 34
25 to 29
20 to 24
15 to 19
10 to 14
5 to 9
1 to 4
Under 1
Age group
34 25.5 17 8.5 0 8.5 17 25.5 34
Rate (per 100,000)
Prevalence
1990
2019
15 11.25 7.5 3.75 0 3.75 7.5 11.25 15
Rate (per 100,000)
Sex
Female
Male
Deaths
1990
2019
6 4.5 3 1.5 0 1.5 3 4.5 6
Rate (per 100,000)
DALYs
1990
2019
56 42 28 14 0 14 28 42 56
Rate (per 100,000)

Qatar

71

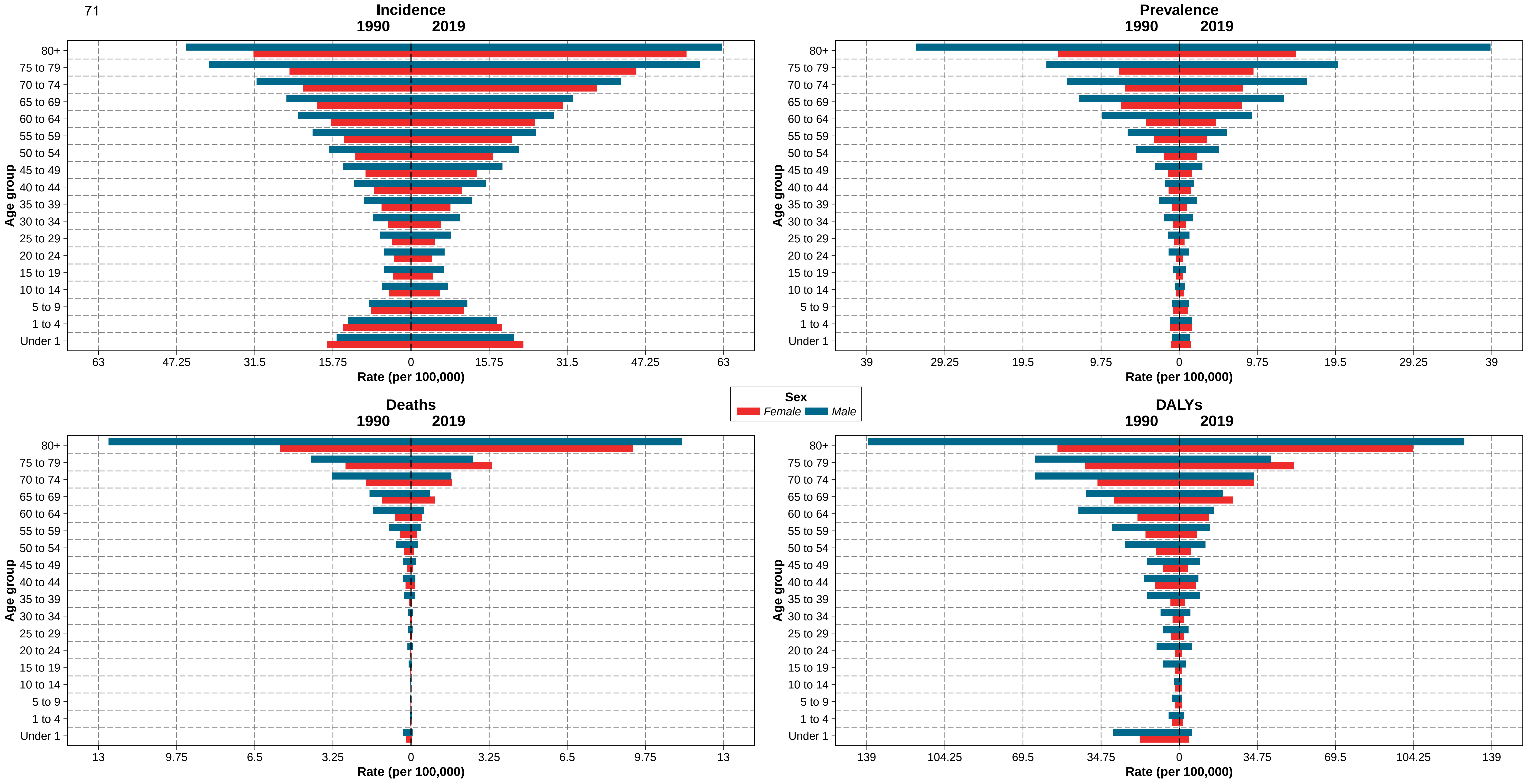

Incidence
1990
2019
80+
75 to 79
70 to 74
65 to 69
60 to 64
55 to 59
50 to 54
45 to 49
40 to 44
35 to 39
30 to 34
25 to 29
20 to 24
15 to 19
10 to 14
5 to 9
1 to 4
Under 1
Age group
63
47.25
31.5
15.75
0
15.75
31.5
47.25
63
Rate (per 100,000)
Prevalence
1990
2019
39
29.25
19.5
9.75
0
9.75
19.5
29.25
39
Rate (per 100,000)
Sex
Female
Male
Deaths
1990
2019
13
9.75
6.5
3.25
0
3.25
6.5
9.75
13
Rate (per 100,000)
DALYs
1990
2019
139
104.25
69.5
34.75
0
34.75
69.5
104.25
139
Rate (per 100,000)

# Saudi Arabia

72

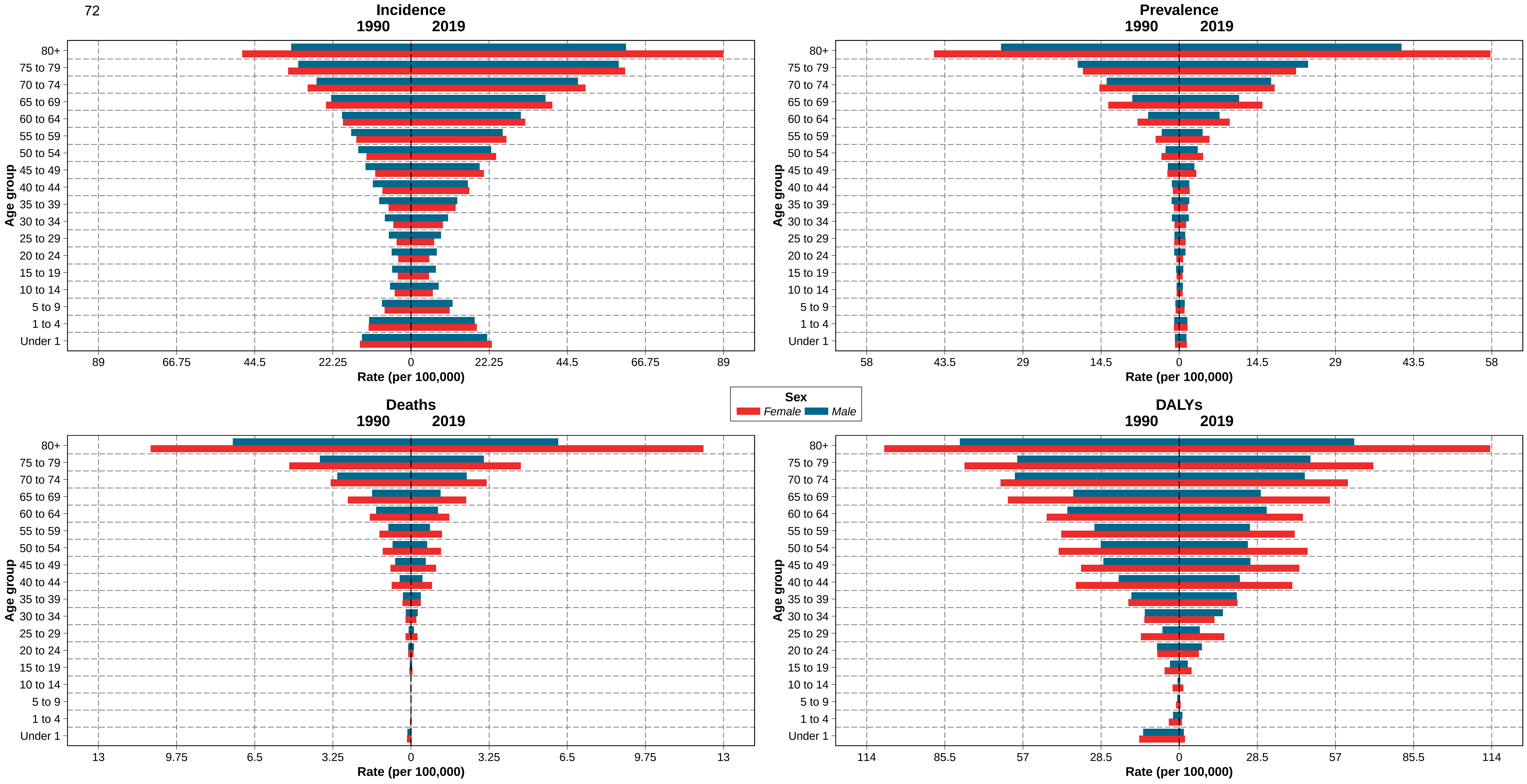

# Sudan

73

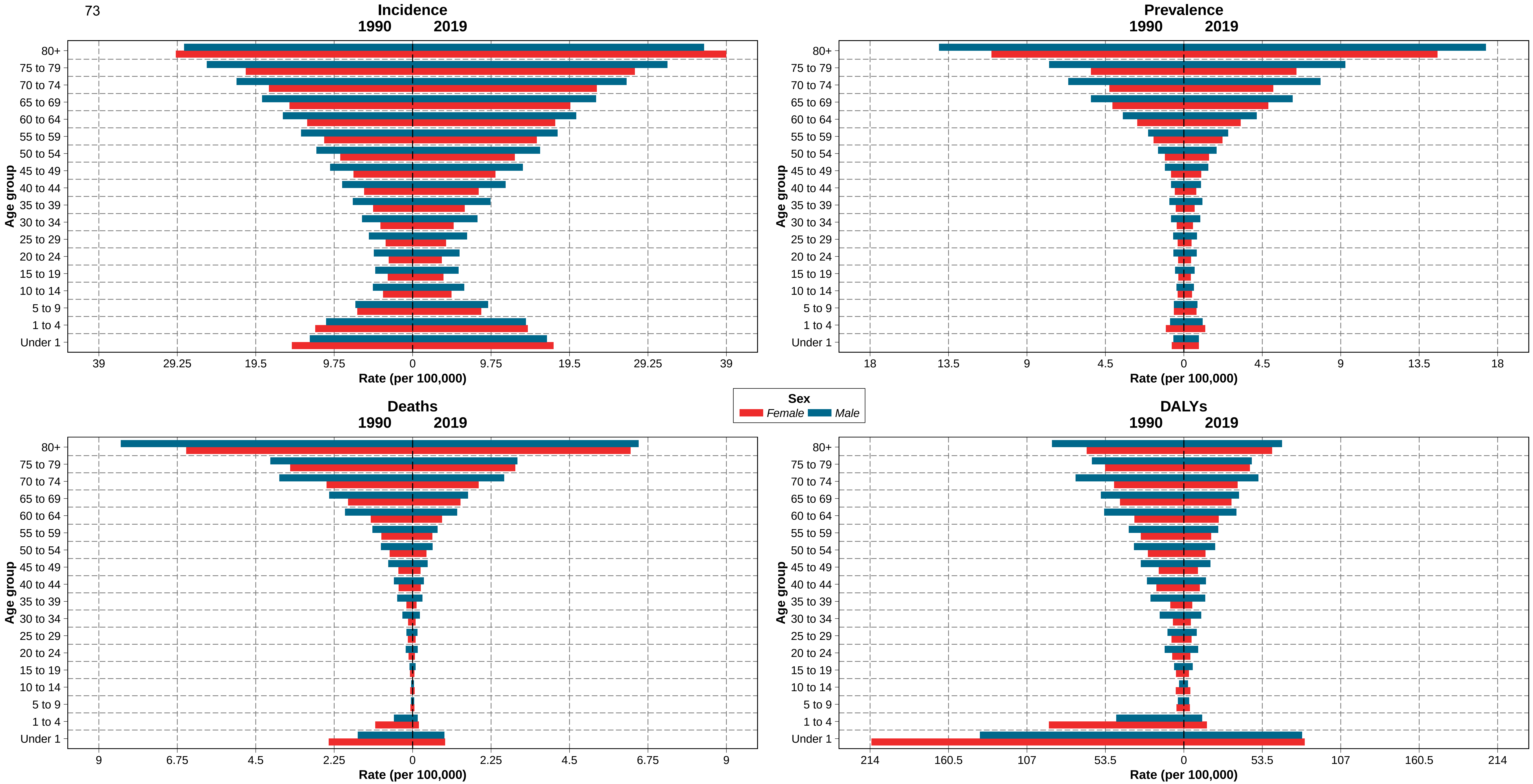

Incidence
1990
2019
Prevalence
1990
2019
Deaths
1990
2019
DALYs
1990
2019
Age group
80+
75 to 79
70 to 74
65 to 69
60 to 64
55 to 59
50 to 54
45 to 49
40 to 44
35 to 39
30 to 34
25 to 29
20 to 24
15 to 19
10 to 14
5 to 9
1 to 4
Under 1
Rate (per 100,000)
39
29.25
19.5
9.75
0
9.75
19.5
29.25
39
18
13.5
9
4.5
0
4.5
9
13.5
18
9
6.75
4.5
2.25
0
2.25
4.5
6.75
9
214
160.5
107
53.5
0
53.5
107
160.5
214
Sex
Female
Male

# Syrian Arab Republic

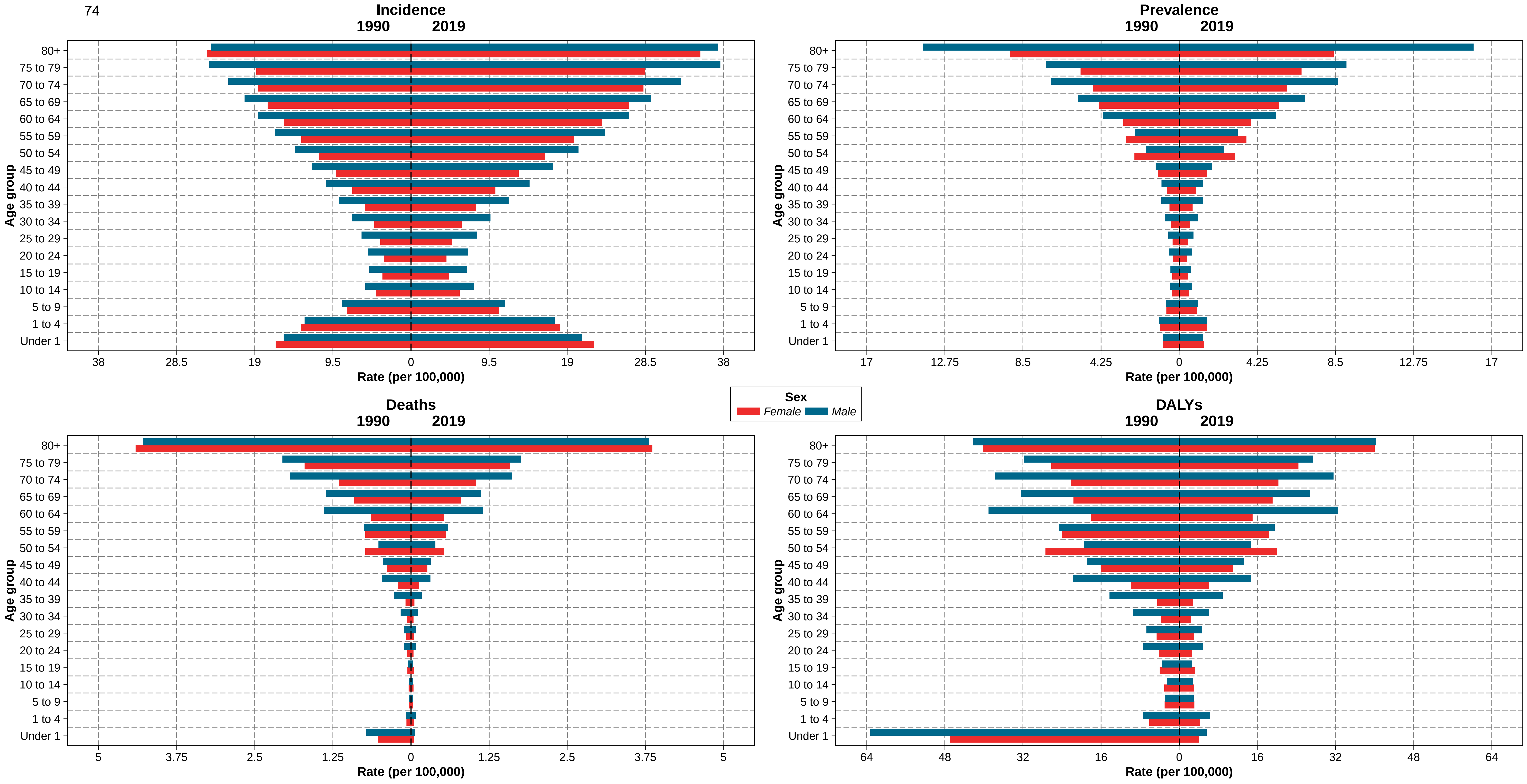

Tunisia

75

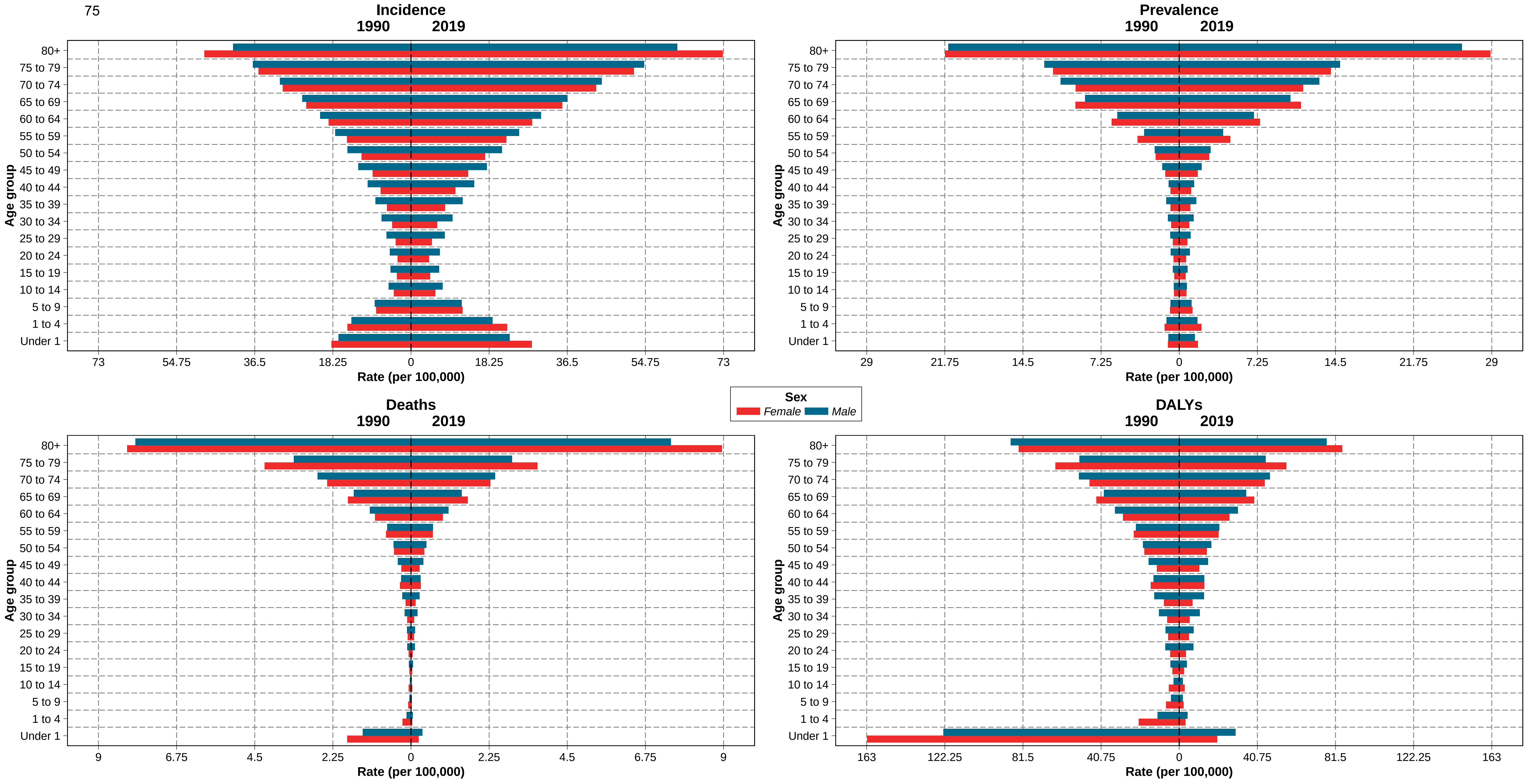
Incidence
1990
2019
80+
75 to 79
70 to 74
65 to 69
60 to 64
55 to 59
50 to 54
45 to 49
40 to 44
35 to 39
30 to 34
25 to 29
20 to 24
15 to 19
10 to 14
5 to 9
1 to 4
Under 1
Age group
73
54.75
36.5
18.25
0
18.25
36.5
54.75
73
Rate (per 100,000)
Prevalence
1990
2019
Age group
29
21.75
14.5
7.25
0
7.25
14.5
21.75
29
Rate (per 100,000)
Sex
Female
Male
Deaths
1990
2019
Age group
9
6.75
4.5
2.25
0
2.25
4.5
6.75
9
Rate (per 100,000)
DALYs
1990
2019
Age group
163
122.25
81.5
40.75
0
40.75
81.5
122.25
163
Rate (per 100,000)

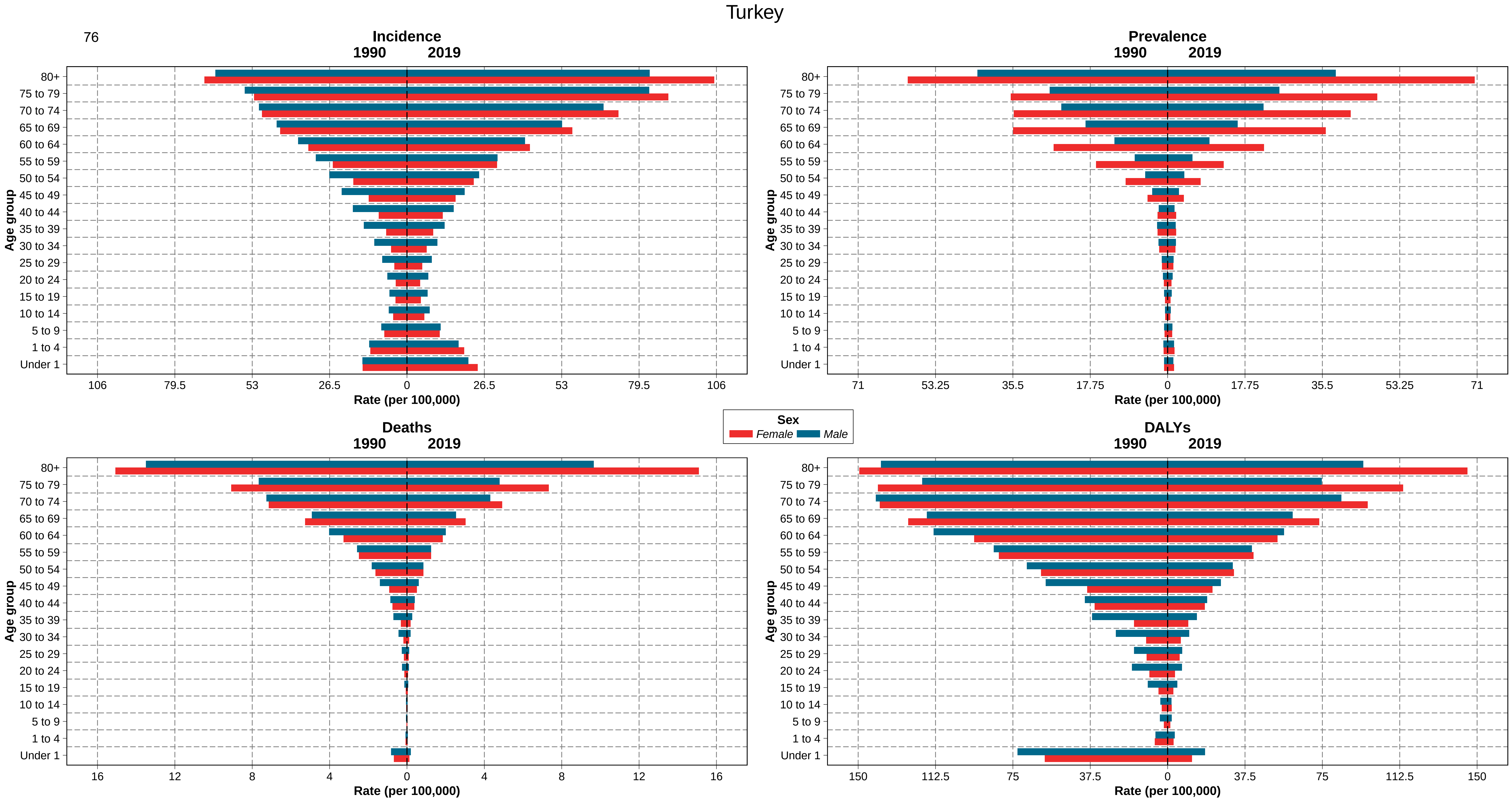
Turkey
Incidence
1990
2019
Prevalence
Deaths
DALYs
Age group
80+
75 to 79
70 to 74
65 to 69
60 to 64
55 to 59
50 to 54
45 to 49
40 to 44
35 to 39
30 to 34
25 to 29
20 to 24
15 to 19
10 to 14
5 to 9
1 to 4
Under 1
106
79.5
53
26.5
0
71
53.25
35.5
17.75
16
12
8
4
150
112.5
75
37.5
Rate (per 100,000)
Sex
Female
Male

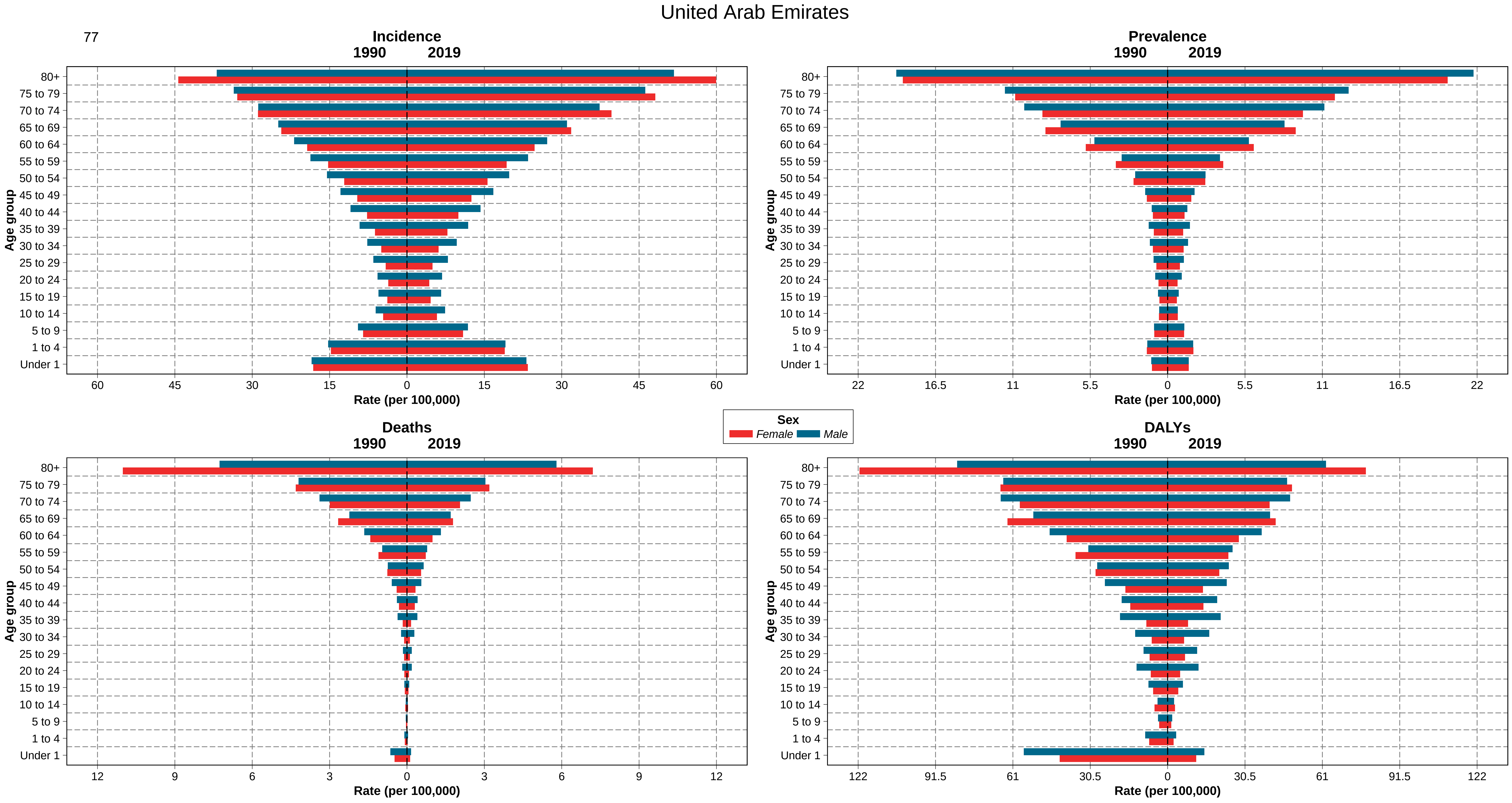

United Arab Emirates
Incidence
1990
2019
Prevalence
1990
2019
Deaths
1990
2019
DALYs
1990
2019
Age group
80+
75 to 79
70 to 74
65 to 69
60 to 64
55 to 59
50 to 54
45 to 49
40 to 44
35 to 39
30 to 34
25 to 29
20 to 24
15 to 19
10 to 14
5 to 9
1 to 4
Under 1
60 45 30 15 0 15 30 45 60
22 16.5 11 5.5 0 5.5 11 16.5 22
12 9 6 3 0 3 6 9 12
122 91.5 61 30.5 0 30.5 61 91.5 122
Rate (per 100,000)
Sex
Female
Male

# Yemen

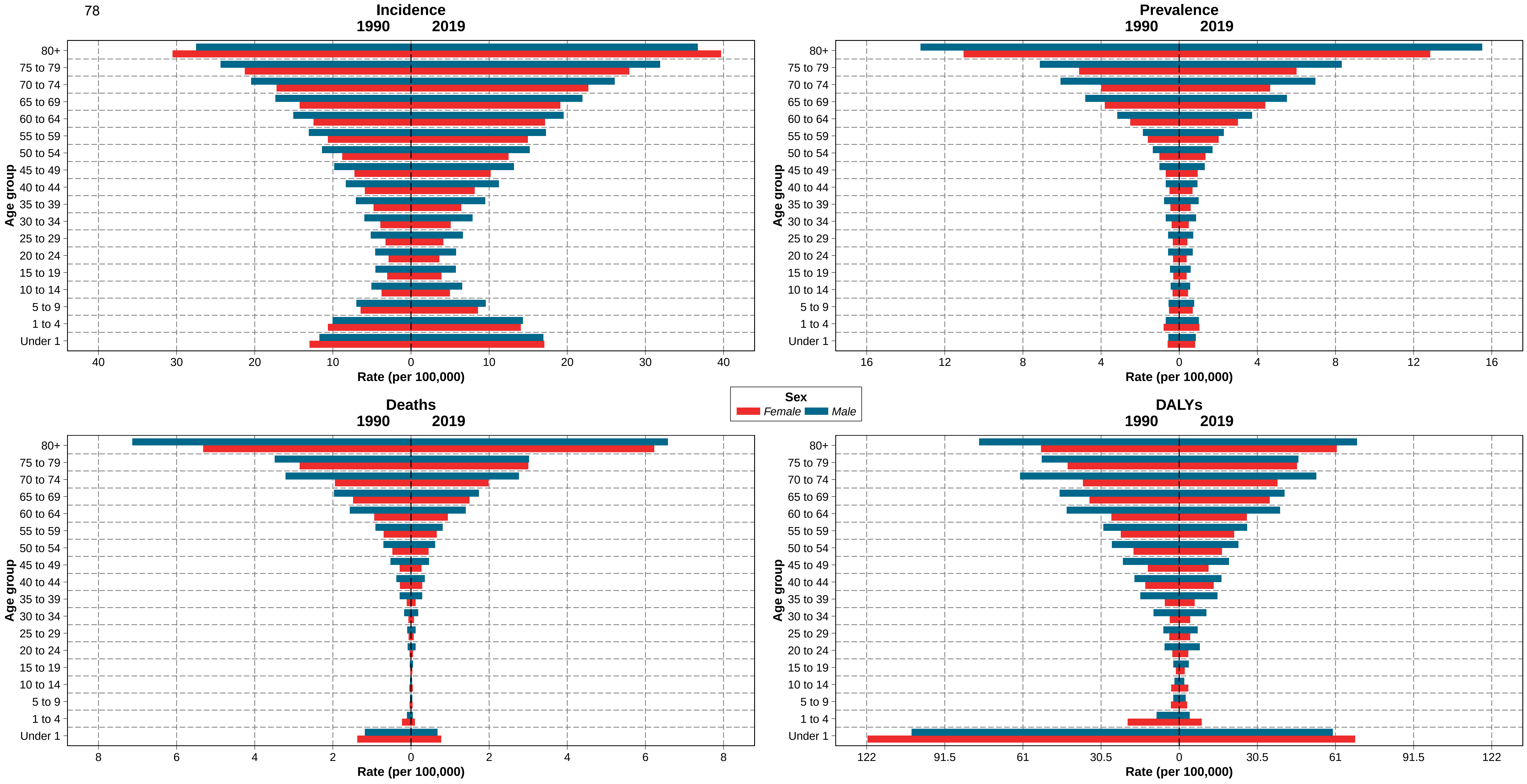

Incidence
1990
2019
Prevalence
1990
2019
Deaths
1990
2019
DALYs
1990
2019
Age group
80+
75 to 79
70 to 74
65 to 69
60 to 64
55 to 59
50 to 54
45 to 49
40 to 44
35 to 39
30 to 34
25 to 29
20 to 24
15 to 19
10 to 14
5 to 9
1 to 4
Under 1
Rate (per 100,000)
40 30 20 10 0 10 20 30 40
16 12 8 4 0 4 8 12 16
8 6 4 2 0 2 4 6 8
122 91.5 61 30.5 0 30.5 61 91.5 122
Sex
Female
Male

**S20 Fig.**

**Temporal trend of age-standardized incidence rate per 100,000 in North Africa and the Middle East region and the 21 countries of this region**

**(Reference: https://vizhub.healthdata.org/gbd-compare/)**

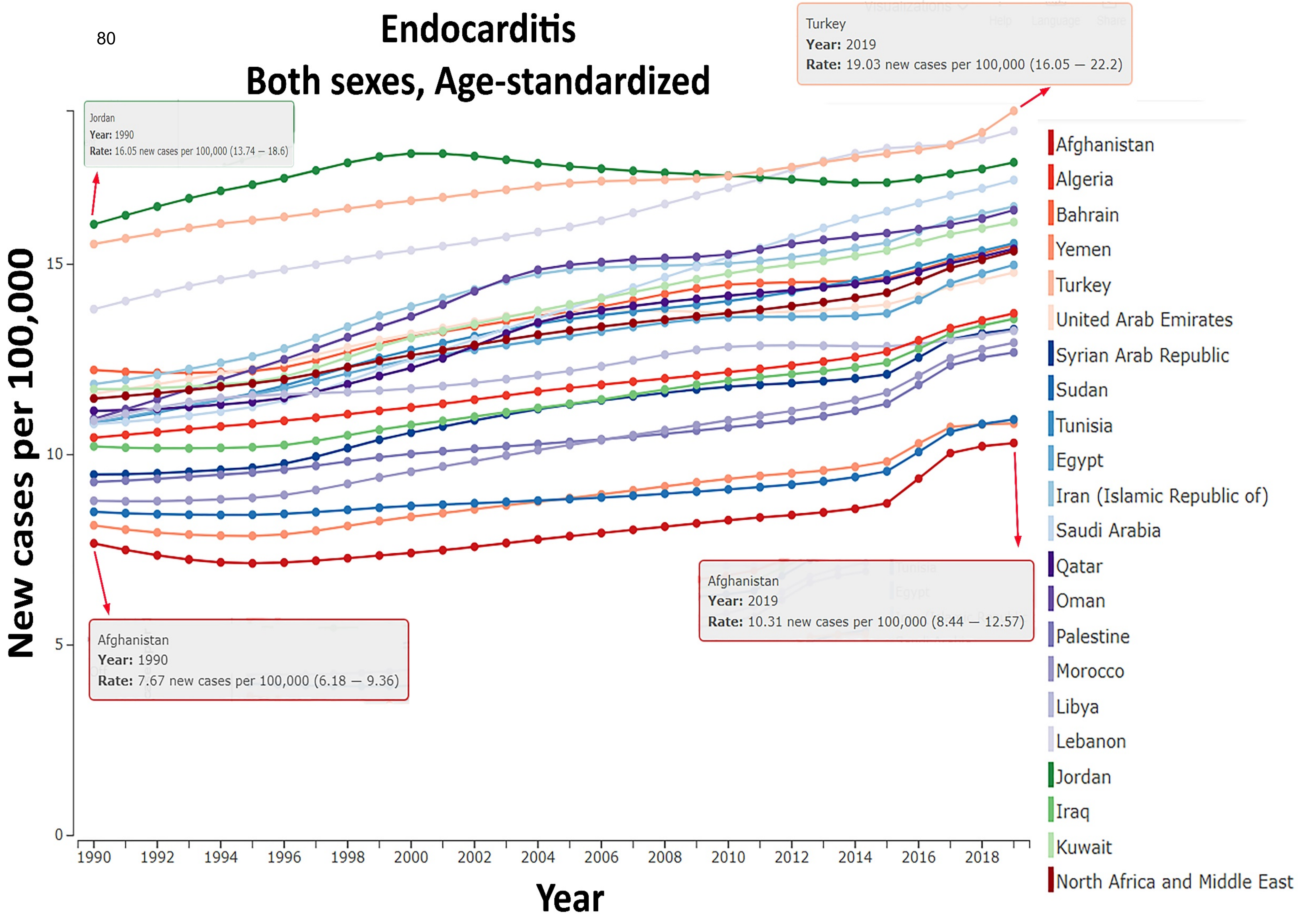
Endocarditis
Both sexes, Age-standardized
Turkey
Year: 2019
Rate: 19.03 new cases per 100,000 (16.05 — 22.2)
Jordan
Year: 1990
Rate: 16.05 new cases per 100,000 (13.74 — 18.6)
Afghanistan
Year: 2019
Rate: 10.31 new cases per 100,000 (8.44 — 12.57)
Afghanistan
Year: 1990
Rate: 7.67 new cases per 100,000 (6.18 — 9.36)
New cases per 100,000
0
5
10
15
Year
1990
1992
1994
1996
1998
2000
2002
2004
2006
2008
2010
2012
2014
2016
2018
Afghanistan
Algeria
Bahrain
Yemen
Turkey
United Arab Emirates
Syrian Arab Republic
Sudan
Tunisia
Egypt
Iran (Islamic Republic of)
Saudi Arabia
Qatar
Oman
Palestine
Morocco
Libya
Lebanon
Jordan
Iraq
Kuwait
North Africa and Middle East
