# Supplementary material for: Infective Endocarditis in North Africa and the Middle East, 1990‒2019: Updates from the Global Burden of Disease Study 2019
Source: Arch Iran Med. 2024 May 1;27(5):229–38. doi: 10.34172/aim.2024.34 (PMC11097324; doi:10.34172/aim.2024.34)
Supplement: Supplementary file 3 — contains Tables S1 to S13. [file aim-27-229-s003.pdf]

S3 Appendix

Table of Contents		
**Number**	**Title**	**Page**

Number	Title	Page
**S1 Table**	**Age-standardized rates and percent changes in age-standardized rates of incidence of infective endocarditis in North Africa and the Middle East and its 21 countries between 1990 and 2019, sorted from largest to smallest**	**1**
**S2 Table**	**Decomposition analysis of the incidence rate change between 1990 and 2019 in North Africa and the Middle East and the twenty-one countries of this region according to sex**	**2**
**S3 Table**	**Age-standardized rates and percent changes in age-standardized rates of prevalence of infective endocarditis in North Africa and the Middle East and its 21 countries between 1990 and 2019, sorted from largest to smallest**	**4**
**S4 Table**	**Age-standardized rates and percent changes in age-standardized rates of death of infective endocarditis in North Africa and the Middle East and its 21 countries between 1990 and 2019, sorted from largest to smallest**	**5**

Table of Contents		
**Number**	**Title**	**Page**
**S5 Table**	**Age-standardized rate of death (ASDR) due to resistant culprit pathogens of infective endocarditis and other cardiac infections in North America and the Middle East (NAME) and its 21 countries for both sexes**	**6**
**S6 Table**	**Age-standardized rates and percent changes in age-standardized rates of DALY of infective endocarditis in North Africa and the Middle East and its 21 countries between 1990 and 2019, sorted from largest to smallest**	**7**
**S7 Table**	**Age-standardized rate of DALY due to resistant culprit pathogens of infective endocarditis and other cardiac infections in North America and the Middle East (NAME) and its 21 countries for both sexes**	**8**
**S8 Table**	**Age-standardized rates and percent changes in age-standardized rates of YLL of infective endocarditis in North Africa and the Middle East and its 21 countries between 1990 and 2019, sorted from largest to smallest**	**9**

Table of Contents		
**Number**	**Title**	**Page**

Number	Title	Page
**S9 Table**	**Age-standardized rates and percent changes in age-standardized rates of YLD of infective endocarditis in North Africa and the Middle East and its 21 countries between 1990 and 2019, sorted from largest to smallest**	**10**
**S10 Table**	**Age-standardized rates of incidence, prevalence, deaths, DALYs, YLLs, and YLDs of infective endocarditis in North Africa and the Middle East in 1990, 1997, 2007 and 2019**	**11**
**S11 Table**	**Age-standardized incidence, prevalence, deaths, Disability-Adjusted Life Years (DALYs), Years of Life Lost (YLLs) and Years Lived with Disability (YLDs) rates and number in twenty-one countries of North Africa and the Middle East in1990, 1997, 2007, and 2019 according to sex**	**12**
**S12 Table**	**Summary of the highest and lowest epidemiologic measures and burden of infective endocarditis in North Africa and the Middle East at a glance**	**50**
**S13 Table**	**Predisposing conditions and risk factors for infective endocarditis**	**51**

S1 Table. Age-standardized incidence rate (ASIR) and percent change in ASIR between 1990 and 2019 in the North Africa and the Middle East (NAME) region and its 21 countries, sorted from largest to smallest							
**Age-standardized incidence rate (ASIR) sorted from largest to smallest**				**Percent change in age-standardized incidence rate (ASIR) sorted from largest to smallest**			
**Location's name**	**value**	**Upper bound**	**Lower bound**	**Location's name**	**value**	**Upper bound**	**Lower bound**
**Türkiye**	**19.03**	**22.20**	**16.05**	**Saudi Arabia**	**0.59**	**0.68**	**0.51**
Lebanon	18.50	21.75	15.69	Oman	0.50	0.58	0.43
Jordan	17.68	21.06	14.89	Morocco	0.47	0.56	0.40
Saudi Arabia	17.21	20.43	14.42	Tunisia	0.44	0.50	0.37
Iran (Islamic Republic of)	16.52	19.79	13.71	Syrian Arab Republic	0.40	0.48	0.33
Oman	16.42	19.39	13.76	Iran (Islamic Republic of)	0.39	0.42	0.37
Kuwait	16.11	19.42	13.28	Egypt	0.38	0.46	0.31
Tunisia	15.55	18.65	13.00	Qatar	0.38	0.45	0.31
Bahrain	15.50	18.66	12.82	Kuwait	0.37	0.44	0.32
Qatar	15.40	18.53	12.61	Palestine	0.37	0.43	0.31
**North Africa and the Middle East**	**15.35**	**18.20**	**12.89**	Afghanistan	0.34	0.41	0.28
Egypt	14.98	17.59	12.63	Lebanon	0.34	0.40	0.28
United Arab Emirates	14.79	17.46	12.25	**North Africa and the Middle East**	**0.34**	**0.37**	**0.31**
Algeria	13.71	16.35	11.37	Yemen	0.33	0.38	0.27
Iraq	13.57	16.12	11.23	Iraq	0.33	0.39	0.27
Syrian Arab Republic	13.29	16.22	10.89	Algeria	0.31	0.37	0.26
Libya	13.25	16.05	10.92	Sudan	0.29	0.35	0.22
Morocco	12.94	15.57	10.64	United Arab Emirates	0.27	0.34	0.22
Palestine	12.68	15.55	10.30	Bahrain	0.27	0.34	0.20
Sudan	10.92	13.28	8.88	Türkiye	0.23	0.30	0.14
Yemen	10.82	13.07	8.82	Libya	0.22	0.28	0.15
**Afghanistan**	**10.31**	**12.57**	**8.44**	**Jordan**	**0.10**	**0.16**	**0.04**

S2 Table . Decomposition analysis of the incidence rate change between 1990 and 2019 in North Africa and the Middle East and the twenty-one countries of this region according to sex

	Location	Sex	New cases		Expected new cases in 2019		% 1990 - 2019 new cases change cause			% 1990 - 2019 new cases overall change
			1990	2019	Population growth	Population growth + Aging	Population growth	Age structure change	Incidence rate change	
North Africa and the MiddleEast		Female	14854	36488	25818	26635	73.8%	5.5%	66.3%	145.6%
		Male	18517	45039	33129	35402	78.9%	12.3%	52%	143.2%
Countries	Afghanistan	Female	380	1579	1228	1151	223%	-20.3%	112.5%	315.3%
		Male	463	2011	1611	1485	247.7%	-27.2%	113.6%	334%
	Algeria	Female	1002	2395	1655	1801	65.2%	14.6%	59.3%	139.1%
		Male	1193	2914	1979	2244	65.8%	22.2%	56.2%	144.2%
	Bahrain	Female	17	64	44	51	155.2%	37.8%	77%	270.1%
		Male	29	140	88	108	204.8%	70.6%	108.1%	383.5%
	Egypt	Female	2468	5395	4329	4054	75.5%	-11.2%	54.4%	118.7%
		Male	3056	7306	5505	5515	80.2%	0.3%	58.6%	139.1%
	Iran (Islamic Republic of)	Female	2801	5974	4060	4223	44.9%	5.8%	62.5%	113.3%
		Male	3268	7018	4676	5155	43.1%	14.7%	57%	114.8%
	Iraq	Female	720	2164	1717	1615	138.4%	-14.2%	76.2%	200.4%
		Male	866	2643	2081	2044	140.3%	-4.4%	69.2%	205.1%
	Jordan	Female	197	727	594	704	201.6%	55.5%	11.7%	268.8%
		Male	226	926	710	790	214.6%	35.5%	60.4%	310.6%
	Kuwait	Female	60	242	164	178	173.6%	22.4%	107.4%	303.4%
		Male	96	351	226	256	135.2%	31.5%	99.6%	266.4%
	Lebanon	Female	163	412	264	294	62.2%	18.1%	73%	153.3%
		Male	246	550	378	420	54%	16.9%	53%	123.9%
	Libya	Female	205	340	330	270	61.1%	-29.4%	34.2%	65.9%
		Male	246	439	386	352	57%	-13.8%	35.5%	78.7%
	Morocco	Female	881	1983	1241	1318	40.9%	8.7%	75.5%	125.1%
		Male	1064	2381	1526	1663	43.4%	12.8%	67.5%	123.7%

S2 Table. Decomposition analysis of the incidence rate change between 1990 and 2019 in North Africa and the Middle East and the twenty-one countries of this region according to sex

Location		Sex	New cases		Expected new cases in 2019		% 1990 - 2019 new cases change cause			% 1990 - 2019 new casesoverall change
			1990	2019	Population growth	Population growth + Aging	Population growth	Age structure change	Incidence rate change	
North Africa and the MiddleEast		Female	14854	36488	25818	26635	73.8%	5.5%	66.3%	145.6%
		Male	18517	45039	33129	35402	78.9%	12.3%	52%	143.2%
Countries	Oman	Female	67	181	134	124	101.3%	-14.6%	85.2%	171.9%
		Male	101	359	265	257	160.6%	-7.7%	100.4%	253.4%
	Palestine	Female	78	243	185	173	136.7%	-15.9%	89.6%	210.5%
		Male	90	294	217	220	142.1%	2.9%	83.1%	228.1%
	Qatar	Female	11	72	54	52	392.2%	-15.9%	175%	551.4%
		Male	28	265	200	205	618.7%	18.6%	217.7%	855%
	Saudi Arabia	Female	557	1983	1170	1146	110.2%	-4.4%	150.5%	256.3%
		Male	822	2965	1911	2007	132.5%	11.6%	116.6%	260.8%
	Sudan	Female	684	1639	1378	1294	101.3%	-12.2%	50.4%	139.4%
		Male	837	2115	1697	1646	102.8%	-6.1%	56%	152.7%
	Syrian Arab Republic	Female	480	818	566	576	18%	2.1%	50.6%	70.7%
		Male	602	999	644	714	7%	11.6%	47.4%	66%
	Tunisia	Female	354	863	495	590	39.6%	27.1%	77%	143.7%
		Male	428	960	576	673	34.7%	22.6%	67.1%	124.4%
	Türkiye	Female	3224	7851	4405	5819	36.6%	43.9%	63%	143.5%
		Male	4197	7858	5692	7282	35.6%	37.9%	13.7%	87.2%
	United Arab Emirates	Female	52	249	200	196	282.9%	-7.2%	101.7%	377.5%
		Male	116	904	640	710	454.1%	60.1%	168.4%	682.6%
	Yemen	Female	443	1275	1025	960	131.2%	-14.8%	71.1%	187.6%
		Male	532	1593	1211	1190	127.7%	-4%	75.7%	199.4%

S3 Table. Age-standardized prevalence rate (ASPR) and percent change in ASIR between 1990 and 2019 in the North Africa and the Middle East (NAME) region and its 21 countries, sorted from largest to smallest							
**Age-standardized prevalence rate (ASPR) sorted from largest to smallest**				**Percent change in age-standardized prevalence rate (ASPR) sorted from largest to smallest**			
**Location's name**	**value**	**Upper bound**	**Lower bound**	**Location's name**	**value**	**Upper bound**	**Lower bound**
**Türkiye**	**5.73**	**6.34**	**5.15**	**Oman**	**0.45**	**0.55**	**0.36**
Jordan	5.12	5.98	4.39	Syrian Arab Republic	0.28	0.35	0.22
Saudi Arabia	3.92	4.64	3.34	Saudi Arabia	0.26	0.35	0.18
Bahrain	3.59	4.28	3.06	Afghanistan	0.24	0.31	0.16
Kuwait	3.48	4.11	2.95	Egypt	0.24	0.35	0.12
**North Africa and the Middle East**	**3.44**	**3.95**	**3.07**	Sudan	0.23	0.30	0.17
Iran (Islamic Republic of)	3.42	4.09	2.89	Tunisia	0.23	0.30	0.16
Qatar	3.41	4.15	2.88	Libya	0.22	0.29	0.16
Lebanon	3.30	3.82	2.84	Yemen	0.22	0.29	0.16
Egypt	3.12	3.95	2.53	Morocco	0.22	0.28	0.15
Tunisia	2.93	3.39	2.52	Lebanon	0.21	0.27	0.15
Oman	2.90	3.34	2.52	Palestine	0.20	0.26	0.13
Iraq	2.67	3.17	2.28	Qatar	0.16	0.30	0.05
United Arab Emirates	2.64	3.07	2.27	Algeria	0.15	0.21	0.09
Libya	2.57	3.01	2.22	Iran (Islamic Republic of)	0.14	0.18	0.11
Algeria	2.45	2.87	2.11	Iraq	0.13	0.20	0.07
Morocco	2.15	2.51	1.84	Kuwait	0.12	0.20	0.06
Palestine	1.97	2.31	1.69	**North Africa and the Middle East**	**0.12**	**0.20**	**0.05**
Syrian Arab Republic	1.93	2.27	1.66	United Arab Emirates	0.10	0.17	0.05
Sudan	1.70	1.99	1.46	Bahrain	0.09	0.16	0.03
Yemen	1.56	1.83	1.34	Jordan	0.07	0.14	0.01
**Afghanistan**	**1.31**	**1.54**	**1.11**	**Türkiye**	**-0.02**	**0.13**	**-0.15**

S4 Table . Age-standardized prevalence rate (ASDR) and percent change in ASDR between 1990 and 2019 in the North Africa and the Middle East (NAME) region and its 21 countries, sorted from largest to smallest

Age-standardized death rate sorted from largest to smallest				Percent change in age-standardized death rate sorted from largest to smallest			
**Location's name**	**value**	**Upper bound**	**Lower bound**	**Location's name**	**value**	**Upper bound**	**Lower bound**
**Türkiye**	**0.83**	**1.06**	**0.61**	**Morocco**	**0.28**	**0.95**	**-0.13**
Jordan	0.82	1.13	0.65	Kuwait	0.14	0.81	-0.44
Egypt	0.78	1.11	0.46	Afghanistan	0.02	0.39	-0.24
Oman	0.71	0.91	0.51	Oman	0.01	0.94	-0.40
**North Africa and the Middle East**	**0.65**	**0.79**	**0.51**	Iraq	-0.01	0.31	-0.24
Saudi Arabia	0.65	0.84	0.44	Yemen	-0.05	0.33	-0.31
Morocco	0.59	0.77	0.42	Saudi Arabia	-0.08	0.44	-0.47
Algeria	0.57	0.73	0.42	Tunisia	-0.15	0.16	-0.38
Iran (Islamic Republic of)	0.55	0.85	0.45	Qatar	-0.18	0.16	-0.43
Afghanistan	0.55	0.78	0.37	Egypt	-0.19	0.06	-0.40
United Arab Emirates	0.53	0.76	0.34	Syrian Arab Republic	-0.20	0.12	-0.41
Iraq	0.53	0.69	0.38	Iran (Islamic Republic of)	-0.21	-0.05	-0.35
Tunisia	0.51	0.68	0.36	**North Africa and the Middle East**	**-0.22**	**-0.09**	**-0.31**
Lebanon	0.50	0.63	0.36	United Arab Emirates	-0.24	0.04	-0.46
Yemen	0.50	0.80	0.32	Palestine	-0.25	0.03	-0.45
Sudan	0.49	0.80	0.32	Algeria	-0.27	-0.03	-0.46
Bahrain	0.48	0.62	0.36	Lebanon	-0.30	-0.09	-0.49
Libya	0.44	0.61	0.30	Libya	-0.30	0.02	-0.53
Qatar	0.44	0.62	0.29	Sudan	-0.34	0.10	-0.60
Kuwait	0.42	0.54	0.27	Türkiye	-0.37	-0.14	-0.54
Syrian Arab Republic	0.32	0.44	0.22	Bahrain	-0.39	-0.13	-0.54
**Palestine**	**0.31**	**0.39**	**0.20**	**Jordan**	**-0.43**	**-0.23**	**-0.60**

**S5 Table . Age-standardized rate of death (ASDR) due to resistant culprit pathogens of infective endocarditis and other cardiac infections in North America and the Middle East (NAME) and its 21 countries for both sexes in 2019 sorted from largest to smallest**					
	**Country name**	**ASDR**	**Lower bound**	**Upper bound**	**The most common resistant culprit pathogen**
**1**	**Syrian Arab Republic**	**0.68**	**0.35**	**1.1**	**Acinetobacter baumannii**
2	Afghanistan	0.45	0.24	0.7	Klebsiella pneumoniae
3	Egypt	0.27	0.16	0.41	Acinetobacter baumannii
4	Jordan	0.24	0.17	0.35	Acinetobacter baumannii
5	Tunisia	0.22	0.13	0.32	Acinetobacter baumannii
6	saudi Arabia	0.21	0.13	0.33	Staphylococcus aureus
7	Iran (Islamic Republic of)	0.19	0.13	0.27	Acinetobacter baumannii
8	Bahrain	0.18	0.11	0.29	Staphylococcus aureus
9	Türkiye	0.18	0.12	0.25	Staphylococcus aureus
10	Qatar	0.16	0.092	0.26	Staphylococcus aureus
11	Oman	0.16	0.11	0.22	Staphylococcus aureus
12	Morocco	0.14	0.091	0.19	Klebsiella pneumoniae
13	Yemen	0.14	0.085	0.21	Klebsiella pneumoniae
**14**	**North Africa and the Middle East**	**0.13**	**0.09**	**0.18**	**Staphylococcus aureus**
15	Kuwait	0.12	0.08	0.17	Staphylococcus aureus
16	Sudan	0.12	0.076	0.18	Klebsiella pneumoniae
17	Iraq	0.11	0.074	0.16	Staphylococcus aureus
18	Alegria	0.1	0.073	0.14	Staphylococcus aureus
19	United Arab Emirates	0.098	0.062	0.15	Staphylococcus aureus
20	Lebanon	0.083	0.049	0.14	Staphylococcus aureus
21	Palestine	0.083	0.054	0.12	Staphylococcus aureus
**22**	**Libya**	**0.026**	**0.016**	**0.039**	**Staphylococcus aureus**

**S6 Table. Age-standardized rate (ASR) of DALY and percent change in ASR of DALY between 1990 and 2019 in the North Africa and the Middle East (NAME) region and its 21 countries, sorted from largest to smallest**							
**Age-standardized rate of DALY sorted from largest to smallest**				**Percent change in age-standardized rate of DALY sorted from largest to smallest**			
**Location's name**	**value**	**Upper bound**	**Lower bound**	**Location's name**	**value**	**Upper bound**	**Lower bound**
**Egypt**	**25.40**	**36.03**	**13.99**	**Kuwait**	**0.10**	**0.72**	**-0.48**
Jordan	21.65	26.84	16.55	Morocco	0.08	0.60	-0.24
Türkiye	19.61	24.70	14.57	Afghanistan	-0.03	0.30	-0.27
Saudi Arabia	17.64	23.96	10.93	Saudi Arabia	-0.06	0.46	-0.47
**North Africa and the Middle East**	**17.62**	**21.40**	**12.91**	Yemen	-0.11	0.31	-0.41
Oman	17.26	21.53	12.58	Oman	-0.13	0.55	-0.44
Libya	16.85	25.19	10.09	Iraq	-0.14	0.16	-0.35
United Arab Emirates	15.26	21.85	9.91	United Arab Emirates	-0.21	0.12	-0.45
Afghanistan	15.07	21.09	10.48	Tunisia	-0.28	-0.03	-0.49
Sudan	14.83	23.60	9.44	Syrian Arab Republic	-0.29	0.04	-0.50
Morocco	14.64	19.44	10.58	Palestine	-0.32	0.00	-0.52
Iran (Islamic Republic of)	14.41	21.07	12.05	Iran (Islamic Republic of)	-0.33	-0.17	-0.47
Algeria	14.37	18.65	10.28	Lebanon	-0.33	-0.10	-0.51
Yemen	13.98	22.12	8.87	**North Africa and the Middle East**	**-0.34**	**-0.19**	**-0.46**
Iraq	13.38	17.92	9.76	Qatar	-0.35	-0.06	-0.56
Lebanon	13.09	17.12	9.30	Algeria	-0.36	-0.05	-0.55
Tunisia	12.55	17.22	8.91	Egypt	-0.42	-0.04	-0.63
Bahrain	10.33	13.26	7.58	Bahrain	-0.44	-0.18	-0.58
Kuwait	9.92	12.94	6.27	Sudan	-0.44	0.17	-0.69
Qatar	9.09	12.74	6.08	Türkiye	-0.45	-0.27	-0.59
Syrian Arab Republic	8.95	12.43	5.81	Jordan	-0.46	-0.25	-0.60
**Palestine**	**7.73**	**9.90**	**4.95**	**Libya**	**-0.48**	**-0.11**	**-0.69**

S7 Table. Age-standardized rate of DALY due to resistant culprit pathogens of infective endocarditis and other cardiac infections in North America and the Middle East (NAME) and its 21 countries for both sexes in 2019 sorted from largest to smallest

	Country name	ASDR	Lower bound	Upper bound	The most common resistant culprit pathogen
**1**	**Syrian Arab Republics**	**12.7**	**6.6**	**20.8**	Acinetobacter baumannii
2	Egypt	7.8	4.8	12.1	Acinetobacter baumannii
3	Afghanistan	7.5	4.1	12.1	Klebsiella pneumoniae
4	Jordan	5.8	4.2	8.0	Acinetobacter baumannii
5	Lebanon	5.4	3.1	8.6	Staphylococcus aureus
6	Tunisia	5.2	3.1	8.0	Acinetobacter baumannii
7	Saudi Arabia	5.1	3.1	8.0	Staphylococcus aureus
8	Iran (Islamic Republic of)	4.9	3.4	7.0	Acinetobacter baumannii
9	Türkiye	4.1	2.8	5.9	Staphylococcus aureus
10	Yemen	4.0	2.4	6.5	Klebsiella pneumoniae
11	Oman	3.9	2.7	5.7	Staphylococcus aureus
12	Sudan	3.5	2.1	5.7	Klebsiella pneumoniae
13	Libya	3.5	2.1	5.6	Staphylococcus aureus
14	Bahrain	3.4	2.0	3.5	Staphylococcus aureus
**15**	**North Africa and the Middle East**	**3.3**	**2.3**	**4.6**	Staphylococcus aureus
16	Morocco	3.2	2.1	4.6	Klebsiella pneumoniae
17	Qatar	3.1	1.8	5.0	Staphylococcus aureus
18	Kuwait	2.9	1.9	4.4	Staphylococcus aureus
19	Iraq	2.9	1.9	4.5	Staphylococcus aureus
20	Alegria	2.7	1.9	3.9	Staphylococcus aureus
21	United Arab Emirates	2.6	1.6	3.8	Staphylococcus aureus
**22**	**Palestine**	**2.0**	**1.3**	**3.0**	Staphylococcus aureus

S8 Table. Age-standardized rate (ASR) of YLL and percent change in ASR of YLL between 1990 and 2019 in the North Africa and the Middle East (NAME) region and its 21 countries, sorted from largest to smallest

Age-standardized rate of YLL sorted from largest to smallest				Percent change in age-standardized rate of YLL sorted from largest to smallest			
**Location's name**	**value**	**Upper bound**	**Lower bound**	**Location's name**	**value**	**Upper bound**	**Lower bound**
**Egypt**	**25.15**	**35.83**	**13.75**	**Kuwait**	**0.10**	**0.74**	**-0.49**
Jordan	21.24	26.43	16.28	Morocco	0.08	0.61	-0.24
Türkiye	19.16	24.30	14.09	Afghanistan	-0.03	0.30	-0.28
**North Africa and the Middle East**	**17.35**	**21.13**	**12.70**	Saudi Arabia	-0.06	0.47	-0.48
Saudi Arabia	17.34	23.69	10.64	Yemen	-0.11	0.31	-0.42
Oman	17.04	21.30	12.32	Oman	-0.13	0.56	-0.44
Libya	16.65	25.00	9.89	Iraq	-0.14	0.16	-0.36
United Arab Emirates	15.06	21.69	9.70	United Arab Emirates	-0.21	0.12	-0.45
Afghanistan	14.97	20.98	10.40	Tunisia	-0.29	-0.03	-0.50
Sudan	14.70	23.47	9.31	Syrian Arab Republic	-0.29	0.03	-0.50
Morocco	14.47	19.26	10.43	Palestine	-0.32	-0.01	-0.53
Algeria	14.19	18.46	10.11	Iran (Islamic Republic of)	-0.33	-0.18	-0.47
Iran (Islamic Republic of)	14.14	20.81	11.81	Lebanon	-0.34	-0.11	-0.52
Yemen	13.86	22.00	8.74	**North Africa and the Middle East**	**-0.34**	**-0.20**	**-0.47**
Iraq	13.17	17.71	9.54	Qatar	-0.36	-0.06	-0.57
Lebanon	12.84	16.88	9.05	Algeria	-0.36	-0.05	-0.55
Tunisia	12.32	16.98	8.65	Egypt	-0.42	-0.04	-0.63
Bahrain	10.05	13.00	7.32	Sudan	-0.44	0.17	-0.69
Kuwait	9.66	12.71	6.04	Bahrain	-0.44	-0.19	-0.59
Qatar	8.82	12.46	5.83	Türkiye	-0.46	-0.28	-0.60
Syrian Arab Republic	8.81	12.32	5.67	Jordan	-0.46	-0.26	-0.61
**Palestine**	**7.58**	**9.78**	**4.79**	**Libya**	**-0.49**	**-0.11**	**-0.70**

S9 Table. Age-standardized rate (ASR) of YLD and percent change in ASR of YLD between 1990 and 2019 in North Africa and the Middle East (NAME) region and its 21 countries, sorted from largest to smallest							
**Age-standardized rate of YLD sorted from largest to smallest**				**Percent change (PC) in the age-standardized rate of YLD sorted from largest to smallest**			
**Location's name**	**ASR of YLD**	**Upper bound**	**Lower bound**	**Location's name**	**PC in ASR of YLD**	**Upper bound**	**Lower bound**
**Türkiye**	**0.45**	**0.64**	**0.30**	**Oman**	**0.42**	**0.55**	**0.31**
Jordan	0.41	0.59	0.27	Syrian Arab Republic	0.24	0.31	0.17
Saudi Arabia	0.30	0.43	0.20	Libya	0.21	0.30	0.13
Bahrain	0.28	0.39	0.18	Sudan	0.21	0.28	0.13
Kuwait	0.27	0.38	0.18	Saudi Arabia	0.21	0.35	0.08
**North Africa and the Middle East**	**0.27**	**0.37**	**0.18**	Egypt	**0.20**	**0.34**	**0.08**
Qatar	0.27	0.39	0.18	Afghanistan	0.20	0.28	0.12
Iran (Islamic Republic of)	0.26	0.38	0.18	Yemen	0.19	0.27	0.12
Lebanon	0.25	0.36	0.16	Tunisia	0.19	0.28	0.10
Egypt	0.25	0.36	0.16	Lebanon	0.18	0.26	0.10
Tunisia	0.22	0.32	0.15	Morocco	0.17	0.26	0.07
Oman	0.22	0.31	0.14	Palestine	0.15	0.22	0.08
Iraq	0.21	0.30	0.14	Algeria	0.11	0.19	0.04
United Arab Emirates	0.20	0.29	0.13	Qatar	0.11	0.30	-0.05
Libya	0.20	0.28	0.13	Iran (Islamic Republic of)	0.11	0.15	0.07
Algeria	0.19	0.26	0.12	Iraq	0.10	0.18	0.02
Morocco	0.16	0.23	0.11	Kuwait	0.09	0.19	0.00
Palestine	0.15	0.21	0.10	**North Africa and the Middle East**	**0.09**	**0.20**	**-0.02**
Syrian Arab Republic	0.14	0.20	0.09	United Arab Emirates	0.07	0.15	0.01
Sudan	0.13	0.18	0.08	Bahrain	0.07	0.19	-0.05
Yemen	0.12	0.16	0.07	Jordan	0.06	0.16	-0.03
**Afghanistan**	**0.10**	**0.14**	**0.06**	**Türkiye**	**-0.06**	**0.20**	**-0.25**

S10 Table. Age-standardized rates of incidence, prevalence, deaths, DALYs, YLLs, and YLDs of infective endocarditis in North Africa and the Middle East in 1990, 1997, 2007 and 2019

	1990			1997			2007			2019		
	Point estimate	95% uncertainty interval		Point estimate	95% uncertainty interval		Point estimate	95% uncertainty interval		Point estimate	95% uncertainty interval	
		Lower	Upper		Lower	Upper		Lower	Upper		Lower	Upper
**Incidence**	11.5	9.6	13.6	12.1	10.3	14.2	13.5	11.4	15.8	15.4	12.9	18.2
**Prevalence**	3.1	2.6	3.7	3.2	2.4	3.8	3.5	3.1	4	3.4	3.1	4
**Deaths**	0.8	0.6	1	0.8	0.6	1	0.7	0.5	0.9	0.7	0.5	0.8
**DALYs**	26.7	18.8	33	24.1	17.6	28.4	20.5	14.5	24.4	17.6	12.9	21.4
**YLLs**	23.8	17.4	28.1	26.4	18.5	32.7	20.2	14.3	24.1	17.4	12.7	21.1
**YLDs**	0.25	0.17	0.35	0.25	0.17	0.36	0.27	0.19	0.38	0.27	0.18	0.37

S11 Table. Age-standardized incidence, prevalence, deaths, Disability-Adjusted Life Years (DALYs), Years of Life Lost (YLLs) and Years Lived with Disability (YLDs) rates and number in twenty-one countries of North Africa and the Middle East in1990, 1997, 2007, and 2019 according to sex

Country	Measure	Sex	1990		1997		2007		2019	
			Rate†	Number‡	Rate	Number	Rate	Number	Rate	Number
Afghanistan	Incidence	Both	7.7 (6.2 to 9.4)	843 (659 to 1058)	7.2 (5.9 to 8.7)	1305 (1012 to 1665)	8 (6.6 to 9.7)	1928 (1471 to 2512)	10.3 (8.4 to 12.6)	3589 (2738 to 4654)
		Female	6.9 (5.6 to 8.4)	380 (297 to 480)	6.4 (5.3 to 7.8)	573 (445 to 730)	7.2 (5.9 to 8.7)	847 (645 to 1121)	9.3 (7.6 to 11.4)	1579 (1207 to 2063)
		Male	8.5 (6.8 to 10.4)	463 (358 to 586)	8 (6.6 to 9.7)	732 (569 to 934)	8.9 (7.2 to 10.7)	1081 (823 to 1410)	11.3 (9.1 to 13.8)	2011 (1529 to 2603)
	Prevalence	Both	1.1 (0.9 to 1.3)	88 (72 to 105)	1 (0.9 to 1.2)	124 (103 to 149)	1.1 (0.9 to 1.3)	174 (142 to 215)	1.3 (1.1 to 1.5)	306 (247 to 380)
		Female	0.9 (0.8 to 1.1)	36 (30 to 44)	0.9 (0.7 to 1)	51 (42 to 62)	0.9 (0.8 to 1.1)	73 (59 to 91)	1.1 (0.9 to 1.3)	128 (103 to 160)
		Male	1.2 (1 to 1.4)	51 (42 to 62)	1.2 (1 to 1.4)	73 (61 to 88)	1.3 (1.1 to 1.5)	102 (82 to 125)	1.5 (1.3 to 1.8)	178 (144 to 220)
	Deaths	Both	0.5 (0.4 to 0.8)	38 (26 to 53)	0.5 (0.4 to 0.8)	49 (34 to 67)	0.6 (0.4 to 0.8)	60 (42 to 82)	0.5 (0.4 to 0.8)	81 (56 to 115)
		Female	0.5 (0.3 to 0.7)	18 (11 to 26)	0.5 (0.3 to 0.8)	23 (15 to 35)	0.5 (0.3 to 0.8)	28 (18 to 41)	0.6 (0.3 to 0.8)	40 (25 to 63)
		Male	0.6 (0.4 to 0.9)	21 (13 to 31)	0.6 (0.4 to 0.9)	26 (17 to 39)	0.6 (0.4 to 0.9)	31 (21 to 47)	0.5 (0.3 to 0.8)	41 (27 to 62)
	DALYs	Both	15.5 (10.9 to 21.3)	1397 (958 to 1984)	15.8 (11.1 to 22)	1960 (1306 to 2828)	16 (11.1 to 22.3)	2439 (1604 to 3482)	15.1 (10.5 to 21.1)	3304 (2218 to 4776)

S11 Table. Age-standardized incidence, prevalence, deaths, Disability-Adjusted Life Years (DALYs), Years of Life Lost (YLLs) and Years Lived with Disability (YLDs) rates and number in twenty-one countries of North Africa and the Middle East in 1990, 1997, 2007, and 2019 according to sex

Country	Measure	Sex	1990		1997		2007		2019	
			Rate†	Number‡	Rate	Number	Rate	Number	Rate	Number
		Female	14.6 (9.7 to 22.8)	677 (387 to 1117)	14.9 (9.7 to 23)	948 (514 to 1595)	15.1 (9.9 to 22.8)	1147 (618 to 1820)	14.8 (9.5 to 23.2)	1555 (900 to 2577)
		Male	16.5 (10.9 to 23.8)	721 (476 to 1032)	16.7 (11.2 to 24.4)	1012 (668 to 1478)	16.9 (11.1 to 25.1)	1292 (803 to 1952)	15.4 (10.1 to 22.8)	1749 (1112 to 2651)
	YLLs	Both	15.5 (10.8 to 21.2)	1391 (949 to 1977)	15.7 (11 to 22)	1952 (1299 to 2821)	15.9 (11.1 to 22.2)	2428 (1592 to 3471)	15 (10.4 to 21)	3285 (2191 to 4758)
		Female	14.6 (9.6 to 22.7)	674 (385 to 1115)	14.9 (9.6 to 22.9)	945 (510 to 1589)	15 (9.8 to 22.7)	1142 (613 to 1814)	14.7 (9.5 to 23.2)	1547 (890 to 2569)
		Male	16.4 (10.8 to 23.7)	717 (472 to 1028)	16.6 (11.1 to 24.4)	1007 (663 to 1473)	16.8 (11 to 25)	1286 (798 to 1946)	15.2 (10 to 22.7)	1738 (1100 to 2641)
	YLDs	Both	0.1 (0.1 to 0.1)	6 (4 to 9)	0.1 (0.1 to 0.1)	8 (5 to 12)	0.1 (0.1 to 0.1)	12 (7 to 17)	0.1 (0.1 to 0.1)	20 (12 to 29)
		Female	0.1 (0 to 0.1)	2 (2 to 4)	0.1 (0 to 0.1)	3 (2 to 5)	0.1 (0 to 0.1)	5 (3 to 7)	0.1 (0.1 to 0.1)	8 (5 to 12)
		Male	0.1 (0.1 to 0.1)	4 (2 to 5)	0.1 (0.1 to 0.1)	5 (3 to 7)	0.1 (0.1 to 0.1)	7 (4 to 10)	0.1 (0.1 to 0.2)	12 (7 to 17)
**Algeria**	Incidence	Both	10.4 (8.6 to 12.5)	2195 (1757 to 2695)	11 (9.2 to 13.1)	2637 (2152 to 3194)	11.9 (10 to 14.2)	3442 (2809 to 4122)	13.7 (11.4 to 16.4)	5309 (4303 to 6426)
		Female	9.6 (7.9 to 11.6)	1002 (800 to 1234)	10.2 (8.6 to 12)	1197 (978 to 1455)	11.2 (9.4 to 13.2)	1557 (1275 to 1860)	12.8 (10.6 to 15.3)	2395 (1947 to 2880)

S11 Table. Age-standardized incidence, prevalence, deaths, Disability-Adjusted Life Years (DALYs), Years of Life Lost (YLLs) and Years Lived with Disability (YLDs) rates and number in twenty-one countries of North Africa and the Middle East in 1990, 1997, 2007, and 2019 according to sex

Country	Measure	Sex	1990		1997		2007		2019	
			Rate†	Number‡	Rate	Number	Rate	Number	Rate	Number
		Male	11.3 (9.2 to 13.5)	1193 (949 to 1450)	11.8 (9.8 to 14.2)	1441 (1165 to 1753)	12.7 (10.5 to 15.1)	1885 (1524 to 2275)	14.6 (12 to 17.7)	2914 (2362 to 3549)
	Prevalence	Both	2.1 (1.8 to 2.5)	328 (279 to 385)	2.2 (1.9 to 2.6)	408 (351 to 475)	2.2 (1.9 to 2.6)	537 (461 to 626)	2.5 (2.1 to 2.9)	849 (732 to 989)
		Female	2 (1.7 to 2.4)	151 (128 to 179)	2.1 (1.8 to 2.4)	187 (160 to 219)	2.1 (1.8 to 2.5)	244 (208 to 286)	2.3 (2 to 2.7)	382 (328 to 449)
		Male	2.3 (1.9 to 2.7)	177 (150 to 208)	2.4 (2 to 2.8)	221 (189 to 258)	2.4 (2 to 2.8)	293 (252 to 341)	2.6 (2.2 to 3.1)	467 (398 to 548)
	Deaths	Both	0.8 (0.6 to 1)	100 (65 to 135)	0.7 (0.5 to 0.9)	107 (76 to 143)	0.6 (0.5 to 0.8)	129 (95 to 168)	0.6 (0.4 to 0.7)	173 (127 to 225)
		Female	0.8 (0.5 to 1.2)	50 (29 to 72)	0.7 (0.5 to 1)	54 (33 to 76)	0.7 (0.5 to 0.9)	65 (44 to 89)	0.7 (0.4 to 0.9)	89 (58 to 122)
		Male	0.8 (0.5 to 1.1)	50 (31 to 75)	0.7 (0.5 to 0.9)	54 (36 to 76)	0.6 (0.4 to 0.8)	64 (44 to 91)	0.5 (0.4 to 0.7)	84 (58 to 119)
	DALYs	Both	22.4 (13.8 to 30.8)	4526 (2217 to 6581)	19.3 (13.1 to 25.8)	4401 (2570 to 6001)	16.8 (12 to 22.1)	4695 (3233 to 6300)	14.4 (10.3 to 18.7)	5387 (3861 to 7145)
		Female	22.8 (12.1 to 33)	2285 (932 to 3590)	19.4 (11.6 to 27.9)	2184 (1099 to 3287)	17.1 (10.8 to 23.6)	2279 (1356 to 3199)	14.9 (9.6 to 19.9)	2622 (1685 to 3574)
		Male	22.2 (13.5 to 34.1)	2241 (1115 to 3694)	19.2 (12.3 to 27.6)	2217 (1286 to 3348)	16.6 (11.2 to 23.7)	2416 (1578 to 3494)	14.1 (9.5 to 20.4)	2765 (1842 to 4036)

S11 Table. Age-standardized incidence, prevalence, deaths, Disability-Adjusted Life Years (DALYs), Years of Life Lost (YLLs) and Years Lived with Disability (YLDs) rates and number in twenty-one countries of North Africa and the Middle East in 1990, 1997, 2007, and 2019 according to sex

Country	Measure	Sex	1990		1997		2007		2019	
			Rate†	Number‡	Rate	Number	Rate	Number	Rate	Number
	YLLs	Both	22.2 (13.6 to 30.6)	4502 (2194 to 6560)	19.1 (12.9 to 25.6)	4370 (2536 to 5974)	16.6 (11.8 to 21.9)	4655 (3204 to 6258)	14.2 (10.1 to 18.5)	5323 (3800 to 7088)
		Female	22.6 (12 to 32.8)	2274 (921 to 3579)	19.3 (11.4 to 27.8)	2170 (1083 to 3274)	17 (10.6 to 23.4)	2261 (1337 to 3179)	14.8 (9.3 to 19.7)	2593 (1649 to 3549)
		Male	22 (13.3 to 33.9)	2228 (1101 to 3684)	19 (12.1 to 27.4)	2200 (1266 to 3332)	16.4 (11.1 to 23.5)	2394 (1556 to 3473)	13.9 (9.3 to 20.2)	2730 (1811 to 3996)
	YLDs	Both	0.2 (0.1 to 0.2)	24 (16 to 35)	0.2 (0.1 to 0.2)	31 (20 to 43)	0.2 (0.1 to 0.2)	40 (26 to 57)	0.2 (0.1 to 0.3)	63 (42 to 89)
		Female	0.2 (0.1 to 0.2)	11 (7 to 16)	0.2 (0.1 to 0.2)	14 (9 to 20)	0.2 (0.1 to 0.2)	18 (12 to 26)	0.2 (0.1 to 0.2)	29 (19 to 40)
		Male	0.2 (0.1 to 0.3)	13 (8 to 19)	0.2 (0.1 to 0.3)	16 (11 to 23)	0.2 (0.1 to 0.3)	22 (14 to 31)	0.2 (0.1 to 0.3)	35 (23 to 49)
**Bahrain**	Incidence	Both	12.2 (10.2 to 14.7)	46 (36 to 57)	12.5 (10.4 to 14.9)	57 (45 to 70)	14.1 (11.6 to 16.9)	108 (84 to 136)	15.5 (12.8 to 18.7)	204 (158 to 262)
		Female	11 (9.1 to 13.2)	17 (14 to 21)	11.2 (9.3 to 13.2)	22 (17 to 26)	12.4 (10.3 to 14.8)	37 (29 to 46)	13.7 (11.4 to 16.6)	64 (50 to 81)
		Male	13.1 (10.8 to 15.8)	29 (22 to 36)	13.4 (11.1 to 16)	35 (27 to 44)	15.2 (12.6 to 18.1)	72 (55 to 90)	16.6 (13.6 to 19.9)	140 (106 to 180)
	Prevalence	Both	3.3 (2.8 to 3.9)	8 (6 to 9)	3.3 (2.8 to 4)	10 (8 to 11)	3.4 (2.9 to 4.1)	17 (15 to 20)	3.6 (3.1 to 4.3)	37 (31 to 45)

S11 Table. Age-standardized incidence, prevalence, deaths, Disability-Adjusted Life Years (DALYs), Years of Life Lost (YLLs) and Years Lived with Disability (YLDs) rates and number in twenty-one countries of North Africa and the Middle East in 1990, 1997, 2007, and 2019 according to sex

Country	Measure	Sex	1990		1997		2007		2019	
			Rate†	Number‡	Rate	Number	Rate	Number	Rate	Number
		Female	2.8 (2.4 to 3.4)	3 (2 to 3)	2.9 (2.4 to 3.4)	4 (3 to 4)	2.9 (2.5 to 3.5)	6 (5 to 7)	3 (2.6 to 3.6)	12 (10 to 14)
		Male	3.7 (3.1 to 4.5)	5 (4 to 6)	3.8 (3.1 to 4.6)	6 (5 to 7)	3.9 (3.2 to 4.6)	11 (9 to 13)	4 (3.4 to 4.9)	25 (21 to 31)
	Deaths	Both	0.8 (0.5 to 1)	1 (1 to 2)	0.8 (0.5 to 1)	2 (1 to 2)	0.7 (0.4 to 0.8)	2 (2 to 3)	0.5 (0.4 to 0.6)	4 (3 to 5)
		Female	0.8 (0.4 to 1)	1 (0 to 1)	0.8 (0.4 to 1)	1 (0 to 1)	0.6 (0.4 to 0.9)	1 (1 to 1)	0.4 (0.3 to 0.7)	1 (1 to 2)
		Male	0.8 (0.5 to 1.1)	1 (1 to 1)	0.8 (0.5 to 1)	1 (1 to 1)	0.7 (0.4 to 0.9)	1 (1 to 2)	0.5 (0.3 to 0.7)	2 (2 to 3)
	DALYs	Both	18.3 (12.8 to 22.1)	51 (39 to 63)	17.8 (12 to 21.1)	60 (46 to 73)	13.7 (10.7 to 16.7)	80 (65 to 102)	10.3 (7.6 to 13.3)	124 (86 to 171)
		Female	18.1 (10.3 to 22.8)	22 (13 to 28)	17.9 (9.5 to 22.8)	25 (14 to 32)	12.8 (8.6 to 16.8)	27 (19 to 37)	9 (6.5 to 13.7)	36 (26 to 58)
		Male	18.5 (13.4 to 23.8)	30 (22 to 41)	17.6 (12.5 to 22.8)	35 (27 to 49)	14.3 (10.3 to 17.7)	53 (41 to 72)	11.2 (7.5 to 14.9)	88 (56 to 124)
	YLLs	Both	18.1 (12.5 to 21.9)	51 (39 to 63)	17.5 (11.7 to 20.9)	59 (45 to 72)	13.4 (10.4 to 16.5)	79 (64 to 101)	10 (7.3 to 13)	121 (84 to 168)
		Female	17.9 (10.1 to 22.6)	21 (12 to 28)	17.7 (9.3 to 22.6)	25 (14 to 32)	12.5 (8.4 to 16.6)	27 (19 to 37)	8.7 (6.3 to 13.4)	35 (25 to 56)

S11 Table. Age-standardized incidence, prevalence, deaths, Disability-Adjusted Life Years (DALYs), Years of Life Lost (YLLs) and Years Lived with Disability (YLDs) rates and number in twenty-one countries of North Africa and the Middle East in 1990, 1997, 2007, and 2019 according to sex

Country	Measure	Sex	1990		1997		2007		2019	
			Rate†	Number‡	Rate	Number	Rate	Number	Rate	Number
		Male	18.2 (13.1 to 23.6)	29 (22 to 41)	17.3 (12.2 to 22.5)	34 (27 to 48)	14 (10 to 17.5)	52 (40 to 71)	10.9 (7.2 to 14.5)	86 (54 to 122)
	YLDs	Both	0.3 (0.2 to 0.4)	1 (0 to 1)	0.3 (0.2 to 0.4)	1 (0 to 1)	0.3 (0.2 to 0.4)	1 (1 to 2)	0.3 (0.2 to 0.4)	3 (2 to 4)
		Female	0.2 (0.1 to 0.3)	0 (0 to 0)	0.2 (0.1 to 0.3)	0 (0 to 0)	0.2 (0.1 to 0.3)	0 (0 to 1)	0.2 (0.2 to 0.3)	1 (1 to 1)
		Male	0.3 (0.2 to 0.4)	0 (0 to 1)	0.3 (0.2 to 0.4)	0 (0 to 1)	0.3 (0.2 to 0.4)	1 (1 to 1)	0.3 (0.2 to 0.4)	2 (1 to 3)
Egypt	Incidence	Both	10.8 (9 to 12.9)	5523 (4443 to 6741)	11.9 (10.1 to 13.9)	6714 (5543 to 8061)	13.4 (11.4 to 15.6)	8977 (7465 to 10725)	15 (12.6 to 17.6)	12702 (10465 to 15136)
		Female	9.7 (8 to 11.5)	2468 (1990 to 3019)	10.7 (9 to 12.6)	2979 (2444 to 3600)	12.1 (10.3 to 14.1)	3919 (3216 to 4719)	13.6 (11.4 to 16.1)	5395 (4355 to 6553)
		Male	12 (10 to 14.3)	3056 (2445 to 3740)	13.1 (11.1 to 15.3)	3735 (3074 to 4496)	14.5 (12.3 to 17)	5059 (4213 to 6053)	16.3 (13.8 to 19.1)	7306 (6052 to 8736)
	Prevalence	Both	2.5 (2 to 3.2)	954 (781 to 1167)	2.6 (2.1 to 3.3)	1140 (939 to 1386)	2.8 (2.3 to 3.5)	1529 (1259 to 1851)	3.1 (2.5 to 3.9)	2236 (1856 to 2745)
		Female	2.4 (1.9 to 3.1)	451 (367 to 556)	2.5 (2 to 3.1)	529 (430 to 642)	2.7 (2.2 to 3.5)	695 (566 to 851)	3 (2.4 to 3.9)	984 (804 to 1207)
		Male	2.6 (2.1 to 3.4)	503 (407 to 620)	2.7 (2.2 to 3.5)	611 (498 to 745)	2.9 (2.4 to 3.7)	835 (682 to 1008)	3.2 (2.6 to 4.1)	1252 (1028 to 1541)

S11 Table. Age-standardized incidence, prevalence, deaths, Disability-Adjusted Life Years (DALYs), Years of Life Lost (YLLs) and Years Lived with Disability (YLDs) rates and number in twenty-one countries of North Africa and the Middle East in 1990, 1997, 2007, and 2019 according to sex

Country	Measure	Sex	1990		1997		2007		2019	
			Rate†	Number‡	Rate	Number	Rate	Number	Rate	Number
	Deaths	Both	1 (0.6 to 1.3)	450 (192 to 708)	0.9 (0.5 to 1.2)	422 (216 to 594)	0.9 (0.5 to 1.2)	469 (248 to 627)	0.8 (0.5 to 1.1)	511 (292 to 733)
		Female	1 (0.5 to 1.5)	246 (80 to 439)	1 (0.5 to 1.4)	224 (89 to 355)	1 (0.5 to 1.4)	235 (104 to 352)	0.9 (0.5 to 1.4)	241 (126 to 363)
		Male	0.9 (0.5 to 1.2)	204 (97 to 333)	0.8 (0.5 to 1.1)	197 (110 to 281)	0.8 (0.5 to 1.1)	235 (131 to 323)	0.7 (0.4 to 1)	270 (145 to 387)
	DALYs	Both	43.5 (17.9 to 69.1)	29151 (8871 to 51528)	36.7 (17.6 to 54)	24201 (9707 to 38773)	31.6 (15.9 to 43.9)	23710 (10994 to 35133)	25.4 (14 to 36)	21938 (11858 to 31812)
		Female	48.4 (14.6 to 87.3)	16368 (3530 to 32756)	40.4 (14.4 to 67.4)	13122 (3782 to 24139)	33.4 (14 to 51.8)	11830 (4385 to 19849)	25.8 (13 to 39.4)	10013 (5014 to 15641)
		Male	38.8 (18.3 to 63.5)	12783 (4697 to 24016)	33.4 (17.6 to 50)	11079 (5187 to 18155)	30.4 (16.3 to 44.8)	11880 (5885 to 18920)	25.7 (13.3 to 38.9)	11925 (6105 to 18505)
	YLLs	Both	43.3 (17.7 to 68.9)	29077 (8823 to 51463)	36.5 (17.4 to 53.8)	24113 (9640 to 38687)	31.4 (15.7 to 43.7)	23593 (10914 to 35017)	25.2 (13.7 to 35.8)	21766 (11695 to 31616)
		Female	48.2 (14.4 to 87.1)	16332 (3492 to 32713)	40.2 (14.2 to 67.2)	13081 (3745 to 24082)	33.2 (13.8 to 51.6)	11776 (4327 to 19795)	25.6 (12.8 to 39)	9936 (4932 to 15604)
		Male	38.6 (18.1 to 63.3)	12745 (4660 to 23981)	33.2 (17.4 to 49.7)	11032 (5147 to 18110)	30.2 (16 to 44.6)	11817 (5817 to 18863)	25.4 (13 to 38.8)	11830 (6034 to 18386)
	YLDs	Both	0.2 (0.1 to 0.3)	74 (48 to 107)	0.2 (0.1 to 0.3)	88 (58 to 127)	0.2 (0.1 to 0.3)	118 (77 to 171)	0.2 (0.2 to 0.4)	172 (112 to 250)

S11 Table. Age-standardized incidence, prevalence, deaths, Disability-Adjusted Life Years (DALYs), Years of Life Lost (YLLs) and Years Lived with Disability (YLDs) rates and number in twenty-one countries of North Africa and the Middle East in 1990, 1997, 2007, and 2019 according to sex

Country	Measure	Sex	1990		1997		2007		2019	
			Rate†	Number‡	Rate	Number	Rate	Number	Rate	Number
		Female	0.2 (0.1 to 0.3)	36 (22 to 52)	0.2 (0.1 to 0.3)	41 (27 to 59)	0.2 (0.1 to 0.3)	54 (35 to 78)	0.2 (0.2 to 0.4)	76 (49 to 113)
		Male	0.2 (0.1 to 0.3)	39 (25 to 56)	0.2 (0.1 to 0.3)	47 (31 to 67)	0.2 (0.1 to 0.3)	63 (42 to 93)	0.3 (0.2 to 0.4)	95 (61 to 141)
Iran (Islamic Republic of)	Incidence	Both	11.9 (9.7 to 14.4)	6069 (4825 to 7553)	13.1 (10.9 to 15.5)	6915 (5609 to 8317)	14.9 (12.5 to 17.7)	9194 (7543 to 10992)	16.5 (13.7 to 19.8)	12992 (10615 to 15748)
		Female	10.9 (9 to 13.3)	2801 (2212 to 3503)	12.2 (10.2 to 14.5)	3168 (2564 to 3828)	14 (11.6 to 16.6)	4119 (3368 to 4934)	15.7 (12.9 to 18.8)	5974 (4866 to 7272)
		Male	12.7 (10.4 to 15.4)	3268 (2605 to 4051)	13.8 (11.6 to 16.4)	3747 (3046 to 4493)	15.9 (13.3 to 18.9)	5075 (4171 to 6059)	17.4 (14.4 to 20.8)	7018 (5738 to 8523)
	Prevalence	Both	3 (2.5 to 3.6)	1011 (847 to 1201)	3.1 (2.6 to 3.7)	1223 (1037 to 1445)	3.2 (2.7 to 3.8)	1662 (1418 to 1967)	3.4 (2.9 to 4.1)	2504 (2125 to 2983)
		Female	2.8 (2.3 to 3.4)	467 (391 to 552)	2.9 (2.5 to 3.5)	553 (467 to 653)	3 (2.5 to 3.6)	744 (632 to 886)	3.2 (2.7 to 3.8)	1150 (971 to 1372)
		Male	3.2 (2.7 to 3.9)	544 (457 to 647)	3.3 (2.8 to 4)	670 (566 to 793)	3.4 (2.9 to 4.1)	919 (784 to 1084)	3.7 (3.1 to 4.4)	1355 (1142 to 1620)
	Deaths	Both	0.7 (0.5 to 1.1)	214 (176 to 292)	0.7 (0.5 to 1)	233 (188 to 341)	0.7 (0.6 to 0.9)	326 (274 to 433)	0.6 (0.5 to 0.9)	384 (322 to 596)
		Female	0.7 (0.5 to 1.3)	101 (73 to 148)	0.7 (0.5 to 1.1)	109 (86 to 166)	0.7 (0.5 to 1)	146 (107 to 217)	0.5 (0.4 to 0.9)	179 (139 to 315)

S11 Table. Age-standardized incidence, prevalence, deaths, Disability-Adjusted Life Years (DALYs), Years of Life Lost (YLLs) and Years Lived with Disability (YLDs) rates and number in twenty-one countries of North Africa and the Middle East in 1990, 1997, 2007, and 2019 according to sex

Country	Measure	Sex	1990		1997		2007		2019	
			Rate†	Number‡	Rate	Number	Rate	Number	Rate	Number
		Male	0.7 (0.5 to 1.1)	112 (88 to 166)	0.7 (0.5 to 1)	124 (98 to 190)	0.7 (0.5 to 0.9)	179 (140 to 236)	0.6 (0.4 to 0.9)	205 (159 to 314)
	DALYs	Both	21.4 (17.7 to 29.2)	10692 (7615 to 14294)	20.2 (16.4 to 28.4)	10235 (8163 to 13750)	19.3 (15.6 to 23.8)	11840 (9523 to 14417)	14.4 (12 to 21.1)	11411 (9593 to 16793)
		Female	20.5 (15 to 29.6)	5266 (2877 to 7665)	19.7 (15.2 to 28.1)	4977 (3202 to 6583)	17.7 (12.4 to 23.9)	5122 (3609 to 6550)	12.7 (10 to 21.3)	4784 (3832 to 8156)
		Male	22.2 (17.4 to 33)	5425 (3944 to 7818)	20.5 (16.2 to 30.8)	5257 (4117 to 7894)	20.9 (15.8 to 26.1)	6718 (5186 to 8387)	16.1 (12.6 to 23.1)	6627 (5329 to 9588)
	YLLs	Both	21.2 (17.4 to 29)	10615 (7524 to 14230)	19.9 (16.2 to 28.1)	10142 (8051 to 13650)	19.1 (15.4 to 23.5)	11713 (9396 to 14266)	14.1 (11.8 to 20.8)	11218 (9410 to 16577)
		Female	20.3 (14.8 to 29.4)	5231 (2846 to 7631)	19.5 (14.8 to 27.9)	4935 (3155 to 6551)	17.5 (12.2 to 23.7)	5065 (3553 to 6505)	12.5 (9.7 to 21.1)	4695 (3732 to 8073)
		Male	22 (17.2 to 32.8)	5384 (3909 to 7785)	20.2 (16 to 30.6)	5206 (4049 to 7841)	20.7 (15.6 to 25.9)	6648 (5136 to 8337)	15.8 (12.2 to 22.8)	6523 (5212 to 9470)
	YLDs	Both	0.2 (0.2 to 0.3)	77 (51 to 110)	0.2 (0.2 to 0.4)	93 (62 to 133)	0.2 (0.2 to 0.4)	127 (85 to 181)	0.3 (0.2 to 0.4)	192 (129 to 277)
		Female	0.2 (0.1 to 0.3)	35 (24 to 50)	0.2 (0.2 to 0.3)	42 (28 to 60)	0.2 (0.2 to 0.3)	57 (38 to 82)	0.2 (0.2 to 0.3)	88 (59 to 127)
		Male	0.3 (0.2 to 0.4)	41 (27 to 59)	0.3 (0.2 to 0.4)	51 (34 to 73)	0.3 (0.2 to 0.4)	70 (47 to 100)	0.3 (0.2 to 0.4)	104 (70 to 149)

S11 Table. Age-standardized incidence, prevalence, deaths, Disability-Adjusted Life Years (DALYs), Years of Life Lost (YLLs) and Years Lived with Disability (YLDs) rates and number in twenty-one countries of North Africa and the Middle East in 1990, 1997, 2007, and 2019 according to sex

Country	Measure	Sex	1990		1997		2007		2019	
			Rate†	Number‡	Rate	Number	Rate	Number	Rate	Number
Iraq	Incidence	Both	10.2 (8.4 to 12.3)	1587 (1242 to 2011)	10.4 (8.6 to 12.3)	2196 (1754 to 2720)	11.6 (9.7 to 13.8)	3052 (2446 to 3757)	13.6 (11.2 to 16.1)	4807 (3878 to 5830)
		Female	9.3 (7.6 to 11.1)	720 (563 to 918)	9.5 (7.9 to 11.4)	998 (806 to 1252)	10.7 (8.8 to 12.7)	1379 (1106 to 1711)	12.6 (10.3 to 15)	2164 (1735 to 2639)
		Male	11.1 (9.1 to 13.5)	866 (674 to 1088)	11.2 (9.3 to 13.4)	1197 (949 to 1468)	12.5 (10.4 to 15)	1674 (1333 to 2046)	14.5 (12 to 17.3)	2643 (2121 to 3213)
	Prevalence	Both	2.4 (2 to 2.8)	238 (203 to 282)	2.4 (2 to 2.8)	314 (267 to 366)	2.4 (2.1 to 2.8)	434 (369 to 507)	2.7 (2.3 to 3.2)	713 (606 to 832)
		Female	2.2 (1.9 to 2.6)	112 (95 to 132)	2.2 (1.9 to 2.6)	146 (125 to 170)	2.2 (1.9 to 2.6)	201 (171 to 236)	2.5 (2.1 to 2.9)	329 (278 to 387)
		Male	2.5 (2.1 to 3)	126 (107 to 149)	2.5 (2.1 to 3)	168 (142 to 197)	2.6 (2.2 to 3.1)	233 (198 to 273)	2.9 (2.5 to 3.4)	384 (327 to 449)
	Deaths	Both	0.5 (0.4 to 0.7)	50 (37 to 65)	0.6 (0.4 to 0.8)	71 (52 to 94)	0.6 (0.4 to 0.7)	91 (65 to 120)	0.5 (0.4 to 0.7)	120 (88 to 159)
		Female	0.5 (0.3 to 0.7)	24 (15 to 34)	0.6 (0.4 to 0.8)	34 (22 to 47)	0.5 (0.4 to 0.7)	43 (27 to 59)	0.5 (0.3 to 0.7)	54 (37 to 77)
		Male	0.6 (0.4 to 0.8)	26 (18 to 36)	0.6 (0.4 to 0.9)	37 (26 to 54)	0.6 (0.4 to 0.8)	49 (32 to 67)	0.6 (0.4 to 0.8)	66 (45 to 89)
	DALYs	Both	15.6 (11.8 to 20.3)	2127 (1410 to 2987)	17.3 (12.6 to 23.7)	3063 (1893 to 4215)	16.4 (11.5 to 21.8)	3674 (2370 to 4871)	13.4 (9.8 to 17.9)	4053 (2869 to 5561)

S11 Table. Age-standardized incidence, prevalence, deaths, Disability-Adjusted Life Years (DALYs), Years of Life Lost (YLLs) and Years Lived with Disability (YLDs) rates and number in twenty-one countries of North Africa and the Middle East in 1990, 1997, 2007, and 2019 according to sex

Country	Measure	Sex	1990		1997		2007		2019	
			Rate†	Number‡	Rate	Number	Rate	Number	Rate	Number
		Female	14.4 (9.5 to 19.7)	1017 (530 to 1618)	16.1 (10.4 to 22.6)	1467 (728 to 2181)	15.1 (9.7 to 20.9)	1705 (970 to 2394)	11.7 (8.1 to 16.8)	1740 (1177 to 2510)
		Male	16.7 (12 to 23.5)	1110 (730 to 1657)	18.5 (12.8 to 27.8)	1596 (981 to 2393)	17.7 (11.7 to 24.6)	1969 (1183 to 2827)	15.1 (10.2 to 20.3)	2313 (1540 to 3254)
	YLLs	Both	15.4 (11.6 to 20.1)	2109 (1397 to 2971)	17.1 (12.5 to 23.4)	3040 (1869 to 4196)	16.2 (11.3 to 21.7)	3643 (2333 to 4839)	13.2 (9.5 to 17.7)	4001 (2796 to 5517)
		Female	14.3 (9.3 to 19.5)	1009 (525 to 1610)	15.9 (10.2 to 22.4)	1456 (719 to 2170)	14.9 (9.6 to 20.7)	1690 (955 to 2379)	11.5 (8 to 16.6)	1716 (1148 to 2486)
		Male	16.5 (11.8 to 23.3)	1100 (717 to 1646)	18.3 (12.6 to 27.5)	1583 (967 to 2380)	17.5 (11.5 to 24.5)	1952 (1165 to 2809)	14.8 (9.9 to 20.1)	2285 (1518 to 3229)
	YLDs	Both	0.2 (0.1 to 0.3)	18 (12 to 25)	0.2 (0.1 to 0.3)	23 (15 to 32)	0.2 (0.1 to 0.3)	32 (21 to 44)	0.2 (0.1 to 0.3)	52 (34 to 74)
		Female	0.2 (0.1 to 0.3)	8 (6 to 12)	0.2 (0.1 to 0.2)	11 (7 to 15)	0.2 (0.1 to 0.2)	15 (10 to 21)	0.2 (0.1 to 0.3)	24 (16 to 34)
		Male	0.2 (0.1 to 0.3)	9 (6 to 13)	0.2 (0.1 to 0.3)	12 (8 to 17)	0.2 (0.1 to 0.3)	17 (11 to 24)	0.2 (0.1 to 0.3)	28 (19 to 40)
**Jordan**	Incidence	Both	16.1 (13.7 to 18.6)	423 (344 to 511)	17.5 (15 to 20.2)	556 (463 to 661)	17.5 (14.9 to 20.2)	822 (684 to 974)	17.7 (14.9 to 21.1)	1653 (1362 to 1991)
		Female	16.8 (14.3 to 19.3)	197 (161 to 238)	18.8 (16.2 to 21.7)	266 (222 to 317)	18.3 (15.7 to 21.1)	387 (322 to 460)	17.3 (14.7 to 20.3)	727 (603 to 869)

S11 Table. Age-standardized incidence, prevalence, deaths, Disability-Adjusted Life Years (DALYs), Years of Life Lost (YLLs) and Years Lived with Disability (YLDs) rates and number in twenty-one countries of North Africa and the Middle East in 1990, 1997, 2007, and 2019 according to sex

Country	Measure	Sex	1990		1997		2007		2019	
			Rate†	Number‡	Rate	Number	Rate	Number	Rate	Number
		Male	15.3 (12.8 to 17.9)	226 (180 to 275)	16.3 (13.9 to 19)	291 (239 to 348)	16.7 (14.3 to 19.6)	436 (356 to 522)	18 (15 to 21.5)	926 (753 to 1124)
	Prevalence	Both	4.8 (4.1 to 5.6)	90 (77 to 104)	4.9 (4.2 to 5.8)	120 (103 to 138)	5 (4.3 to 5.8)	182 (156 to 210)	5.1 (4.4 to 6)	394 (337 to 455)
		Female	5.2 (4.4 to 6.1)	45 (38 to 52)	5.4 (4.6 to 6.3)	61 (51 to 71)	5.4 (4.6 to 6.4)	91 (77 to 105)	5.4 (4.6 to 6.4)	191 (161 to 225)
		Male	4.4 (3.7 to 5.2)	45 (38 to 52)	4.5 (3.9 to 5.4)	59 (51 to 68)	4.6 (4 to 5.4)	91 (78 to 105)	4.9 (4.2 to 5.7)	203 (174 to 234)
	Deaths	Both	1.4 (1 to 1.8)	22 (15 to 28)	1.5 (1.1 to 1.9)	30 (19 to 38)	1 (0.8 to 1.3)	31 (22 to 37)	0.8 (0.6 to 1.1)	54 (42 to 69)
		Female	1.9 (1.2 to 2.6)	13 (7 to 18)	2.1 (1.2 to 2.7)	19 (10 to 25)	1.4 (0.9 to 1.8)	18 (11 to 23)	1 (0.7 to 1.5)	30 (20 to 40)
		Male	1 (0.7 to 1.3)	9 (6 to 11)	1 (0.8 to 1.4)	11 (8 to 15)	0.7 (0.6 to 1)	13 (9 to 16)	0.7 (0.5 to 1)	25 (18 to 34)
	DALYs	Both	39.9 (26.3 to 50.8)	865 (548 to 1122)	42.3 (26.7 to 54.6)	1160 (693 to 1517)	28 (20.1 to 33.8)	1146 (780 to 1398)	21.7 (16.6 to 26.8)	1881 (1376 to 2360)
		Female	50.3 (27.3 to 68.1)	491 (253 to 671)	54.7 (28.7 to 74.7)	683 (330 to 947)	34.9 (20.9 to 44.5)	645 (373 to 842)	24.9 (16.4 to 33.2)	961 (601 to 1284)
		Male	30 (20.6 to 38.4)	374 (235 to 502)	30.8 (21.2 to 40.2)	477 (302 to 628)	21.9 (16 to 27.3)	501 (342 to 620)	18.7 (13.6 to 25.5)	920 (637 to 1245)

S11 Table. Age-standardized incidence, prevalence, deaths, Disability-Adjusted Life Years (DALYs), Years of Life Lost (YLLs) and Years Lived with Disability (YLDs) rates and number in twenty-one countries of North Africa and the Middle East in 1990, 1997, 2007, and 2019 according to sex

Country	Measure	Sex	1990		1997		2007		2019	
			Rate†	Number‡	Rate	Number	Rate	Number	Rate	Number
	YLLs	Both	39.5 (25.8 to 50.4)	858 (540 to 1116)	41.9 (26.3 to 54.2)	1150 (684 to 1508)	27.6 (19.7 to 33.4)	1132 (765 to 1382)	21.2 (16.3 to 26.4)	1849 (1341 to 2329)
		Female	49.9 (26.9 to 67.7)	487 (249 to 668)	54.3 (28.2 to 74.2)	678 (324 to 942)	34.4 (20.5 to 44.1)	638 (365 to 835)	24.5 (16 to 32.8)	945 (583 to 1267)
		Male	29.6 (20.2 to 38.1)	371 (232 to 500)	30.4 (20.8 to 39.8)	472 (297 to 623)	21.5 (15.6 to 26.9)	494 (335 to 613)	18.3 (13.2 to 25)	904 (623 to 1228)
	YLDs	Both	0.4 (0.3 to 0.6)	7 (5 to 10)	0.4 (0.3 to 0.6)	9 (6 to 14)	0.4 (0.3 to 0.6)	14 (10 to 20)	0.4 (0.3 to 0.6)	31 (21 to 45)
		Female	0.4 (0.3 to 0.6)	4 (2 to 5)	0.4 (0.3 to 0.6)	5 (3 to 7)	0.4 (0.3 to 0.6)	7 (5 to 11)	0.4 (0.3 to 0.6)	16 (10 to 22)
		Male	0.4 (0.2 to 0.5)	4 (2 to 5)	0.4 (0.2 to 0.5)	5 (3 to 7)	0.4 (0.2 to 0.5)	7 (5 to 10)	0.4 (0.3 to 0.6)	16 (11 to 22)
**Kuwait**	Incidence	Both	11.7 (9.6 to 14.2)	156 (120 to 195)	12.3 (10.1 to 14.9)	175 (138 to 216)	14.3 (11.8 to 17)	292 (232 to 360)	16.1 (13.3 to 19.4)	594 (467 to 752)
		Female	11.4 (9.5 to 13.8)	60 (47 to 75)	12.3 (10.3 to 14.6)	66 (52 to 80)	14.4 (12 to 17)	109 (87 to 132)	16.1 (13.4 to 19.1)	242 (192 to 304)
		Male	11.7 (9.3 to 14.4)	96 (73 to 121)	12.2 (9.9 to 14.9)	109 (85 to 137)	14.2 (11.6 to 17.1)	183 (143 to 228)	16.4 (13.3 to 20)	351 (274 to 444)
	Prevalence	Both	3.1 (2.6 to 3.7)	26 (22 to 30)	3.1 (2.6 to 3.7)	31 (26 to 36)	3.3 (2.8 to 3.9)	51 (44 to 60)	3.5 (3 to 4.1)	101 (86 to 119)

S11 Table. Age-standardized incidence, prevalence, deaths, Disability-Adjusted Life Years (DALYs), Years of Life Lost (YLLs) and Years Lived with Disability (YLDs) rates and number in twenty-one countries of North Africa and the Middle East in 1990, 1997, 2007, and 2019 according to sex

Country	Measure	Sex	1990		1997		2007		2019	
			Rate[†]	Number[‡]	Rate	Number	Rate	Number	Rate	Number
		Female	3.8 (3.2 to 4.7)	12 (10 to 14)	3.9 (3.3 to 4.7)	14 (12 to 17)	4.2 (3.5 to 5.1)	23 (20 to 27)	4.3 (3.6 to 5.2)	50 (42 to 59)
		Male	2.5 (2.1 to 3)	14 (11 to 16)	2.6 (2.2 to 3)	17 (14 to 20)	2.8 (2.4 to 3.2)	28 (23 to 33)	2.9 (2.5 to 3.5)	51 (43 to 60)
	Deaths	Both	0.4 (0.3 to 0.6)	3 (2 to 4)	0.3 (0.3 to 0.5)	3 (2 to 5)	0.5 (0.3 to 0.5)	7 (4 to 8)	0.4 (0.3 to 0.5)	11 (7 to 14)
		Female	0.5 (0.4 to 0.9)	1 (1 to 2)	0.4 (0.3 to 0.8)	1 (1 to 2)	0.6 (0.4 to 0.7)	3 (2 to 3)	0.6 (0.3 to 0.8)	5 (3 to 8)
		Male	0.3 (0.2 to 0.4)	1 (1 to 2)	0.3 (0.2 to 0.5)	2 (1 to 3)	0.4 (0.2 to 0.5)	4 (2 to 4)	0.3 (0.2 to 0.4)	5 (3 to 7)
	DALYs	Both	9.1 (7 to 14.7)	101 (79 to 165)	8.5 (7 to 13.1)	108 (86 to 171)	11.6 (7.7 to 13.5)	230 (145 to 274)	9.9 (6.3 to 12.9)	356 (211 to 473)
		Female	11.8 (8.8 to 21.4)	49 (38 to 84)	10.2 (8 to 18.1)	47 (37 to 84)	13.3 (8.4 to 15.7)	87 (56 to 102)	11.7 (6.5 to 16.4)	161 (87 to 233)
		Male	7.1 (5.4 to 11.6)	53 (40 to 93)	7.5 (6.1 to 12.1)	61 (48 to 104)	10.7 (6.3 to 12.6)	143 (79 to 176)	8.9 (5.4 to 12.1)	196 (113 to 270)
	YLLs	Both	8.8 (6.8 to 14.5)	99 (77 to 163)	8.3 (6.7 to 12.8)	106 (84 to 169)	11.4 (7.4 to 13.2)	226 (141 to 270)	9.7 (6 to 12.7)	349 (204 to 465)
		Female	11.5 (8.5 to 21.1)	48 (37 to 83)	9.9 (7.7 to 17.8)	46 (36 to 83)	12.9 (8.1 to 15.4)	85 (54 to 101)	11.4 (6.2 to 16)	157 (83 to 229)

S11 Table. Age-standardized incidence, prevalence, deaths, Disability-Adjusted Life Years (DALYs), Years of Life Lost (YLLs) and Years Lived with Disability (YLDs) rates and number in twenty-one countries of North Africa and the Middle East in 1990, 1997, 2007, and 2019 according to sex

Country	Measure	Sex	1990		1997		2007		2019	
			Rate[†]	Number[‡]	Rate	Number	Rate	Number	Rate	Number
		Male	6.9 (5.2 to 11.4)	52 (39 to 92)	7.3 (6 to 11.9)	60 (46 to 103)	10.5 (6 to 12.4)	141 (77 to 174)	8.7 (5.1 to 11.8)	192 (109 to 266)
	YLDs	Both	0.2 (0.2 to 0.3)	2 (1 to 3)	0.2 (0.2 to 0.4)	2 (2 to 3)	0.3 (0.2 to 0.4)	4 (3 to 5)	0.3 (0.2 to 0.4)	7 (5 to 11)
		Female	0.3 (0.2 to 0.4)	1 (1 to 1)	0.3 (0.2 to 0.5)	1 (1 to 2)	0.3 (0.2 to 0.5)	2 (1 to 3)	0.3 (0.2 to 0.5)	4 (2 to 5)
		Male	0.2 (0.1 to 0.3)	1 (1 to 1)	0.2 (0.1 to 0.3)	1 (1 to 2)	0.2 (0.1 to 0.3)	2 (1 to 3)	0.2 (0.1 to 0.3)	4 (2 to 5)
Lebanon	Incidence	Both	13.8 (11.7 to 16.1)	409 (340 to 484)	15 (12.8 to 17.2)	499 (422 to 579)	16.4 (13.9 to 19)	645 (549 to 750)	18.5 (15.7 to 21.8)	963 (813 to 1130)
		Female	10.9 (9 to 12.9)	163 (132 to 197)	11.8 (9.9 to 13.9)	197 (164 to 234)	13.2 (11.1 to 15.7)	262 (219 to 312)	15.2 (12.6 to 18.1)	412 (341 to 491)
		Male	16.9 (14.4 to 19.5)	246 (207 to 291)	18.3 (15.6 to 20.8)	302 (257 to 351)	19.7 (16.8 to 22.9)	383 (325 to 445)	22.2 (19 to 25.8)	550 (469 to 640)
	Prevalence	Both	2.7 (2.3 to 3.1)	67 (57 to 77)	2.8 (2.4 to 3.3)	83 (71 to 96)	3 (2.6 to 3.5)	114 (99 to 133)	3.3 (2.8 to 3.8)	170 (146 to 197)
		Female	2.4 (2 to 2.8)	29 (25 to 34)	2.4 (2.1 to 2.9)	36 (31 to 42)	2.6 (2.2 to 3.1)	50 (43 to 59)	2.9 (2.5 to 3.4)	79 (68 to 93)
		Male	3.1 (2.6 to 3.6)	37 (32 to 43)	3.2 (2.7 to 3.7)	47 (40 to 54)	3.4 (3 to 3.9)	64 (56 to 75)	3.8 (3.2 to 4.4)	91 (78 to 105)

S11 Table. Age-standardized incidence, prevalence, deaths, Disability-Adjusted Life Years (DALYs), Years of Life Lost (YLLs) and Years Lived with Disability (YLDs) rates and number in twenty-one countries of North Africa and the Middle East in 1990, 1997, 2007, and 2019 according to sex

Country	Measure	Sex	1990		1997		2007		2019	
			Rate[†]	Number[‡]	Rate	Number	Rate	Number	Rate	Number
	Deaths	Both	0.7 (0.6 to 0.9)	16 (12 to 20)	0.7 (0.5 to 0.9)	18 (14 to 23)	0.5 (0.4 to 0.7)	20 (13 to 26)	0.5 (0.4 to 0.6)	26 (18 to 33)
		Female	0.7 (0.5 to 0.9)	7 (5 to 10)	0.7 (0.5 to 1)	9 (6 to 13)	0.5 (0.3 to 0.8)	9 (6 to 14)	0.4 (0.3 to 0.6)	13 (8 to 18)
		Male	0.7 (0.6 to 1)	8 (6 to 11)	0.7 (0.5 to 0.9)	9 (6 to 12)	0.5 (0.4 to 0.7)	10 (7 to 14)	0.5 (0.4 to 0.7)	13 (9 to 17)
	DALYs	Both	19.5 (15.4 to 26)	549 (407 to 721)	17.7 (13.5 to 22.9)	561 (422 to 735)	13.6 (9.6 to 17.9)	538 (379 to 713)	13.1 (9.3 to 17.1)	687 (484 to 895)
		Female	16.7 (12 to 23)	230 (153 to 326)	16 (11.2 to 22.3)	249 (172 to 349)	11.4 (7.1 to 16.9)	229 (141 to 340)	10.2 (6.4 to 14.4)	284 (178 to 400)
		Male	22.4 (16.6 to 31.5)	319 (228 to 449)	19.3 (13.8 to 25.9)	311 (221 to 423)	15.8 (10.7 to 21.9)	309 (205 to 430)	16.1 (10.8 to 22.4)	403 (269 to 558)
	YLLs	Both	19.3 (15.1 to 25.7)	544 (402 to 717)	17.5 (13.3 to 22.8)	554 (417 to 729)	13.3 (9.4 to 17.7)	529 (371 to 705)	12.8 (9 to 16.9)	674 (474 to 882)
		Female	16.5 (11.8 to 22.9)	228 (151 to 325)	15.9 (11 to 22.1)	247 (169 to 346)	11.2 (6.9 to 16.8)	225 (137 to 337)	9.9 (6.2 to 14.2)	278 (172 to 395)
		Male	22.2 (16.4 to 31.3)	316 (225 to 447)	19.1 (13.6 to 25.7)	308 (218 to 420)	15.5 (10.4 to 21.7)	304 (202 to 426)	15.9 (10.5 to 22.1)	397 (261 to 551)
	YLDs	Both	0.2 (0.1 to 0.3)	5 (3 to 7)	0.2 (0.1 to 0.3)	6 (4 to 9)	0.2 (0.1 to 0.3)	9 (6 to 12)	0.2 (0.2 to 0.4)	13 (8 to 18)

S11 Table. Age-standardized incidence, prevalence, deaths, Disability-Adjusted Life Years (DALYs), Years of Life Lost (YLLs) and Years Lived with Disability (YLDs) rates and number in twenty-one countries of North Africa and the Middle East in 1990, 1997, 2007, and 2019 according to sex

Country	Measure	Sex	1990		1997		2007		2019	
			Rate†	Number‡	Rate	Number	Rate	Number	Rate	Number
		Female	0.2 (0.1 to 0.3)	2 (1 to 3)	0.2 (0.1 to 0.3)	3 (2 to 4)	0.2 (0.1 to 0.3)	4 (3 to 6)	0.2 (0.1 to 0.3)	6 (4 to 9)
		Male	0.2 (0.2 to 0.3)	3 (2 to 4)	0.2 (0.2 to 0.3)	3 (2 to 5)	0.3 (0.2 to 0.4)	5 (3 to 7)	0.3 (0.2 to 0.4)	7 (4 to 9)
Libya	Incidence	Both	10.9 (8.9 to 13.1)	451 (353 to 559)	11.6 (9.7 to 13.7)	508 (410 to 615)	12.5 (10.5 to 14.8)	616 (502 to 737)	13.3 (10.9 to 16.1)	779 (627 to 971)
		Female	10 (8.1 to 12.1)	205 (159 to 259)	10.7 (9 to 12.7)	232 (187 to 283)	11.6 (9.8 to 13.8)	273 (223 to 326)	12.3 (10.1 to 15)	340 (275 to 421)
		Male	11.6 (9.5 to 14.1)	246 (192 to 304)	12.4 (10.2 to 14.7)	275 (221 to 335)	13.3 (11.1 to 15.6)	344 (280 to 411)	14.1 (11.6 to 17.1)	439 (350 to 545)
	Prevalence	Both	2.1 (1.8 to 2.4)	62 (53 to 73)	2.2 (1.9 to 2.6)	73 (63 to 85)	2.4 (2.1 to 2.8)	96 (83 to 110)	2.6 (2.2 to 3)	135 (116 to 157)
		Female	2 (1.7 to 2.3)	29 (25 to 34)	2.1 (1.9 to 2.5)	35 (30 to 40)	2.3 (2 to 2.6)	44 (38 to 51)	2.4 (2.1 to 2.9)	62 (53 to 72)
		Male	2.2 (1.9 to 2.5)	33 (28 to 39)	2.3 (2 to 2.7)	38 (33 to 45)	2.5 (2.2 to 2.9)	52 (45 to 60)	2.7 (2.3 to 3.2)	73 (63 to 85)
	Deaths	Both	0.6 (0.4 to 0.9)	26 (12 to 44)	0.5 (0.4 to 0.8)	21 (12 to 31)	0.5 (0.3 to 0.7)	21 (13 to 28)	0.4 (0.3 to 0.6)	23 (15 to 31)
		Female	0.6 (0.3 to 1)	12 (5 to 23)	0.5 (0.3 to 0.8)	10 (5 to 16)	0.5 (0.3 to 0.7)	10 (5 to 14)	0.5 (0.3 to 0.6)	11 (7 to 16)

S11 Table. Age-standardized incidence, prevalence, deaths, Disability-Adjusted Life Years (DALYs), Years of Life Lost (YLLs) and Years Lived with Disability (YLDs) rates and number in twenty-one countries of North Africa and the Middle East in 1990, 1997, 2007, and 2019 according to sex

Country	Measure	Sex	1990		1997		2007		2019	
			Rate†	Number‡	Rate	Number	Rate	Number	Rate	Number
		Male	0.7 (0.4 to 1)	14 (6 to 26)	0.6 (0.4 to 0.8)	11 (6 to 18)	0.5 (0.3 to 0.7)	11 (7 to 16)	0.4 (0.3 to 0.7)	12 (7 to 18)
	DALYs	Both	32.5 (15.1 to 54.5)	1855 (617 to 3457)	26.1 (14.1 to 40.6)	1351 (622 to 2238)	21.4 (12.2 to 31.6)	1125 (614 to 1717)	16.8 (10.1 to 25.2)	940 (578 to 1355)
		Female	30.7 (12.2 to 57.5)	855 (223 to 1814)	24.8 (11.2 to 42.7)	626 (229 to 1164)	20.7 (10.1 to 32.2)	516 (236 to 845)	16.4 (8.8 to 25)	435 (244 to 630)
		Male	34.1 (15.5 to 64.2)	1000 (343 to 2101)	27.3 (14.7 to 44.3)	726 (340 to 1248)	22.1 (12.2 to 34.9)	609 (322 to 984)	17.2 (9.8 to 28.7)	505 (294 to 793)
	YLLs	Both	32.3 (15 to 54.3)	1851 (613 to 3452)	25.9 (14 to 40.4)	1346 (617 to 2230)	21.2 (12 to 31.4)	1118 (606 to 1709)	16.6 (9.9 to 25)	930 (567 to 1341)
		Female	30.6 (12.1 to 57.3)	853 (220 to 1811)	24.7 (11.1 to 42.6)	623 (226 to 1162)	20.5 (10 to 32)	513 (233 to 841)	16.3 (8.6 to 24.8)	430 (240 to 625)
		Male	34 (15.3 to 64)	998 (340 to 2098)	27.1 (14.5 to 44.1)	723 (337 to 1246)	21.9 (12 to 34.7)	605 (318 to 980)	17 (9.6 to 28.4)	499 (289 to 788)
	YLDs	Both	0.2 (0.1 to 0.2)	4 (3 to 6)	0.2 (0.1 to 0.2)	5 (3 to 7)	0.2 (0.1 to 0.3)	7 (5 to 10)	0.2 (0.1 to 0.3)	10 (7 to 14)
		Female	0.2 (0.1 to 0.2)	2 (1 to 3)	0.2 (0.1 to 0.2)	3 (2 to 4)	0.2 (0.1 to 0.3)	3 (2 to 5)	0.2 (0.1 to 0.3)	5 (3 to 7)
		Male	0.2 (0.1 to 0.2)	2 (2 to 3)	0.2 (0.1 to 0.3)	3 (2 to 4)	0.2 (0.1 to 0.3)	4 (2 to 5)	0.2 (0.1 to 0.3)	5 (4 to 8)

S11 Table. Age-standardized incidence, prevalence, deaths, Disability-Adjusted Life Years (DALYs), Years of Life Lost (YLLs) and Years Lived with Disability (YLDs) rates and number in twenty-one countries of North Africa and the Middle East in 1990, 1997, 2007, and 2019 according to sex

Country	Measure	Sex	1990		1997		2007		2019	
			Rate†	Number‡	Rate	Number	Rate	Number	Rate	Number
Morocco	Incidence	Both	8.8 (7.1 to 10.7)	1946 (1541 to 2381)	9.1 (7.4 to 10.9)	2291 (1847 to 2782)	10.5 (8.7 to 12.6)	3082 (2500 to 3727)	12.9 (10.6 to 15.6)	4365 (3550 to 5293)
		Female	7.9 (6.4 to 9.6)	881 (696 to 1092)	8.2 (6.7 to 9.9)	1042 (840 to 1267)	9.6 (8 to 11.5)	1413 (1155 to 1702)	11.9 (9.9 to 14.5)	1983 (1608 to 2421)
		Male	9.7 (7.8 to 11.7)	1064 (839 to 1313)	10 (8.2 to 12.1)	1249 (999 to 1519)	11.4 (9.4 to 13.7)	1669 (1342 to 2038)	13.9 (11.4 to 16.9)	2381 (1925 to 2922)
	Prevalence	Both	1.8 (1.5 to 2.1)	290 (246 to 338)	1.8 (1.6 to 2.2)	356 (305 to 413)	1.9 (1.6 to 2.2)	468 (404 to 544)	2.2 (1.8 to 2.5)	659 (564 to 768)
		Female	1.6 (1.4 to 1.9)	133 (113 to 156)	1.7 (1.4 to 2)	164 (141 to 190)	1.8 (1.5 to 2.1)	214 (184 to 248)	2 (1.7 to 2.3)	299 (257 to 349)
		Male	1.9 (1.6 to 2.3)	156 (133 to 183)	2 (1.7 to 2.3)	192 (164 to 224)	2.1 (1.8 to 2.4)	254 (218 to 296)	2.3 (2 to 2.7)	360 (306 to 424)
	Deaths	Both	0.5 (0.3 to 0.7)	68 (44 to 100)	0.5 (0.3 to 0.7)	87 (59 to 122)	0.5 (0.4 to 0.8)	120 (84 to 166)	0.6 (0.4 to 0.8)	165 (119 to 216)
		Female	0.4 (0.2 to 0.7)	32 (18 to 50)	0.5 (0.3 to 0.8)	42 (25 to 63)	0.6 (0.3 to 0.9)	61 (40 to 87)	0.6 (0.4 to 0.8)	81 (55 to 113)
		Male	0.5 (0.3 to 0.8)	36 (23 to 55)	0.5 (0.3 to 0.8)	45 (29 to 67)	0.5 (0.3 to 0.8)	59 (39 to 88)	0.6 (0.4 to 0.9)	84 (55 to 120)
	DALYs	Both	13.5 (9 to 19.4)	2899 (1907 to 4221)	14.4 (9.9 to 19.9)	3475 (2396 to 4858)	15.1 (10.8 to 20.2)	4216 (2955 to 5674)	14.6 (10.6 to 19.4)	4776 (3385 to 6411)

S11 Table. Age-standardized incidence, prevalence, deaths, Disability-Adjusted Life Years (DALYs), Years of Life Lost (YLLs) and Years Lived with Disability (YLDs) rates and number in twenty-one countries of North Africa and the Middle East in 1990, 1997, 2007, and 2019 according to sex

Country	Measure	Sex	1990		1997		2007		2019	
			Rate[†]	Number[‡]	Rate	Number	Rate	Number	Rate	Number
		Female	12.6 (7.3 to 19.4)	1394 (743 to 2369)	13.6 (8.4 to 20.4)	1662 (964 to 2710)	14.8 (9.7 to 20.3)	2035 (1286 to 2895)	13.9 (9.4 to 19.6)	2264 (1505 to 3364)
		Male	14.5 (9.3 to 21.8)	1505 (963 to 2306)	15.2 (9.9 to 22.1)	1813 (1181 to 2709)	15.6 (10.4 to 22.9)	2181 (1429 to 3284)	15.3 (10.2 to 22.1)	2512 (1666 to 3659)
	YLLs	Both	13.4 (8.9 to 19.2)	2877 (1891 to 4207)	14.2 (9.8 to 19.8)	3449 (2377 to 4836)	15 (10.7 to 20)	4181 (2923 to 5636)	14.5 (10.4 to 19.3)	4727 (3353 to 6361)
		Female	12.4 (7.2 to 19.2)	1384 (734 to 2360)	13.5 (8.3 to 20.3)	1650 (951 to 2698)	14.7 (9.6 to 20.2)	2019 (1272 to 2878)	13.8 (9.3 to 19.5)	2242 (1483 to 3347)
		Male	14.3 (9.2 to 21.7)	1493 (952 to 2292)	15 (9.8 to 22)	1799 (1169 to 2696)	15.4 (10.2 to 22.8)	2162 (1412 to 3265)	15.2 (10.1 to 21.8)	2485 (1634 to 3628)
	YLDs	Both	0.1 (0.1 to 0.2)	22 (14 to 31)	0.1 (0.1 to 0.2)	27 (18 to 38)	0.1 (0.1 to 0.2)	35 (23 to 49)	0.2 (0.1 to 0.2)	49 (32 to 69)
		Female	0.1 (0.1 to 0.2)	10 (7 to 14)	0.1 (0.1 to 0.2)	12 (8 to 17)	0.1 (0.1 to 0.2)	16 (10 to 22)	0.1 (0.1 to 0.2)	22 (15 to 31)
		Male	0.2 (0.1 to 0.2)	12 (8 to 17)	0.2 (0.1 to 0.2)	14 (10 to 20)	0.2 (0.1 to 0.2)	19 (13 to 27)	0.2 (0.1 to 0.3)	27 (17 to 38)
**Oman**	Incidence	Both	10.9 (9 to 13.1)	168 (132 to 206)	12.8 (10.7 to 15)	217 (174 to 265)	15.1 (12.8 to 17.6)	282 (229 to 339)	16.4 (13.8 to 19.4)	540 (425 to 667)
		Female	9.9 (8.2 to 12)	67 (51 to 84)	11.9 (10 to 13.9)	86 (69 to 106)	14.2 (12 to 16.7)	112 (91 to 136)	15.2 (12.7 to 17.8)	181 (144 to 218)

S11 Table. Age-standardized incidence, prevalence, deaths, Disability-Adjusted Life Years (DALYs), Years of Life Lost (YLLs) and Years Lived with Disability (YLDs) rates and number in twenty-one countries of North Africa and the Middle East in 1990, 1997, 2007, and 2019 according to sex

Country	Measure	Sex	1990		1997		2007		2019	
			Rate†	Number‡	Rate	Number	Rate	Number	Rate	Number
		Male	11.4 (9.4 to 13.7)	101 (79 to 125)	13.3 (11 to 15.5)	132 (105 to 161)	15.7 (13.2 to 18.2)	170 (137 to 205)	17 (14.2 to 20.1)	359 (275 to 451)
	Prevalence	Both	2 (1.8 to 2.3)	24 (21 to 28)	2.2 (2 to 2.5)	31 (27 to 36)	2.8 (2.4 to 3.2)	42 (36 to 49)	2.9 (2.5 to 3.3)	79 (67 to 93)
		Female	1.9 (1.7 to 2.2)	10 (8 to 12)	2.1 (1.9 to 2.4)	12 (11 to 15)	2.6 (2.3 to 3)	17 (15 to 20)	2.7 (2.3 to 3.2)	27 (23 to 31)
		Male	2 (1.8 to 2.3)	14 (12 to 17)	2.3 (2 to 2.6)	18 (16 to 21)	2.9 (2.5 to 3.3)	25 (21 to 29)	3 (2.6 to 3.5)	52 (43 to 62)
	Deaths	Both	0.7 (0.4 to 1.1)	6 (3 to 10)	0.8 (0.5 to 1.1)	7 (4 to 10)	0.8 (0.6 to 1)	9 (6 to 11)	0.7 (0.5 to 0.9)	13 (9 to 16)
		Female	0.7 (0.3 to 1.2)	3 (1 to 5)	0.8 (0.3 to 1.4)	3 (1 to 5)	0.8 (0.5 to 1.3)	4 (2 to 6)	0.7 (0.5 to 1)	5 (3 to 6)
		Male	0.7 (0.4 to 1.1)	3 (2 to 6)	0.7 (0.5 to 1)	4 (3 to 6)	0.8 (0.5 to 1)	5 (3 to 7)	0.7 (0.4 to 0.9)	8 (5 to 11)
	DALYs	Both	19.8 (10.7 to 31.7)	284 (142 to 553)	20.8 (12.5 to 29.8)	318 (181 to 524)	21.4 (15.1 to 27.2)	374 (254 to 492)	17.3 (12.6 to 21.5)	527 (379 to 766)
		Female	18.8 (7.6 to 34.1)	119 (39 to 268)	20.6 (8.9 to 37)	132 (49 to 247)	20.6 (12.6 to 31.3)	144 (81 to 214)	17 (11.2 to 22.8)	181 (115 to 260)
		Male	20.1 (11.6 to 33.1)	164 (88 to 330)	20.6 (13 to 31.7)	187 (111 to 319)	21.8 (14.4 to 28.6)	230 (147 to 317)	17.1 (11.5 to 22.9)	345 (232 to 538)

S11 Table. Age-standardized incidence, prevalence, deaths, Disability-Adjusted Life Years (DALYs), Years of Life Lost (YLLs) and Years Lived with Disability (YLDs) rates and number in twenty-one countries of North Africa and the Middle East in 1990, 1997, 2007, and 2019 according to sex

Country	Measure	Sex	1990		1997		2007		2019	
			Rate†	Number‡	Rate	Number	Rate	Number	Rate	Number
	YLLs	Both	19.6 (10.5 to 31.6)	282 (140 to 551)	20.6 (12.3 to 29.6)	316 (179 to 521)	21.2 (14.9 to 27)	371 (252 to 488)	17 (12.3 to 21.3)	521 (375 to 761)
		Female	18.7 (7.4 to 34)	118 (39 to 267)	20.5 (8.7 to 36.9)	131 (48 to 246)	20.4 (12.4 to 31.1)	143 (80 to 213)	16.8 (11 to 22.6)	179 (113 to 258)
		Male	20 (11.4 to 33)	163 (86 to 329)	20.4 (12.8 to 31.5)	185 (110 to 318)	21.6 (14.2 to 28.4)	228 (145 to 315)	16.9 (11.3 to 22.7)	342 (229 to 535)
	YLDs	Both	0.2 (0.1 to 0.2)	2 (1 to 2)	0.2 (0.1 to 0.2)	2 (1 to 3)	0.2 (0.1 to 0.3)	3 (2 to 4)	0.2 (0.1 to 0.3)	6 (4 to 8)
		Female	0.1 (0.1 to 0.2)	1 (0 to 1)	0.2 (0.1 to 0.2)	1 (1 to 1)	0.2 (0.1 to 0.3)	1 (1 to 2)	0.2 (0.1 to 0.3)	2 (1 to 3)
		Male	0.2 (0.1 to 0.2)	1 (1 to 1)	0.2 (0.1 to 0.2)	1 (1 to 2)	0.2 (0.1 to 0.3)	2 (1 to 3)	0.2 (0.1 to 0.3)	4 (2 to 5)
**Palestine**	Incidence	Both	9.3 (7.5 to 11.4)	168 (131 to 213)	9.7 (7.9 to 11.8)	231 (180 to 290)	10.5 (8.5 to 12.7)	351 (274 to 437)	12.7 (10.3 to 15.6)	537 (420 to 669)
		Female	8.4 (6.7 to 10.2)	78 (61 to 100)	8.8 (7.2 to 10.7)	107 (84 to 137)	9.5 (7.8 to 11.6)	161 (126 to 204)	11.7 (9.5 to 14.3)	243 (190 to 304)
		Male	10.3 (8.3 to 12.5)	90 (69 to 115)	10.6 (8.6 to 13)	123 (95 to 156)	11.4 (9.2 to 13.9)	190 (148 to 238)	13.7 (11.1 to 16.9)	294 (230 to 370)
	Prevalence	Both	1.6 (1.4 to 1.9)	22 (18 to 26)	1.7 (1.5 to 2)	29 (24 to 34)	1.7 (1.5 to 2)	43 (36 to 50)	2 (1.7 to 2.3)	67 (56 to 79)

S11 Table. Age-standardized incidence, prevalence, deaths, Disability-Adjusted Life Years (DALYs), Years of Life Lost (YLLs) and Years Lived with Disability (YLDs) rates and number in twenty-one countries of North Africa and the Middle East in 1990, 1997, 2007, and 2019 according to sex

Country	Measure	Sex	1990		1997		2007		2019	
			Rate†	Number‡	Rate	Number	Rate	Number	Rate	Number
		Female	1.5 (1.3 to 1.8)	10 (9 to 12)	1.6 (1.3 to 1.9)	14 (11 to 16)	1.6 (1.4 to 1.9)	20 (17 to 24)	1.8 (1.6 to 2.2)	31 (26 to 36)
		Male	1.8 (1.5 to 2.1)	11 (9 to 14)	1.8 (1.6 to 2.1)	15 (13 to 18)	1.8 (1.6 to 2.2)	23 (19 to 27)	2.1 (1.8 to 2.4)	36 (30 to 43)
	Deaths	Both	0.4 (0.3 to 0.6)	4 (2 to 6)	0.4 (0.3 to 0.5)	5 (3 to 7)	0.3 (0.2 to 0.4)	5 (4 to 6)	0.3 (0.2 to 0.4)	7 (5 to 9)
		Female	0.3 (0.2 to 0.5)	2 (1 to 3)	0.3 (0.2 to 0.5)	2 (1 to 3)	0.3 (0.2 to 0.4)	2 (2 to 3)	0.3 (0.2 to 0.4)	3 (2 to 4)
		Male	0.5 (0.3 to 0.7)	2 (1 to 4)	0.5 (0.3 to 0.6)	3 (2 to 4)	0.4 (0.3 to 0.5)	3 (2 to 4)	0.3 (0.2 to 0.4)	4 (2 to 5)
	DALYs	Both	11.3 (6.5 to 17.2)	164 (80 to 270)	10.5 (6.4 to 15)	187 (99 to 284)	8.4 (6 to 10.2)	196 (137 to 247)	7.7 (5 to 9.9)	251 (158 to 323)
		Female	8.8 (4.6 to 13.5)	68 (29 to 118)	8 (4.5 to 12)	72 (35 to 115)	6.6 (4.3 to 8.4)	76 (49 to 100)	6.5 (3.8 to 8.5)	99 (58 to 130)
		Male	14.1 (7.3 to 22.4)	96 (44 to 170)	13.1 (7.3 to 19.8)	115 (57 to 188)	10.2 (6.7 to 12.9)	120 (77 to 159)	8.9 (5.5 to 11.7)	151 (91 to 206)
	YLLs	Both	11.2 (6.4 to 17.1)	162 (78 to 268)	10.4 (6.3 to 14.9)	185 (97 to 283)	8.2 (5.9 to 10)	193 (134 to 244)	7.6 (4.8 to 9.8)	246 (153 to 318)
		Female	8.7 (4.5 to 13.4)	67 (28 to 117)	7.9 (4.4 to 11.9)	71 (35 to 114)	6.5 (4.2 to 8.3)	75 (48 to 99)	6.4 (3.6 to 8.3)	97 (55 to 127)

S11 Table. Age-standardized incidence, prevalence, deaths, Disability-Adjusted Life Years (DALYs), Years of Life Lost (YLLs) and Years Lived with Disability (YLDs) rates and number in twenty-one countries of North Africa and the Middle East in 1990, 1997, 2007, and 2019 according to sex

Country	Measure	Sex	1990		1997		2007		2019	
			Rate†	Number‡	Rate	Number	Rate	Number	Rate	Number
		Male	14 (7.2 to 22.2)	95 (44 to 169)	13 (7.2 to 19.6)	114 (56 to 187)	10 (6.6 to 12.8)	118 (75 to 157)	8.7 (5.4 to 11.5)	149 (88 to 203)
	YLDs	Both	0.1 (0.1 to 0.2)	2 (1 to 2)	0.1 (0.1 to 0.2)	2 (1 to 3)	0.1 (0.1 to 0.2)	3 (2 to 4)	0.1 (0.1 to 0.2)	5 (3 to 7)
		Female	0.1 (0.1 to 0.2)	1 (0 to 1)	0.1 (0.1 to 0.2)	1 (1 to 1)	0.1 (0.1 to 0.2)	1 (1 to 2)	0.1 (0.1 to 0.2)	2 (1 to 3)
		Male	0.1 (0.1 to 0.2)	1 (1 to 1)	0.1 (0.1 to 0.2)	1 (1 to 2)	0.1 (0.1 to 0.2)	2 (1 to 2)	0.2 (0.1 to 0.2)	2 (2 to 4)
Qatar	Incidence	Both	11.2 (9.1 to 13.6)	39 (29 to 49)	11.7 (9.6 to 14)	50 (38 to 62)	13.9 (11.5 to 16.6)	118 (88 to 150)	15.4 (12.6 to 18.5)	337 (251 to 432)
		Female	9 (7.2 to 11.1)	11 (8 to 14)	9.5 (7.7 to 11.5)	14 (11 to 18)	11.6 (9.6 to 14)	27 (20 to 34)	13.2 (10.8 to 16.1)	72 (55 to 92)
		Male	12.2 (10.1 to 14.8)	28 (21 to 36)	12.7 (10.5 to 15.2)	36 (27 to 45)	14.7 (12.2 to 17.6)	91 (67 to 116)	16.1 (13.2 to 19.4)	265 (195 to 347)
	Prevalence	Both	2.9 (2.6 to 3.4)	7 (6 to 9)	3.2 (2.8 to 3.7)	10 (8 to 11)	3.4 (3 to 4)	22 (19 to 26)	3.4 (2.9 to 4.1)	57 (46 to 68)
		Female	1.8 (1.6 to 2.1)	1 (1 to 2)	1.9 (1.7 to 2.2)	2 (2 to 2)	2 (1.7 to 2.3)	4 (3 to 4)	2.1 (1.8 to 2.5)	9 (7 to 11)
		Male	3.6 (3.1 to 4.3)	6 (5 to 7)	3.9 (3.4 to 4.6)	8 (7 to 9)	4 (3.5 to 4.7)	19 (16 to 22)	3.8 (3.2 to 4.7)	48 (39 to 58)

S11 Table. Age-standardized incidence, prevalence, deaths, Disability-Adjusted Life Years (DALYs), Years of Life Lost (YLLs) and Years Lived with Disability (YLDs) rates and number in twenty-one countries of North Africa and the Middle East in 1990, 1997, 2007, and 2019 according to sex

Country	Measure	Sex	1990		1997		2007		2019	
			Rate†	Number‡	Rate	Number	Rate	Number	Rate	Number
	Deaths	Both	0.5 (0.4 to 0.7)	1 (1 to 1)	0.5 (0.4 to 0.7)	1 (1 to 1)	0.5 (0.3 to 0.6)	2 (1 to 2)	0.4 (0.3 to 0.6)	4 (2 to 6)
		Female	0.3 (0.2 to 0.5)	0 (0 to 0)	0.4 (0.2 to 0.5)	0 (0 to 0)	0.4 (0.2 to 0.6)	0 (0 to 0)	0.4 (0.2 to 0.6)	1 (0 to 1)
		Male	0.7 (0.5 to 0.9)	1 (0 to 1)	0.6 (0.4 to 0.9)	1 (1 to 1)	0.5 (0.3 to 0.6)	1 (1 to 2)	0.4 (0.3 to 0.7)	3 (2 to 5)
	DALYs	Both	14 (10.4 to 17.8)	40 (30 to 53)	12.5 (9.2 to 16.7)	43 (31 to 60)	10.6 (8 to 13.6)	74 (56 to 99)	9.1 (6.1 to 12.7)	168 (106 to 244)
		Female	8.4 (5.8 to 11.9)	7 (5 to 10)	8.1 (5.4 to 11.8)	8 (5 to 12)	9 (5.2 to 12)	13 (9 to 17)	7.4 (4.7 to 10.2)	24 (14 to 36)
		Male	17.3 (12.2 to 22.8)	33 (23 to 45)	14.8 (10.4 to 20.4)	35 (25 to 51)	11.1 (8.1 to 14.7)	62 (44 to 86)	9.6 (6.2 to 13.8)	144 (89 to 214)
	YLLs	Both	13.7 (10.2 to 17.6)	39 (29 to 52)	12.2 (9 to 16.5)	42 (31 to 59)	10.4 (7.7 to 13.3)	73 (54 to 97)	8.8 (5.8 to 12.5)	164 (102 to 240)
		Female	8.3 (5.7 to 11.8)	7 (5 to 10)	7.9 (5.2 to 11.7)	7 (5 to 11)	8.8 (5 to 11.9)	13 (8 to 17)	7.2 (4.5 to 10)	23 (14 to 35)
		Male	17 (11.9 to 22.5)	33 (23 to 45)	14.5 (10.1 to 20.2)	34 (24 to 50)	10.8 (7.8 to 14.3)	60 (43 to 84)	9.3 (5.9 to 13.4)	141 (86 to 210)
	YLDs	Both	0.2 (0.2 to 0.3)	1 (0 to 1)	0.3 (0.2 to 0.4)	1 (1 to 1)	0.3 (0.2 to 0.4)	2 (1 to 2)	0.3 (0.2 to 0.4)	4 (3 to 6)

S11 Table. Age-standardized incidence, prevalence, deaths, Disability-Adjusted Life Years (DALYs), Years of Life Lost (YLLs) and Years Lived with Disability (YLDs) rates and number in twenty-one countries of North Africa and the Middle East in 1990, 1997, 2007, and 2019 according to sex

Country	Measure	Sex	1990		1997		2007		2019	
			Rate†	Number‡	Rate	Number	Rate	Number	Rate	Number
		Female	0.1 (0.1 to 0.2)	0 (0 to 0)	0.2 (0.1 to 0.2)	0 (0 to 0)	0.2 (0.1 to 0.2)	0 (0 to 0)	0.2 (0.1 to 0.2)	1 (0 to 1)
		Male	0.3 (0.2 to 0.4)	0 (0 to 1)	0.3 (0.2 to 0.5)	1 (0 to 1)	0.3 (0.2 to 0.5)	1 (1 to 2)	0.3 (0.2 to 0.4)	4 (2 to 5)
Saudi Arabia	Incidence	Both	10.8 (8.9 to 13)	1379 (1079 to 1729)	11.7 (9.8 to 13.9)	1803 (1432 to 2224)	14.4 (12.1 to 16.9)	2799 (2268 to 3385)	17.2 (14.4 to 20.4)	4948 (3953 to 6101)
		Female	10.2 (8.4 to 12.2)	557 (431 to 711)	11.2 (9.5 to 13.2)	734 (585 to 918)	14.4 (12.3 to 16.9)	1159 (943 to 1410)	17.3 (14.6 to 20.3)	1983 (1622 to 2406)
		Male	11.2 (9.1 to 13.6)	822 (640 to 1023)	11.9 (9.8 to 14.3)	1069 (847 to 1322)	14.3 (11.9 to 17)	1639 (1307 to 2004)	17.1 (14.2 to 20.3)	2965 (2326 to 3704)
	Prevalence	Both	3.1 (2.6 to 3.7)	234 (198 to 275)	3.3 (2.8 to 4)	316 (270 to 369)	3.6 (3.1 to 4.3)	471 (403 to 547)	3.9 (3.3 to 4.6)	808 (689 to 943)
		Female	3.3 (2.8 to 4.1)	104 (88 to 122)	3.6 (3 to 4.3)	140 (119 to 164)	3.9 (3.3 to 4.7)	208 (176 to 243)	4.2 (3.5 to 5)	338 (289 to 396)
		Male	2.8 (2.4 to 3.5)	131 (111 to 153)	3 (2.6 to 3.7)	177 (151 to 208)	3.4 (2.8 to 4)	263 (224 to 307)	3.7 (3.1 to 4.4)	470 (397 to 555)
	Deaths	Both	0.7 (0.4 to 1.1)	47 (30 to 68)	0.7 (0.5 to 1)	62 (42 to 81)	0.7 (0.5 to 0.9)	92 (55 to 119)	0.6 (0.4 to 0.8)	145 (82 to 208)
		Female	0.8 (0.5 to 1.4)	23 (13 to 37)	0.9 (0.6 to 1.3)	31 (19 to 44)	0.9 (0.5 to 1.1)	46 (24 to 60)	0.8 (0.5 to 1.1)	72 (36 to 109)

S11 Table. Age-standardized incidence, prevalence, deaths, Disability-Adjusted Life Years (DALYs), Years of Life Lost (YLLs) and Years Lived with Disability (YLDs) rates and number in twenty-one countries of North Africa and the Middle East in 1990, 1997, 2007, and 2019 according to sex

Country	Measure	Sex	1990		1997		2007		2019	
			Rate†	Number‡	Rate	Number	Rate	Number	Rate	Number
		Male	0.6 (0.4 to 0.9)	24 (15 to 34)	0.6 (0.4 to 0.8)	30 (19 to 40)	0.6 (0.4 to 0.8)	46 (26 to 62)	0.5 (0.3 to 0.7)	73 (39 to 109)
	DALYs	Both	18.7 (12.2 to 26.9)	1803 (1178 to 2510)	18.8 (12.9 to 24.7)	2320 (1594 to 3083)	19.7 (12.5 to 24.8)	3633 (1948 to 4836)	17.6 (10.9 to 24)	5860 (2952 to 8718)
		Female	22.2 (12.5 to 35.7)	871 (487 to 1327)	22.9 (14.2 to 32.2)	1156 (724 to 1605)	23.8 (13.3 to 30.6)	1788 (839 to 2414)	22.1 (12.2 to 31.5)	2834 (1257 to 4449)
		Male	16.3 (10.1 to 22.7)	932 (590 to 1311)	15.8 (10.3 to 20.8)	1163 (767 to 1571)	16.9 (10 to 22)	1845 (961 to 2531)	14.6 (8.4 to 20.9)	3026 (1509 to 4715)
	YLLs	Both	18.5 (11.9 to 26.7)	1785 (1159 to 2494)	18.5 (12.7 to 24.5)	2296 (1569 to 3063)	19.4 (12.3 to 24.5)	3598 (1909 to 4808)	17.3 (10.6 to 23.7)	5800 (2900 to 8651)
		Female	21.9 (12.2 to 35.4)	863 (479 to 1318)	22.6 (13.9 to 31.9)	1146 (714 to 1595)	23.5 (12.9 to 30.3)	1772 (823 to 2399)	21.8 (11.9 to 31.1)	2808 (1239 to 4420)
		Male	16 (10 to 22.4)	922 (580 to 1305)	15.6 (10 to 20.6)	1150 (754 to 1558)	16.6 (9.7 to 21.7)	1826 (934 to 2514)	14.3 (8.1 to 20.5)	2992 (1474 to 4676)
	YLDs	Both	0.2 (0.2 to 0.4)	18 (12 to 25)	0.3 (0.2 to 0.4)	24 (16 to 34)	0.3 (0.2 to 0.4)	35 (23 to 50)	0.3 (0.2 to 0.4)	60 (40 to 85)
		Female	0.3 (0.2 to 0.4)	8 (5 to 11)	0.3 (0.2 to 0.4)	11 (7 to 15)	0.3 (0.2 to 0.4)	16 (10 to 22)	0.3 (0.2 to 0.5)	25 (17 to 36)
		Male	0.2 (0.1 to 0.3)	10 (6 to 14)	0.2 (0.2 to 0.3)	13 (9 to 19)	0.3 (0.2 to 0.4)	19 (13 to 28)	0.3 (0.2 to 0.4)	35 (23 to 50)

S11 Table. Age-standardized incidence, prevalence, deaths, Disability-Adjusted Life Years (DALYs), Years of Life Lost (YLLs) and Years Lived with Disability (YLDs) rates and number in twenty-one countries of North Africa and the Middle East in 1990, 1997, 2007, and 2019 according to sex

Country	Measure	Sex	1990		1997		2007		2019	
			Rate[†]	Number[‡]	Rate	Number	Rate	Number	Rate	Number
Sudan	Incidence	Both	8.5 (7 to 10.1)	1521 (1204 to 1867)	8.5 (7.1 to 10.1)	1886 (1510 to 2340)	8.9 (7.3 to 10.7)	2456 (1948 to 3031)	10.9 (8.9 to 13.3)	3754 (2959 to 4632)
		Female	7.6 (6.3 to 9.2)	684 (537 to 841)	7.6 (6.3 to 9.1)	842 (674 to 1038)	8 (6.5 to 9.7)	1079 (856 to 1346)	9.9 (8 to 12)	1639 (1283 to 2034)
		Male	9.3 (7.7 to 11.2)	837 (663 to 1039)	9.4 (7.8 to 11.1)	1045 (836 to 1298)	9.8 (8.1 to 11.7)	1377 (1094 to 1704)	12 (9.7 to 14.6)	2115 (1656 to 2619)
	Prevalence	Both	1.4 (1.2 to 1.6)	178 (152 to 209)	1.4 (1.2 to 1.7)	228 (196 to 267)	1.5 (1.3 to 1.7)	297 (253 to 349)	1.7 (1.5 to 2)	446 (379 to 527)
		Female	1.2 (1 to 1.4)	77 (65 to 90)	1.2 (1.1 to 1.4)	98 (83 to 115)	1.3 (1.1 to 1.5)	126 (107 to 148)	1.5 (1.2 to 1.7)	186 (155 to 220)
		Male	1.6 (1.3 to 1.8)	101 (86 to 120)	1.6 (1.4 to 1.9)	131 (112 to 154)	1.7 (1.4 to 2)	172 (145 to 200)	1.9 (1.6 to 2.2)	261 (221 to 308)
	Deaths	Both	0.7 (0.5 to 1.1)	102 (52 to 179)	0.6 (0.5 to 0.9)	105 (62 to 166)	0.5 (0.3 to 0.9)	99 (61 to 158)	0.5 (0.3 to 0.8)	110 (70 to 177)
		Female	0.7 (0.4 to 1.2)	50 (19 to 109)	0.6 (0.3 to 0.9)	49 (23 to 99)	0.4 (0.3 to 0.7)	43 (23 to 76)	0.4 (0.3 to 0.7)	46 (27 to 74)
		Male	0.8 (0.5 to 1.2)	53 (28 to 92)	0.7 (0.5 to 1.1)	56 (32 to 92)	0.6 (0.3 to 1.1)	56 (32 to 99)	0.5 (0.3 to 1)	65 (38 to 119)
	DALYs	Both	26.3 (13.8 to 45)	5689 (1700 to 11871)	21.9 (13.1 to 34.7)	5591 (1978 to 10710)	17 (10.8 to 26.8)	5042 (2338 to 8757)	14.8 (9.4 to 23.6)	4847 (2710 to 8247)

S11 Table. Age-standardized incidence, prevalence, deaths, Disability-Adjusted Life Years (DALYs), Years of Life Lost (YLLs) and Years Lived with Disability (YLDs) rates and number in twenty-one countries of North Africa and the Middle East in 1990, 1997, 2007, and 2019 according to sex

Country	Measure	Sex	1990		1997		2007		2019	
			Rate†	Number‡	Rate	Number	Rate	Number	Rate	Number
		Female	25.9 (10.6 to 55.7)	3051 (625 to 7906)	20.6 (9.8 to 40.7)	2877 (735 to 6978)	14.9 (8.4 to 25.8)	2361 (850 to 5139)	12.9 (7.5 to 20.9)	2075 (1022 to 3746)
		Male	27 (14.4 to 46.5)	2638 (945 to 5483)	23.2 (13.6 to 38.4)	2714 (1113 to 5268)	19 (10.7 to 33.1)	2680 (1270 to 4837)	16.7 (9.5 to 30.4)	2772 (1414 to 5177)
	YLLs	Both	26.2 (13.7 to 44.9)	5676 (1690 to 11862)	21.8 (13 to 34.6)	5575 (1962 to 10692)	16.9 (10.6 to 26.6)	5020 (2316 to 8741)	14.7 (9.3 to 23.5)	4815 (2672 to 8226)
		Female	25.8 (10.5 to 55.6)	3046 (621 to 7900)	20.5 (9.7 to 40.6)	2870 (729 to 6972)	14.8 (8.2 to 25.7)	2352 (839 to 5131)	12.8 (7.4 to 20.8)	2062 (1008 to 3734)
		Male	26.8 (14.3 to 46.4)	2631 (938 to 5475)	23.1 (13.5 to 38.2)	2705 (1103 to 5259)	18.9 (10.6 to 33)	2668 (1257 to 4826)	16.6 (9.4 to 30.3)	2753 (1399 to 5156)
	YLDs	Both	0.1 (0.1 to 0.2)	13 (8 to 18)	0.1 (0.1 to 0.2)	16 (11 to 23)	0.1 (0.1 to 0.2)	21 (14 to 30)	0.1 (0.1 to 0.2)	31 (20 to 44)
		Female	0.1 (0.1 to 0.1)	5 (4 to 8)	0.1 (0.1 to 0.1)	7 (4 to 10)	0.1 (0.1 to 0.1)	9 (6 to 13)	0.1 (0.1 to 0.2)	13 (8 to 18)
		Male	0.1 (0.1 to 0.2)	7 (5 to 10)	0.1 (0.1 to 0.2)	9 (6 to 13)	0.1 (0.1 to 0.2)	12 (8 to 17)	0.1 (0.1 to 0.2)	18 (12 to 26)
**Syrian Arab Republic**	Incidence	Both	9.5 (7.6 to 11.5)	1081 (845 to 1381)	9.9 (8.1 to 12)	1343 (1056 to 1680)	11.5 (9.5 to 14)	1987 (1561 to 2463)	13.3 (10.9 to 16.2)	1817 (1450 to 2247)
		Female	8.5 (6.8 to 10.3)	480 (372 to 618)	8.9 (7.2 to 10.7)	589 (460 to 746)	10.3 (8.4 to 12.5)	855 (667 to 1062)	12.1 (9.8 to 14.7)	818 (649 to 1018)

S11 Table. Age-standardized incidence, prevalence, deaths, Disability-Adjusted Life Years (DALYs), Years of Life Lost (YLLs) and Years Lived with Disability (YLDs) rates and number in twenty-one countries of North Africa and the Middle East in 1990, 1997, 2007, and 2019 according to sex

Country	Measure	Sex	1990		1997		2007		2019	
			Rate†	Number‡	Rate	Number	Rate	Number	Rate	Number
		Male	10.5 (8.4 to 12.7)	602 (468 to 759)	11 (9 to 13.2)	754 (595 to 935)	12.7 (10.4 to 15.4)	1132 (891 to 1394)	14.7 (11.9 to 17.8)	999 (802 to 1237)
	Prevalence	Both	1.5 (1.3 to 1.8)	125 (104 to 152)	1.6 (1.4 to 1.9)	157 (132 to 187)	1.8 (1.5 to 2.1)	235 (197 to 278)	1.9 (1.7 to 2.3)	245 (208 to 290)
		Female	1.3 (1.1 to 1.6)	55 (45 to 66)	1.4 (1.2 to 1.6)	68 (56 to 81)	1.6 (1.3 to 1.8)	100 (83 to 118)	1.7 (1.5 to 2)	109 (92 to 129)
		Male	1.7 (1.4 to 2)	70 (59 to 85)	1.8 (1.5 to 2.1)	89 (75 to 106)	2 (1.7 to 2.3)	135 (113 to 161)	2.2 (1.8 to 2.6)	136 (114 to 162)
	Deaths	Both	0.4 (0.3 to 0.5)	27 (18 to 36)	0.4 (0.3 to 0.5)	30 (21 to 39)	0.3 (0.2 to 0.4)	32 (22 to 39)	0.3 (0.2 to 0.4)	37 (24 to 52)
		Female	0.3 (0.2 to 0.5)	11 (7 to 16)	0.4 (0.3 to 0.5)	13 (8 to 17)	0.3 (0.2 to 0.4)	13 (8 to 16)	0.3 (0.2 to 0.4)	16 (10 to 22)
		Male	0.4 (0.3 to 0.6)	16 (10 to 23)	0.4 (0.3 to 0.6)	18 (11 to 24)	0.3 (0.2 to 0.5)	19 (12 to 25)	0.4 (0.2 to 0.5)	21 (12 to 31)
	DALYs	Both	12.6 (8.2 to 17)	1235 (765 to 1766)	11.9 (8 to 15.4)	1330 (906 to 1736)	8.9 (6.2 to 11.1)	1269 (898 to 1567)	9 (5.8 to 12.4)	1224 (761 to 1743)
		Female	10.5 (6.3 to 15.6)	511 (278 to 817)	10.5 (6.5 to 14.6)	570 (338 to 815)	7.7 (5 to 9.8)	504 (327 to 641)	7.8 (4.9 to 10.9)	526 (302 to 763)
		Male	14.5 (8.8 to 20.8)	725 (428 to 1138)	13.3 (8.3 to 18.1)	761 (496 to 1034)	10.2 (6.5 to 13.3)	765 (511 to 988)	10.3 (6.1 to 14.8)	698 (408 to 1003)

S11 Table. Age-standardized incidence, prevalence, deaths, Disability-Adjusted Life Years (DALYs), Years of Life Lost (YLLs) and Years Lived with Disability (YLDs) rates and number in twenty-one countries of North Africa and the Middle East in 1990, 1997, 2007, and 2019 according to sex

Country	Measure	Sex	1990		1997		2007		2019	
			Rate†	Number‡	Rate	Number	Rate	Number	Rate	Number
	YLLs	Both	12.5 (8 to 16.9)	1227 (756 to 1757)	11.8 (7.9 to 15.2)	1320 (898 to 1724)	8.8 (6.1 to 10.9)	1253 (885 to 1551)	8.8 (5.7 to 12.3)	1207 (743 to 1729)
		Female	10.4 (6.2 to 15.5)	507 (274 to 813)	10.4 (6.4 to 14.5)	565 (333 to 811)	7.6 (4.9 to 9.7)	497 (320 to 636)	7.7 (4.8 to 10.7)	519 (293 to 756)
		Male	14.3 (8.7 to 20.7)	720 (422 to 1134)	13.2 (8.2 to 18)	754 (490 to 1028)	10.1 (6.4 to 13.2)	756 (502 to 978)	10.2 (6 to 14.6)	688 (397 to 995)
	YLDs	Both	0.1 (0.1 to 0.2)	9 (6 to 12)	0.1 (0.1 to 0.2)	11 (7 to 15)	0.1 (0.1 to 0.2)	16 (10 to 23)	0.1 (0.1 to 0.2)	17 (11 to 25)
		Female	0.1 (0.1 to 0.1)	4 (2 to 5)	0.1 (0.1 to 0.1)	5 (3 to 7)	0.1 (0.1 to 0.2)	7 (4 to 10)	0.1 (0.1 to 0.2)	8 (5 to 11)
		Male	0.1 (0.1 to 0.2)	5 (3 to 7)	0.1 (0.1 to 0.2)	6 (4 to 9)	0.1 (0.1 to 0.2)	9 (6 to 13)	0.2 (0.1 to 0.2)	10 (6 to 14)
**Tunisia**	Incidence	Both	10.8 (9 to 12.9)	782 (637 to 952)	12.1 (10.1 to 14.3)	991 (822 to 1184)	13.8 (11.5 to 16.3)	1307 (1085 to 1562)	15.6 (13 to 18.7)	1823 (1502 to 2192)
		Female	10.1 (8.4 to 12.1)	354 (287 to 433)	11.3 (9.5 to 13.4)	454 (375 to 542)	13.1 (11 to 15.5)	612 (508 to 731)	14.7 (12.3 to 17.6)	863 (713 to 1044)
		Male	11.5 (9.5 to 13.8)	428 (347 to 520)	12.8 (10.6 to 15.3)	537 (440 to 643)	14.4 (12.1 to 17.2)	695 (573 to 838)	16.4 (13.6 to 19.6)	960 (788 to 1159)
	Prevalence	Both	2.4 (2 to 2.8)	136 (116 to 159)	2.5 (2.2 to 3)	178 (152 to 208)	2.7 (2.3 to 3.2)	236 (204 to 274)	2.9 (2.5 to 3.4)	344 (297 to 402)

S11 Table. Age-standardized incidence, prevalence, deaths, Disability-Adjusted Life Years (DALYs), Years of Life Lost (YLLs) and Years Lived with Disability (YLDs) rates and number in twenty-one countries of North Africa and the Middle East in 1990, 1997, 2007, and 2019 according to sex

Country	Measure	Sex	1990		1997		2007		2019	
			Rate†	Number‡	Rate	Number	Rate	Number	Rate	Number
		Female	2.3 (2 to 2.8)	64 (55 to 75)	2.5 (2.1 to 2.9)	86 (73 to 100)	2.7 (2.3 to 3.1)	117 (101 to 137)	2.9 (2.5 to 3.4)	175 (150 to 205)
		Male	2.4 (2 to 2.8)	71 (61 to 84)	2.6 (2.2 to 3)	92 (79 to 108)	2.7 (2.3 to 3.2)	119 (103 to 139)	3 (2.5 to 3.4)	170 (145 to 199)
	Deaths	Both	0.6 (0.5 to 0.8)	31 (24 to 39)	0.6 (0.5 to 0.8)	38 (30 to 50)	0.6 (0.4 to 0.8)	47 (33 to 63)	0.5 (0.4 to 0.7)	58 (41 to 79)
		Female	0.6 (0.4 to 0.8)	15 (10 to 21)	0.6 (0.4 to 0.9)	19 (13 to 26)	0.6 (0.4 to 0.8)	23 (15 to 33)	0.5 (0.3 to 0.7)	30 (19 to 44)
		Male	0.6 (0.4 to 0.8)	16 (11 to 21)	0.6 (0.4 to 0.8)	19 (14 to 27)	0.6 (0.4 to 0.8)	23 (15 to 33)	0.5 (0.3 to 0.7)	28 (19 to 39)
	DALYs	Both	17.5 (13.1 to 22.8)	1284 (845 to 1777)	16.7 (12.5 to 21.4)	1322 (961 to 1713)	14.9 (10.3 to 20.2)	1396 (976 to 1917)	12.5 (8.9 to 17.2)	1528 (1082 to 2108)
		Female	18 (11.5 to 25.2)	661 (352 to 1038)	16.6 (10.9 to 21.8)	648 (403 to 869)	14 (9 to 19.1)	653 (424 to 901)	11.7 (7.6 to 17)	727 (476 to 1071)
		Male	17 (12 to 23.7)	623 (400 to 946)	16.8 (11.8 to 23.4)	674 (455 to 955)	15.7 (10.1 to 22.5)	743 (474 to 1076)	13.4 (8.8 to 18.8)	801 (525 to 1129)
	YLLs	Both	17.3 (12.9 to 22.6)	1274 (835 to 1764)	16.5 (12.3 to 21.3)	1308 (949 to 1700)	14.7 (10.1 to 20)	1378 (957 to 1898)	12.3 (8.6 to 17)	1501 (1055 to 2087)
		Female	17.8 (11.2 to 25)	656 (347 to 1034)	16.4 (10.7 to 21.7)	641 (397 to 862)	13.8 (8.8 to 18.9)	644 (414 to 894)	11.5 (7.4 to 16.7)	714 (464 to 1058)

S11 Table. Age-standardized incidence, prevalence, deaths, Disability-Adjusted Life Years (DALYs), Years of Life Lost (YLLs) and Years Lived with Disability (YLDs) rates and number in twenty-one countries of North Africa and the Middle East in 1990, 1997, 2007, and 2019 according to sex

Country	Measure	Sex	1990		1997		2007		2019	
			Rate†	Number‡	Rate	Number	Rate	Number	Rate	Number
		Male	16.9 (11.8 to 23.5)	618 (395 to 940)	16.6 (11.6 to 23.1)	667 (446 to 949)	15.5 (9.9 to 22.3)	734 (466 to 1064)	13.2 (8.5 to 18.7)	788 (512 to 1118)
	YLDs	Both	0.2 (0.1 to 0.3)	10 (7 to 15)	0.2 (0.1 to 0.3)	14 (9 to 19)	0.2 (0.1 to 0.3)	18 (12 to 25)	0.2 (0.2 to 0.3)	26 (18 to 38)
		Female	0.2 (0.1 to 0.3)	5 (3 to 7)	0.2 (0.1 to 0.3)	7 (4 to 9)	0.2 (0.1 to 0.3)	9 (6 to 13)	0.2 (0.1 to 0.3)	14 (9 to 19)
		Male	0.2 (0.1 to 0.3)	5 (4 to 8)	0.2 (0.1 to 0.3)	7 (5 to 10)	0.2 (0.1 to 0.3)	9 (6 to 13)	0.2 (0.1 to 0.3)	13 (9 to 19)
Türkiye	Incidence	Both	15.5 (13.3 to 18)	7421 (6294 to 8790)	16.4 (14.1 to 18.8)	9134 (7812 to 10693)	17.2 (14.8 to 19.8)	11462 (9820 to 13314)	19 (16.1 to 22.2)	15709 (13143 to 18382)
		Female	13.6 (11.6 to 15.8)	3224 (2700 to 3827)	14.4 (12.4 to 16.8)	3999 (3395 to 4698)	15.9 (13.6 to 18.4)	5345 (4588 to 6198)	18.5 (15.5 to 21.6)	7851 (6565 to 9170)
		Male	17.4 (14.8 to 20.2)	4197 (3531 to 4980)	18.2 (15.7 to 20.9)	5135 (4371 to 6018)	18.4 (15.8 to 21.5)	6117 (5197 to 7200)	19.4 (16.1 to 22.7)	7858 (6547 to 9307)
	Prevalence	Both	5.9 (5 to 7.1)	2239 (1897 to 2656)	6 (5.1 to 7.3)	2849 (2408 to 3386)	6.7 (6.1 to 7.4)	4090 (3712 to 4515)	5.7 (5.1 to 6.3)	4868 (4370 to 5405)
		Female	7 (5.7 to 8.5)	1364 (1124 to 1654)	7.2 (5.9 to 8.7)	1743 (1446 to 2101)	8.2 (7.3 to 9.1)	2668 (2400 to 2969)	7.1 (6.4 to 8)	3240 (2880 to 3624)
		Male	4.6 (3.9 to 5.6)	874 (748 to 1018)	4.8 (4.1 to 5.7)	1106 (945 to 1292)	4.9 (4.4 to 5.4)	1422 (1288 to 1573)	4.1 (3.7 to 4.5)	1628 (1455 to 1813)

S11 Table. Age-standardized incidence, prevalence, deaths, Disability-Adjusted Life Years (DALYs), Years of Life Lost (YLLs) and Years Lived with Disability (YLDs) rates and number in twenty-one countries of North Africa and the Middle East in 1990, 1997, 2007, and 2019 according to sex

Country	Measure	Sex	1990		1997		2007		2019	
			Rate†	Number‡	Rate	Number	Rate	Number	Rate	Number
	Deaths	Both	1.3 (0.9 to 1.7)	485 (339 to 606)	1.2 (0.8 to 1.6)	552 (380 to 690)	0.9 (0.6 to 1.2)	524 (351 to 699)	0.8 (0.6 to 1.1)	707 (515 to 901)
		Female	1.3 (0.7 to 1.7)	238 (140 to 319)	1.2 (0.7 to 1.5)	267 (154 to 353)	0.9 (0.5 to 1.3)	292 (171 to 413)	0.9 (0.6 to 1.2)	407 (258 to 533)
		Male	1.4 (0.9 to 1.8)	248 (174 to 319)	1.3 (0.9 to 1.7)	285 (195 to 370)	0.8 (0.6 to 1.1)	232 (160 to 310)	0.7 (0.5 to 1)	301 (212 to 395)
	DALYs	Both	35.7 (25.2 to 44.1)	15690 (11483 to 19380)	32.5 (23 to 40)	17034 (12355 to 20939)	21.6 (15.4 to 27.7)	14162 (10067 to 17998)	19.6 (14.6 to 24.7)	17294 (12707 to 21921)
		Female	32.5 (19.4 to 43.8)	7022 (4161 to 9596)	29 (17.1 to 38)	7519 (4512 to 9883)	21.2 (13.3 to 28.8)	7166 (4449 to 9639)	19.8 (12.6 to 25.8)	9058 (5648 to 11863)
		Male	38.7 (27.8 to 49.6)	8669 (6248 to 11145)	35.9 (25.5 to 45.8)	9514 (6833 to 12046)	21.7 (15.6 to 28.7)	6996 (5026 to 9342)	19.1 (13.9 to 25.1)	8236 (5966 to 10880)
	YLLs	Both	35.2 (24.7 to 43.4)	15510 (11283 to 19206)	32 (22.5 to 39.5)	16804 (12154 to 20661)	21 (14.8 to 27.1)	13835 (9724 to 17644)	19.2 (14.1 to 24.3)	16908 (12295 to 21562)
		Female	31.9 (18.7 to 43.2)	6909 (4040 to 9487)	28.4 (16.5 to 37.5)	7377 (4350 to 9753)	20.5 (12.7 to 27.9)	6950 (4273 to 9407)	19.2 (12.1 to 25.2)	8799 (5382 to 11587)
		Male	38.4 (27.3 to 49.1)	8600 (6171 to 11062)	35.5 (25.1 to 45.4)	9427 (6768 to 11991)	21.3 (15.2 to 28.3)	6884 (4887 to 9194)	18.8 (13.6 to 24.8)	8109 (5850 to 10758)
	YLDs	Both	0.5 (0.3 to 0.7)	181 (119 to 262)	0.5 (0.3 to 0.7)	230 (150 to 337)	0.5 (0.4 to 0.8)	327 (221 to 463)	0.5 (0.3 to 0.6)	386 (257 to 547)

S11 Table. Age-standardized incidence, prevalence, deaths, Disability-Adjusted Life Years (DALYs), Years of Life Lost (YLLs) and Years Lived with Disability (YLDs) rates and number in twenty-one countries of North Africa and the Middle East in 1990, 1997, 2007, and 2019 according to sex

Country	Measure	Sex	1990		1997		2007		2019	
			Rate[†]	Number[‡]	Rate	Number	Rate	Number	Rate	Number
		Female	0.6 (0.4 to 0.9)	112 (72 to 166)	0.6 (0.4 to 0.9)	143 (90 to 212)	0.7 (0.4 to 0.9)	215 (141 to 310)	0.6 (0.4 to 0.8)	260 (170 to 378)
		Male	0.4 (0.2 to 0.5)	68 (46 to 98)	0.4 (0.2 to 0.6)	87 (57 to 123)	0.4 (0.3 to 0.5)	112 (75 to 156)	0.3 (0.2 to 0.4)	126 (86 to 176)
United Arab Emirates	Incidence	Both	11.6 (9.6 to 13.8)	168 (128 to 207)	12.6 (10.5 to 14.7)	256 (198 to 315)	13.8 (11.5 to 16.2)	528 (399 to 671)	14.8 (12.2 to 17.5)	1153 (850 to 1500)
		Female	10.4 (8.6 to 12.3)	52 (39 to 66)	11.4 (9.6 to 13.3)	72 (56 to 90)	12.6 (10.7 to 14.7)	127 (98 to 155)	13.4 (11 to 15.9)	249 (188 to 316)
		Male	12.1 (10 to 14.5)	116 (86 to 145)	13.2 (11 to 15.4)	183 (141 to 231)	14.2 (11.8 to 16.7)	401 (299 to 522)	15.4 (12.8 to 18.1)	904 (656 to 1195)
	Prevalence	Both	2.4 (2 to 2.8)	23 (19 to 27)	2.5 (2.1 to 2.9)	35 (29 to 42)	2.5 (2.1 to 2.8)	72 (58 to 87)	2.6 (2.3 to 3.1)	157 (125 to 191)
		Female	2.4 (2 to 2.8)	8 (6 to 9)	2.5 (2.1 to 2.9)	10 (9 to 13)	2.5 (2.1 to 2.9)	18 (15 to 22)	2.6 (2.2 to 3.1)	37 (30 to 44)
		Male	2.4 (2 to 2.8)	16 (13 to 19)	2.5 (2.1 to 2.9)	25 (20 to 30)	2.5 (2.1 to 2.9)	54 (43 to 65)	2.7 (2.3 to 3.1)	120 (95 to 147)
	Deaths	Both	0.7 (0.5 to 1)	5 (3 to 7)	0.8 (0.5 to 1.1)	7 (5 to 11)	0.8 (0.4 to 1.1)	14 (10 to 19)	0.5 (0.3 to 0.8)	33 (21 to 52)
		Female	0.7 (0.4 to 1.1)	1 (1 to 2)	0.9 (0.5 to 1.4)	2 (1 to 3)	1.1 (0.5 to 1.6)	3 (2 to 4)	0.5 (0.3 to 0.8)	5 (3 to 9)

S11 Table. Age-standardized incidence, prevalence, deaths, Disability-Adjusted Life Years (DALYs), Years of Life Lost (YLLs) and Years Lived with Disability (YLDs) rates and number in twenty-one countries of North Africa and the Middle East in 1990, 1997, 2007, and 2019 according to sex

Country	Measure	Sex	1990		1997		2007		2019	
			Rate†	Number‡	Rate	Number	Rate	Number	Rate	Number
		Male	0.7 (0.4 to 1)	3 (2 to 6)	0.7 (0.4 to 1)	6 (4 to 9)	0.6 (0.4 to 0.9)	11 (8 to 16)	0.5 (0.3 to 0.8)	27 (16 to 44)
	DALYs	Both	19.3 (13.5 to 27.2)	238 (158 to 365)	20.4 (13.7 to 28.2)	345 (245 to 506)	19.6 (12.2 to 26.1)	672 (476 to 936)	15.3 (9.9 to 21.8)	1459 (890 to 2336)
		Female	17.9 (10.2 to 28.4)	63 (33 to 109)	20.1 (11.5 to 29.9)	82 (49 to 117)	21.1 (11 to 30.8)	119 (77 to 160)	12.5 (7.6 to 18.5)	229 (140 to 348)
		Male	19.7 (13.1 to 30.5)	175 (106 to 291)	20.2 (13.4 to 29)	263 (173 to 412)	18.1 (11.6 to 24.1)	553 (372 to 798)	16.4 (10.2 to 24.3)	1230 (721 to 2056)
	YLLs	Both	19.1 (13.3 to 27)	236 (157 to 364)	20.2 (13.5 to 28)	343 (242 to 504)	19.4 (11.9 to 25.9)	666 (471 to 931)	15.1 (9.7 to 21.7)	1448 (881 to 2328)
		Female	17.7 (10 to 28.2)	63 (32 to 108)	19.9 (11.3 to 29.6)	81 (48 to 116)	20.9 (10.8 to 30.6)	117 (75 to 159)	12.3 (7.3 to 18.3)	226 (137 to 346)
		Male	19.5 (12.9 to 30.3)	174 (105 to 290)	20 (13.1 to 28.8)	262 (172 to 410)	17.9 (11.3 to 24)	549 (368 to 794)	16.2 (10 to 24.1)	1222 (712 to 2047)
	YLDs	Both	0.2 (0.1 to 0.3)	2 (1 to 2)	0.2 (0.1 to 0.3)	3 (2 to 4)	0.2 (0.1 to 0.3)	5 (3 to 7)	0.2 (0.1 to 0.3)	11 (7 to 16)
		Female	0.2 (0.1 to 0.3)	1 (0 to 1)	0.2 (0.1 to 0.3)	1 (0 to 1)	0.2 (0.1 to 0.3)	1 (1 to 2)	0.2 (0.1 to 0.3)	3 (2 to 4)
		Male	0.2 (0.1 to 0.3)	1 (1 to 2)	0.2 (0.1 to 0.3)	2 (1 to 3)	0.2 (0.1 to 0.3)	4 (2 to 6)	0.2 (0.1 to 0.3)	8 (5 to 12)

S11 Table. Age-standardized incidence, prevalence, deaths, Disability-Adjusted Life Years (DALYs), Years of Life Lost (YLLs) and Years Lived with Disability (YLDs) rates and number in twenty-one countries of the North Africa and the Middle East in 1990, 1997, 2007 and 2019 according to sex

Country	Measure	Sex	1990		1997		2007		2019	
			Rate†	Number‡	Rate	Number	Rate	Number	Rate	Number
Yemen	Incidence	Both	8.1 (6.6 to 9.9)	975 (752 to 1255)	8 (6.6 to 9.6)	1172 (916 to 1490)	9.1 (7.4 to 11)	1782 (1403 to 2239)	10.8 (8.8 to 13.1)	2868 (2259 to 3570)
		Female	7.3 (6 to 8.9)	443 (338 to 573)	7.2 (5.8 to 8.7)	523 (410 to 668)	8.1 (6.6 to 9.9)	782 (617 to 992)	9.8 (8 to 11.9)	1275 (1006 to 1604)
		Male	8.9 (7.3 to 10.8)	532 (407 to 682)	8.8 (7.2 to 10.5)	650 (501 to 831)	10 (8.2 to 12)	1001 (781 to 1258)	11.8 (9.5 to 14.3)	1593 (1255 to 1988)
	Prevalence	Both	1.3 (1.1 to 1.5)	101 (84 to 122)	1.3 (1.1 to 1.5)	126 (105 to 151)	1.4 (1.2 to 1.6)	190 (159 to 227)	1.6 (1.3 to 1.8)	307 (257 to 366)
		Female	1.1 (0.9 to 1.3)	45 (38 to 55)	1.1 (1 to 1.3)	55 (46 to 67)	1.2 (1 to 1.4)	82 (68 to 98)	1.4 (1.2 to 1.6)	133 (111 to 160)
		Male	1.5 (1.2 to 1.7)	55 (46 to 67)	1.5 (1.3 to 1.7)	70 (59 to 84)	1.6 (1.3 to 1.8)	108 (91 to 130)	1.8 (1.5 to 2.1)	174 (146 to 207)
	Deaths	Both	0.5 (0.3 to 0.8)	36 (22 to 55)	0.5 (0.3 to 0.8)	40 (26 to 62)	0.5 (0.3 to 0.8)	56 (35 to 88)	0.5 (0.3 to 0.8)	75 (48 to 120)
		Female	0.4 (0.3 to 0.7)	17 (9 to 28)	0.4 (0.2 to 0.7)	19 (10 to 31)	0.4 (0.3 to 0.8)	25 (14 to 42)	0.4 (0.3 to 0.7)	34 (19 to 53)
		Male	0.6 (0.4 to 1)	19 (11 to 30)	0.6 (0.3 to 0.9)	22 (13 to 36)	0.6 (0.4 to 0.9)	31 (19 to 51)	0.5 (0.3 to 0.9)	42 (26 to 69)
	DALYs	Both	15.8 (10.3 to 23)	1871 (884 to 3414)	14.6 (9.6 to 21.5)	2000 (1042 to 3626)	14.8 (9.5 to 22.8)	2665 (1444 to 4606)	14 (8.9 to 22.1)	3145 (1881 to 5358)

S11 Table. Age-standardized incidence, prevalence, deaths, Disability-Adjusted Life Years (DALYs), Years of Life Lost (YLLs) and Years Lived with Disability (YLDs) rates and number in twenty-one countries of North Africa and the Middle East in 1990, 1997, 2007, and 2019 according to sex

Country	Measure	Sex	1990		1997		2007		2019	
			Rate†	Number‡	Rate	Number	Rate	Number	Rate	Number
		Female	14 (8.1 to 21.1)	935 (360 to 1900)	12.8 (7.5 to 19.5)	966 (419 to 1980)	13 (7.4 to 20.5)	1241 (546 to 2318)	12.3 (7.1 to 19.4)	1412 (715 to 2435)
		Male	17.8 (10.8 to 27.8)	936 (457 to 1797)	16.4 (10.3 to 26.3)	1034 (549 to 2087)	16.7 (10.3 to 27)	1425 (743 to 2587)	15.7 (9.9 to 25.8)	1732 (998 to 3055)
	YLLs	Both	15.7 (10.2 to 22.9)	1864 (877 to 3406)	14.5 (9.5 to 21.4)	1991 (1033 to 3619)	14.7 (9.5 to 22.8)	2652 (1428 to 4592)	13.9 (8.7 to 22)	3124 (1862 to 5332)
		Female	13.9 (8 to 21)	931 (356 to 1896)	12.8 (7.4 to 19.4)	963 (415 to 1976)	12.9 (7.3 to 20.4)	1235 (541 to 2311)	12.2 (7 to 19.3)	1403 (707 to 2425)
		Male	17.7 (10.7 to 27.7)	932 (452 to 1793)	16.3 (10.2 to 26.1)	1029 (543 to 2083)	16.6 (10.2 to 26.9)	1417 (738 to 2580)	15.5 (9.8 to 25.6)	1720 (987 to 3043)
	YLDs	Both	0.1 (0.1 to 0.1)	7 (4 to 10)	0.1 (0.1 to 0.1)	9 (6 to 12)	0.1 (0.1 to 0.1)	13 (8 to 19)	0.1 (0.1 to 0.2)	21 (13 to 30)
		Female	0.1 (0.1 to 0.1)	3 (2 to 4)	0.1 (0.1 to 0.1)	4 (2 to 5)	0.1 (0.1 to 0.1)	6 (4 to 8)	0.1 (0.1 to 0.1)	9 (6 to 13)
		Male	0.1 (0.1 to 0.2)	4 (2 to 5)	0.1 (0.1 to 0.2)	5 (3 to 7)	0.1 (0.1 to 0.2)	7 (5 to 11)	0.1 (0.1 to 0.2)	12 (8 to 17)

† Age-standardized rate per 100,000
‡ All ages number
Data in parentheses are 95% uncertainty intervals (95% UI)
YLLs= years of life lost; YLDs= years lived with disability; DALYs=disability-adjusted life years

S12 Table. Summary of the highest and lowest epidemiologic measures and burden of infective endocarditis in North Africa and the Middle East at a glance

	ASIR	PC-ASIR (absolute value of percentage)	ASPR	PC-ASPR (absolute value of percentage)	ASDR	PC-ASDR (absolute value of percentage)	ASR-DALY	PC-ASR-DALY (absolute value of percentage)	ASR-YLL	PC-ASR-YLL (absolute value of percentage)	ASR-YLD	PC-ASR-YLD (absolute value of percentage)
**Highest**	Türkiye	Saudi Arabia	Türkiye	Oman	Türkiye	Jordan	Egypt	Libya	Egypt	Libya	Türkiye	Oman
**Lowest**	Afghanistan	Jordan	Afghanistan	Türkiye	Palestine	Iraq and Oman	Palestine	Afghanistan	Palestine	Afghanistan	Afghanistan	Türkiye and Jordan

ASIR: age-standardized incidence rate, ASPR: age-standardized prevalence rate, ASDR: age-standardized death rate, ASR: age-standardized rate, DALY: disability-adjusted life years, PC: percent change, YLL: years of life lost, YLD: years lived with disability

S13 Table. Predisposing conditions and risk factors for infective endocarditis

Patients with underlying cardiac disease	Patients without underlying heart disease
**High-risk**	Intravenous drug user
Prosthetic cardiac valves	Indwelling central venous catheter
Past history of endocarditis	Primary and secondary immune deficiency
Complex cyanotic congenital heart disease	Diabetes mellitus
Surgically- created systemic to pulmonary artery shunt	Systemic lupus erythematosus
**Moderate-risk**	Patients with shunts for hemodialysis
Untreated left to right shunt across patent ductus arteriosus, ventricular septal defect or coronary sinus type and primum types of atrial septal defects (the more the turbulence of blood flow, the higher the risk of endocarditis)	Inflammatory bowel disease
Bicuspid aortic valve	Chronic kidney disease
Prolapse of the mitral valve with mitral regurgitation	Age > 60 years
Rheumatic heart disease	
Hypertrophic cardiomyopathy	
**Others**	
Transvenous indwelling pacemaker	
Intracardiac pacemaker leads	
Degenerative lesions of the heart in the elderly	
Cardiac transplantation and other solid organ transplantations	
Patients with intracardiac devices	
Patients with right ventricular to pulmonary artery valved conduits	
Transcatheter pulmonary valve replacement	
Transcatheter aortic valve implantation (TAVI)	
Left ventricular assist device (LVAD)	
Implantable cardioverter defibrillator (ICD)	
Hematopoietic stem cell transplantation (HSCT)	
