# Supplementary material for: Infective Endocarditis in North Africa and the Middle East, 1990‒2019: Updates from the Global Burden of Disease Study 2019
Source: Arch Iran Med. 2024 May 1;27(5):229–38. doi: 10.34172/aim.2024.34 (PMC11097324; doi:10.34172/aim.2024.34)
Supplement: Supplementary file 4 — List of the GBD 2019 NAME Endocarditis Collaborators and their affiliations and contribution. [file aim-27-229-s004.pdf]

S4 Appendix

GBD 2019 NAME Endocarditis Collaborators

Elaheh Malakan Rad[1¶], Sara Momtazmanesh[2,3¶], Sahar Saeedi Moghaddam[3,4¶], Negar Rezaei[3,5], NazilaRezaei[3], Mohsen Abbasi-Kangevari[3,6], Zeinab Abbasi-Kangevari[3], Hedayat Abbastabar[7], Hassan Abidi[8], Muhammad Sohail Afzal[9], Sepideh Ahmadi[10], Haroon Ahmed[11], Sami Almustanyir[12,13], Jalal Arabloo[14], Zahra Aryan[15,3], Samaneh Asgari[16], Mohammadreza Azangou-Khyavy[3,6], Sara Bagherieh[17], Akshaya Srikanth Bhagavathula[18,19], Ali Bijani[20], Ahmad Daryani[21], Muhammed Elhadi [22], Hossein Farrokhpour[2,23], Seyyed-Hadi Ghamari[3,6], Ahmad Ghashghaee[24], Laszlo Göbölös[25,26], Mohamad Golitaleb[27],Mostafa Hadei[28], Khezar Hayat[29,30], Mohammad-Salar Hosseini[31], Seyed Kianoosh Hosseini[32], Seyed Sina Naghibi Irvani[33], Elham Jamshidi[34,35], Hamid Reza Koohestani[36], Savita Lasrado[37], Ata Mahmoodpoor[38], Mohammad-Reza Malekpour[3], Yosef Manla[39], Ritesh G Menezes[40], Yousef Mohammad[41], Paula Moraga[42], Zuhair S Natto[43,44], Fatemeh Pashazadeh Kan[45], Akram Pourshams[46], Mohammad Rabiee[47], Navid Rabiee[48,49], Alireza Rafiei[50,51], Sima Rafiei[52], Samira Raoofi[53], Sina Rashedi[3,54], Sahba Rezazadeh-Khadem[3], Masoumeh Sadeghi[55], Farhad Saeedi[56], Abdallah M Samy [57],Parnian Shobeiri [2,3,58], Soraya Siabani[59,60], Majid Taheri[61,62], Hamed Tavolinejad[3,63], Sahel Valadan Tahbaz[64,65],Seyed Hossein Yahyazadeh Jabbari[64], Mazyar Zahir[66,67], Mohammad Zoladl[68], Hamid Reza Jamshidi[69,70], Mohsen Naghavi[71,72], Bagher Larijani[5], Farshad Farzadfar[3,5]*

¶ Co-first authors

These authors contributed equally to this work and shared the first authorship.

* Corresponding author

Affiliations

1. Department of Pediatric Cardiology, Tehran University of Medical Sciences, Tehran, Iran

2. School of Medicine, Tehran University of Medical Sciences, Tehran, Iran

3. Non-communicable Diseases Research Center, Tehran University of Medical Sciences, Tehran, Iran

4. Kiel Institute for the World Economy, Kiel, Germany

5. Endocrinology and Metabolism Research Institute, Tehran University of Medical Sciences, Tehran, Iran
6. Social Determinants of Health Research Center, Shahid Beheshti University of Medical Sciences, Tehran, Iran
7. Advanced Diagnostic and Interventional Radiology Research Center, Tehran University of Medical Sciences, Tehran, Iran
8. Laboratory Technology Sciences Department, Yasuj University of Medical Sciences, Yasuj, Iran
9. Department of Life Sciences, University of Management and Technology, Lahore, Pakistan
10. School of Advanced Technologies in Medicine, Shahid Beheshti University of Medical Sciences, Tehran, Iran
11. Department of Biosciences, COMSATS Institute of Information Technology, Islamabad, Pakistan
12. College of Medicine, Alfaisal University, Riyadh, Saudi Arabia
13. Ministry of Health, Riyadh, Saudi Arabia
14. Health Management and Economics Research Center, Iran University of Medical Sciences, Tehran, Iran
15. Brigham and Women's Hospital, Harvard University, Boston, MA, USA
16. Prevention of Metabolic Disorders Research Center, Shahid Beheshti University of Medical Sciences, Tehran, Iran
17. School of Medicine, Isfahan University of Medical Sciences, Isfahan, Iran
18. Department of Social and Clinical Pharmacy, Charles University, Hradec Kralova, Czech Republic
19. Institute of Public Health, United Arab Emirates University, Al Ain, United Arab Emirates
20. Social Determinants of Health Research Center, Babol University of Medical Sciences, Babol, Iran
21. Toxoplasmosis Research Center, Mazandaran University of Medical Sciences, Sari, Iran

22. Faculty of Medicine, University of Tripoli, Tripoli, Libya
23. Endocrinology and Metabolism Research Institute, Non-Communicable Diseases Research Center (NCDRC), Tehran, Iran
24. School of Public Health, Qazvin University of Medical Sciences, Qazvin, Iran
25. Heart and Vascular Institute, Cleveland Clinic Abu Dhabi, Abu Dhabi, United Arab Emirates
26. Lerner College of Medicine, Case Western Reserve University, Cleveland, OH, United States
27. Department of Nursing, Arak University of Medical Sciences, Arak, Iran
28. Department of Environmental Health Engineering, Tehran University of Medical Sciences, Tehran, Iran
29. Institute of Pharmaceutical Sciences, University of Veterinary and Animal Sciences, Lahore, Pakistan
30. Department of Pharmacy Administration and Clinical Pharmacy, Xian Jiaotong University, Xian, China
31. Student Research Committee, Tabriz University of Medical Sciences, Tabriz, Iran
32. Department of Interventional Cardiology, Hamedan University of Medical Sciences, Hamadan, Iran
33. Independent Consultant, Tabriz, Iran
34. Functional Neurosurgery Research Center, Shahid Beheshti University of Medical Sciences, Tehran, Iran
35. Division of Pulmonary Medicine, Lausanne University Hospital (CHUV), Lausanne, Switzerland
36. Social Determinants of Health Research Center, Saveh University of Medical Sciences, Saveh, Iran
37. Department of Otorhinolaryngology, Father Muller Medical College, Mangalore, India
38. Anesthesiology and Critical Care, Tabriz University of Medical Sciences, Tabriz, Iran

39. Heart and Vascular Institute, Cleveland Clinic Abu Dhabi, Abu Dhabi, United Arab Emirates

40. Forensic Medicine Division, Imam Abdulrahman Bin Faisal University, Dammam, Saudi Arabia

41. Internal Medicine Department, King Saud University, Riyadh, Saudi Arabia

42. Computer, Electrical, and Mathematical Sciences and Engineering Division, King Abdullah University of Science and Technology, Thuwal, Saudi Arabia

43. Department of Dental Public Health, King Abdulaziz University, Jeddah, Saudi Arabia

44. Department of Health Policy and Oral Epidemiology, Harvard University, Boston, MA, United States

45. Iran University of Medical Sciences, Tehran, Iran

46. Digestive Diseases Research Institute, Tehran University of Medical Sciences, Tehran, Iran

47. Biomedical Engineering Department, Amirkabir University of Technology, Tehran, Iran

48. School of Engineering, Macquarie University, Sydney, NSW, Australia

49. Pohang University of Science and Technology, South Korea

50. Department of Immunology, Mazandaran University of Medical Sciences, Sari, Iran

51. Molecular and Cell Biology Research Center, Mazandaran University of Medical Sciences, Sari, Iran

52. Social Determinants of Health Research Center, Qazvin University of Medical Sciences, Qazvin, Iran

53. Independent Consultant, Tehran, Iran

54. Department of Cardiology, Tehran University of Medical Sciences, Tehran, Iran

55. Cardiac Rehabilitation Research Center, Isfahan University of Medical Sciences, Isfahan, Iran

56. Cardiovascular Diseases Research Center, Birjand University of Medical Sciences, Birjand, Iran

57. Department of Entomology, Ain Shams University, Cairo, Egypt

58. Department of Radiology, Memorial Sloan Kettering Cancer Center (MSKCC), New York City, United States

59. Department of Health Education and Health Promotion, Kermanshah University of Medical Sciences, Kermanshah, Iran

60. School of Health, University of Technology Sydney, Sydney, NSW, Australia

61. Trauma and Injury Research Center, Iran University of Medical Sciences, Tehran, Iran

62. Medical Ethics and Law Research Center, Shahid Beheshti University of Medical Sciences, Tehran, Iran

63. Tehran Heart Center, Tehran University of Medical Sciences, Tehran, Iran

64. Clinical Cancer Research Center, Milad General Hospital, Tehran, Iran

65. Department of Microbiology, Islamic Azad University, Tehran, Iran

66. Faculty of Medicine, Islamic Azad University, Tehran, Iran

67. Department of Pharmacology, Tehran University of Medical Sciences, Tehran, Iran

68. Department of Nursing, Yasuj University of Medical Sciences, Yasuj, Iran

69. Department of Pharmacology, Shahid Beheshti University of Medical Sciences, Tehran, Iran

70. Ministry of Health and Medical Education, Tehran, Iran

71. Institute for Health Metrics and Evaluation, University of Washington, Seattle, WA, USA

72. Department of Health Metrics Sciences, School of Medicine, University of Washington, Seattle,

WA, United States

Author Contributions

Providing data or critical feedback on data sources

Hedayat Abbastabar, Hassan Abidi, Muhammad Sohail Afzal, Sepideh Ahmadi, Sami Almustanyir, Jalal Arabloo, Zahra Aryan, Sara Bagherieh, Akshaya Srikanth Bhagavathula, Ahmad Daryani, Hossein Farrokhpour, Farshad Farzadfar, Ahmad Ghashghaee, Seyed Sina Naghibi Irvani, Savita Lasrado, Ata Mahmoodpoor, Elaheh Malakan Rad, Ritesh G Menezes, Sara Momtazmanesh, Mohsen Naghavi, Zuhair S Natto, Fatemeh Pashazadeh Kan, Mohammad Rabiee, Navid Rabiee, Alireza Rafiei, Samira Raoofi, Sina Rashedi, Sahar Saeedi Moghaddam, Abdallah M Samy, Parnian Shobeiri, Soraya Siabani, Majid Taheri, Sahel Valadan Tahbaz, Seyed Hossein Yahyazadeh Jabbari, and Mohammad Zoladl.

Developing methods or computational machinery

Zahra Aryan, Mohsen Naghavi, and Sahar Saeedi Moghaddam.

Providing critical feedback on methods or results

Hedayat Abbastabar, Hassan Abidi, Muhammad Sohail Afzal, Haroon Ahmed, Sami Almustanyir, Jalal Arabloo, Zahra Aryan, Samaneh Asgari, Mohammadreza Azangou-Khyavy, Sara Bagherieh, Akshaya Srikanth Bhagavathula, Ali Bijani, Ahmad Daryani, Muhammed Elhadi, Hossein Farrokhpour, Farshad Farzadfar, Ahmad Ghashghaee, Laszlo Göbölös, Mohamad Golitaleb, Mostafa Hadei, Khezar Hayat, Mohammad-Salar Hosseini, Seyed Kianoosh Hosseini, Seyed Sina Naghibi Irvani, Elham Jamshidi, Hamid Reza Koohestani, Bagher Larijani, Savita Lasrado, Ata Mahmoodpoor, Elaheh Malakan Rad, Mohammad- Reza Malekpour, Yosef Manla, Ritesh G Menezes, Yousef Mohammad, Sara Momtazmanesh, Paula Moraga, Mohsen Naghavi, Zuhair S Natto, Fatemeh Pashazadeh Kan, Akram Pourshams, Mohammad Rabiee, Navid Rabiee, Alireza Rafiei, Sima Rafiei, Samira Raoofi, Sina Rashedi, Negar Rezaei, Sahba Rezazadeh-Khadem, Farhad Saeedi, Sahar Saeedi Moghaddam, Abdallah M Samy, Parnian Shobeiri, Soraya Siabani, Majid Taheri, Hamed

Tavolinejad, Sahel Valadan Tahbaz, Seyed Hossein Yahyazadeh Jabbari, and Mohammad Zoladl.

Drafting the work or revising is critically for important intellectual content

Mohsen Abbasi-Kangevari, Zeinab Abbasi-Kangevari, Hedayat Abbastabar, Hassan Abidi, Muhammad Sohail Afzal, Sepideh Ahmadi, Sami Almustanyir, Jalal Arabloo, Zahra Aryan, Mohammadreza Azangou- Khyavy, Sara Bagherieh, Akshaya Srikanth Bhagavathula, Ahmad Daryani, Muhammed Elhadi,Farshad Farzadfar, Seyyed-Hadi Ghamari, Ahmad Ghashghaee, Laszlo Göbölös, Mohamad Golitaleb, Mostafa Hadei, Khezar Hayat, Mohammad-Salar Hosseini, Seyed Sina Naghibi Irvani, Elham Jamshidi, Savita Lasrado, Ata Mahmoodpoor, Elaheh Malakan Rad, Mohammad-Reza Malekpour, Yosef Manla, Ritesh G Menezes, Yousef Mohammad, Sara Momtazmanesh, Mohsen Naghavi, Zuhair S Natto, Fatemeh Pashazadeh Kan, Mohammad Rabiee, Navid Rabiee, Alireza Rafiei, Samira Raoofi, Nazila Rezaei, Negar Rezaei, Sahba Rezazadeh-Khadem, Masoumeh Sadeghi, Sahar Saeedi Moghaddam, Abdallah M Samy, Parnian Shobeiri, Majid Taheri, Hamed Tavolinejad, Sahel Valadan Tahbaz, Seyed Hossein Yahyazadeh Jabbari, Mazyar Zahir, and Mohammad Zoladl.

Managing the estimation or publications process

Farshad Farzadfar, Elaheh Malakan Rad, Sara Momtazmanesh, Mohsen Naghavi, Nazila Rezaei, and Sahar Saeedi Moghaddam.3

Sina Rashedi, Sahar Saeedi Moghaddam, Abdallah M Samy, Parnian Shobeiri, Soraya Siabani, Majid Taheri, Sahel Valadan Tahbaz, Seyed Hossein Yahyazadeh Jabbari, and Mohammad Zoladl.

Developing methods or computational machinery

Zahra Aryan, Mohsen Naghavi, and Sahar Saeedi Moghaddam.

Providing critical feedback on methods or results

Hedayat Abbastabar, Hassan Abidi, Muhammad Sohail Afzal, Haroon Ahmed, Sami Almustanyir, Jalal Arabloo, Zahra Aryan, Samaneh Asgari, Mohammadreza Azangou-Khyavy, Sara Bagherieh, Akshaya Srikanth Bhagavathula, Ali Bijani, Ahmad Daryani, Muhammed Elhadi, Hossein Farrokhpour, Farshad Farzadfar, Ahmad Ghashghaee, Laszlo Göbölös, Mohamad Golitaleb, Mostafa Hadei, Khezar Hayat, Mohammad-Salar Hosseini, Seyed Kianoosh Hosseini, Seyed Sina Naghibi Irvani, Elham Jamshidi, Hamid Reza Koohestani, Bagher Larijani, Savita Lasrado, Ata Mahmoodpoor, Elaheh Malakan Rad, Mohammad- Reza Malekpour, Yosef Manla, Ritesh G Menezes, Yousef Mohammad, Sara Momtazmanesh, Paula Moraga, Mohsen Naghavi, Zuhair S Natto, Fatemeh Pashazadeh Kan, Akram Pourshams, Mohammad Rabiee, Navid Rabiee, Alireza Rafiei, Sima Rafiei, Samira Raoofi, Sina Rashedi, Negar Rezaei, Sahba Rezazadeh-Khadem, Farhad Saeedi, Sahar Saeedi Moghaddam, Abdallah M Samy, Parnian Shobeiri, Soraya Siabani, Majid Taheri, Hamed Tavolinejad, Sahel Valadan Tahbaz, Seyed Hossein Yahyazadeh Jabbari, and Mohammad Zoladl.

Drafting the work or revising is critically for important intellectual content

Mohsen Abbasi-Kangevari, Zeinab Abbasi-Kangevari, Hedayat Abbastabar, Hassan Abidi, Muhammad Sohail Afzal, Sepideh Ahmadi, Sami Almustanyir, Jalal Arabloo, Zahra Aryan, Mohammadreza Azangou- Khyavy, Sara Bagherieh, Akshaya Srikanth Bhagavathula, Ahmad Daryani, Muhammed Elhadi,Farshad Farzadfar, Seyyed-Hadi Ghamari, Ahmad Ghashghaee, Laszlo Göbölös, Mohamad Golitaleb, Mostafa Hadei, Khezar Hayat, Mohammad-Salar Hosseini, Seyed Sina Naghibi Irvani, Elham Jamshidi, Savita Lasrado, Ata Mahmoodpoor, Elaheh Malakan Rad, Mohammad-Reza Malekpour, Yosef Manla, Ritesh G Menezes, Yousef Mohammad, Sara Momtazmanesh, Mohsen Naghavi, Zuhair S Natto, Fatemeh Pashazadeh Kan, Mohammad Rabiee, Navid Rabiee, Alireza Rafiei, Samira Raoofi, Nazila Rezaei, Negar Rezaei, Sahba Rezazadeh-Khadem, Masoumeh Sadeghi, Sahar Saeedi Moghaddam, Abdallah

M Samy, Parnian Shobeiri, Majid Taheri, Hamed Tavolinejad, Sahel Valadan Tahbaz, Seyed Hossein Yahyazadeh Jabbari, Mazyar Zahir, and Mohammad Zoladl.

Managing the estimation or publications process

Farshad Farzadfar, Elaheh Malakan Rad, Sara Momtazmanesh, Mohsen Naghavi, Nazila Rezaei, and Sahar Saeedi Moghaddam.
